# Causal role of immune cells in chronic periodontitis: a bidirectional Mendelian randomization study

**DOI:** 10.1186/s12903-024-04592-0

**Published:** 2024-07-16

**Authors:** Yu Chen, Xinyang Jin, Qi Wang, Sai Hu, Xu Huang

**Affiliations:** Department of Stomatology, the Fourth Affiliated Hospital of School of Medicine, and International School of Medicine, International Institutes of Medicine, Zhejiang University, No.N1, Shangcheng Avenue, Yiwu City, Zhejiang Province 322000 China

**Keywords:** Chronic periodontitis, Immune cells, Immunity, Mendelian randomization, Causal inference

## Abstract

**Background:**

This study aims to explore the bidirectional causal relationship between immune cell phenotypes and chronic periodontitis using a Mendelian randomization framework.

**Materials and methods:**

Through a two-sample Mendelian randomization analysis, this research examined genetic data related to 731 immune cell traits and chronic periodontitis. Instrumental variables were chosen based on their genetic links to either immune traits or periodontitis. Various statistical techniques, including MR-Egger regression, weighted median, and inverse-variance weighted (IVW) analysis, were employed to determine the causal connections.

**Results:**

Predominantly using the IVW method, 26 distinct immune phenotypes were identified as potentially influencing periodontitis (*P* < 0.05). Conversely, periodontitis potentially affected 33 different immune phenotypes (*P* < 0.05). The results for pleiotropy and sensitivity tests were stable. However, these associations lost significance after adjusting for the False Discovery Rate.

**Conclusion:**

This study uncovers a complex bidirectional causal relationship between certain immune cell phenotypes and chronic periodontitis, underscoring the intricate interaction between the immune system and the pathogenesis of periodontal disease.

**Supplementary Information:**

The online version contains supplementary material available at 10.1186/s12903-024-04592-0.

## Introduction

Periodontitis is the most common chronic inflammatory disease affecting humans. Data from 2011 to 2020 reveal that about 62% of adults suffer from periodontitis, with a significant 23.6% experiencing severe forms of the disease [1]. Moreover, another study on the global burden of disease underscores an astonishing 99.0% increase in the global prevalence of periodontitis from 1990 to 2019 [2]. Chronic periodontitis not only leads to tooth loss and disability but also adversely impacts chewing functions and aesthetics [3]. It contributes to social inequality and significantly lowers the quality of life [4]. Additionally, periodontitis is linked with various systemic diseases, including cardiovascular and respiratory diseases, diabetes, Alzheimer’s disease, cancer, and adverse pregnancy outcomes [5]. Therefore, researching its pathogenic mechanisms is crucial for promoting early diagnosis and treatment, providing vital support in the fight against this disease.

Recent studies indicate that the interaction between periodontal pathogens and the body's immune response is critical in the development and advancement of periodontal disease [6]. Central to the body's defence against these pathogens are key immune cells such as neutrophils, macrophages, and lymphocytes. The presence of neutrophils induces the expression of RANKL, which may promote the formation of osteoclasts [7]. Polymorphonuclear neutrophils can exacerbate the progression of periodontitis by promoting inflammation and tissue damage [8]. Furthermore, lipopolysaccharide (LPS) antigens play a crucial role in the development and advancement of periodontitis by influencing immune responses and inflammatory processes. LPS from Porphyromonas gingivalis can activate toll-like receptors (TLR) in human periodontal ligament stromal cells (hPDLSCs) and gingival mesenchymal stromal cells (hGMSCs), leading to increased production of inflammatory cytokines such as IL-8, IL-6, and MCP-1 [8]. Additionally, LPS from Porphyromonas gingivalis can regulate the Th17/Treg balance and induce the maturation of dendritic cells with a CD14 + CD16 + phenotype [9, 10]. Moreover, complement activation links the attack on periodontal microbiota with the immune response. Studies have shown that patients with periodontitis exhibit increased complement activation, with elevated levels of total C3, C3dg, and C3c in saliva and plasma. Complement component C3 is also associated with inflammation and bone loss that promote periodontitis [11].

Yet, the link between periodontal disease and immune responses is intricate and influenced by various factors, such as genetic predisposition, environmental factors, and coexisting systemic conditions [12].

Mendelian randomization (MR) is an epidemiological method that employs genetic variation as an instrumental variable [13]. This approach is grounded in Mendel's laws of genetics, positing that the distribution of genetic variations within a population is random [14]. This randomness aids in discerning the causal relationship between exposure and outcomes, as opposed to mere correlations [15]. Currently, researchers have utilized Mendelian randomization to reveal associations between immune cells and various diseases, including schizophrenia, type 2 diabetes, multiple sclerosis, systemic lupus erythematosus, atrial fibrillation, and chronic obstructive pulmonary disease. This provides significant evidence for the involvement of immune responses in the pathogenesis of multiple systemic diseases [16–21].

Building on the aforementioned content, the present study aims to thoroughly investigate the causal link between chronic periodontitis and immune cell phenotypes through the application of bidirectional Mendelian randomization. To test this, we propose the following null hypotheses: 1) Immune cell phenotypes have no causal effect on chronic periodontitis. 2) Chronic periodontitis has no causal effect on immune cell phenotypes.

## Methods

### Study design and data resource

**Fig. 1:**
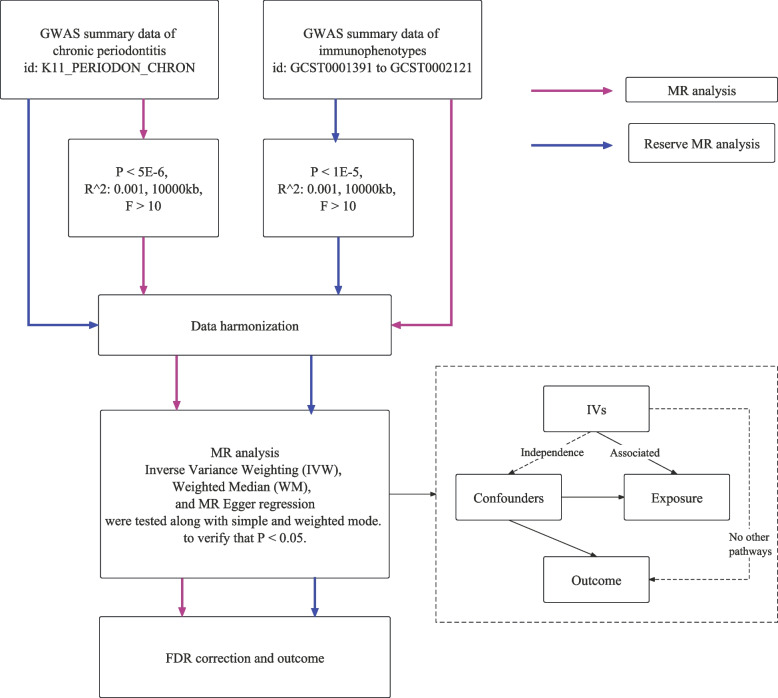
Study design and process

Based on the STROBE-MR and a two-sample MR analysis [22], this study investigates the causal relationship between 731 immune cell traits and chronic periodontitis. Mendelian randomization uses genetic variations as proxies for risk factors, requiring that effective instrumental variables (IVs) meet three crucial criteria:) All selected IVs should be highly associated with the exposure; 2) All IVs must be independent of confounders related to the exposure; 3) All selected IVs should influence the outcome only through the exposure, not by other pathways. The studies included in this analysis have received approval from appropriate institutional review committees, and all participants have given informed consent. The study aims to establish the bidirectional causal relationship between the morphology of immune cells and chronic periodontitis, treating each in turn as the exposure and the outcome. Specific details of the experimental design are presented in Fig. 1.

GWAS summary statistics for chronic periodontitis were obtained from the Finnish database (K11_PERIODON_CHRON), using its latest version (R9), which included 4,434 cases and 259,234 controls. Cases were diagnosed with chronic periodontitis following ICD-10, excluding Chronic periodontitis, complicated. GWAS summary statistics for each immune trait were publicly available from the GWAS catalog (access numbers GCST0001391 to GCST0002121), totaling 731 immune phenotypes, including absolute cell (AC) counts (*n* = 118), median fluorescence intensity (MFI) reflecting surface antigen levels (*n* = 389), morphological parameters (MP) (*n* = 32), and relative cell (RC) counts (*n* = 192) [23]. The original GWAS of immune traits used data from 3,757 European individuals with no overlapping cohorts. Approximately 22 million SNPs, genotyped using high-density arrays, were imputed based on the Sardinian sequence reference panel and tested for associations after adjusting covariates (i.e., gender, age, and age squared).

Access to the database was facilitated by YC and XJ on October 15, 2023, for a duration of one week. The access included one dataset on periodontitis and 731 datasets on immune cell phenotypes. Detailed data availability is in Supplementary Table S1.

### Selection of instrumental variables

All IVs used for further analysis were strictly screened based on three key assumptions [24]. Following previous articles, we set the significance level for each immune trait’s IVs at 1 × 10 − 5, excluding SNPs with an *F*-value < 10. Due to strong linkage disequilibrium among selected SNPs, which could bias results, a clustering process was conducted (r2 < 0.001, physical window = 10,000 kb), where LD r2 was calculated based on the 1000 Genomes Project as the reference panel [25]. For periodontitis, we adjusted the significance level to 5 × 10–6. Ultimately, we identified 98 IVs related to immune cell phenotypes and 19 IVs related to periodontitis. Detailed information on instrumental variables and calculation formulas can be found in Supplementary Tables S2 and S3.

### Statistical analysis

This study utilized five methods to explore the genetic link between immune cell phenotypes and chronic periodontitis: MR-Egger regression, weighted median, inverse-variance weighted (IVW), simple mode, and weighted mode. The IVW method, which combines SNP-exposure and SNP-outcome associations using a weighted linear regression model, was designated as the primary analytical approach due to its potential for the most precise estimates assuming all SNPs are valid instruments [26]. To assess heterogeneity, Cochrane's Q test was applied, and funnel plots were used to illustrate symmetry. If significant heterogeneity was detected, random effects IVW analysis was employed instead of fixed-effects IVW.

To evaluate pleiotropy, the MR-Egger intercept test and the MR pleiotropy residual sum and outliers (MR-PRESSO) global test were conducted [27]. The MR-Egger regression method accounts for directional pleiotropy by providing an intercept that indicates its presence, while MR-PRESSO identifies outliers and recalculates estimates post-exclusion to ensure the robustness of the findings. These tests help determine whether the observed associations are influenced by pleiotropy and identify any outlier SNPs that may affect the results.

Additionally, a leave-one-out analysis was performed to further assess the robustness of the findings [28]. This method involves sequentially removing each SNP and reanalyzing the remaining SNPs to examine their individual impact on the overall results. All statistical analyses were executed using R software (version 4.3.1) and the TwoSampleMR package [29]. The threshold for statistical significance was set at a *P*-value of < 0.05, ensuring a rigorous evaluation of the genetic associations between immune cell phenotypes and chronic periodontitis.

## Results

### The effect of immune cell phenotypes on chronic periodontitis

**Fig. 2:**
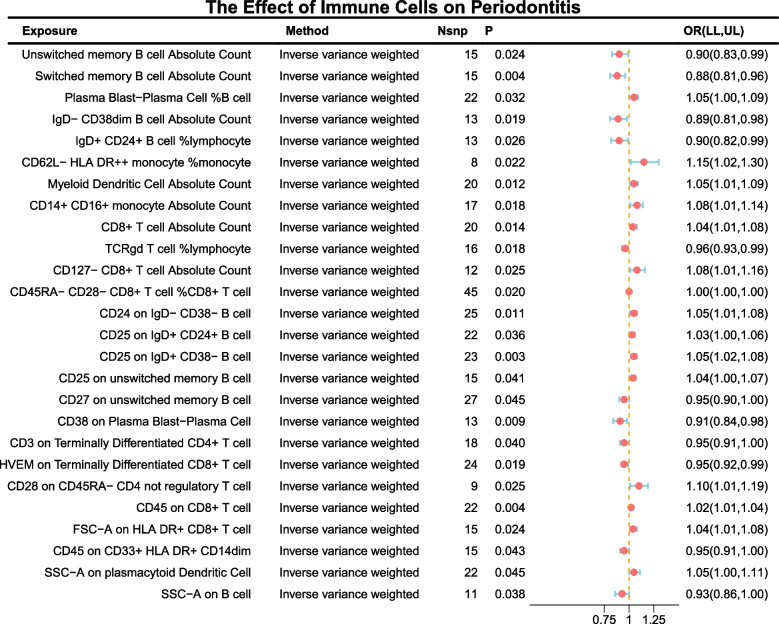
The effect of immune cell phenotypes on chronic periodontitis

The IVW method was primarily used to assess the impact of immune cell phenotypes on chronic periodontitis. At a significance level of 0.05, 26 potential immune cell phenotypes were identified (Fig. 2). Among them, in terms of absolute count, the increase in Unswitched Memory B cells (OR = 0.90 [0.83,0.99], *P* = 0.024), Switched Memory B cells (OR = 0.88 [0.81,0.96], *P* = 0.004), and IgD- CD38dim B cells (OR = 0.89 [0.81,0.98], *P* = 0.019) was associated with a reduced incidence of chronic periodontitis. Conversely, an increase in Myeloid Dendritic Cells (OR = 1.05 [1.01,1.09], *P* = 0.012), CD14 + CD16 + monocytes (OR = 1.08 [1.01,1.14], *P* = 0.018), CD8 + T cells (OR = 1.04 [1.01,1.08], *P* = 0.014), and CD127- CD8 + T cells (OR = 1.08 [1.01,1.16], *P* = 0.025) was associated with an increased incidence of chronic periodontitis.

In terms of relative cell count, an increase in IgD + CD24 + B cell %lymphocyte (OR = 0.90 [0.82,0.99], *P* = 0.026), TCRgd T cell %lymphocyte (OR = 0.96 [0.93,0.99], *P* = 0.018), and CD45RA- CD28- CD8 + T cell %CD8 + T cell (OR = 0.9989 [0.9980,0.9998], *P* = 0.020) was associated with a decreased incidence of chronic periodontitis. However, an increase in Plasma Blast-Plasma Cell %B cell (OR = 1.05 [1.00,1.09], *P* = 0.032) and CD62L- HLA DR +  + monocyte %monocyte (OR = 1.15 [1.02,1.30], *P* = 0.022) was associated with an increased incidence.

In terms of median fluorescence intensity, an increase in CD27 on Unswitched Memory B cells (0.95 [0.90,1.00], *P* = 0.045), CD38 on Plasma Blast-Plasma Cell (OR = 0.91 [0.84,0.98], *P* = 0.009), CD3 on Terminally Differentiated CD4 + T cells (OR = 0.95 [0.91,1.00], *P* = 0.040), HVEM on Terminally Differentiated CD8 + T cells (OR = 0.95 [0.92,0.99], *P* = 0.019), and CD45 on CD33 + HLA DR + CD14dim (0.95 [0.91,1.00], *P* = 0.043) was associated with a reduced incidence of chronic periodontitis. Conversely, an increase in CD24 on IgD- CD38- B cells (OR = 1.05 [1.01,1.08], *P* = 0.011), CD25 on IgD + CD24 + B cells (OR = 1.03 [1.00,1.06], *P* = 0.036), CD25 on IgD + CD38- B cells (OR = 1.05 [1.02,1.08], *P* = 0.003), CD25 on Unswitched Memory B cells (OR = 1.04 [1.00,1.07], *P* = 0.041), CD28 on CD45RA- CD4 non-regulatory T cells (OR = 1.10 [1.01,1.19], *P* = 0.025), and CD45 on CD8 + T cells (OR = 1.02 [1.01,1.04], *P* = 0.004) was associated with an increased incidence of chronic periodontitis.

In terms of morphological parameters, an increase in SSC-A on B cells (OR = 0.93 [0.86,1.00], *P* = 0.038) was associated with a reduced incidence of chronic periodontitis, while an increase in FSC-A on HLA DR + CD8 + T cells (OR = 1.04 [1.01,1.08], *P* = 0.024) and SSC-A on plasmacytoid Dendritic Cells (OR = 1.05 [1.00,1.11], *P* = 0.045) was associated with an increased incidence of chronic periodontitis.

The outcomes of the remaining methods, along with the sensitivity analysis, substantiated the robustness of the observed causal associations (see Supplementary Table S4, 6). Specifically, the MR-Egger intercept and the MR-PRESSO global test negated the likelihood of horizontal pleiotropy. Furthermore, scatter plots and funnel plots corroborated the stability of the results (refer to Supplementary Figs. 1). However, subsequent to multiple test adjustments employing the False Discovery Rate (FDR) method, no immune trait emerged as significant at the 0.05 threshold.

### The effect of chronic periodontitis on immune cell phenotypes

**Fig. 3:**
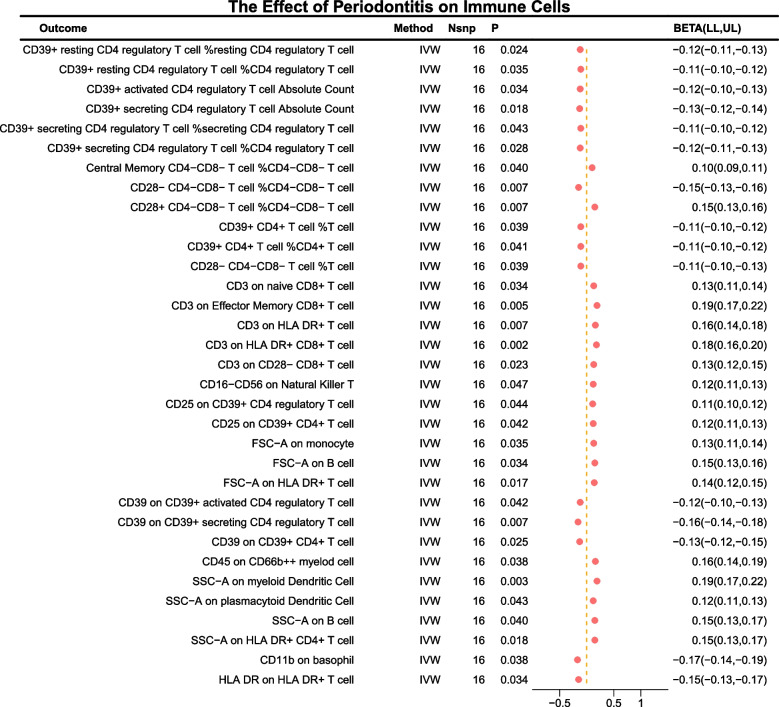
The effect of chronic periodontitis on immune cell phenotypes

In the reverse MR analysis, at a significance level of 0.05, 33 potential immune cell phenotypes were affected (Fig. 3). In terms of Absolute Count, having chronic periodontitis led to a decrease in CD39 + activated CD4 regulatory T cell Absolute Count (Beta = -0.12 [-0.10, -0.13], *P* = 0.03) and CD39 + secreting CD4 regulatory T cell Absolute Count (Beta = -0.13 [-0.12, -0.14], *P* = 0.02).

Regarding relative cell count, chronic periodontitis was associated with a decrease in CD39 + resting CD4 regulatory T cell %resting CD4 regulatory T cell (Beta = -0.12 [-0.11, -0.13], *P* = 0.02), CD39 + resting CD4 regulatory T cell %CD4 regulatory T cell (Beta = -0.11 [-0.10, -0.12], *P* = 0.04), CD39 + secreting CD4 regulatory T cell %secreting CD4 regulatory T cell (Beta = -0.11 [-0.10, -0.12], *P* = 0.04), CD39 + secreting CD4 regulatory T cell %CD4 regulatory T cell (Beta = -0.12 [-0.11, -0.13], *P* = 0.03), CD28- CD4-CD8- T cell %CD4-CD8- T cell (Beta = -0.15 [-0.13, -0.16], *P* = 0.01), CD39 + CD4 + T cell %T cell (Beta = -0.11 [-0.10, -0.12], *P* = 0.04), CD39 + CD4 + T cell %CD4 + T cell (Beta = -0.11 [-0.10, -0.12], *P* = 0.04), and CD28- CD4-CD8- T cell %T cell (Beta = -0.11 [-0.10, -0.13], *P* = 0.04). There was an increase in CD28 + CD4-CD8- T cell %CD4-CD8- T cell (Beta = 0.15 [0.13, 0.16], *P* = 0.01) and Central Memory CD4-CD8- T cell %CD4-CD8- T cell (Beta = 0.10 [0.09, 0.11], *P* = 0.04).

In terms of median fluorescence intensity, chronic periodontitis resulted in a decrease in CD39 on CD39 + activated CD4 regulatory T cell (Beta = -0.12 [-0.10, -0.13], *P* = 0.04), CD39 on CD39 + secreting CD4 regulatory T cell (Beta = -0.16 [-0.14, -0.18], *P* = 0.01), CD39 on CD39 + CD4 + T cell (Beta = -0.13 [-0.12, -0.15], *P* = 0.02), CD11b on basophil (Beta = -0.17 [-0.14, -0.19], *P* = 0.04), HLA DR on HLA DR + T cell (Beta = -0.15 [-0.13, -0.17], *P* = 0.03). There was an increase in CD3 on naive CD8 + T cell (Beta = 0.13 [0.11, 0.14], *P* = 0.03), CD3 on Effector Memory CD8 + T cell (Beta = 0.19 [0.17, 0.22], *P* = 0.01), CD3 on HLA DR + T cell (Beta = 0.16 [0.14, 0.18], *P* = 0.01), CD3 on HLA DR + CD8 + T cell (Beta = 0.18 [0.16, 0.20], *P* = 0.00), CD3 on CD28- CD8 + T cell (Beta = 0.13 [0.12, 0.15], *P* = 0.02), CD16-CD56 on Natural Killer T (Beta = 0.12 [0.11, 0.13], *P* = 0.05), CD25 on CD39 + CD4 regulatory T cell (Beta = 0.11 [0.10, 0.12], *P* = 0.04), CD25 on CD39 + CD4 + T cell (Beta = 0.12 [0.11, 0.13], *P* = 0.04), and CD45 on CD66b +  + myeloid cell (Beta = 0.16 [0.14, 0.19], *P* = 0.04).

Lastly, in the morphological parameters, having periodontitis led to an increase in FSC-A on monocyte (Beta = 0.13 [0.11, 0.14], *P* = 0.03), FSC-A on B cell (Beta = 0.15 [0.13, 0.16], *P* = 0.03), FSC-A on HLA DR + T cell (Beta = 0.14 [0.12, 0.15], *P* = 0.02), SSC-A on myeloid Dendritic Cell (Beta = 0.19 [0.17, 0.22], *P* = 0.00), SSC-A on plasmacytoid Dendritic Cell (Beta = 0.12 [0.11, 0.13], *P* = 0.04), SSC-A on B cell (Beta = 0.15 [0.13, 0.17], *P* = 0.04), and SSC-A on HLA DR + CD4 + T cell (Beta = 0.15 [0.13, 0.17], *P* = 0.02).

Additionally, the outcomes of the remaining methods, coupled with the sensitivity analysis, reinforced the robustness of the observed causal associations (see Supplementary Tables S5, S7). Moreover, scatter plots and funnel plots further confirmed the stability of these results (refer to Supplementary Fig. 2). Similarly, following multiple test adjustments using the FDR method, no immune trait was found to be significant at the 0.05 threshold.

## Discussion

Utilizing publicly available genetic data, our research explored the causal relationships between various immune cell phenotypes and chronic periodontitis. To our knowledge, this is the first Mendelian randomization analysis to investigate the causal connections between multiple immune phenotypes and chronic periodontitis. In our analysis of the impact of immune cell phenotypes on chronic periodontitis, 26 potential immune cell phenotypes demonstrated significant associations with chronic periodontitis (*p* < 0.05). Meanwhile, in the reserve analysis of the impact of chronic periodontitis on immune cell phenotypes, 33 potential immune cell phenotypes were identified as having significant associations (*p* < 0.05).

Previous observational studies have supported the findings of our current research. These studies have shown that in tissues with healthy gingiva and gingivitis, the dominant B cell type is the CD19 + CD27 + CD38- memory B cell. In contrast, in periodontitis tissues, there is a marked decrease in these memory B cells [30]. Comparing immune profiles of healthy individuals and those with chronic periodontitis reveals significant differences. Patients with chronic periodontitis exhibit a notable increase in CD14 + CD16 + monocytes in their peripheral blood [31], indicating a systemic shift in immune cell distribution. This increase is also evident in the gingival tissues of these patients, suggesting a local tissue reaction [32]. Additionally, these monocytes in periodontitis patients show higher levels of HLA-DR expression [33]. Supporting these observations, Fluorescence-activated cell sorting (FACS) analysis in another study demonstrated a considerable increase in blood myeloid dendritic cells in individuals with chronic periodontitis compared to healthy subjects [34]. Moreover, a recent comprehensive meta-analysis illuminated a wide array of immune changes associated with periodontal disease. It revealed a significant average increase in various circulating immune cells, including subsets of T cells (CD4 + , CD4 + CD45RO + , and IFNγ-expressing CD4 + and CD8 +), B cell types (CD19 + CD27 + and CD5 +), as well as CD14 + CD16 + monocytes and CD16 + neutrophils [35]. Despite these advancements in our understanding, there remains a significant knowledge gap regarding the more detailed and specific phenotypes of immune cells in periodontal disease. This underscores the necessity for more targeted research to further understand the nuances of immune cell dynamics and their involvement in the development and progression of periodontal disease.

In the context of periodontitis, B cells play a crucial role in both the immune response and the disease's progression [36]. They evolve into plasma cells, which are essential for producing antibodies specifically targeting periodontal pathogens [37]. This action is vital for neutralizing these pathogens and averting further tissue damage. We have noted a reduction in memory B cells, likely due to the inflammation-triggered extensive differentiation of plasma cells, leading to a decreased proportion of memory B cells [30]. Moreover, activated B cells can serve as antigen-presenting cells for CD4 and CD8 T cells, distinct from dendritic cells [38, 39]. B cells can selectively present homologous antigens they have collected through surface immunoglobulins, enabling even low concentrations of antigens to be presented [40]. These cells also directly contribute to bone loss in periodontitis, possibly by enhancing RANKL expression, which in turn promotes osteoclastogenesis [41].

T cells are crucial in the progression of periodontitis [42]. The various subsets of CD4 + T cells influence the inflammatory response in this disease by producing different cytokines, such as Th1, Th2, and Th17 cells [36]. Th1 cells produce IFN-γ, IL-2, and TNF-α, which activate macrophages and promote the production of IgG2a antibodies, thus mediating a macrophage-dominant host defense response. Th2 cells secrete cytokines like IL-4, IL-5, and IL-9, aiding in B cell activation, proliferation, and antibody class switching, and are involved in humoral immune responses [43]. Some cytokines from Th2 cells can inhibit macrophage function, leading to the perception of Th2 cells as mediators of a host defense independent of macrophages. Th17 cells primarily produce IL-17 and are involved in various inflammatory responses. IL-17 can promote the formation of bone-resorbing osteoclasts [44].

Regulatory T cells (Tregs) have a balancing role, secreting transforming growth factor-beta (TGF-β) and IL-10 to inhibit the excessive activation of Th1, Th2, and Th17 cells, thereby providing an immune regulatory function [45]. Additionally, activated T cells can promote B cell proliferation, antibody production, and the extensive release of IL-1, indirectly causing tissue destruction. CD8 + T cell clones, which can originate from periodontitis-affected tissues, may secrete cytokines like IL-4 and IL-5 to inhibit the production of IFN-γ and promote humoral immune responses [46]. CD8 + T cells, like CD4 + T cells, express cytokines such as IFN-γ and IL-5 in pathological periodontal tissues, reflecting the dominant presence of Th1 cells in the affected tissues [47].

In addition to B and T cells, other immune cells like dendritic cells, monocytes, and natural killer (NK) cells also have significant roles in periodontitis. An increase in dendritic cells may signify heightened immune activation, which could lead to chronic inflammation [48]. The rise in CD14 + CD16 + monocytes can further aggravate tissue destruction and inflammation within periodontal tissues [33]. Additionally, changes in cell phenotypes, such as elevated expression of CD27 and CD38 on B cells, indicate shifts in the activation and differentiation states of immune cells. These alterations could significantly influence the efficiency and pathological nature of the immune response [49].

The morphological features of cells, exemplified by variations in FSC-A (forward scatter area) and SSC-A (side scatter area), are critical for discerning cell activation and functional states. Typically, an increase in FSC-A suggests a growth in cell size, often linked to an activated state [50]. A rise in SSC-A indicates a greater internal complexity, which may be related to improved antigen processing and presentation abilities. These morphological changes provide essential insights into the activation and functional dynamics of immune cells in the context of periodontitis.

In addition to enhancing our understanding of the relationship between periodontitis and immune responses, the results of this study also have significant clinical implications. Understanding the bidirectional relationship between immune cell phenotypes and periodontitis may facilitate the development of predictive biomarkers. These biomarkers could be used to identify individuals at higher risk for periodontitis, allowing for earlier intervention and prevention strategies. For example, research indicates that using a combination of periodontal pathogens like Porphyromonas gingivalis and salivary biomarkers such as interleukin-1β (IL-1β) and prostaglandin E2 (PGE2) can aid in predicting chronic periodontitis in older individuals [51]. Moreover, managing systemic immune conditions may have a beneficial impact on periodontal health, and vice versa. For instance, conditions like diabetes, cardiovascular disease, and rheumatoid arthritis have been closely linked with periodontal disease, suggesting that managing these systemic conditions can help mitigate periodontal issues. Conversely, improving periodontal health can positively influence systemic conditions by reducing the overall inflammatory burden and microbial load in the body [52].

However, it is worth noting that despite the robust analysis, no significant findings remained after adjusting for the False Discovery Rate (FDR). The FDR adjustment is a critical step in studies involving multiple comparisons, as it controls the expected proportion of false positives, thus providing a more conservative and reliable interpretation of the data. While this adjustment increases the stringency of our results, leading to the loss of statistical significance, it does not diminish the potential biological relevance of the identified associations. Despite the loss of statistical significance after FDR correction, these results remain valuable. They suggest areas of interest that could be explored in future studies with larger sample sizes or more refined phenotypic definitions. Moreover, the observed associations provide a basis for generating new hypotheses and guiding future research directions.

This study also has several limitations. The genetic data and aggregate statistics used primarily originate from European populations, which may not be applicable to all groups. Further inclusion of cohorts from other populations is needed. Additionally, periodontitis is a complex disease with considerable phenotypic heterogeneity. Our research focuses on chronic periodontitis, potentially overlooking other forms or stages of the disease. Moreover, the classification of periodontitis based on ICD-10 might not comprehensively reflect the clinical manifestations of the disease.

## Conclusion

In conclusion, this Mendelian randomization study reveals a bidirectional causal relationship between specific immune cell phenotypes and chronic periodontitis, highlighting the complexity of periodontal disease pathogenesis and the significant role of the immune system. Future research should aim for broader generalizability and deeper exploration of these intricate relationships.

### Supplementary Information

Supplementary Material 1.

Supplementary Material 2.

Supplementary Material 3.

## Data Availability

Datasets supporting the conclusions of this article are available in the Finngen and GWAS Catalog repository, [K11_PERIODON_CHRON, https://risteys.finregistry.fi/endpoints/K11_PERIODON_CHRON; GCST0001391 to GCST0002121, https://www.ebi.ac.uk/gwas/publications/32929287].
