# Supplementary material for: Causal role of immune cells in chronic periodontitis: a bidirectional Mendelian randomization study
Source: BMC Oral Health. 2024 Jul 16;24:806. doi: 10.1186/s12903-024-04592-0 (PMC11253491; doi:10.1186/s12903-024-04592-0)
Supplement: Supplementary file 2 — Supplementary Material 2. [file 12903_2024_4592_MOESM2_ESM.pdf]

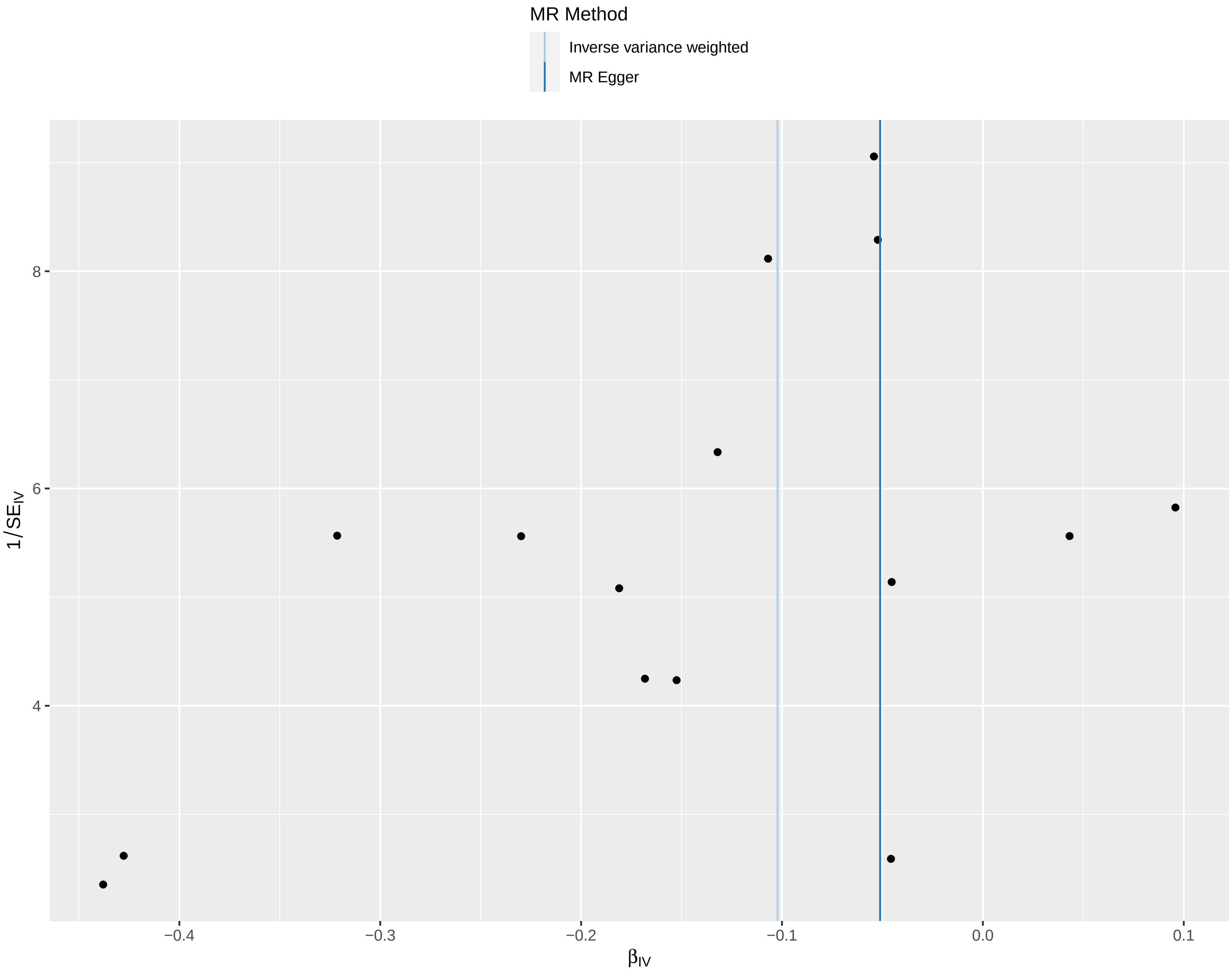
MR Method
Inverse variance weighted
MR Egger
8
6
4
1/SE_IV
−0.4
−0.3
−0.2
−0.1
0.0
0.1
β_IV

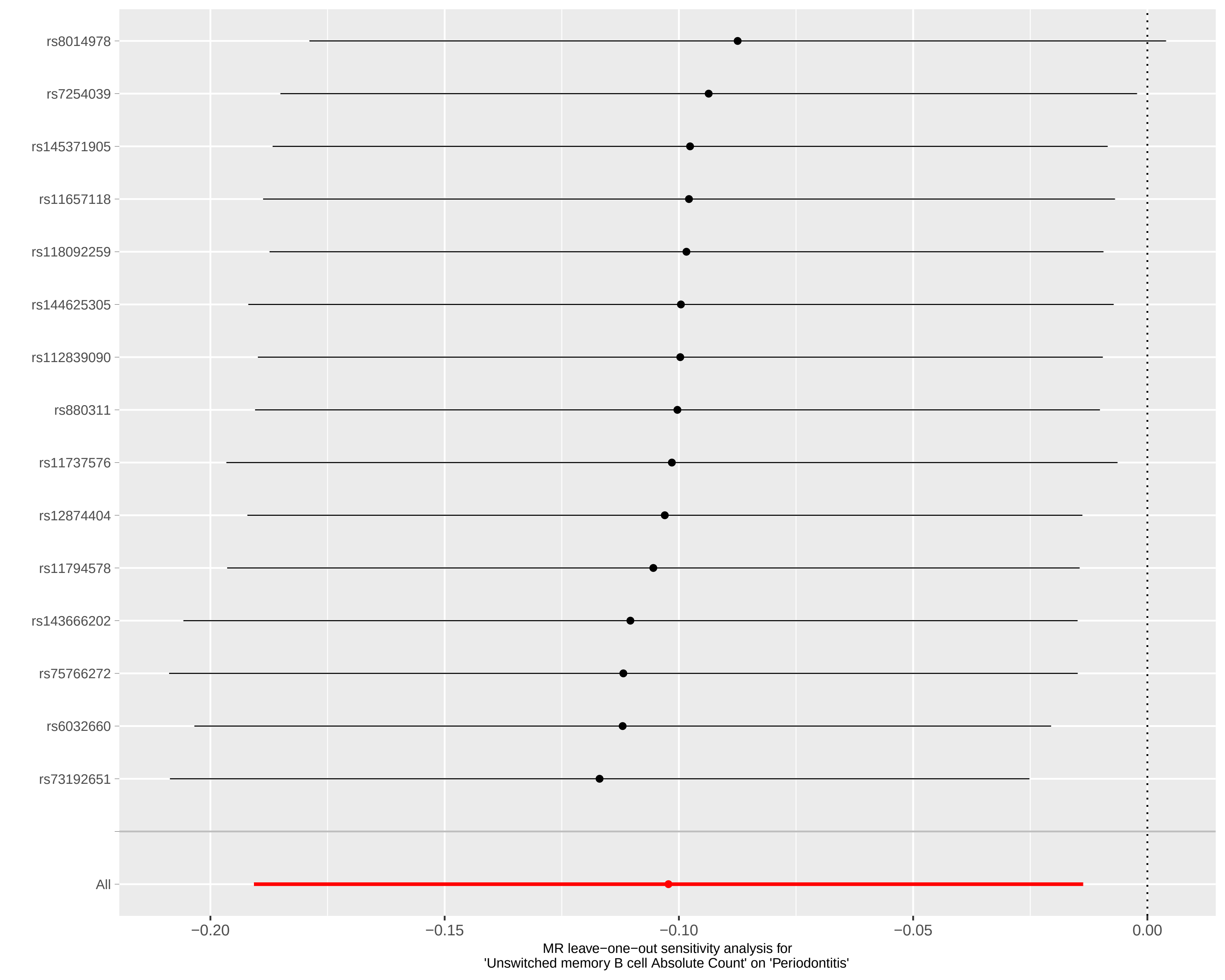
rs8014978
rs7254039
rs145371905
rs11657118
rs118092259
rs144625305
rs112839090
rs880311
rs11737576
rs12874404
rs11794578
rs143666202
rs75766272
rs6032660
rs73192651
All
−0.20
−0.15
−0.10
−0.05
0.00
MR leave−one−out sensitivity analysis for
'Unswitched memory B cell Absolute Count' on 'Periodontitis'

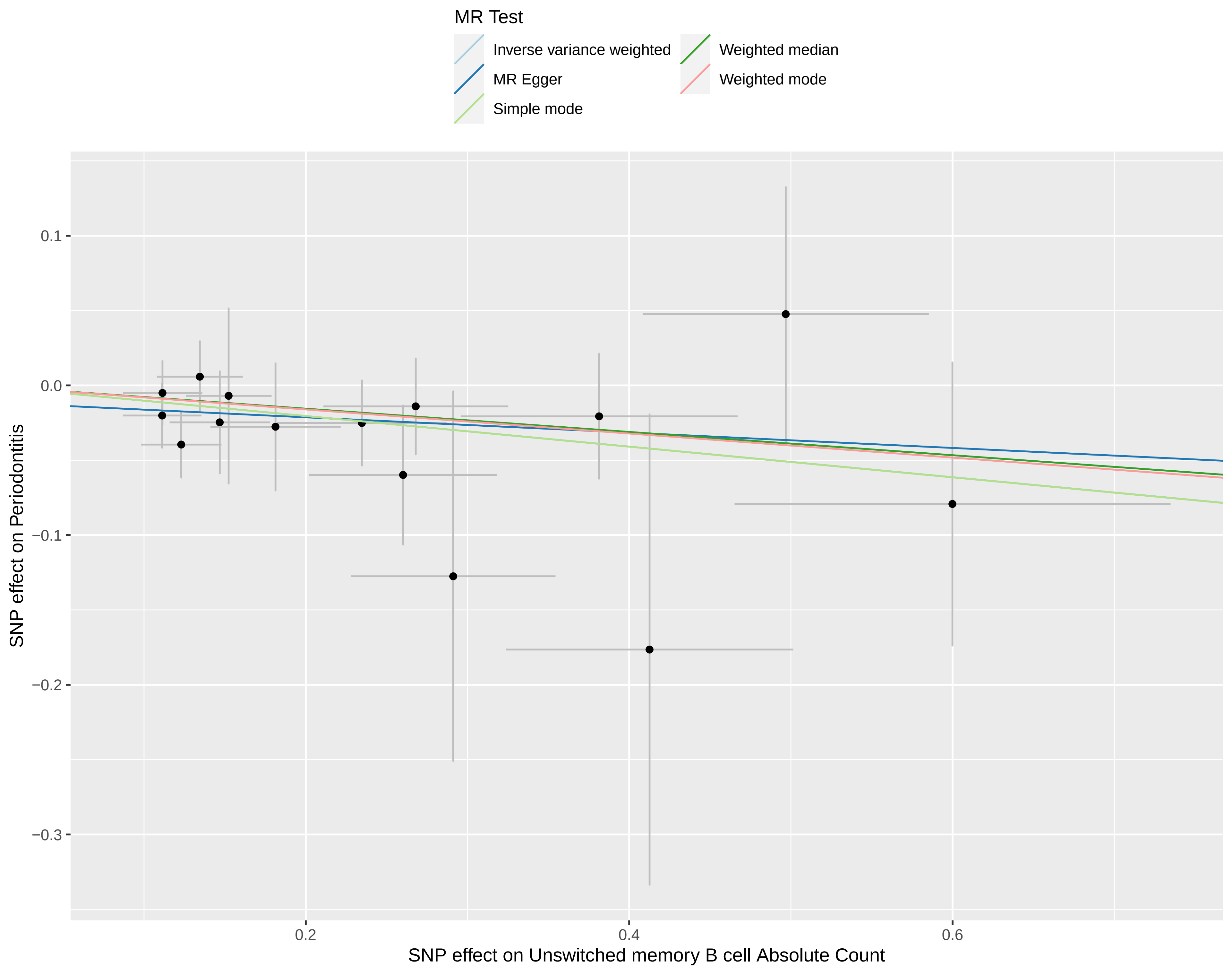
MR Test
Inverse variance weighted
MR Egger
Simple mode
Weighted median
Weighted mode
0.1
0.0
−0.1
−0.2
−0.3
0.2
0.4
0.6
SNP effect on Periodontitis
SNP effect on Unswitched memory B cell Absolute Count

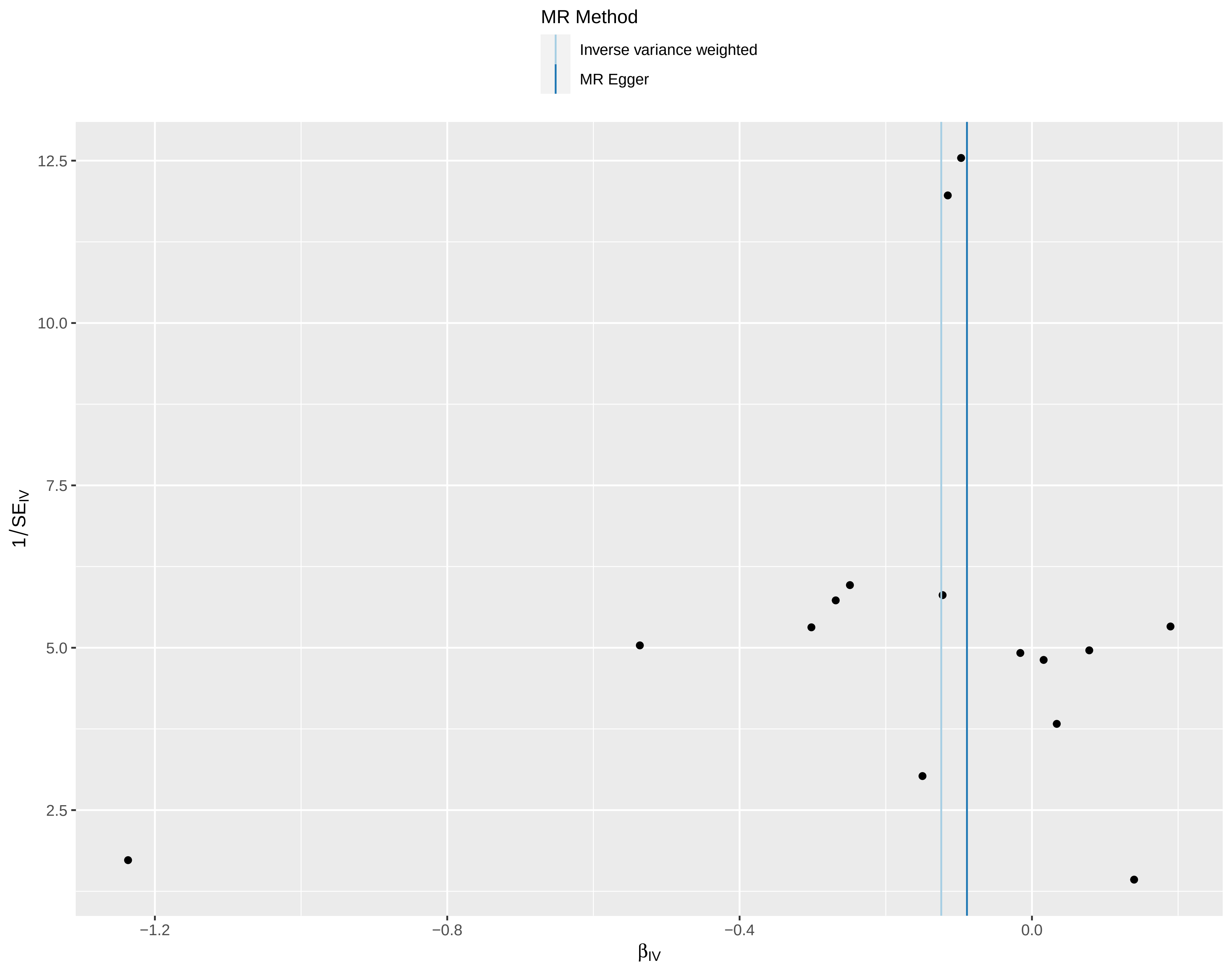
MR Method
Inverse variance weighted
MR Egger
12.5
10.0
7.5
5.0
2.5
$1/SE_{IV}$
−1.2
−0.8
−0.4
0.0
β_{IV}

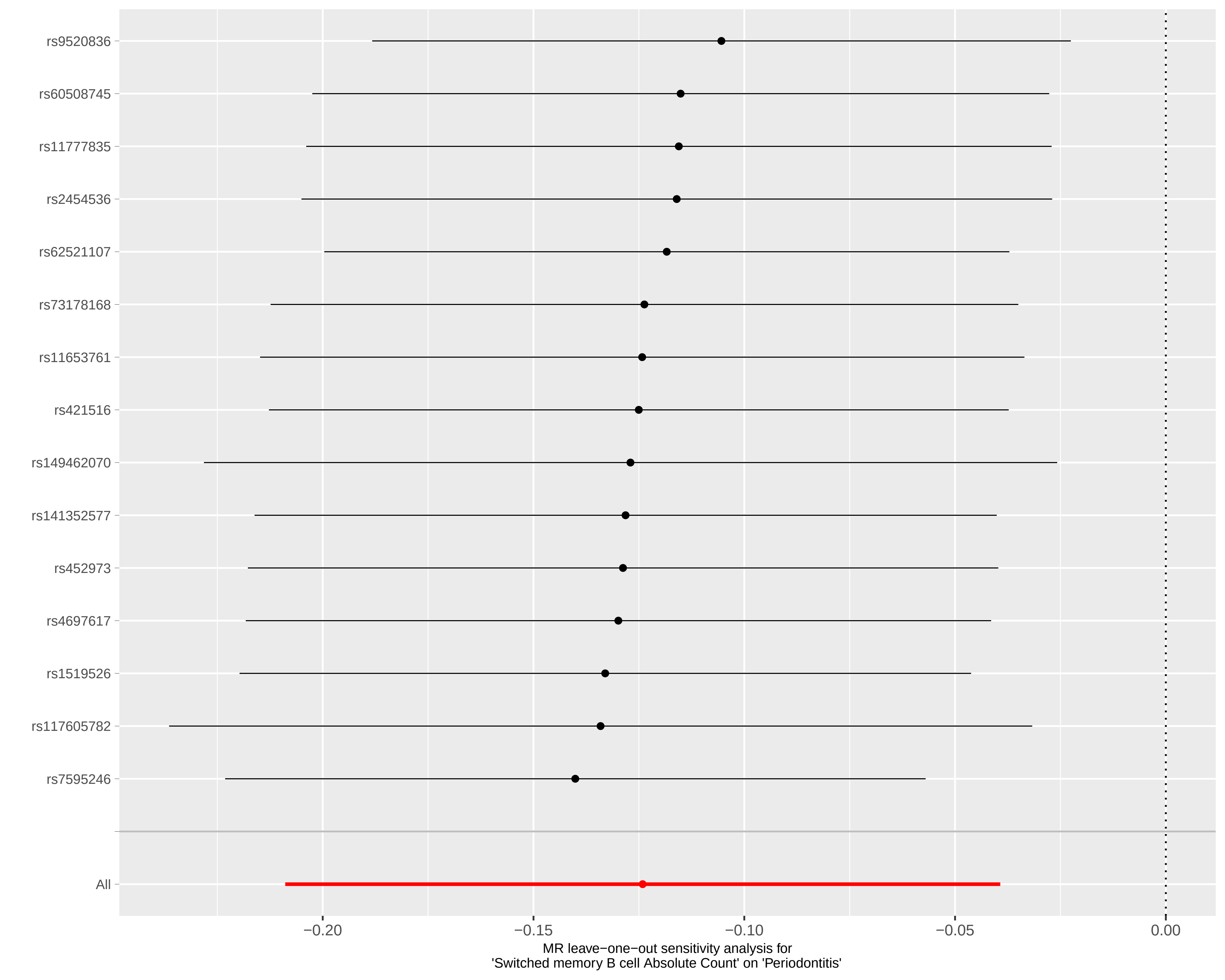
rs9520836
rs60508745
rs11777835
rs2454536
rs62521107
rs73178168
rs11653761
rs421516
rs149462070
rs141352577
rs452973
rs4697617
rs1519526
rs117605782
rs7595246
All
−0.20
−0.15
−0.10
−0.05
0.00
MR leave−one−out sensitivity analysis for
'Switched memory B cell Absolute Count' on 'Periodontitis'

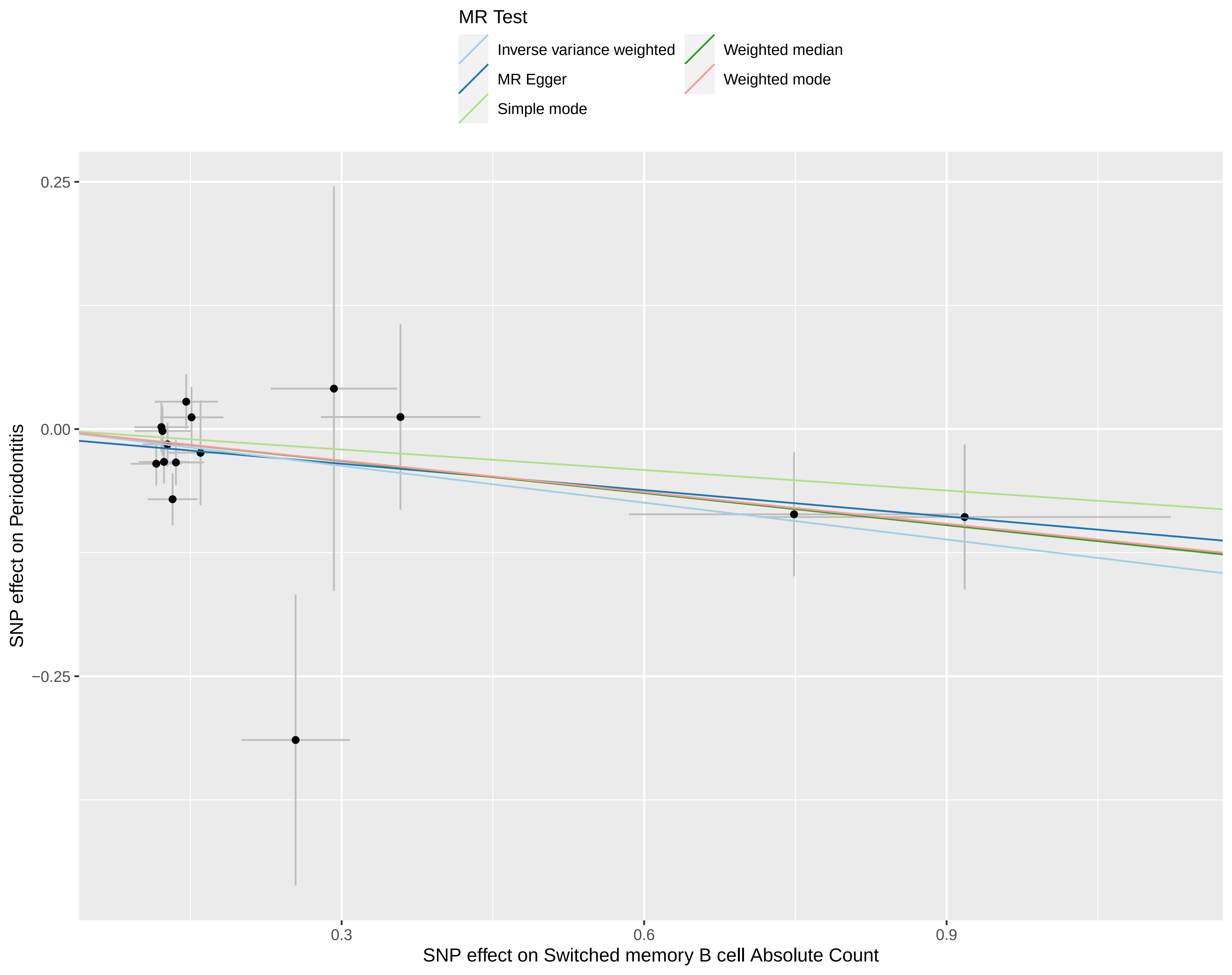
MR Test
Inverse variance weighted
MR Egger
Simple mode
Weighted median
Weighted mode
0.25
0.00
−0.25
SNP effect on Periodontitis
0.3
0.6
0.9
SNP effect on Switched memory B cell Absolute Count

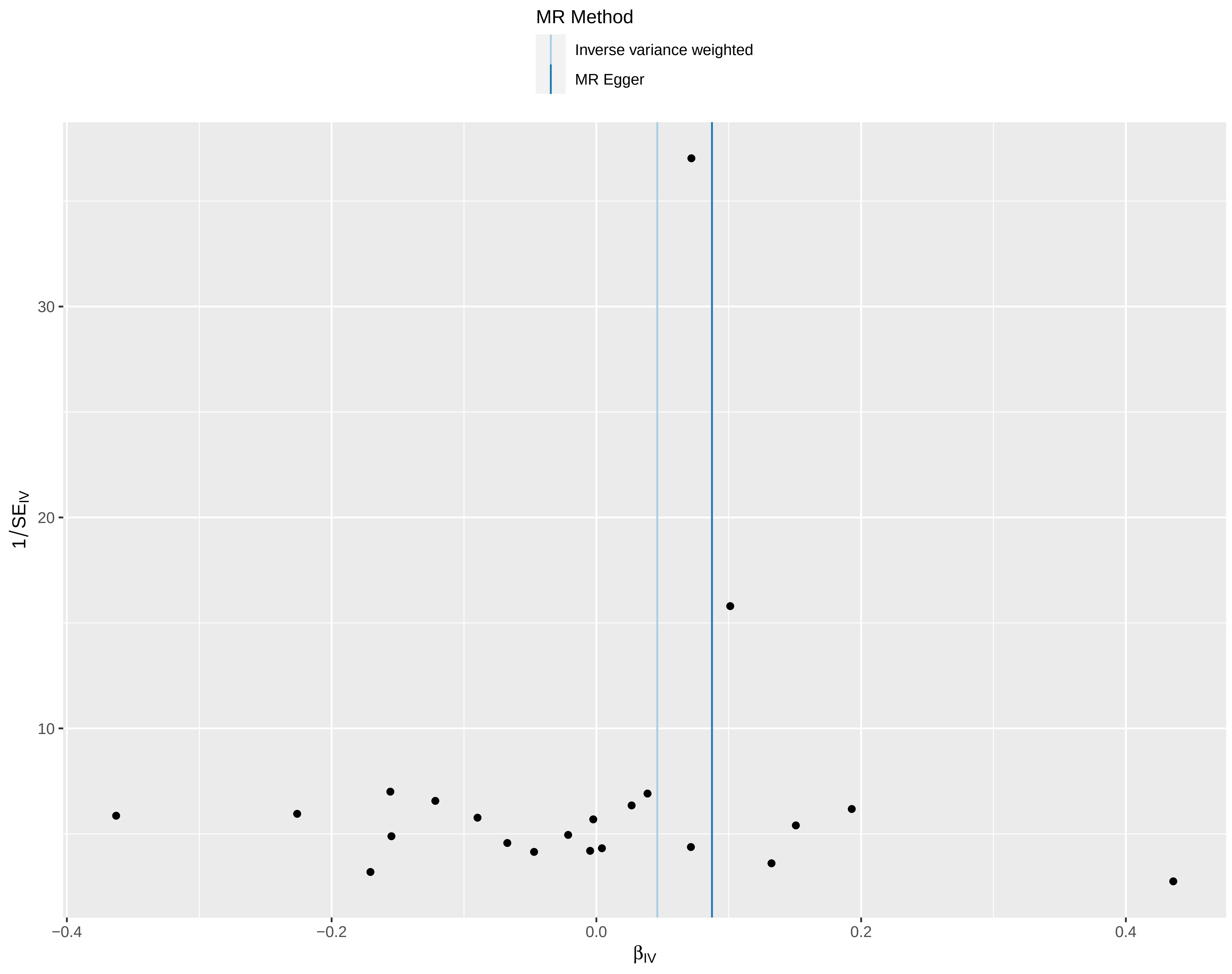
MR Method
Inverse variance weighted
MR Egger
30
20
10
1/SE_IV
−0.4
−0.2
0.0
0.2
0.4
β_IV

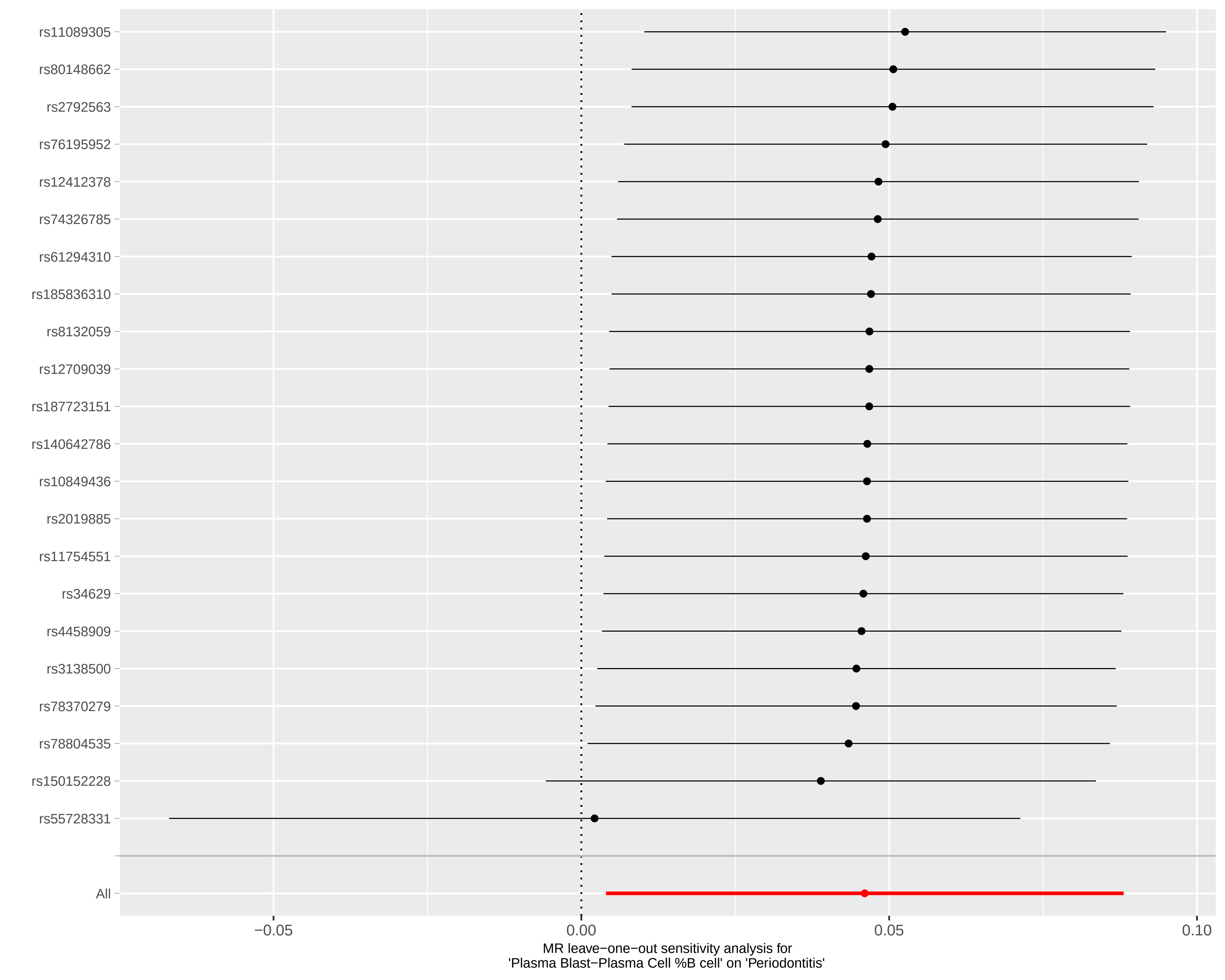
rs11089305
rs80148662
rs2792563
rs76195952
rs12412378
rs74326785
rs61294310
rs185836310
rs8132059
rs12709039
rs187723151
rs140642786
rs10849436
rs2019885
rs11754551
rs34629
rs4458909
rs3138500
rs78370279
rs78804535
rs150152228
rs55728331
All
−0.05
0.00
0.05
0.10
MR leave−one−out sensitivity analysis for
'Plasma Blast−Plasma Cell %B cell' on 'Periodontitis'

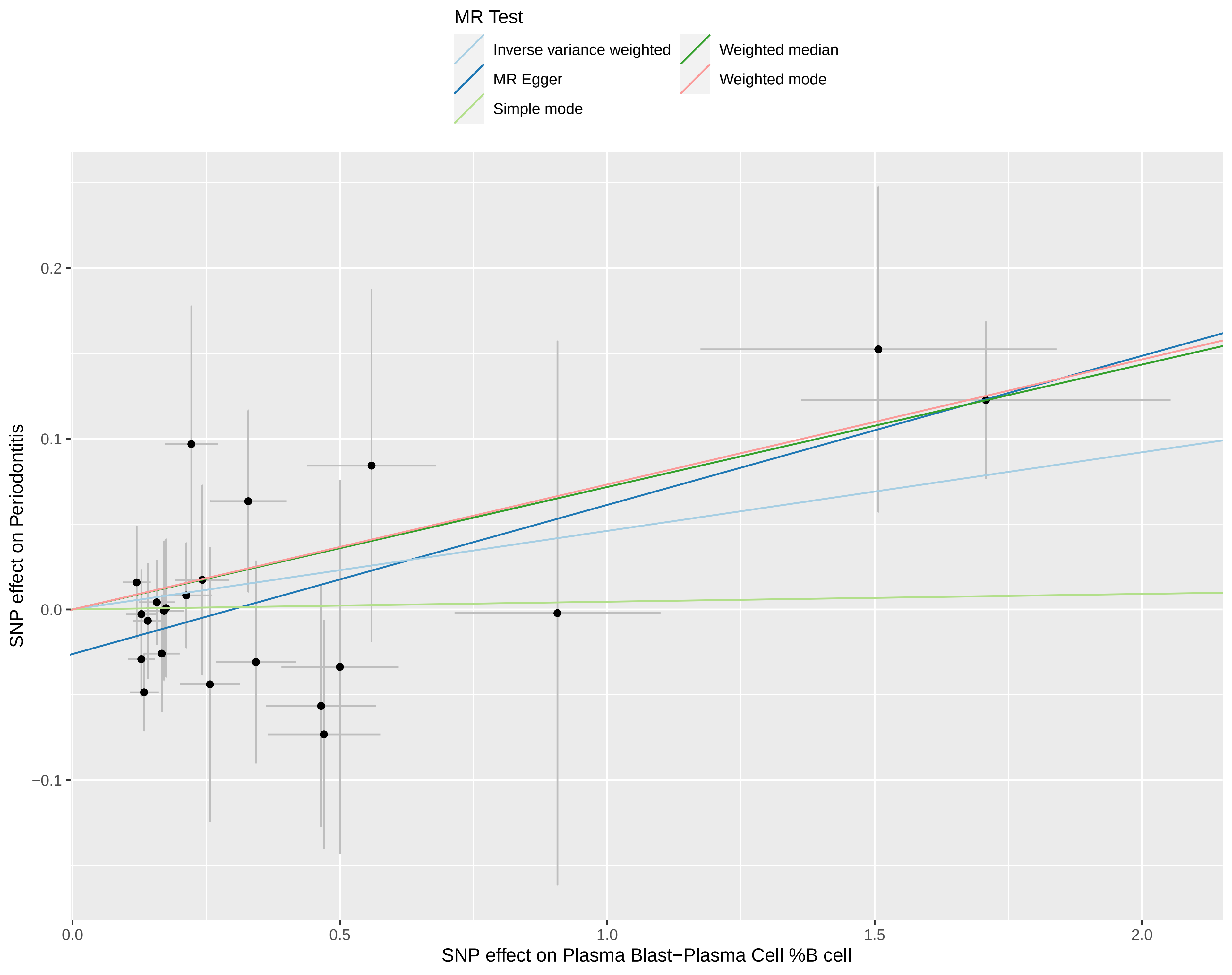
MR Test
Inverse variance weighted
MR Egger
Simple mode
Weighted median
Weighted mode
SNP effect on Periodontitis
0.2
0.1
0.0
−0.1
0.0
0.5
1.0
1.5
2.0
SNP effect on Plasma Blast−Plasma Cell %B cell

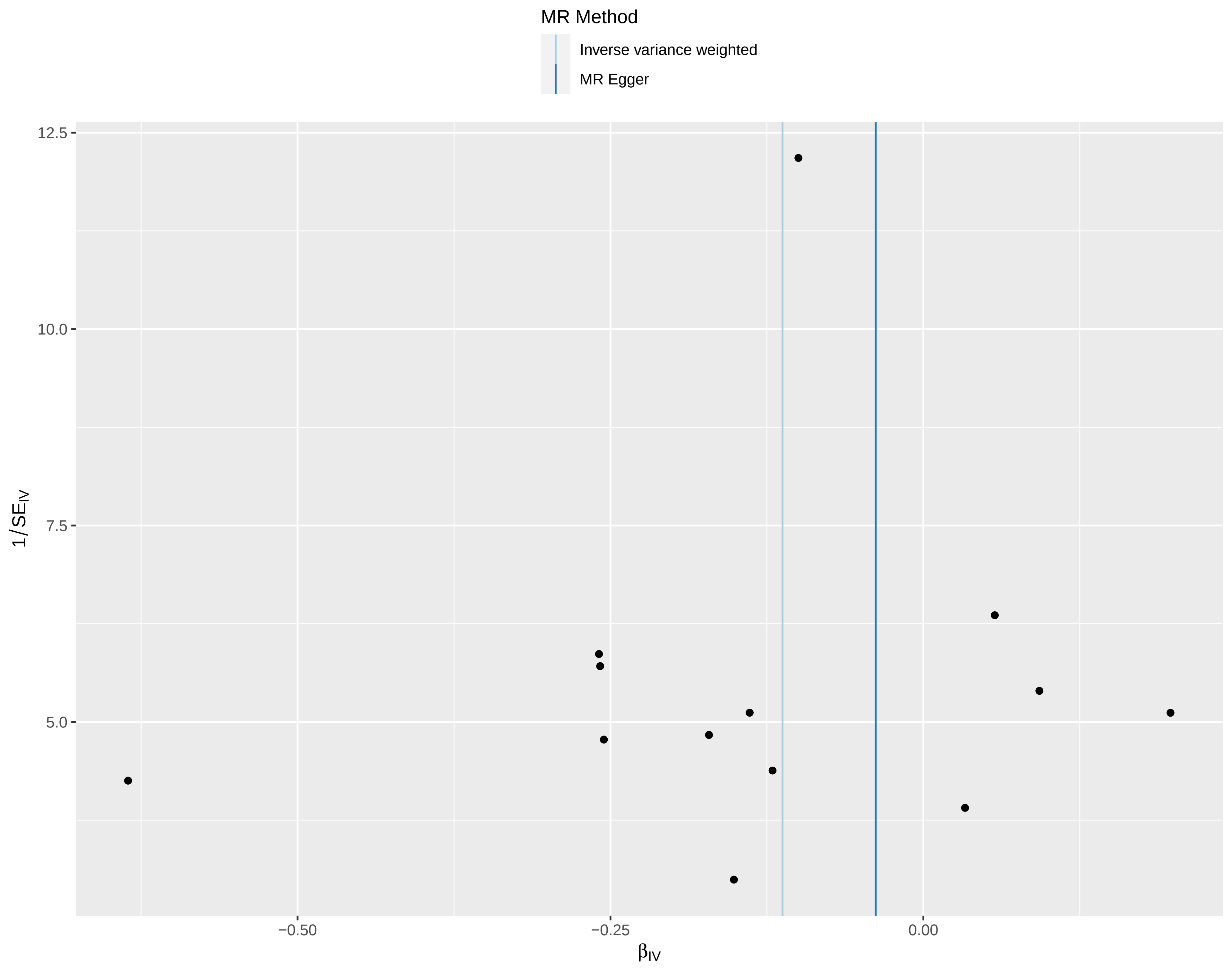

MR Method
Inverse variance weighted
MR Egger
12.5
10.0
7.5
5.0
1/SE_IV
−0.50
−0.25
0.00
β_IV

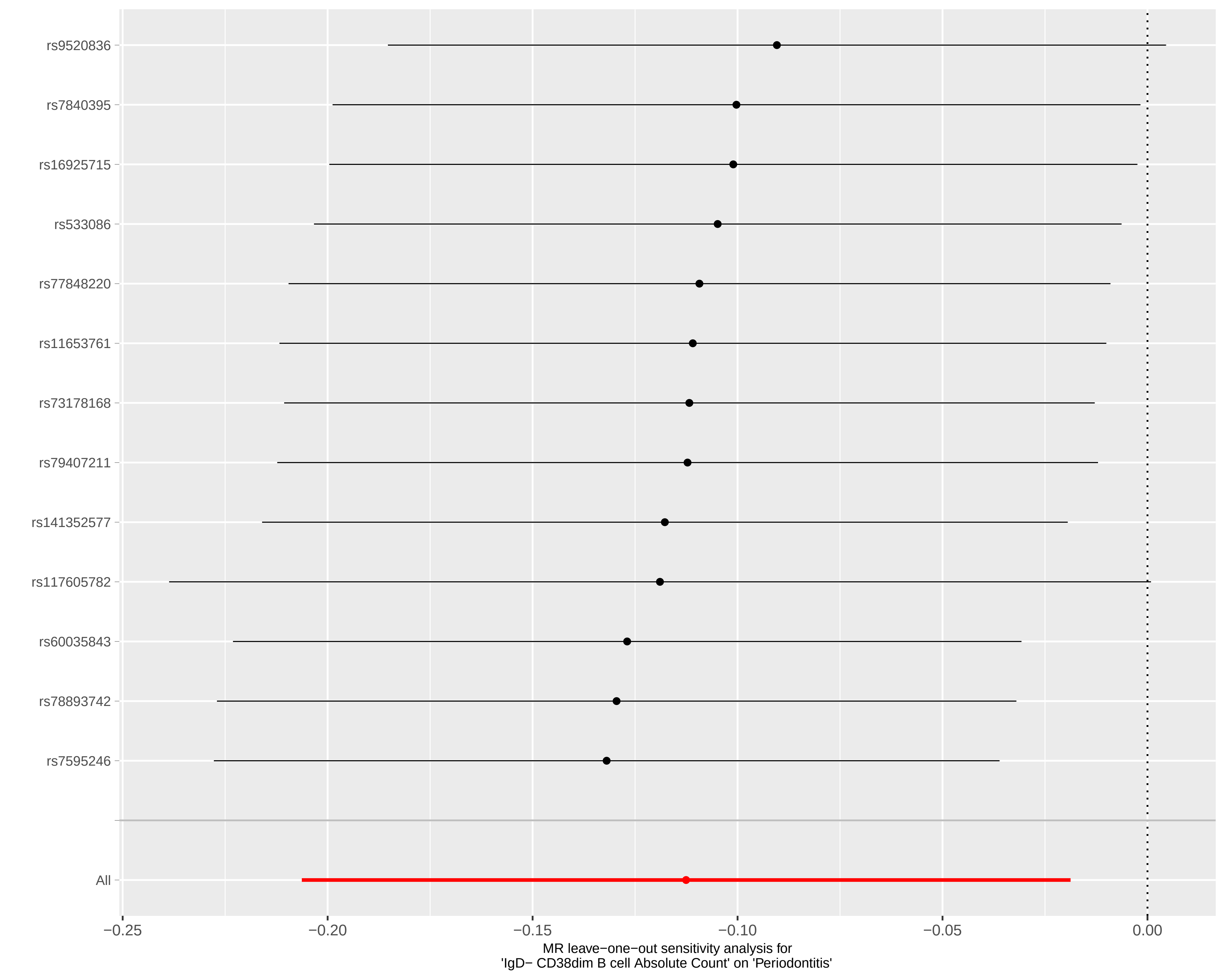

rs9520836
rs7840395
rs16925715
rs533086
rs77848220
rs11653761
rs73178168
rs79407211
rs141352577
rs117605782
rs60035843
rs78893742
rs7595246
All
−0.25
−0.20
−0.15
−0.10
−0.05
0.00
MR leave−one−out sensitivity analysis for
'IgD− CD38dim B cell Absolute Count' on 'Periodontitis'

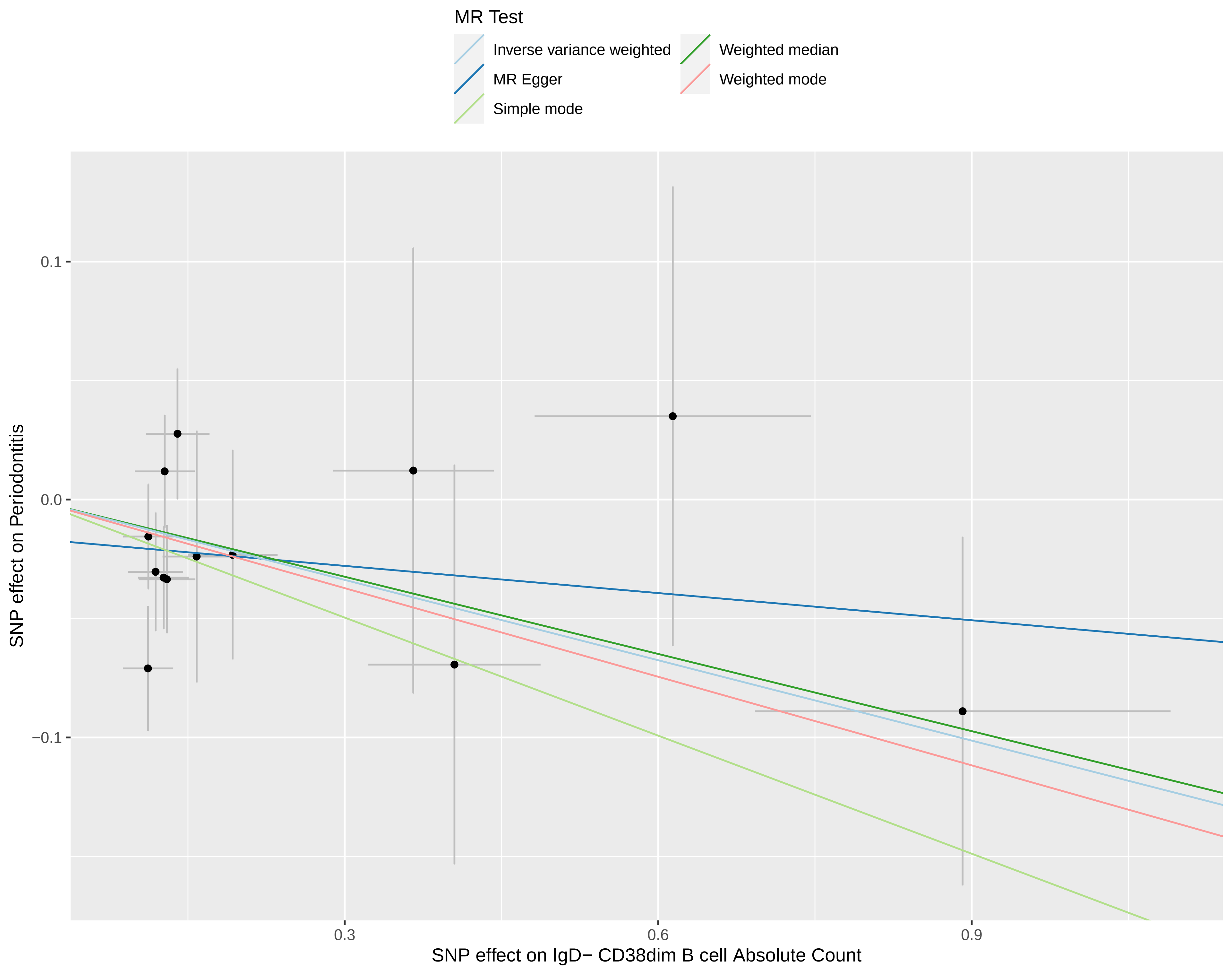
MR Test
Inverse variance weighted
MR Egger
Simple mode
Weighted median
Weighted mode
0.1
0.0
−0.1
0.3
0.6
0.9
SNP effect on Periodontitis
SNP effect on IgD− CD38dim B cell Absolute Count

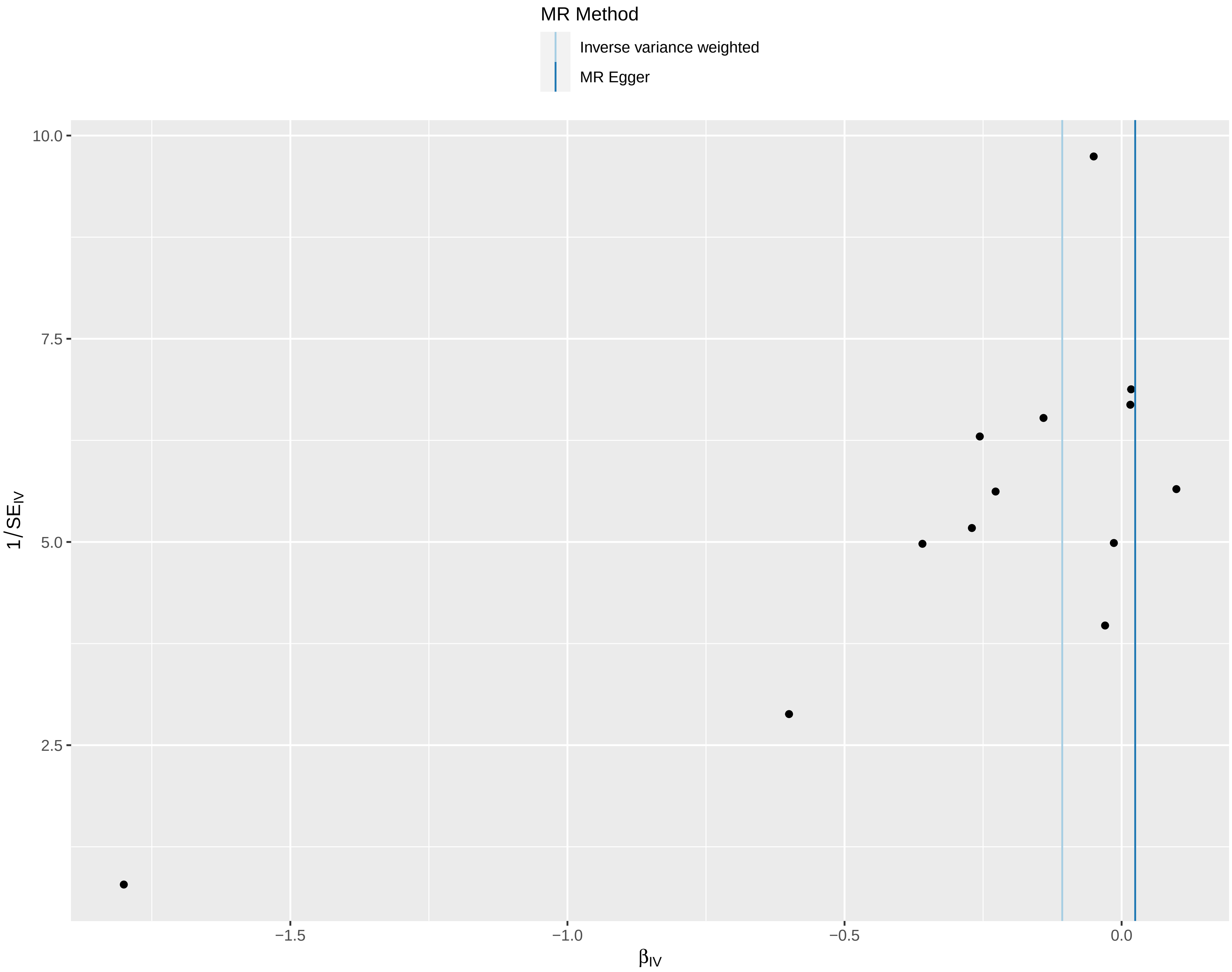
MR Method
Inverse variance weighted
MR Egger
10.0
7.5
5.0
2.5
1/SE_IV
−1.5
−1.0
−0.5
0.0
β_IV

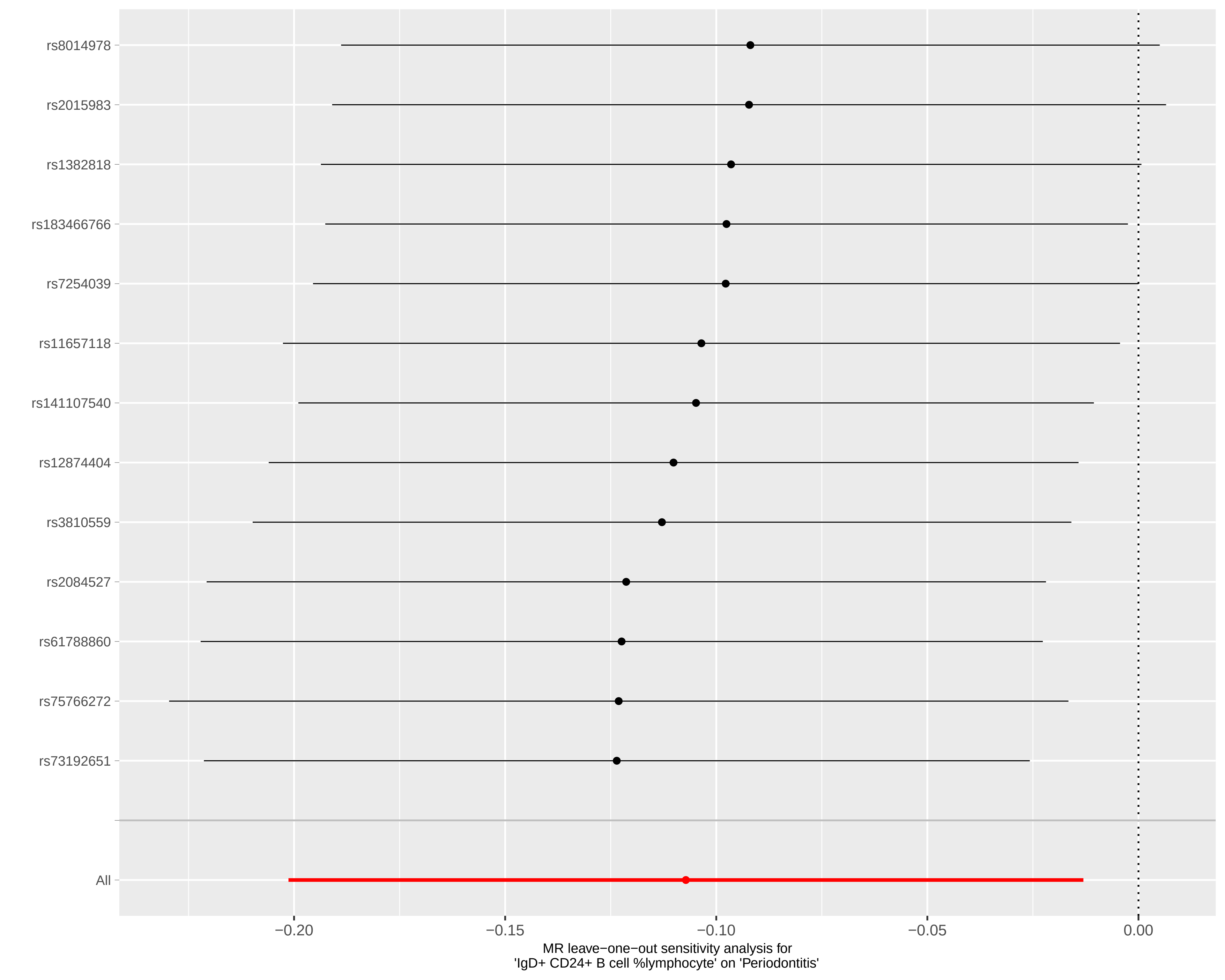
rs8014978
rs2015983
rs1382818
rs183466766
rs7254039
rs11657118
rs141107540
rs12874404
rs3810559
rs2084527
rs61788860
rs75766272
rs73192651
All
−0.20
−0.15
−0.10
−0.05
0.00
MR leave−one−out sensitivity analysis for
'IgD+ CD24+ B cell %lymphocyte' on 'Periodontitis'

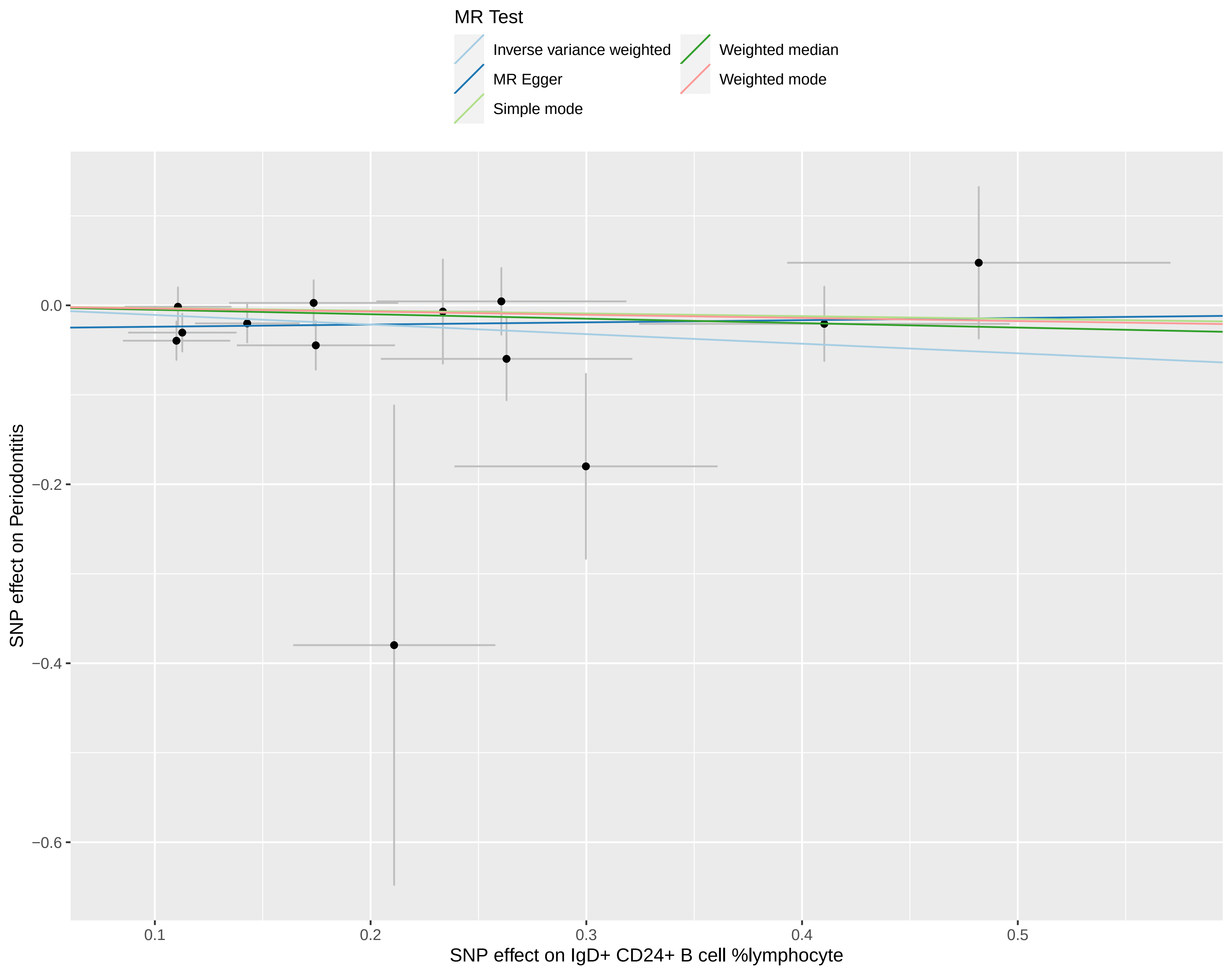
MR Test
Inverse variance weighted
MR Egger
Simple mode
Weighted median
Weighted mode
0.0
−0.2
−0.4
−0.6
SNP effect on Periodontitis
0.1
0.2
0.3
0.4
0.5
SNP effect on IgD+ CD24+ B cell %lymphocyte

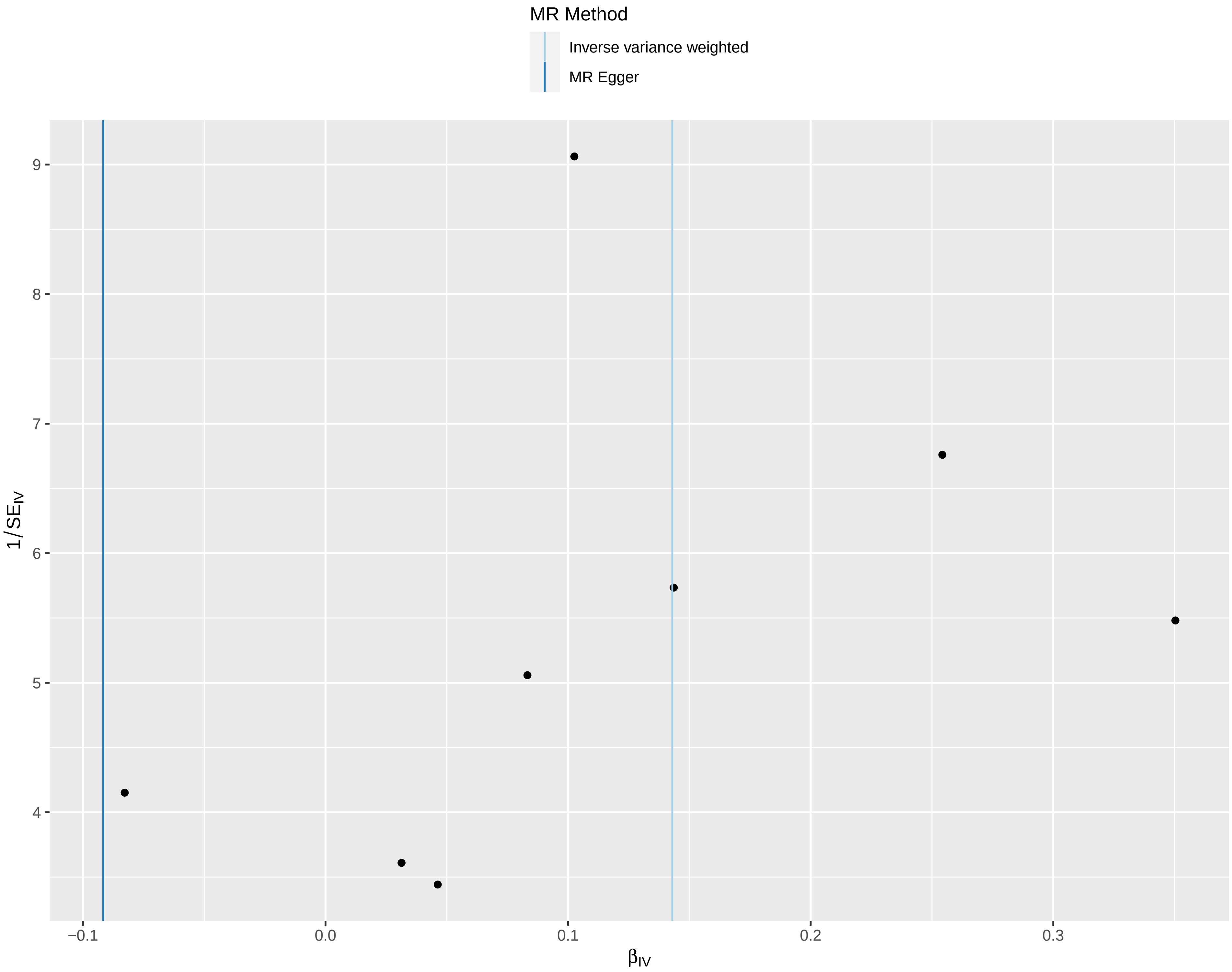

MR Method
Inverse variance weighted
MR Egger
9
8
7
6
5
4
1/SE$_{IV}$
−0.1
0.0
0.1
0.2
0.3
β$_{IV}$

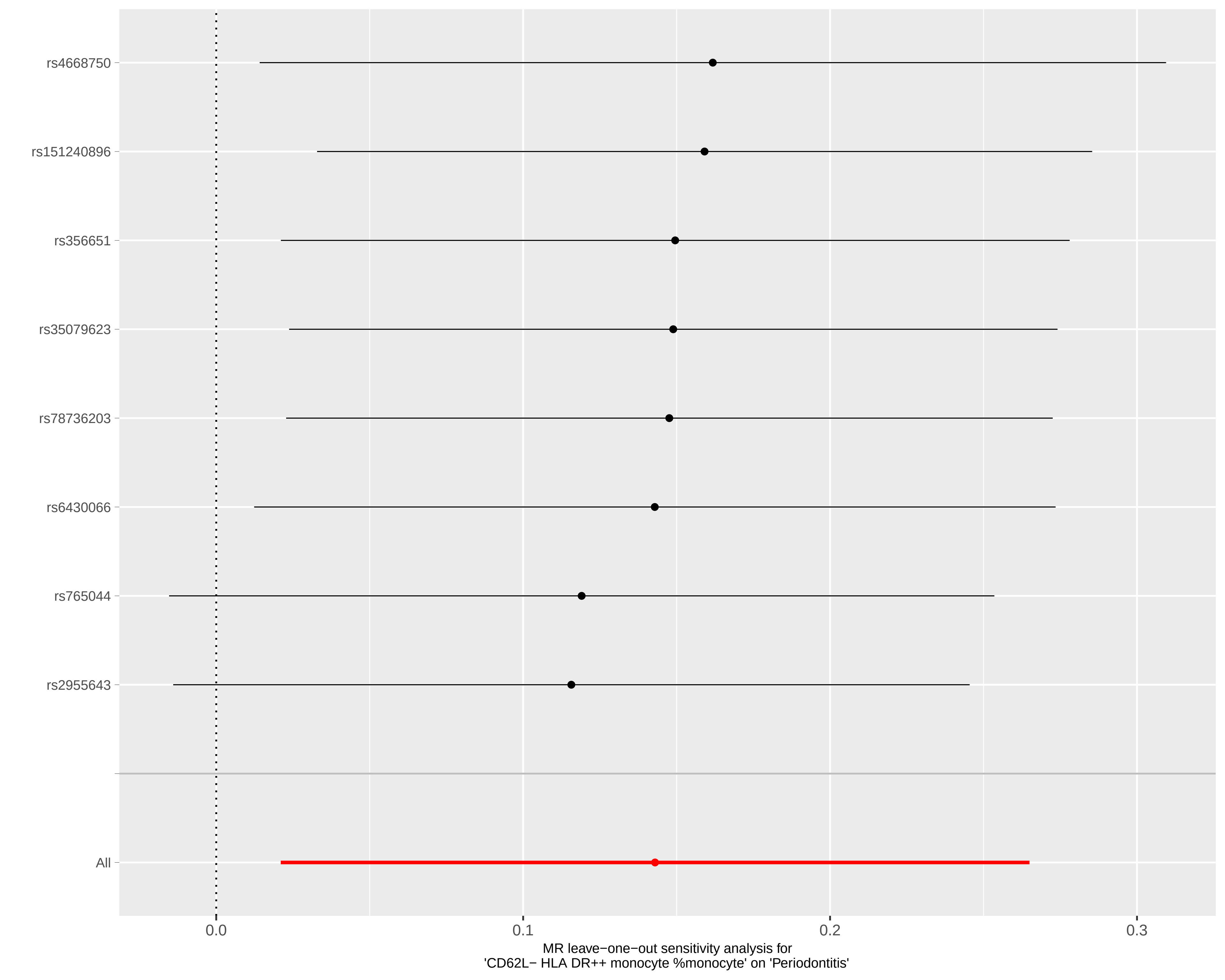

rs4668750
rs151240896
rs356651
rs35079623
rs78736203
rs6430066
rs765044
rs2955643
All
0.0
0.1
0.2
0.3
MR leave−one−out sensitivity analysis for
'CD62L− HLA DR++ monocyte %monocyte' on 'Periodontitis'

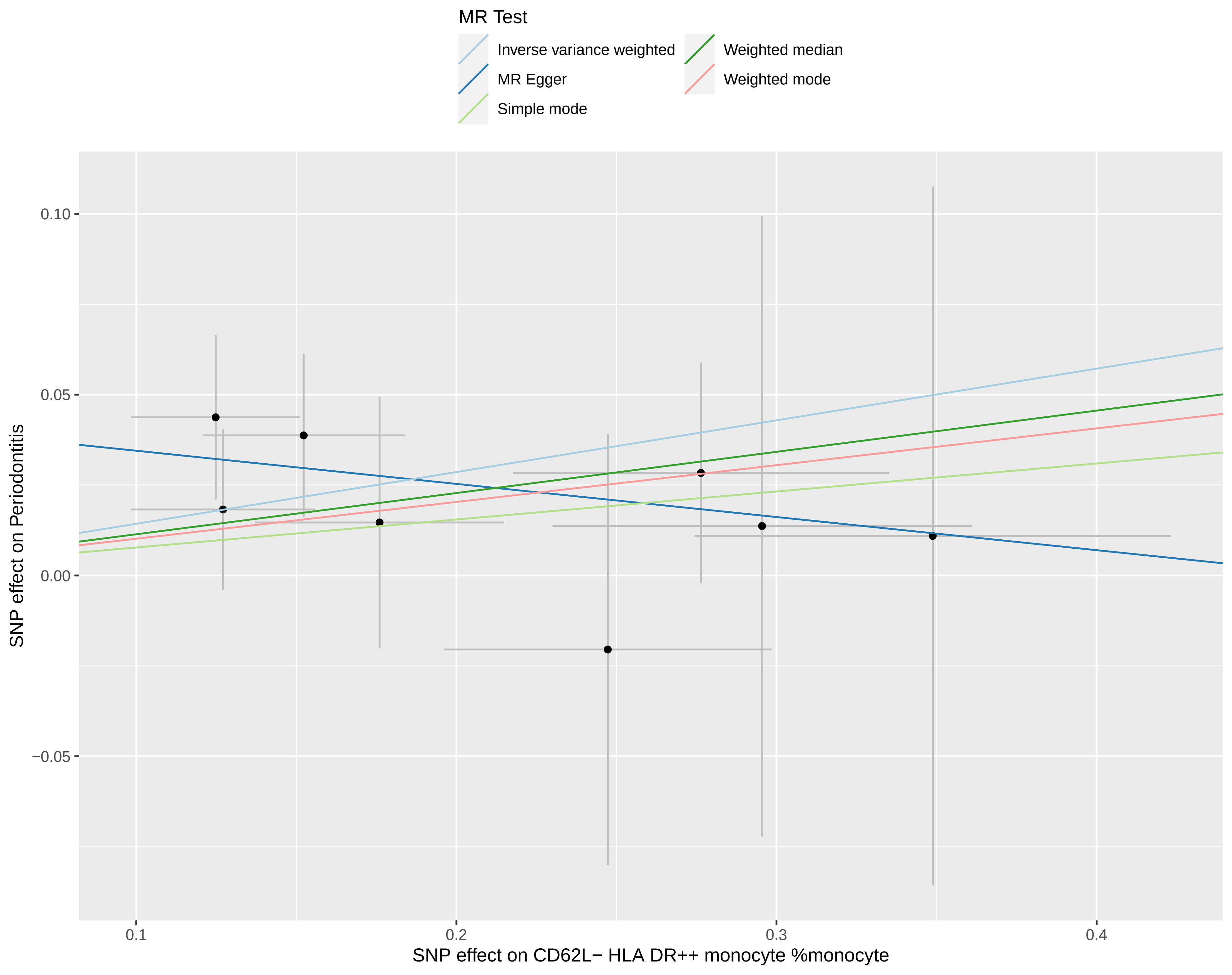
MR Test
Inverse variance weighted
MR Egger
Simple mode
Weighted median
Weighted mode
0.10
0.05
0.00
−0.05
0.1
0.2
0.3
0.4
SNP effect on Periodontitis
SNP effect on CD62L− HLA DR++ monocyte %monocyte

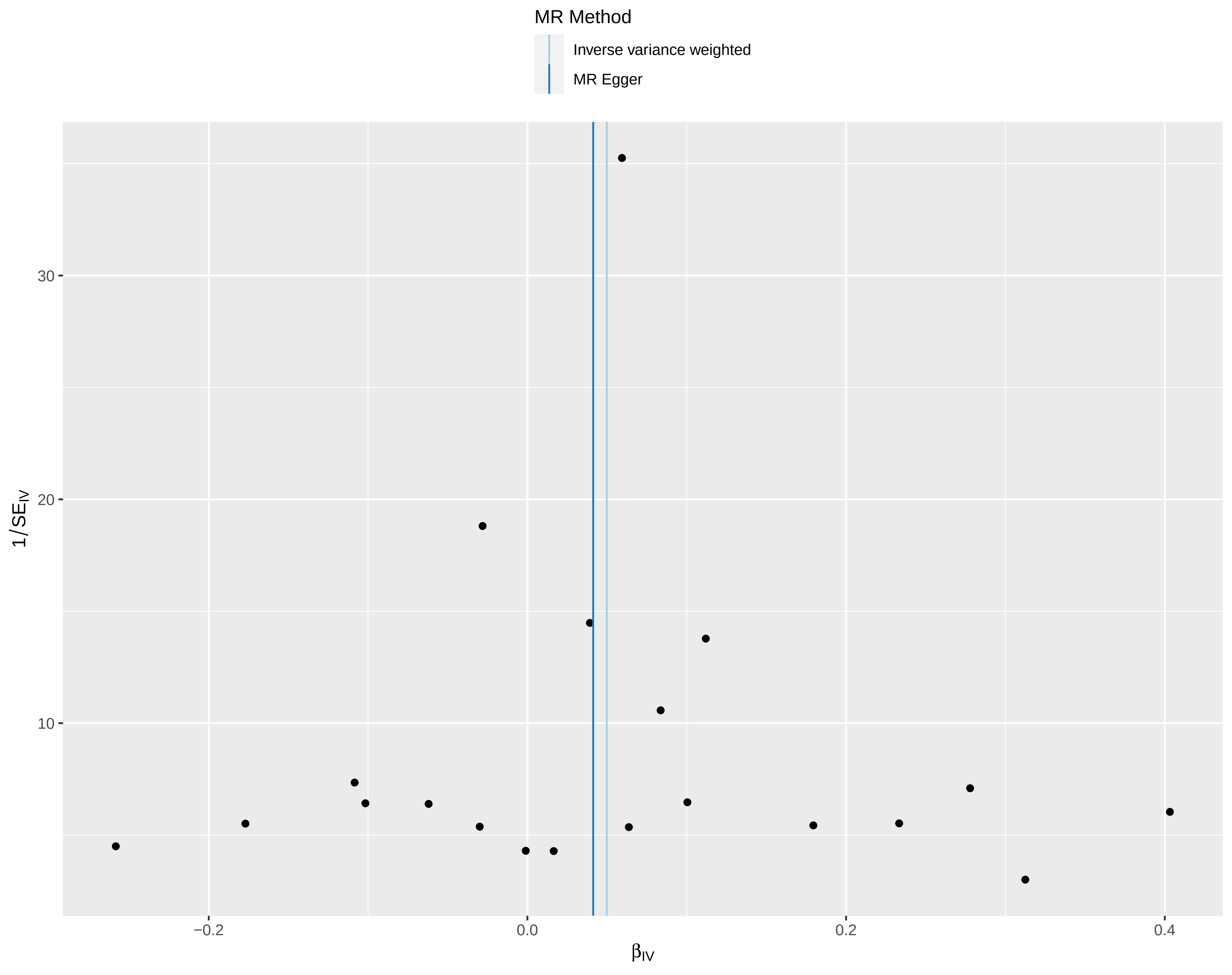

MR Method
Inverse variance weighted
MR Egger
30
20
10
1/SE_IV
−0.2
0.0
0.2
0.4
β_IV

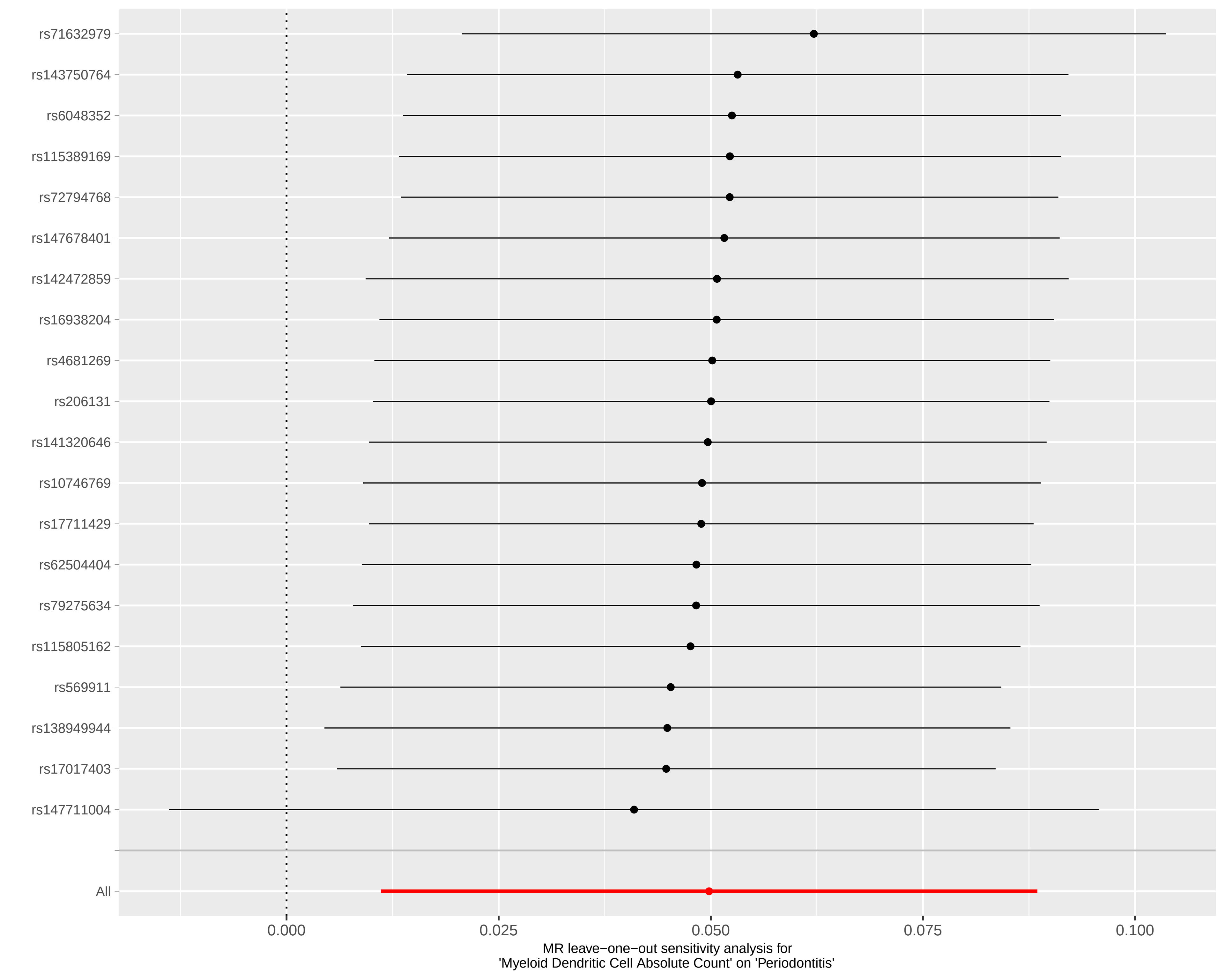
rs71632979
rs143750764
rs6048352
rs115389169
rs72794768
rs147678401
rs142472859
rs16938204
rs4681269
rs206131
rs141320646
rs10746769
rs17711429
rs62504404
rs79275634
rs115805162
rs569911
rs138949944
rs17017403
rs147711004
All
0.000
0.025
0.050
0.075
0.100
MR leave−one−out sensitivity analysis for
'Myeloid Dendritic Cell Absolute Count' on 'Periodontitis'

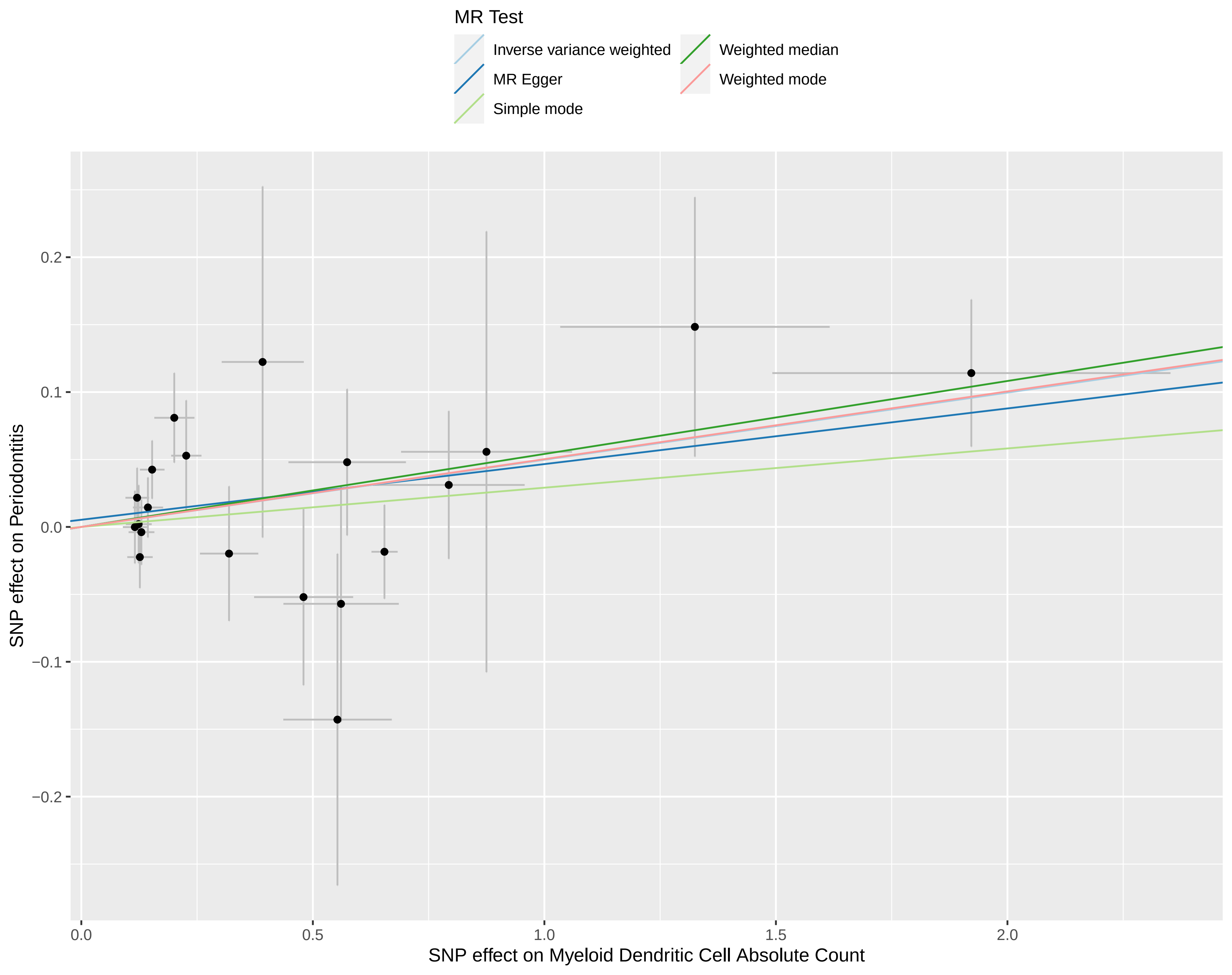
MR Test
Inverse variance weighted
MR Egger
Simple mode
Weighted median
Weighted mode
SNP effect on Periodontitis
SNP effect on Myeloid Dendritic Cell Absolute Count
0.2
0.1
0.0
−0.1
−0.2
0.0
0.5
1.0
1.5
2.0

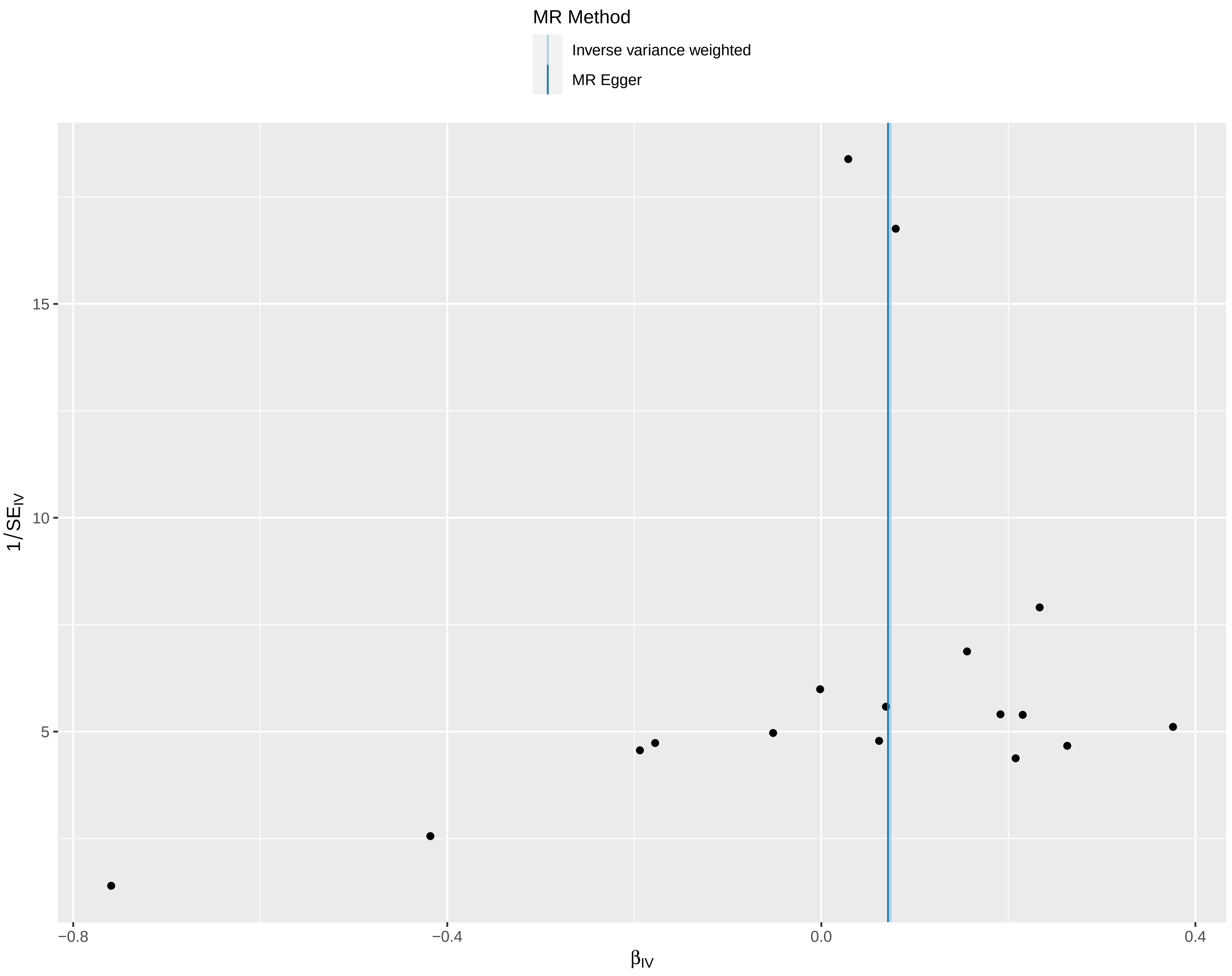
MR Method
Inverse variance weighted
MR Egger
15
10
5
1/SE$_{IV}$
−0.8
−0.4
0.0
0.4
β$_{IV}$

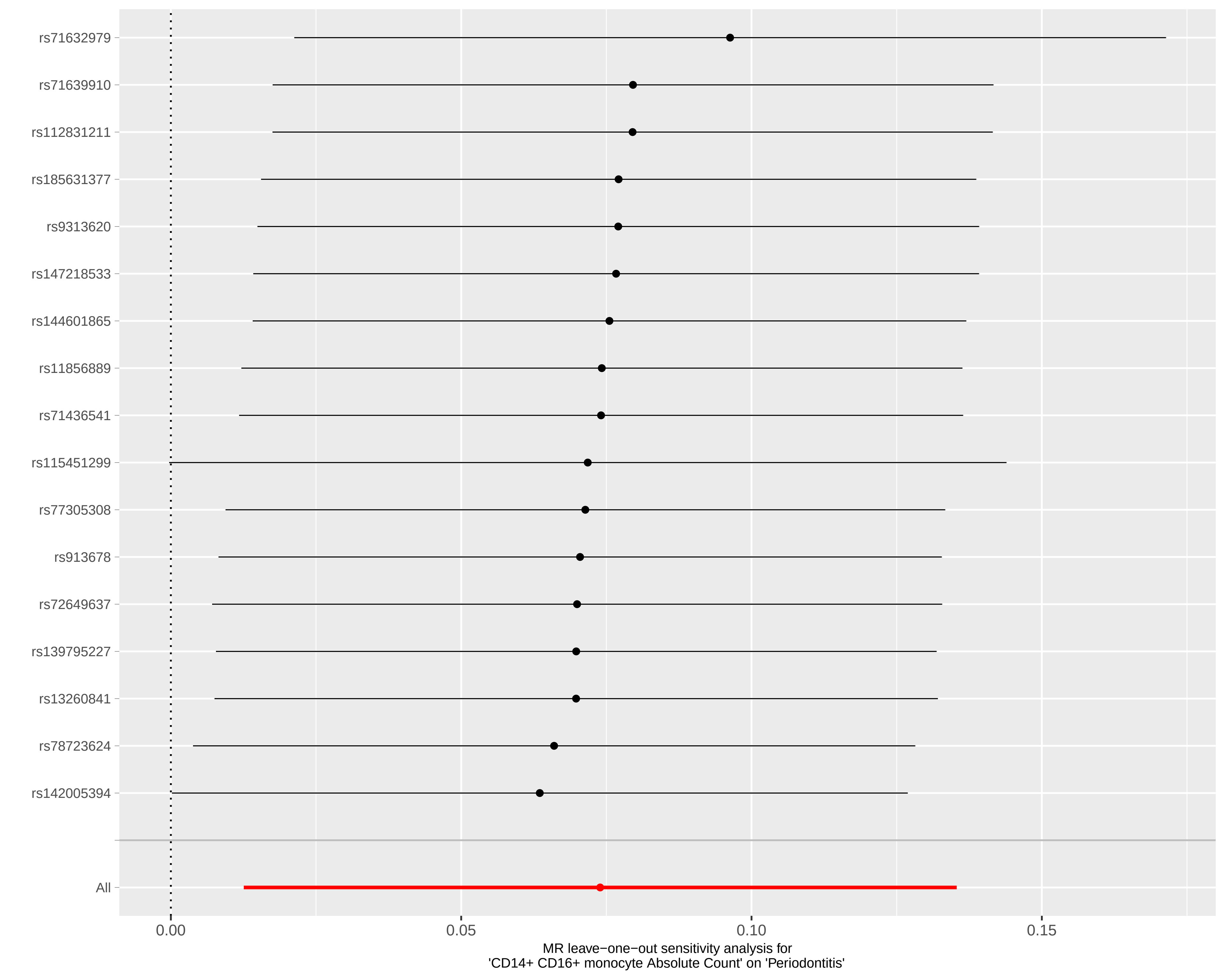
rs71632979
rs71639910
rs112831211
rs185631377
rs9313620
rs147218533
rs144601865
rs11856889
rs71436541
rs115451299
rs77305308
rs913678
rs72649637
rs139795227
rs13260841
rs78723624
rs142005394
All
0.00
0.05
0.10
0.15
MR leave−one−out sensitivity analysis for
'CD14+ CD16+ monocyte Absolute Count' on 'Periodontitis'

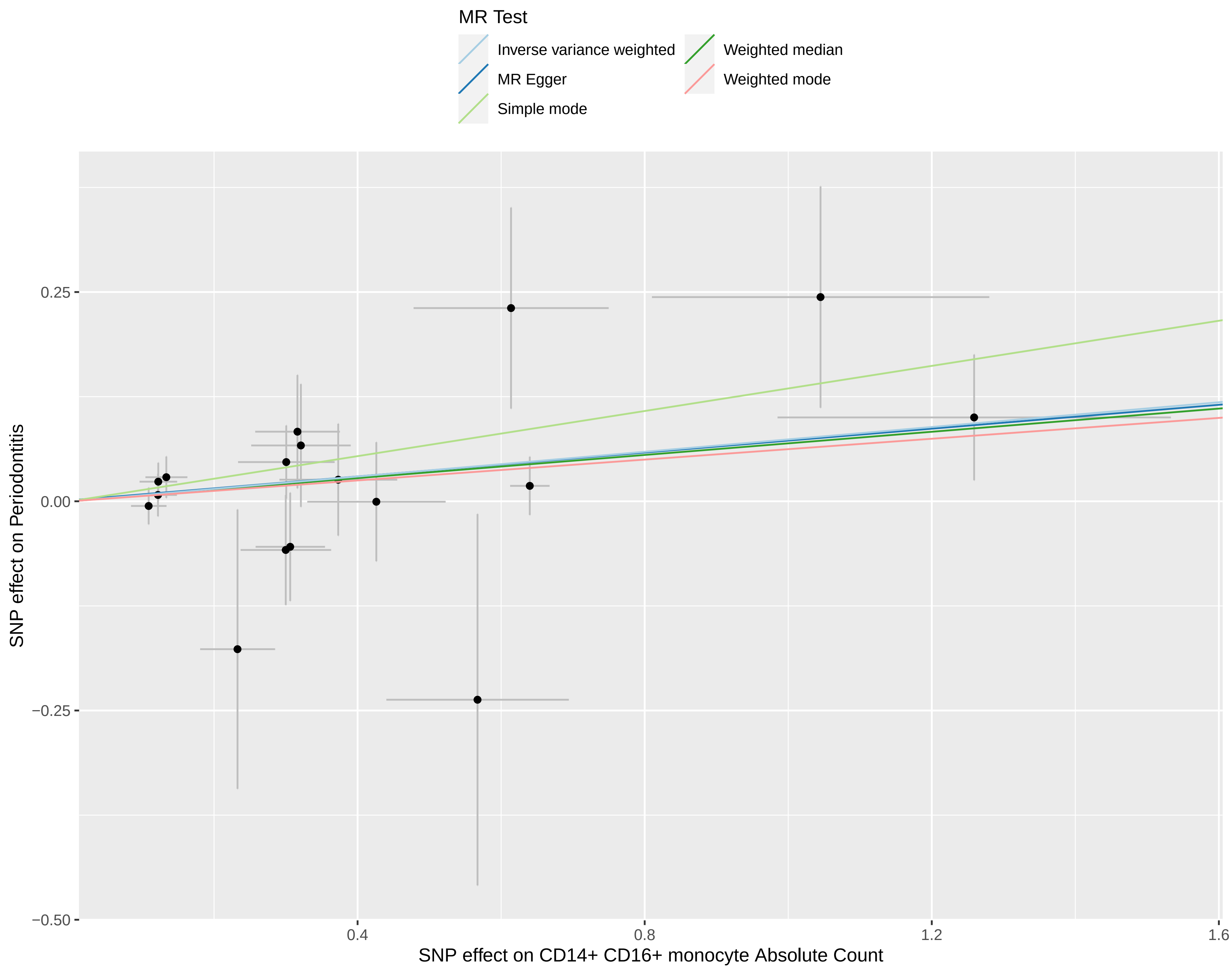

MR Test
Inverse variance weighted
MR Egger
Simple mode
Weighted median
Weighted mode
0.25
0.00
−0.25
−0.50
0.4
0.8
1.2
1.6
SNP effect on Periodontitis
SNP effect on CD14+ CD16+ monocyte Absolute Count

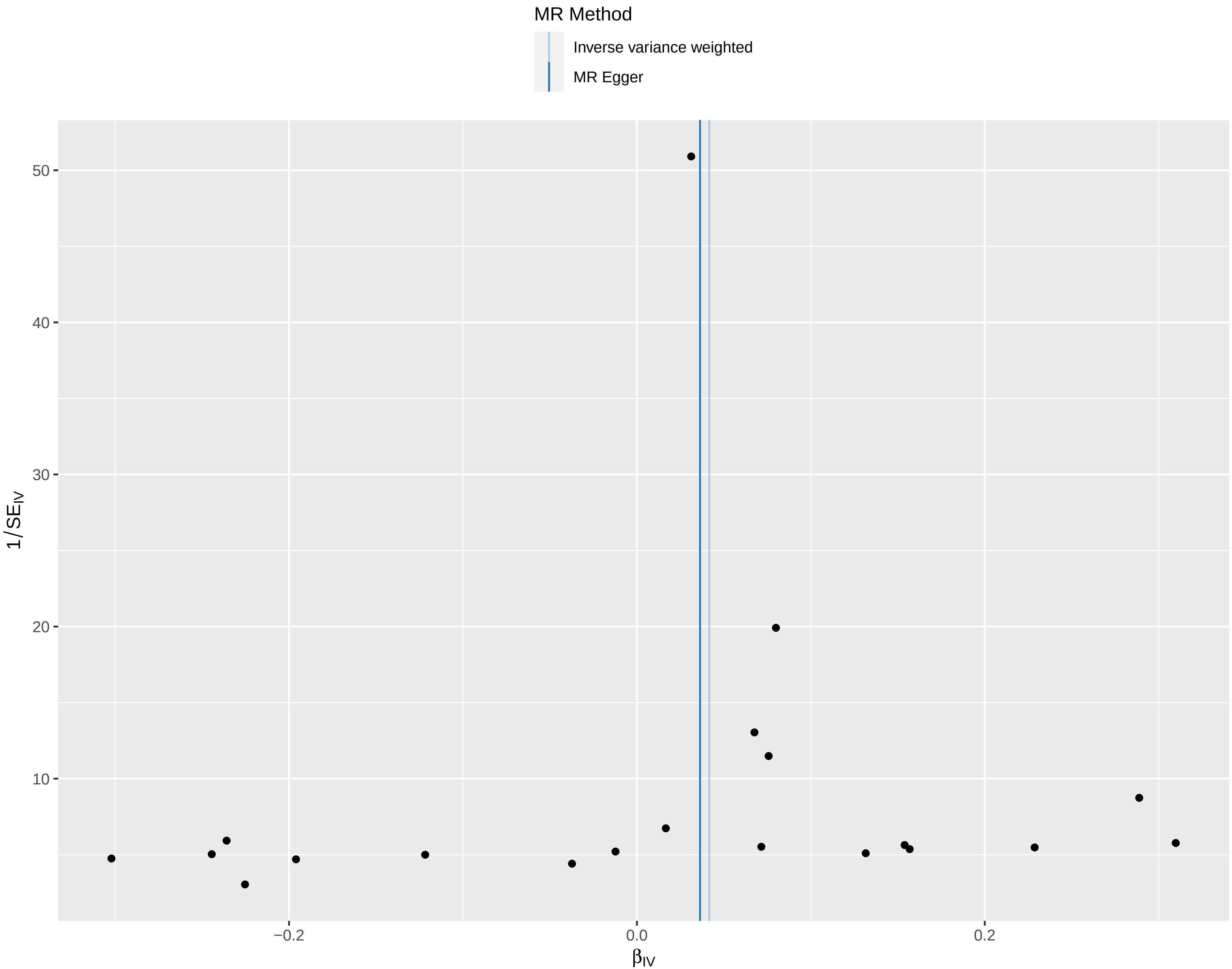
MR Method
Inverse variance weighted
MR Egger
50
40
30
20
10
$1/SE_{IV}$
−0.2
0.0
0.2
β_{IV}

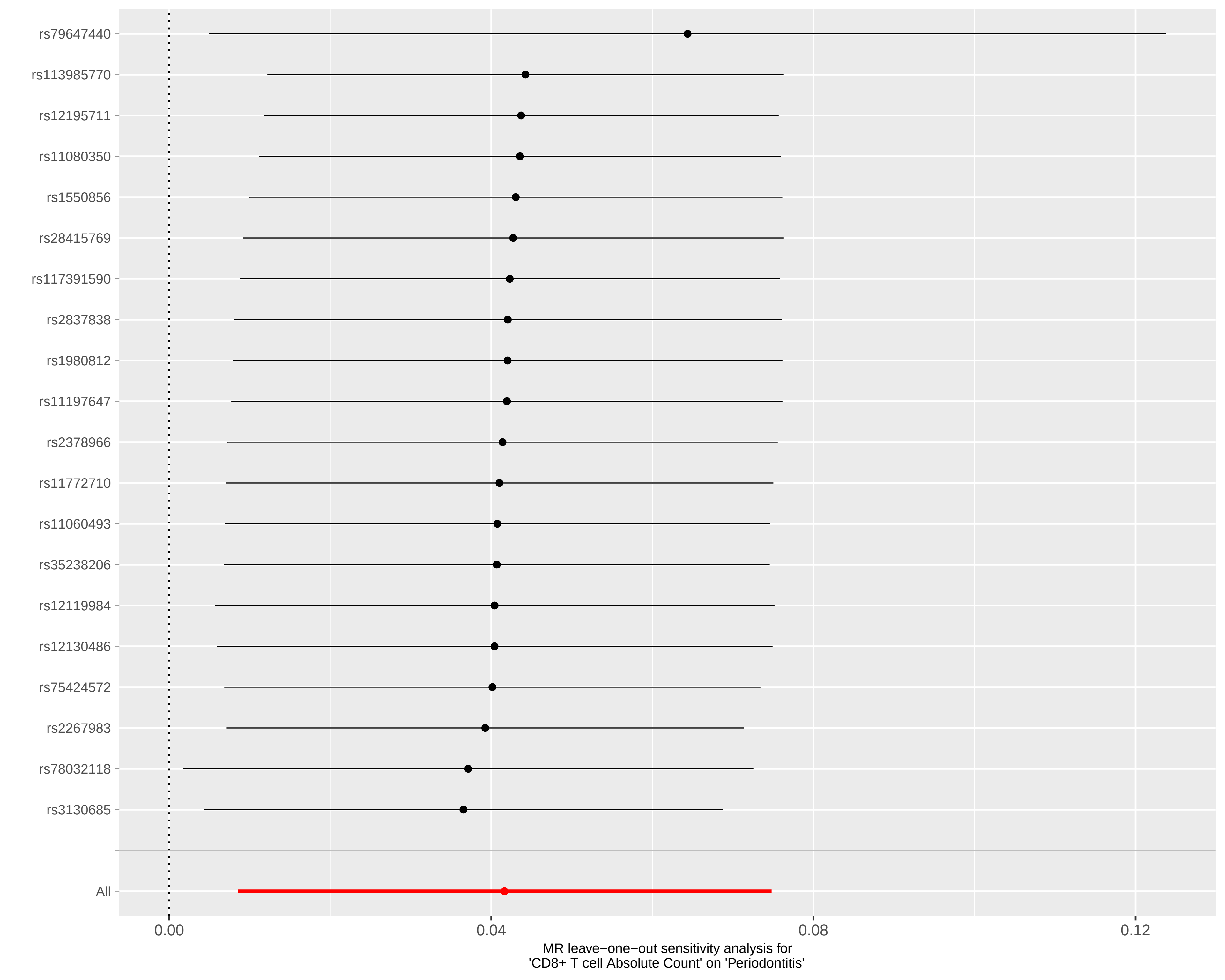
rs79647440
rs113985770
rs12195711
rs11080350
rs1550856
rs28415769
rs117391590
rs2837838
rs1980812
rs11197647
rs2378966
rs11772710
rs11060493
rs35238206
rs12119984
rs12130486
rs75424572
rs2267983
rs78032118
rs3130685
All
0.00
0.04
0.08
0.12
MR leave−one−out sensitivity analysis for
'CD8+ T cell Absolute Count' on 'Periodontitis'

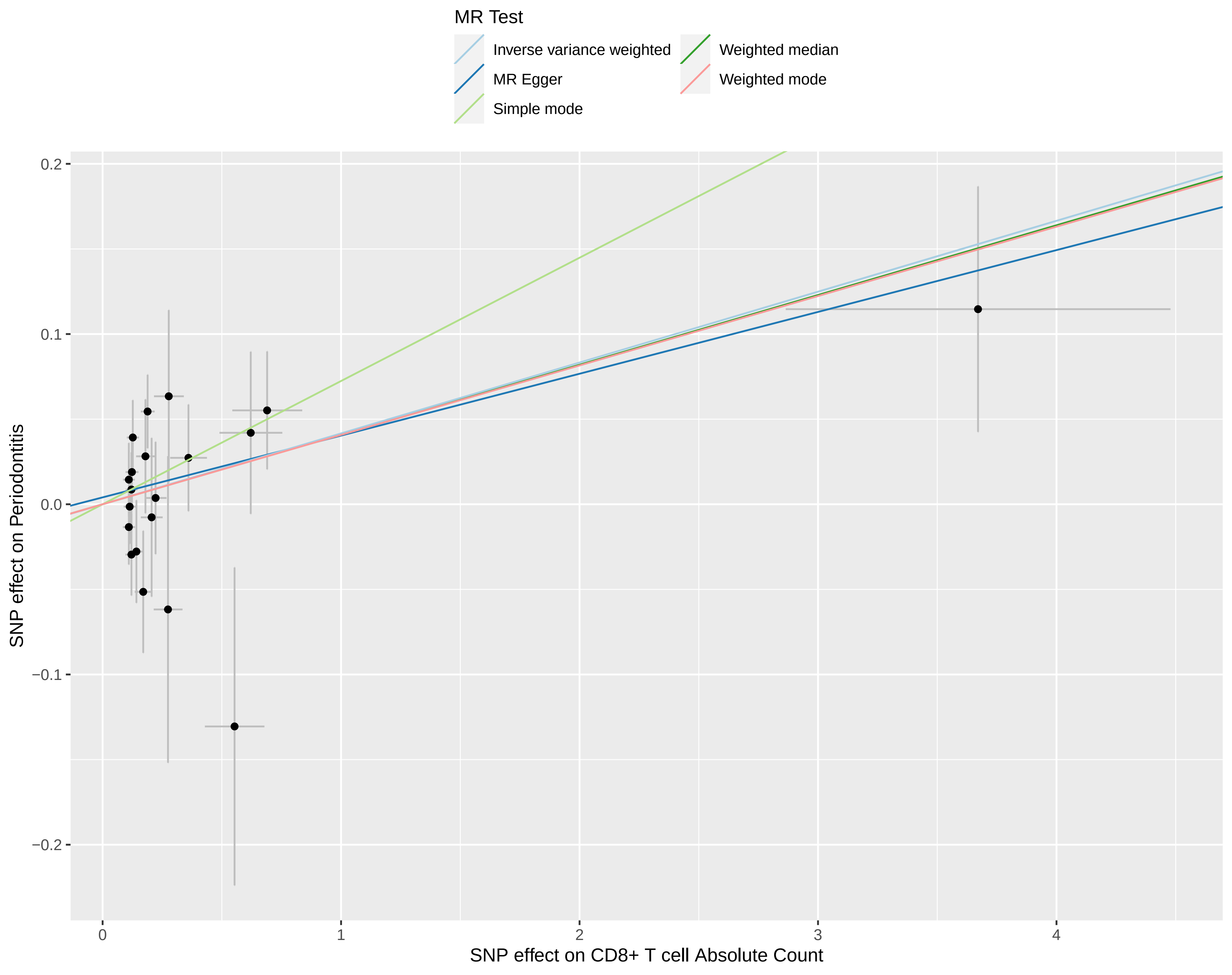

MR Test
Inverse variance weighted
MR Egger
Simple mode
Weighted median
Weighted mode
0.2
0.1
0.0
−0.1
−0.2
SNP effect on Periodontitis
0
1
2
3
4
SNP effect on CD8+ T cell Absolute Count

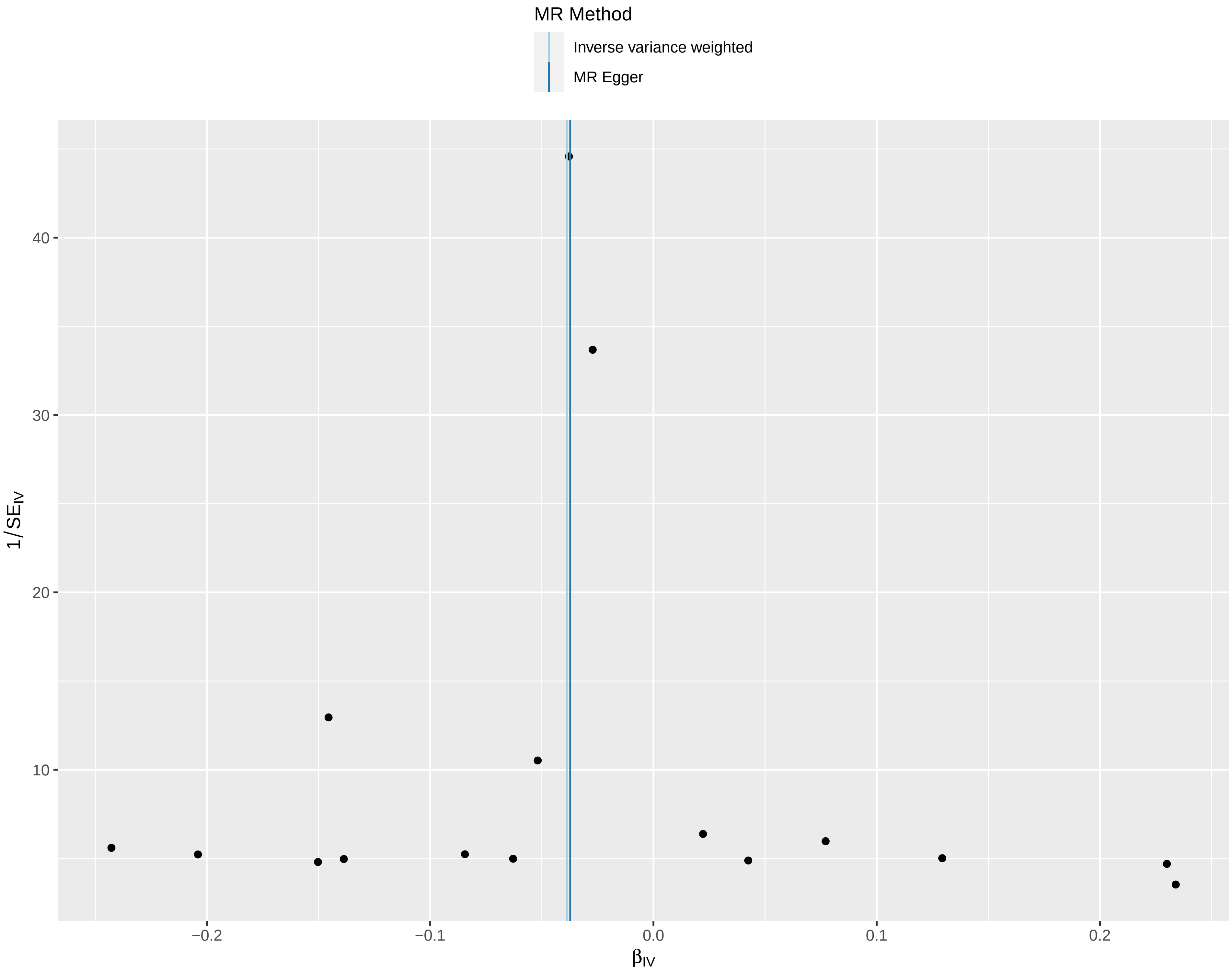
MR Method
Inverse variance weighted
MR Egger
1/SE_IV
β_IV
40
30
20
10
−0.2
−0.1
0.0
0.1
0.2

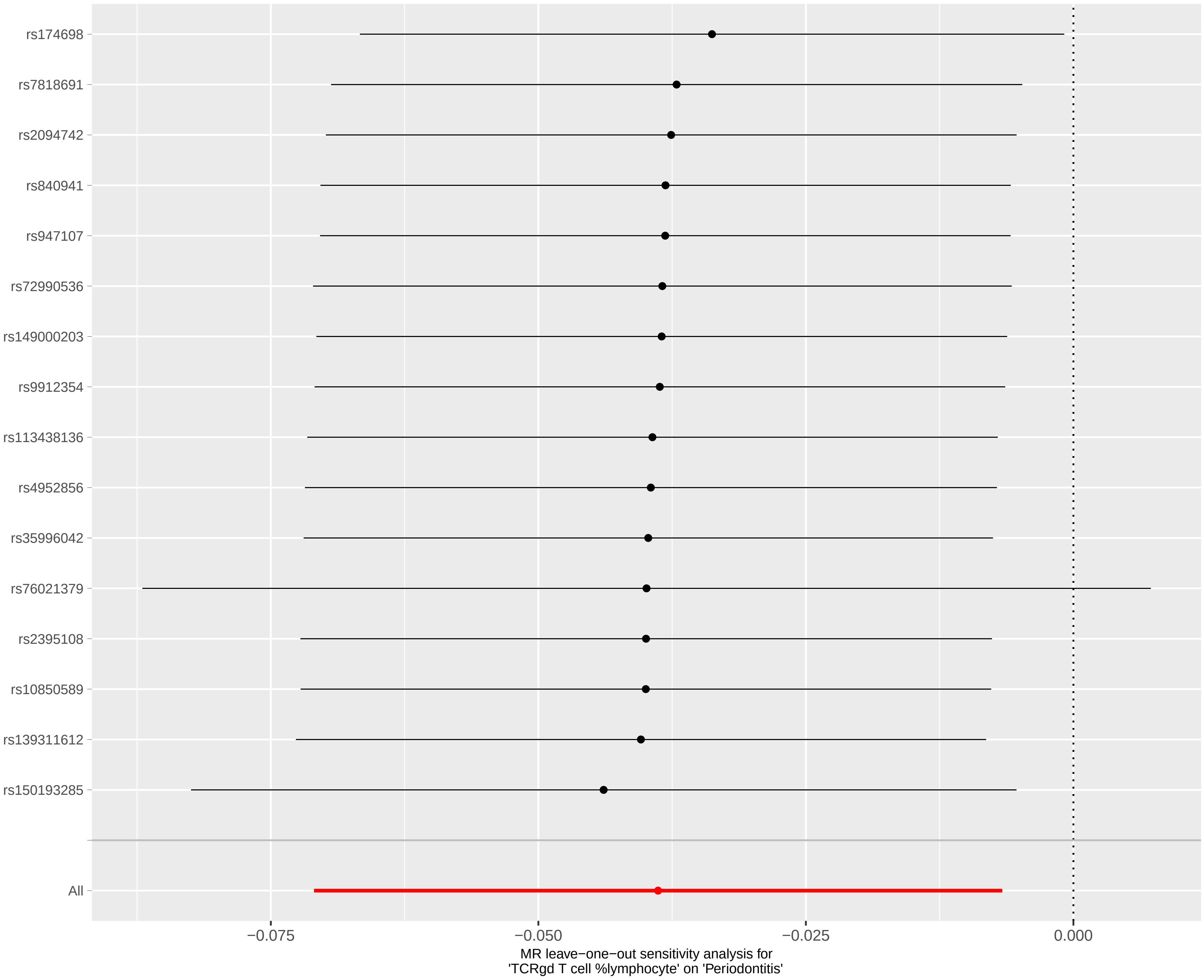
rs174698
rs7818691
rs2094742
rs840941
rs947107
rs72990536
rs149000203
rs9912354
rs113438136
rs4952856
rs35996042
rs76021379
rs2395108
rs10850589
rs139311612
rs150193285
All
−0.075
−0.050
−0.025
0.000
MR leave−one−out sensitivity analysis for
'TCRgd T cell %lymphocyte' on 'Periodontitis'

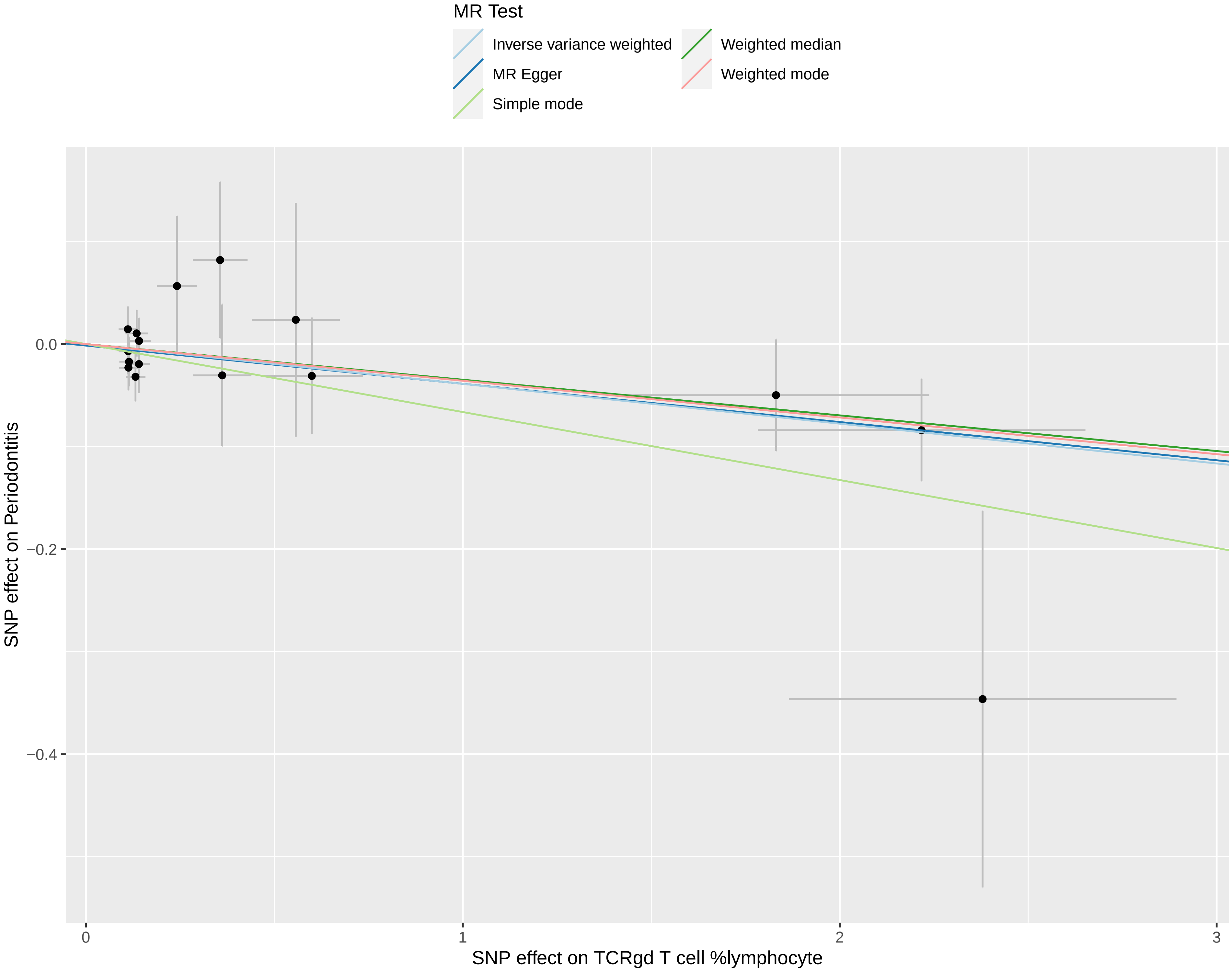
MR Test
Inverse variance weighted
MR Egger
Simple mode
Weighted median
Weighted mode
SNP effect on Periodontitis
0.0
−0.2
−0.4
0
1
2
3
SNP effect on TCRgd T cell %lymphocyte

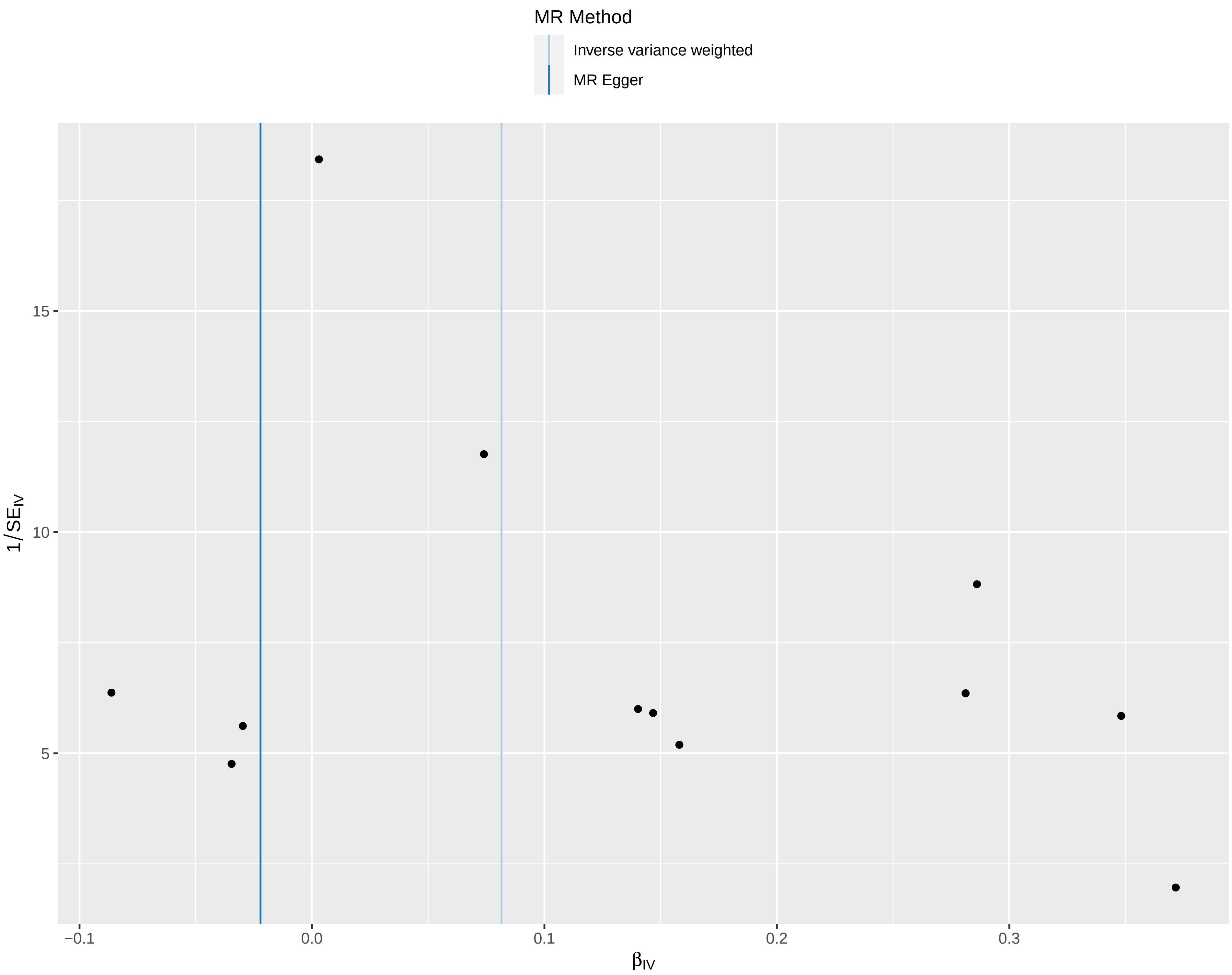
MR Method
Inverse variance weighted
MR Egger
15
10
5
1/SE_IV
−0.1
0.0
0.1
0.2
0.3
β_IV

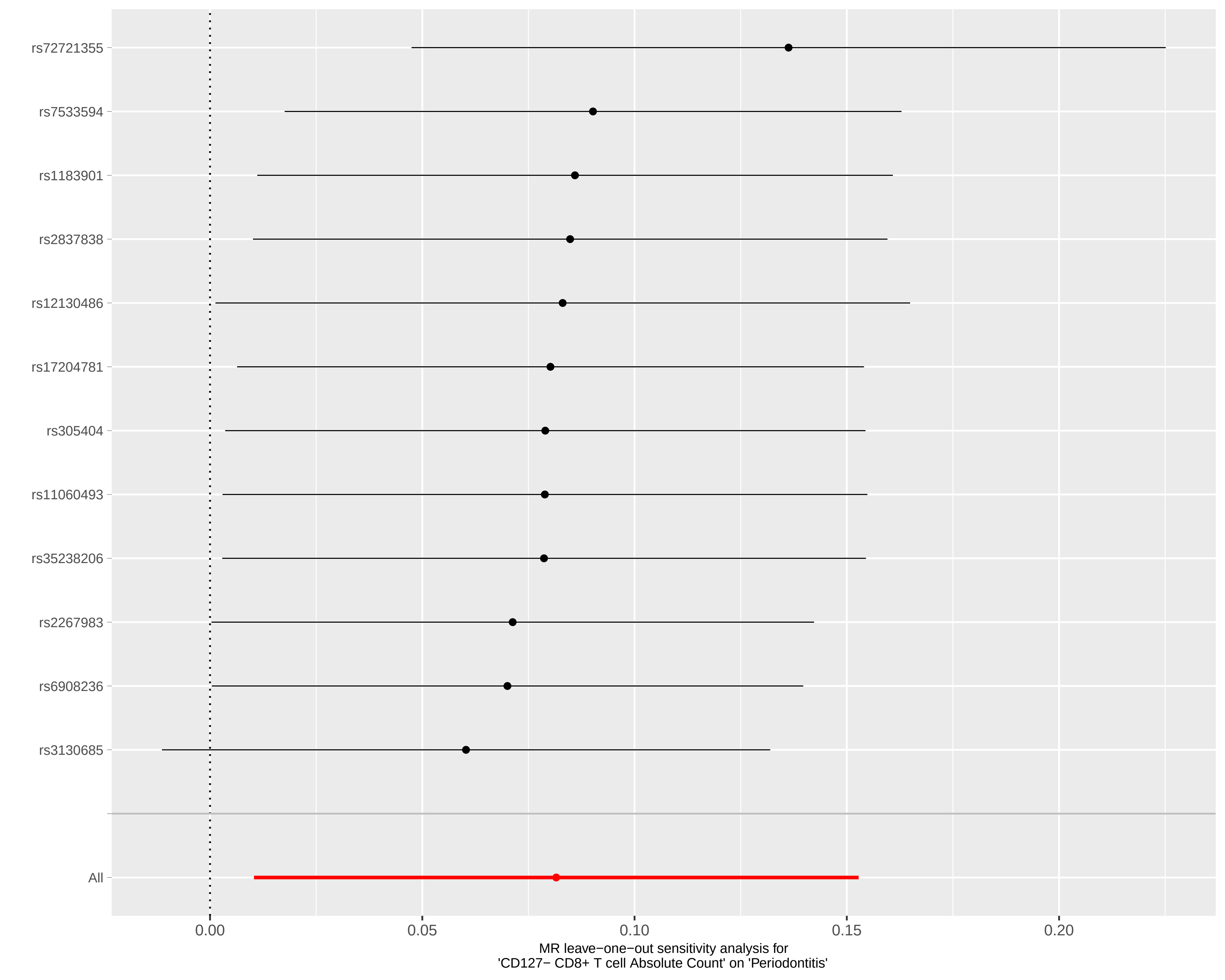
rs72721355
rs7533594
rs1183901
rs2837838
rs12130486
rs17204781
rs305404
rs11060493
rs35238206
rs2267983
rs6908236
rs3130685
All
0.00
0.05
0.10
0.15
0.20
MR leave−one−out sensitivity analysis for
'CD127− CD8+ T cell Absolute Count' on 'Periodontitis'

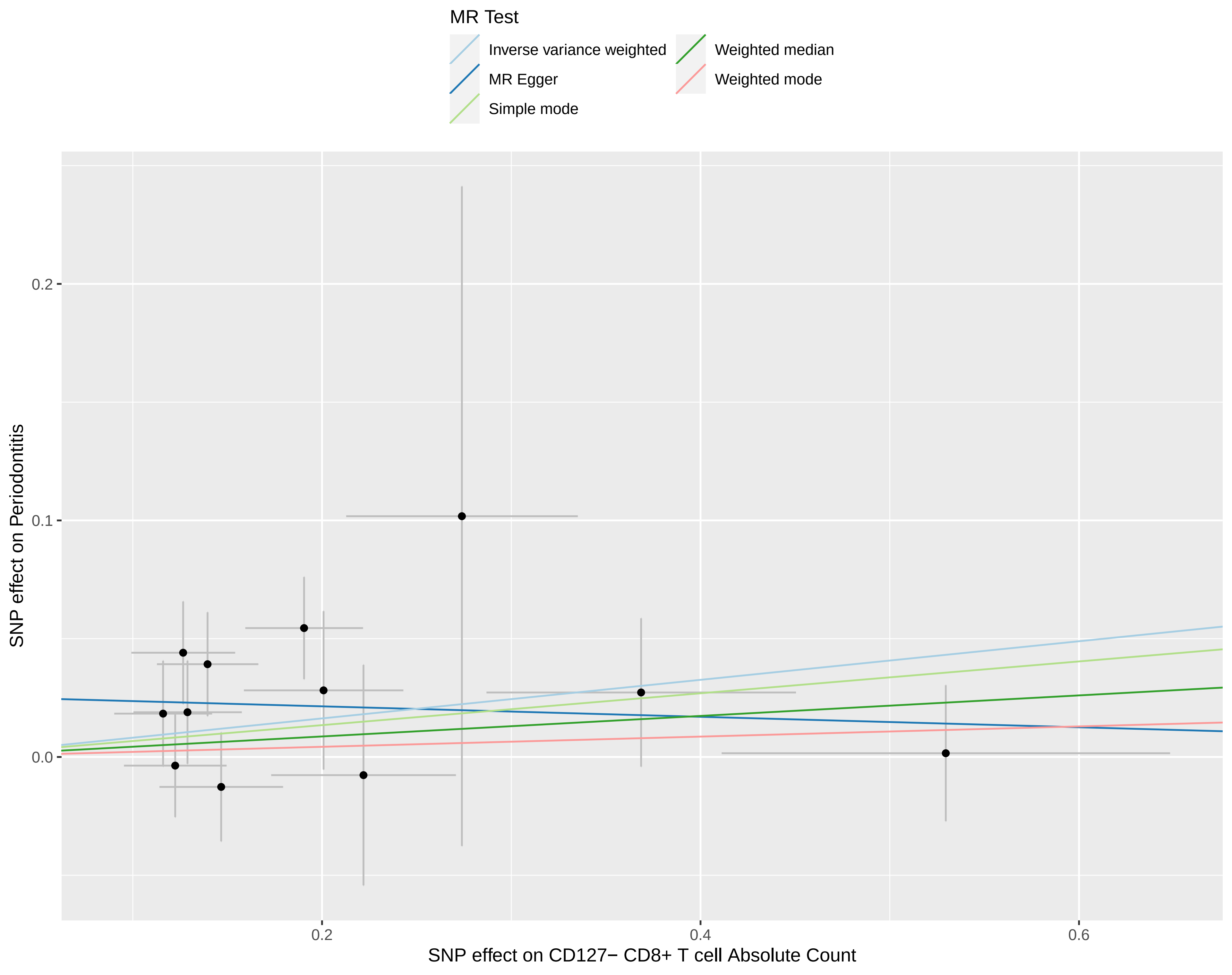

MR Test
Inverse variance weighted
MR Egger
Simple mode
Weighted median
Weighted mode
0.2
0.1
0.0
SNP effect on Periodontitis
0.2
0.4
0.6
SNP effect on CD127− CD8+ T cell Absolute Count

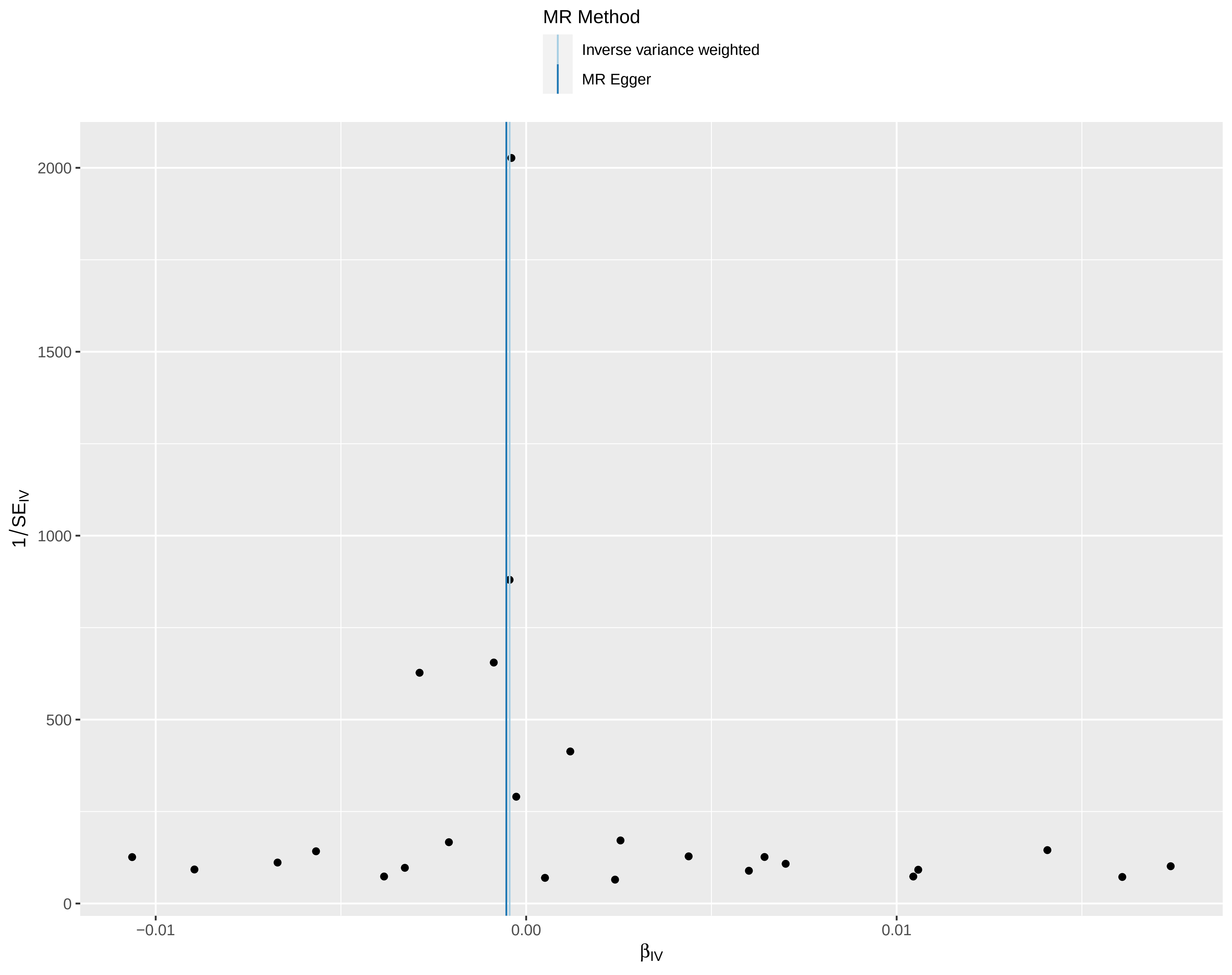
MR Method
Inverse variance weighted
MR Egger
2000
1500
1000
500
0
$1/SE_{IV}$
−0.01
0.00
0.01
β_{IV}

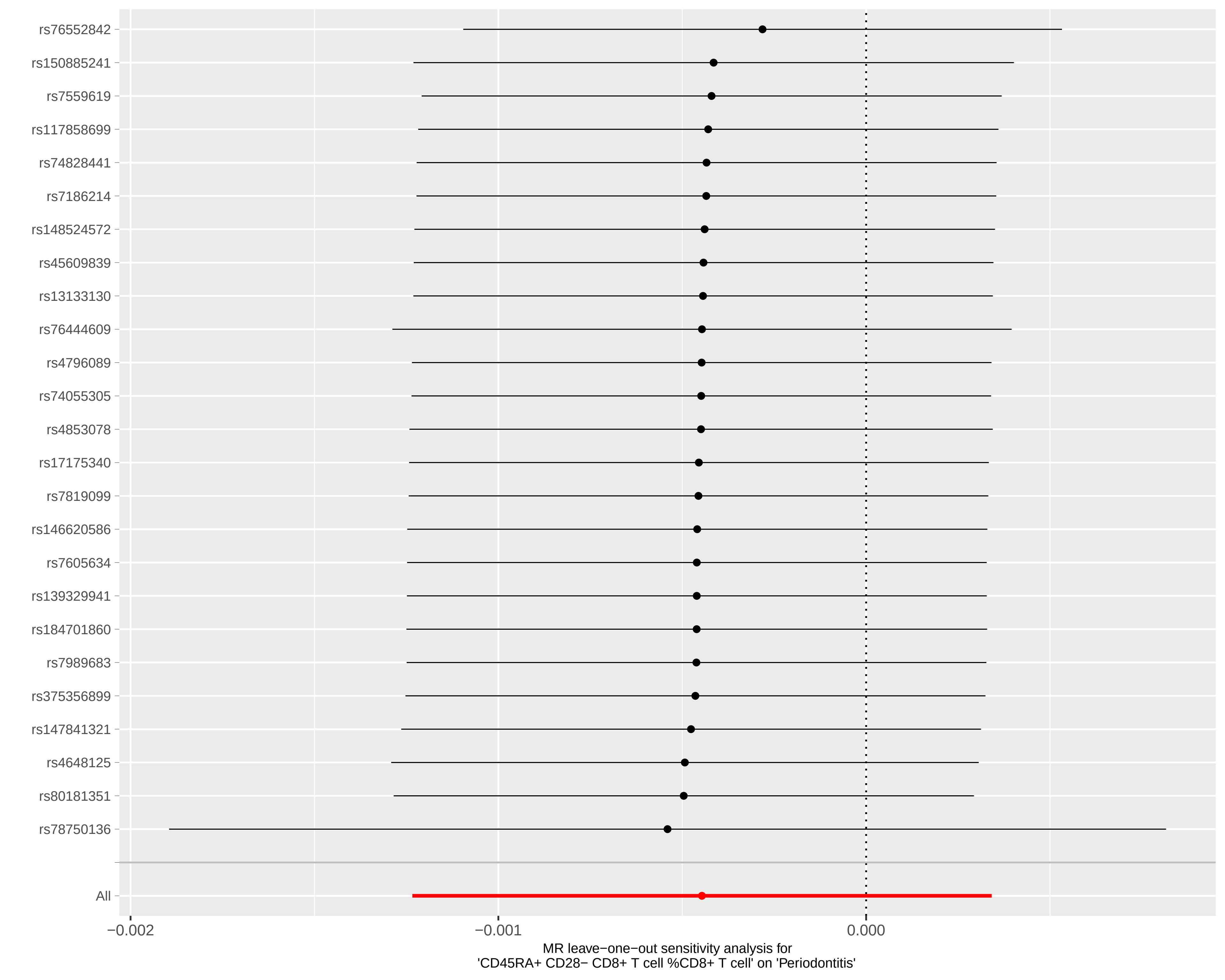

rs76552842
rs150885241
rs7559619
rs117858699
rs74828441
rs7186214
rs148524572
rs45609839
rs13133130
rs76444609
rs4796089
rs74055305
rs4853078
rs17175340
rs7819099
rs146620586
rs7605634
rs139329941
rs184701860
rs7989683
rs375356899
rs147841321
rs4648125
rs80181351
rs78750136
All
−0.002
−0.001
0.000
MR leave−one−out sensitivity analysis for
'CD45RA+ CD28− CD8+ T cell %CD8+ T cell' on 'Periodontitis'

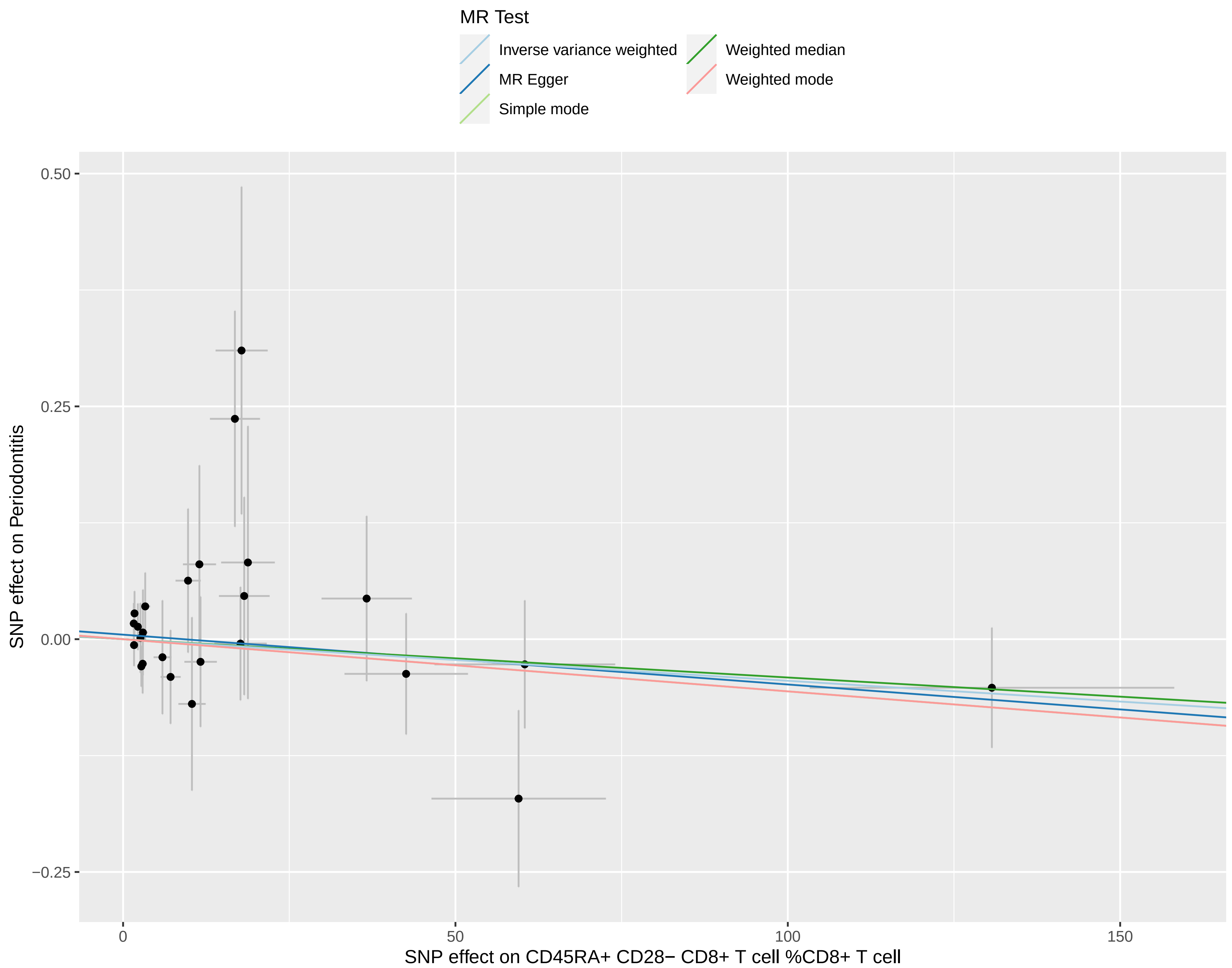

MR Test
Inverse variance weighted
MR Egger
Simple mode
Weighted median
Weighted mode
SNP effect on Periodontitis
0.50
0.25
0.00
−0.25
0
50
100
150
SNP effect on CD45RA+ CD28− CD8+ T cell %CD8+ T cell

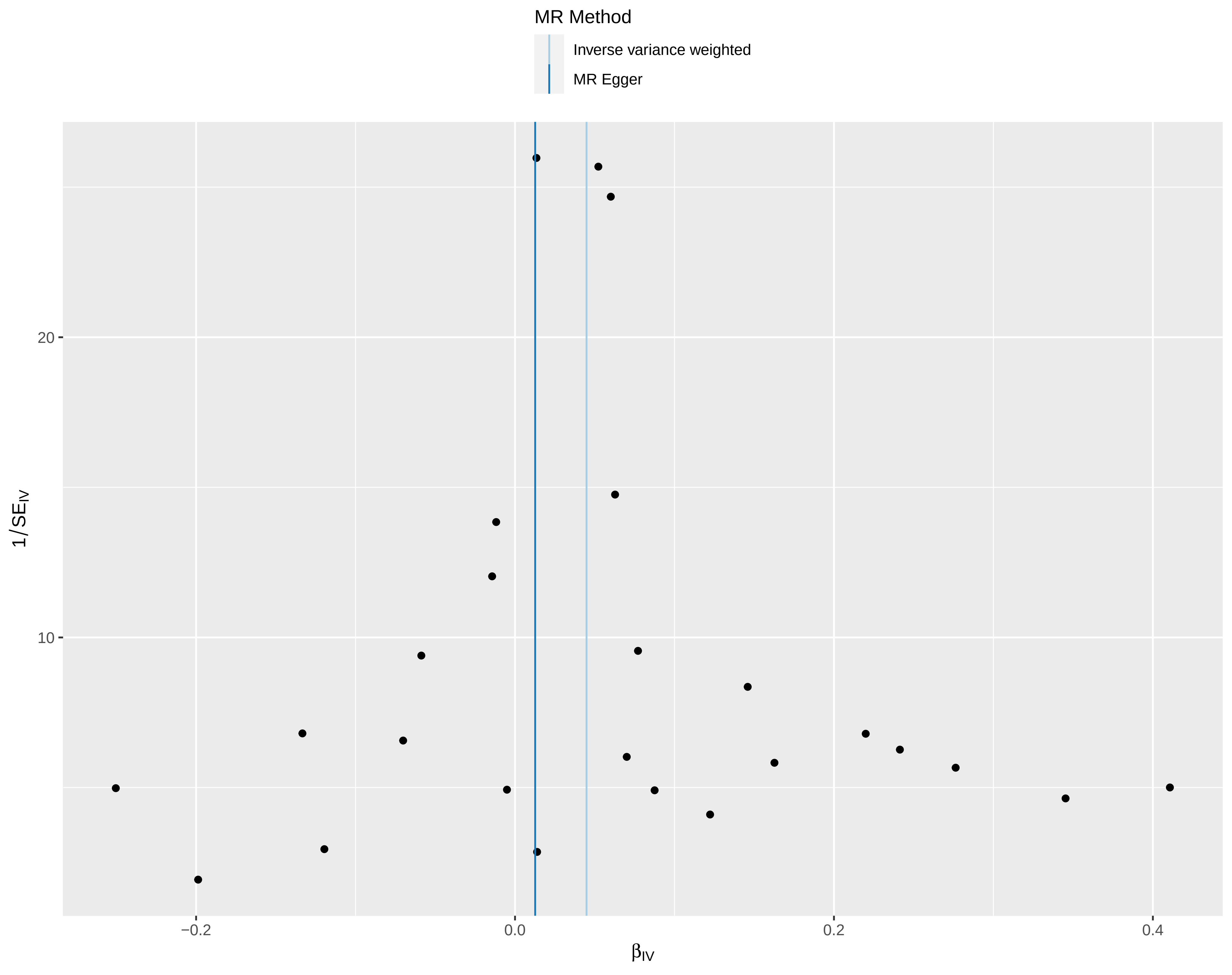
MR Method
Inverse variance weighted
MR Egger
20
10
1/SE_IV
−0.2
0.0
0.2
0.4
β_IV

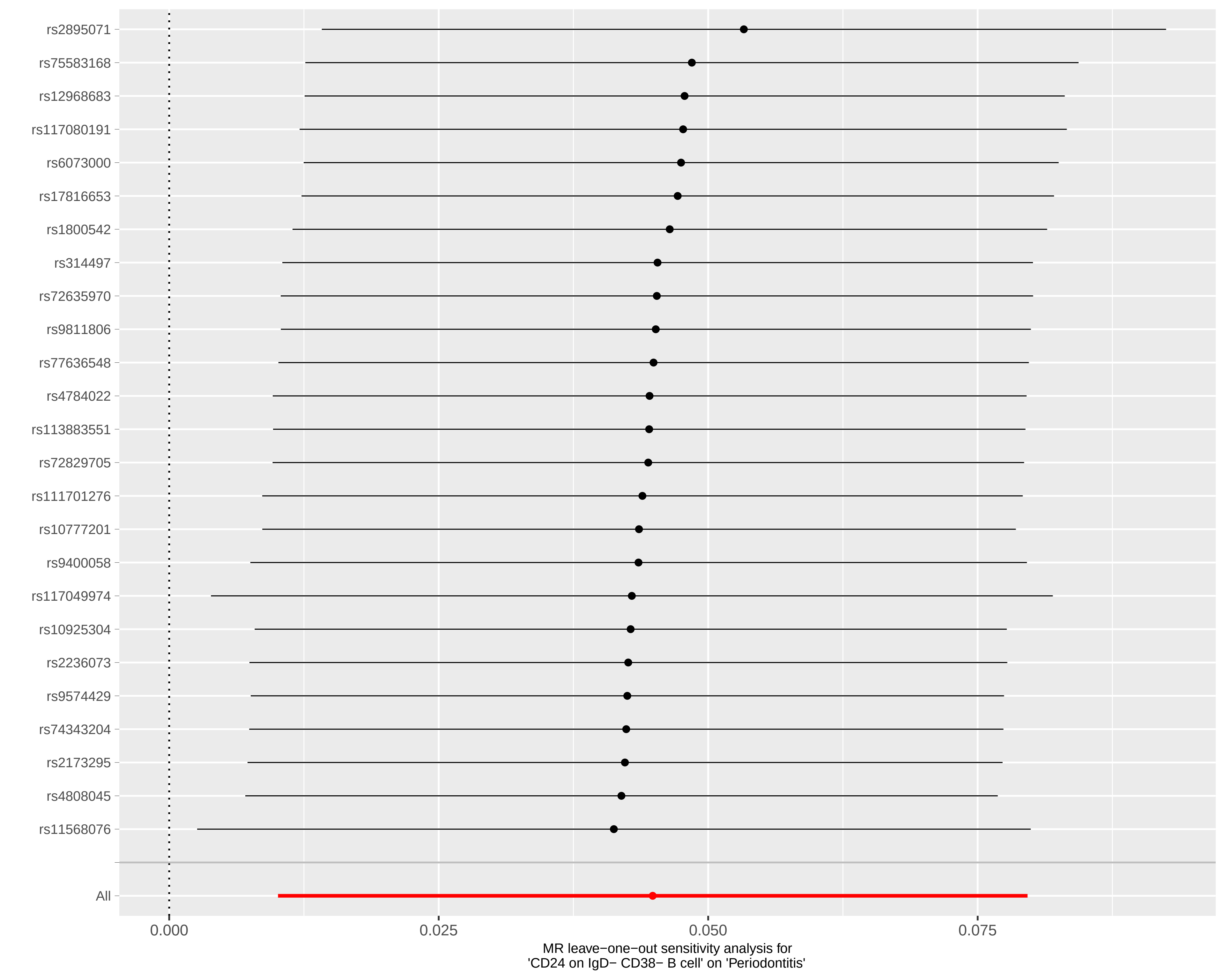
rs2895071
rs75583168
rs12968683
rs117080191
rs6073000
rs17816653
rs1800542
rs314497
rs72635970
rs9811806
rs77636548
rs4784022
rs113883551
rs72829705
rs111701276
rs10777201
rs9400058
rs117049974
rs10925304
rs2236073
rs9574429
rs74343204
rs2173295
rs4808045
rs11568076
All
0.000
0.025
0.050
0.075
MR leave−one−out sensitivity analysis for
'CD24 on IgD− CD38− B cell' on 'Periodontitis'

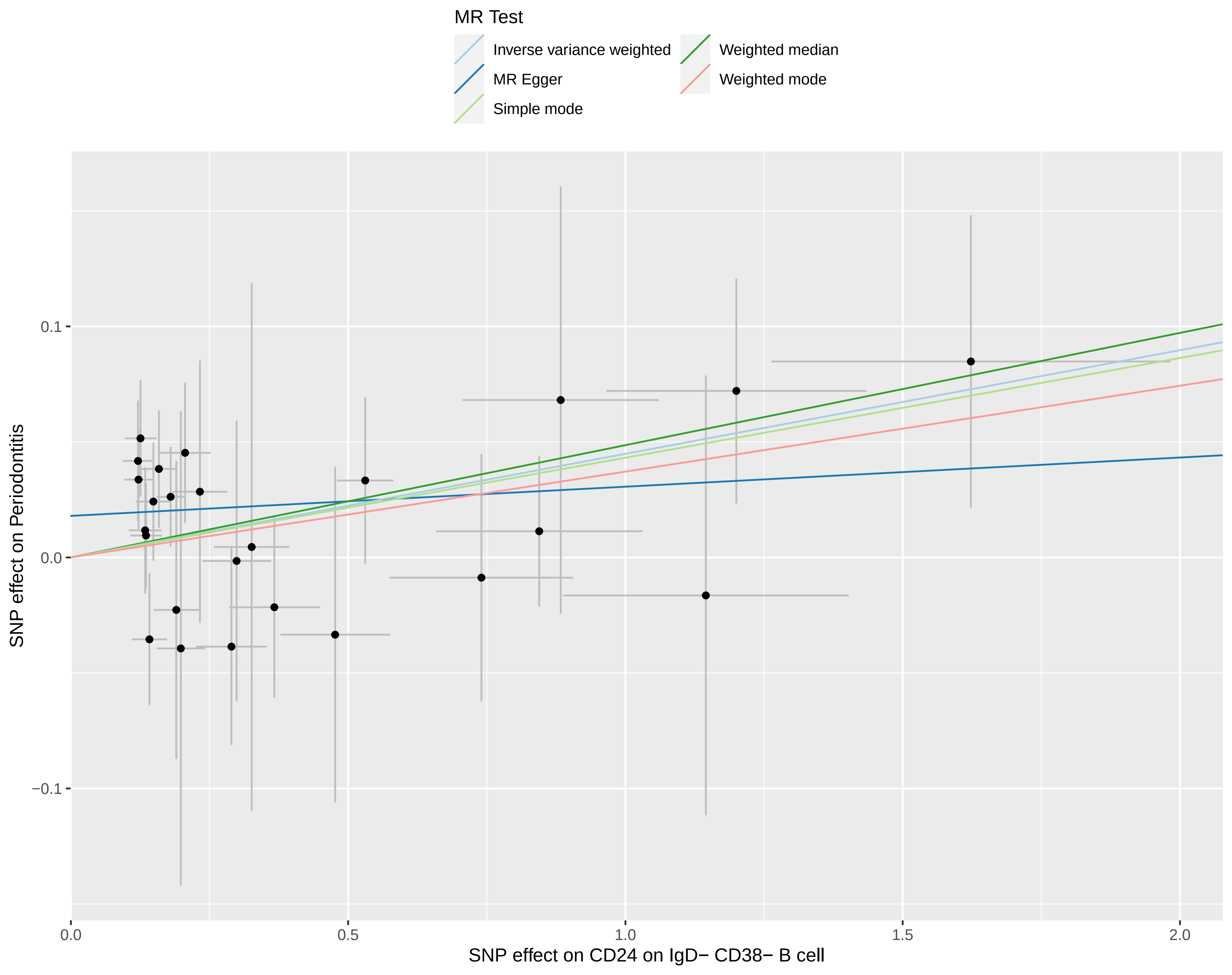
MR Test
Inverse variance weighted
MR Egger
Simple mode
Weighted median
Weighted mode
0.1
0.0
−0.1
0.0
0.5
1.0
1.5
2.0
SNP effect on Periodontitis
SNP effect on CD24 on IgD− CD38− B cell

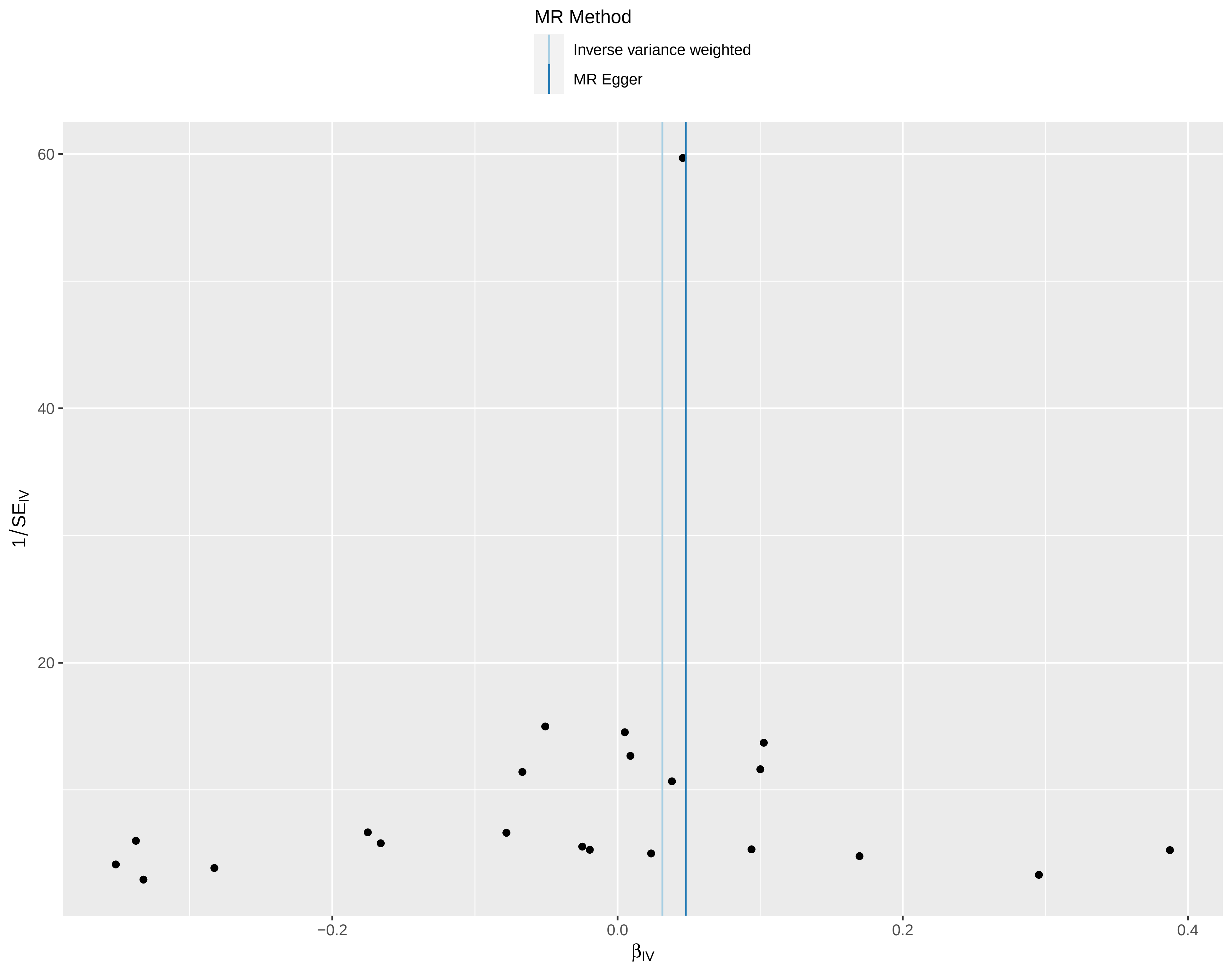
MR Method
Inverse variance weighted
MR Egger
60
40
20
1/SE_IV
−0.2
0.0
0.2
0.4
β_IV

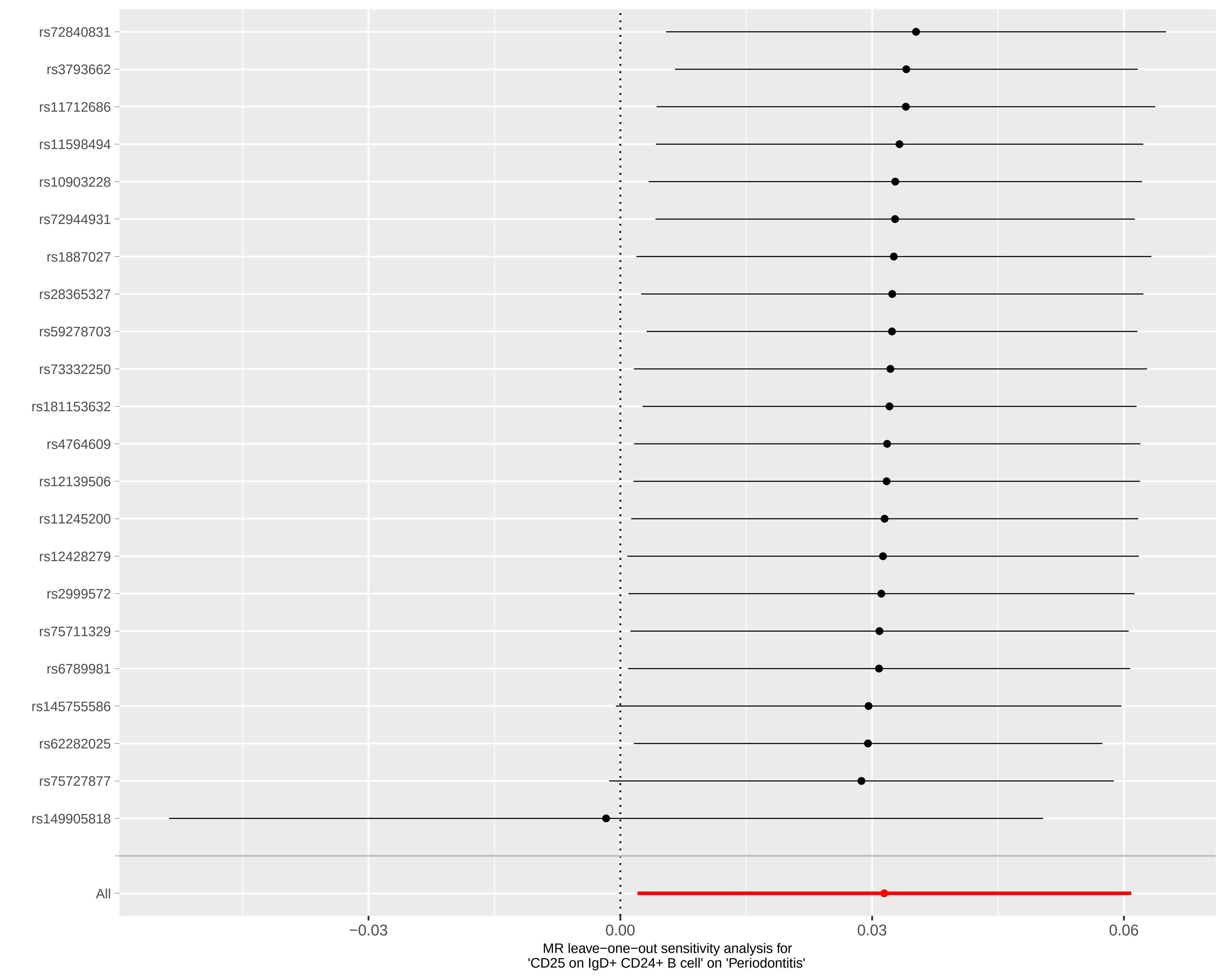
rs72840831
rs3793662
rs11712686
rs11598494
rs10903228
rs72944931
rs1887027
rs28365327
rs59278703
rs73332250
rs181153632
rs4764609
rs12139506
rs11245200
rs12428279
rs2999572
rs75711329
rs6789981
rs145755586
rs62282025
rs75727877
rs149905818
All
−0.03
0.00
0.03
0.06
MR leave−one−out sensitivity analysis for
'CD25 on IgD+ CD24+ B cell' on 'Periodontitis'

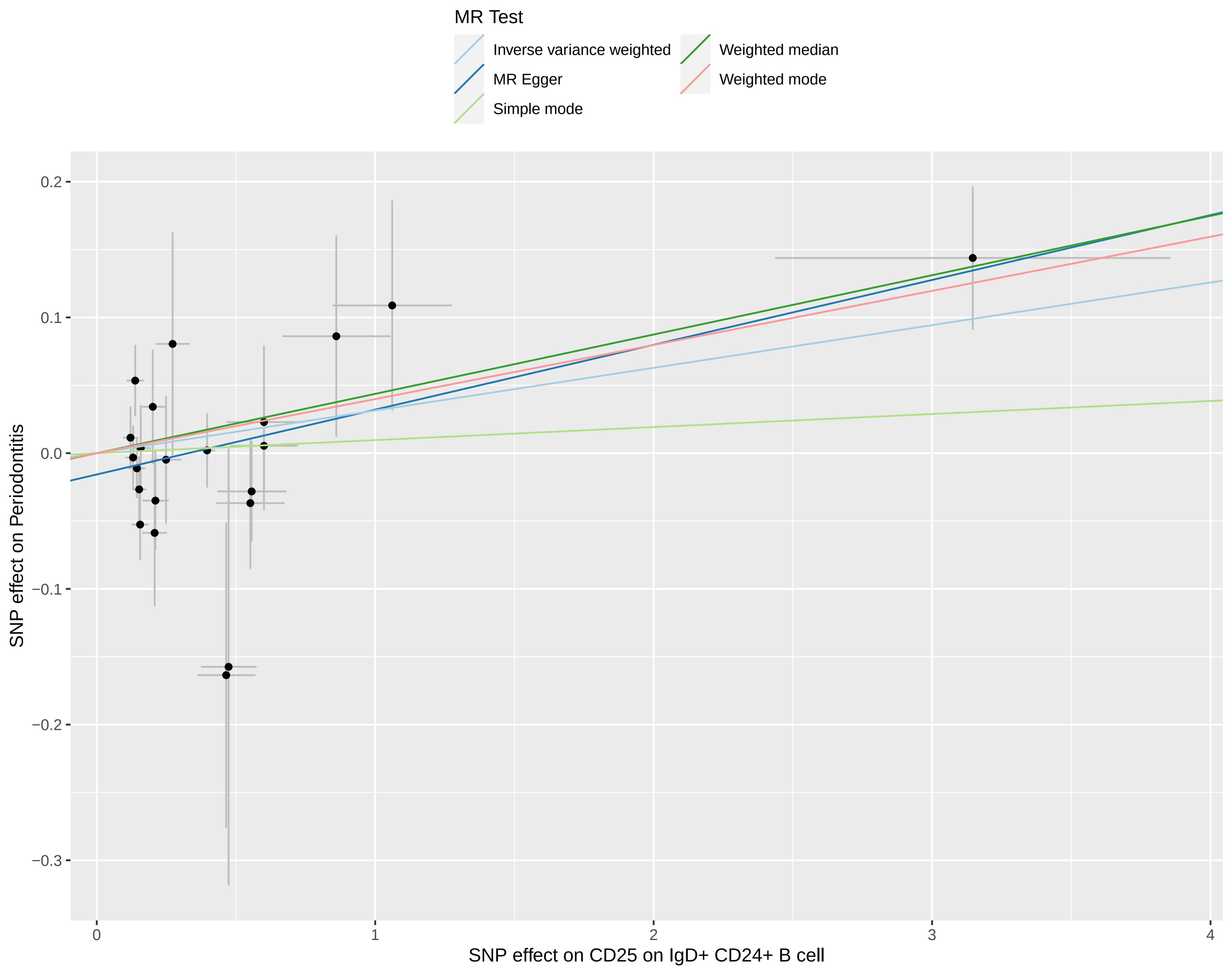
MR Test
Inverse variance weighted
MR Egger
Simple mode
Weighted median
Weighted mode
SNP effect on Periodontitis
SNP effect on CD25 on IgD+ CD24+ B cell
0.2
0.1
0.0
−0.1
−0.2
−0.3
0
1
2
3
4

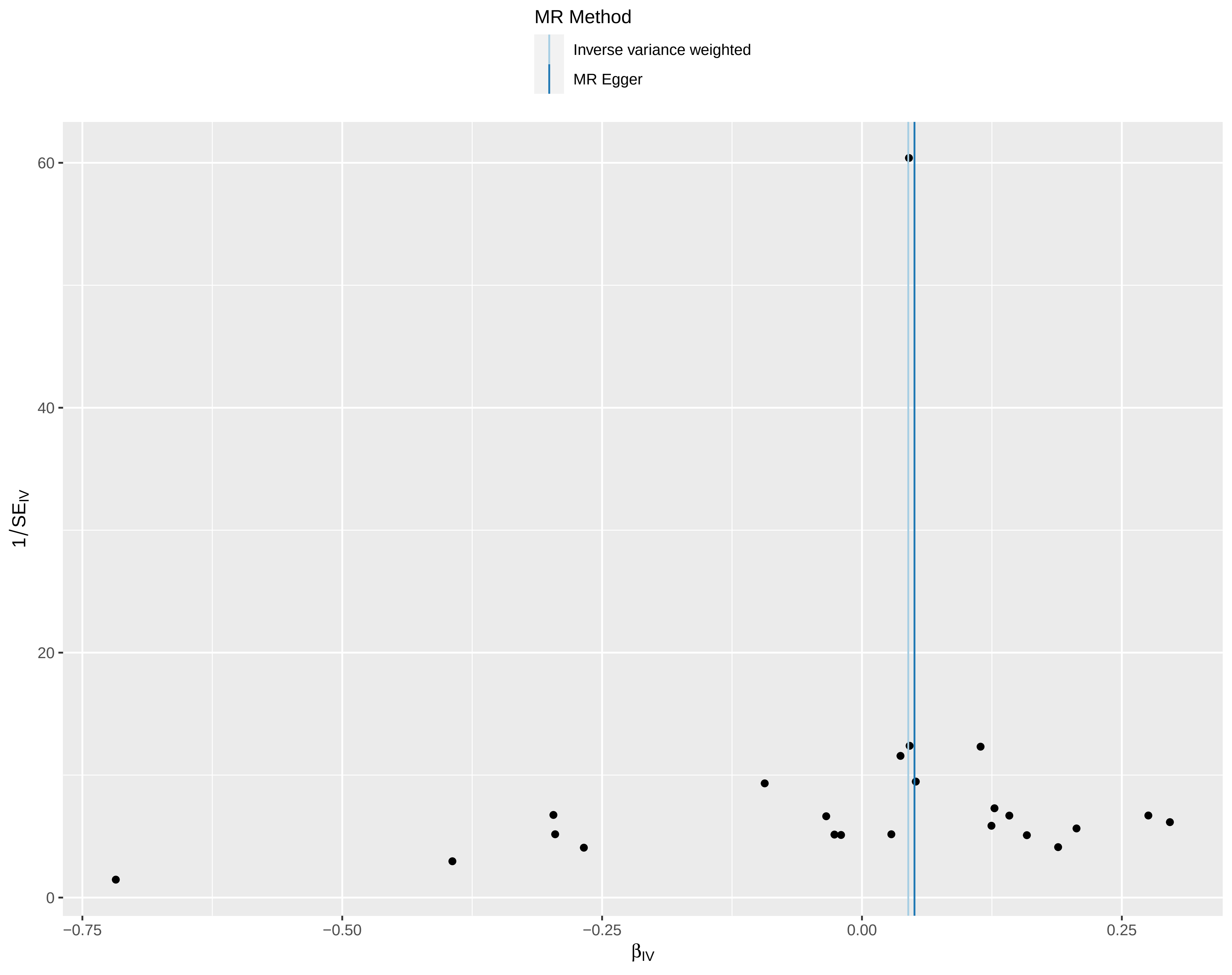
MR Method
Inverse variance weighted
MR Egger
$1/SE_{IV}$
60
40
20
0
−0.75
−0.50
−0.25
0.00
0.25
β_{IV}

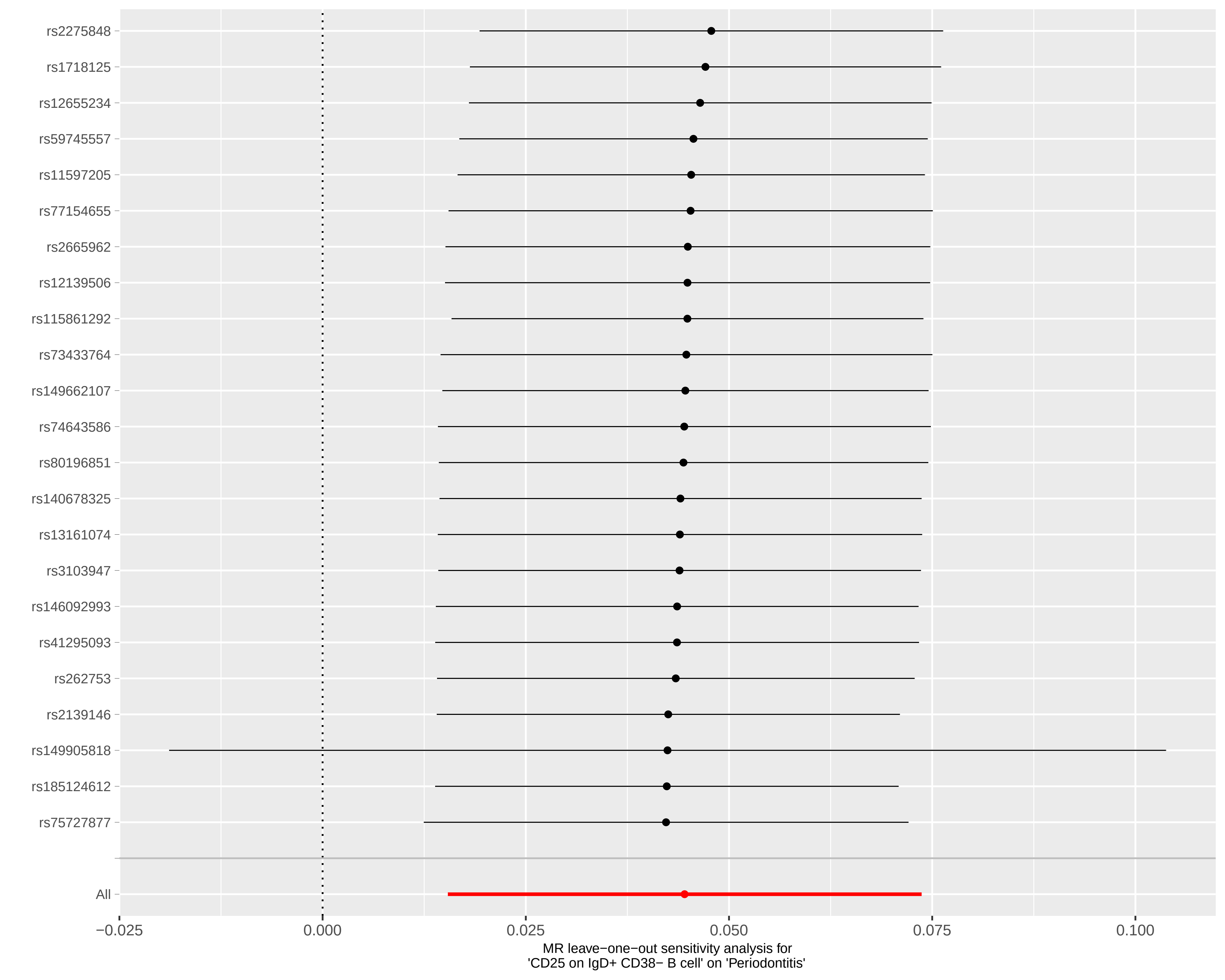

rs2275848
rs1718125
rs12655234
rs59745557
rs11597205
rs77154655
rs2665962
rs12139506
rs115861292
rs73433764
rs149662107
rs74643586
rs80196851
rs140678325
rs13161074
rs3103947
rs146092993
rs41295093
rs262753
rs2139146
rs149905818
rs185124612
rs75727877
All
−0.025
0.000
0.025
0.050
0.075
0.100
MR leave−one−out sensitivity analysis for
'CD25 on IgD+ CD38− B cell' on 'Periodontitis'

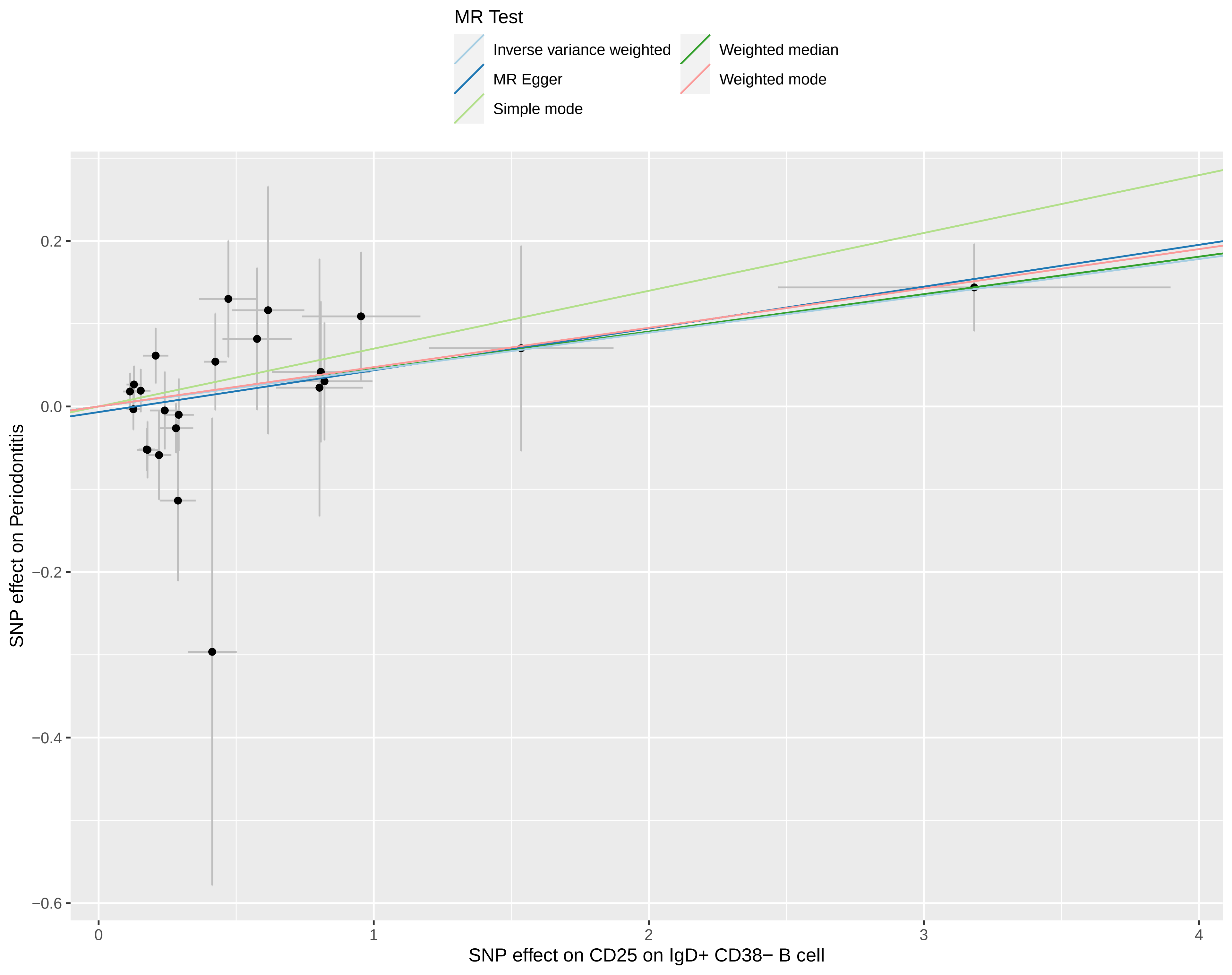

MR Test
Inverse variance weighted
MR Egger
Simple mode
Weighted median
Weighted mode
0.2
0.0
−0.2
−0.4
−0.6
SNP effect on Periodontitis
0
1
2
3
4
SNP effect on CD25 on IgD+ CD38− B cell

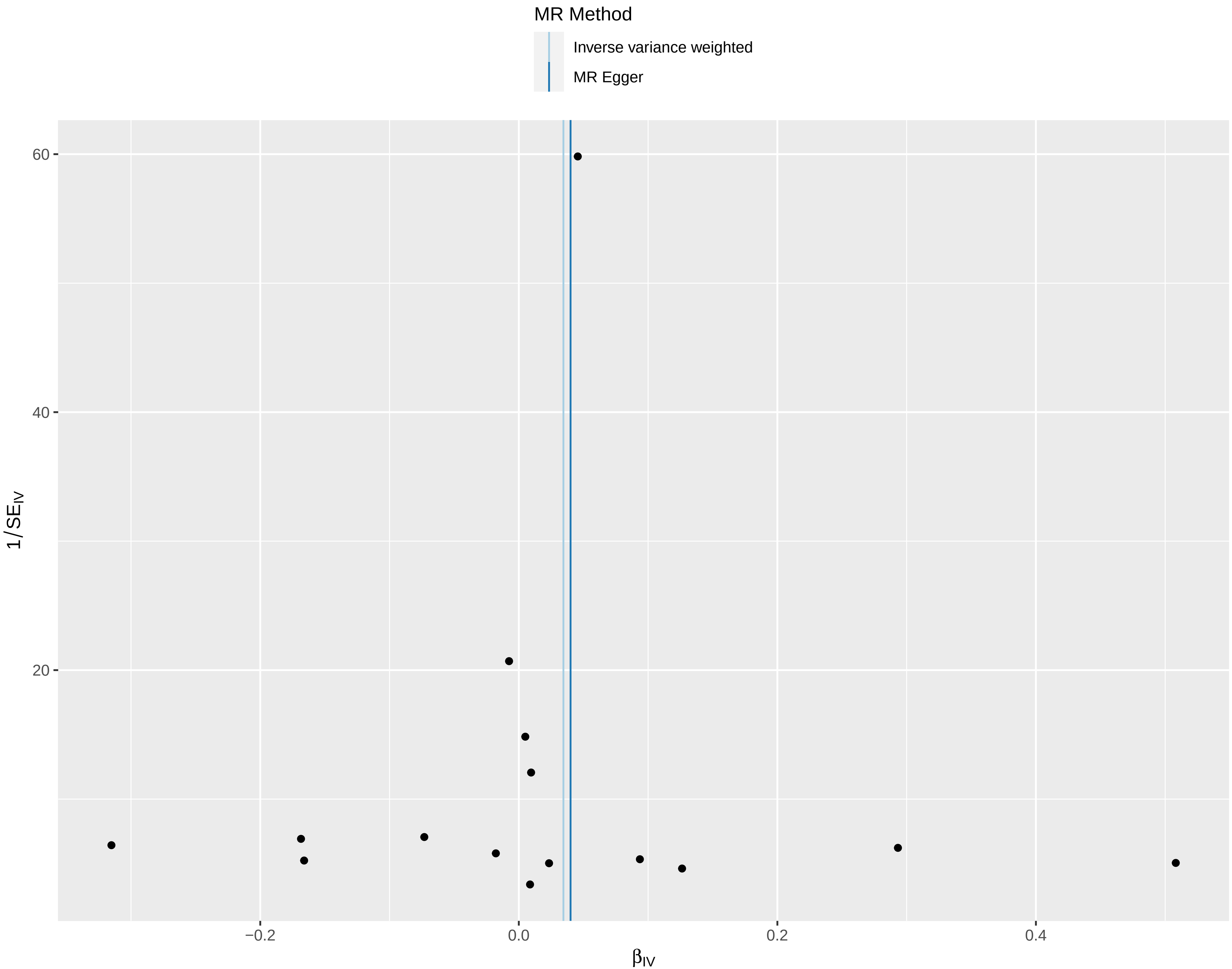
MR Method
Inverse variance weighted
MR Egger
60
40
20
$1/SE_{IV}$
−0.2
0.0
0.2
0.4
β_{IV}

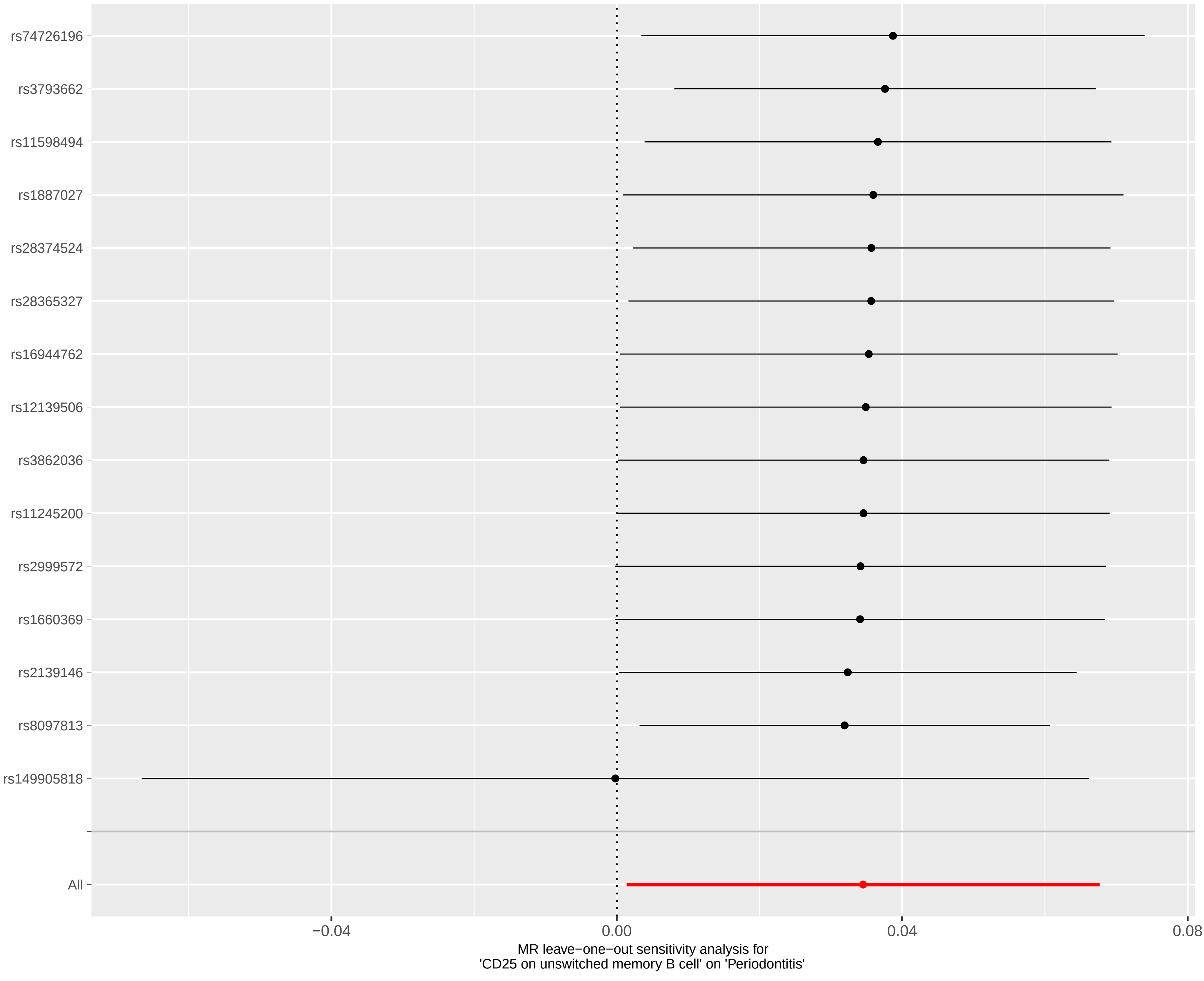
rs74726196
rs3793662
rs11598494
rs1887027
rs28374524
rs28365327
rs16944762
rs12139506
rs3862036
rs11245200
rs2999572
rs1660369
rs2139146
rs8097813
rs149905818
All
−0.04
0.00
0.04
0.08
MR leave−one−out sensitivity analysis for
'CD25 on unswitched memory B cell' on 'Periodontitis'

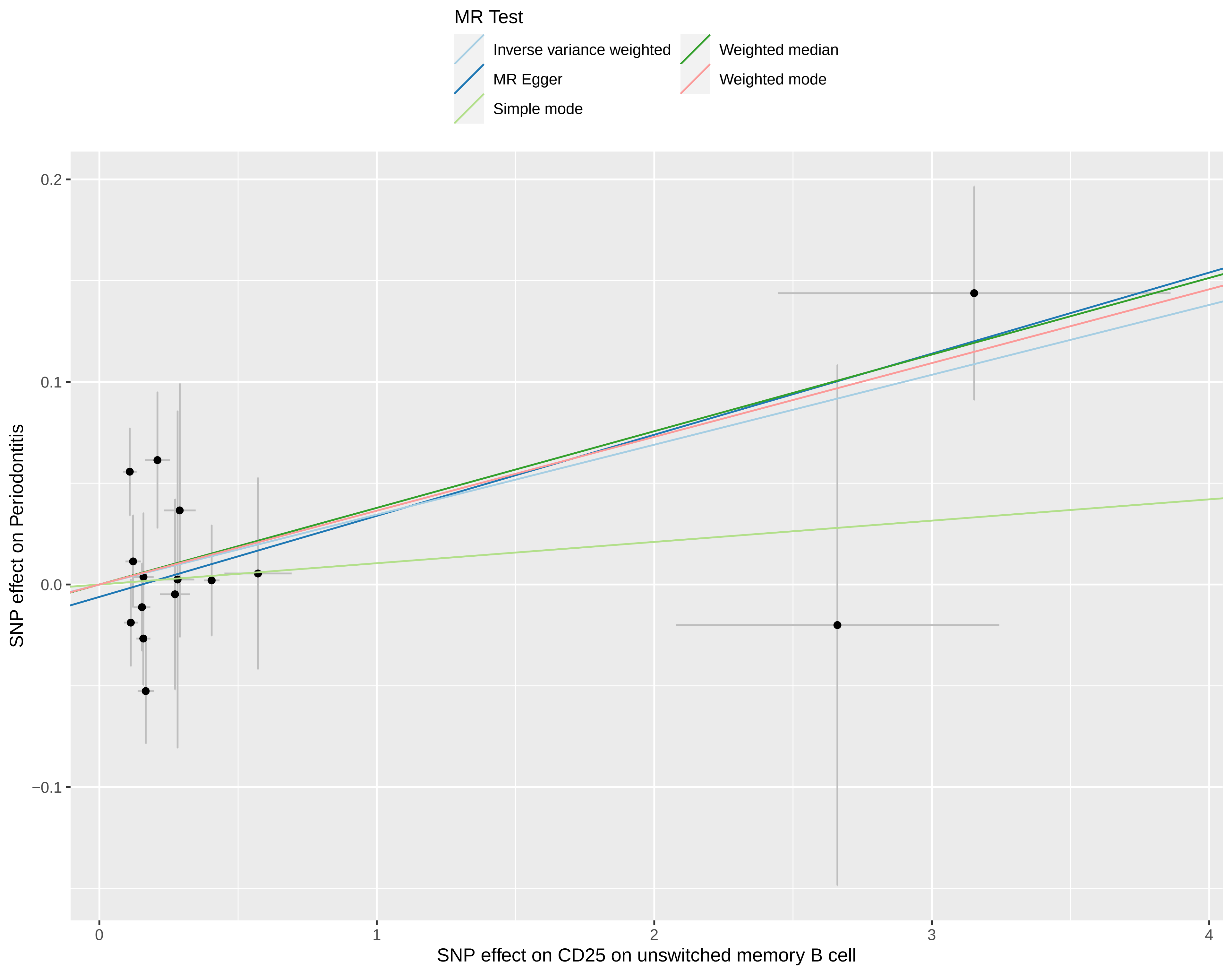
MR Test
Inverse variance weighted
MR Egger
Simple mode
Weighted median
Weighted mode
0.2
0.1
0.0
−0.1
0
1
2
3
4
SNP effect on Periodontitis
SNP effect on CD25 on unswitched memory B cell

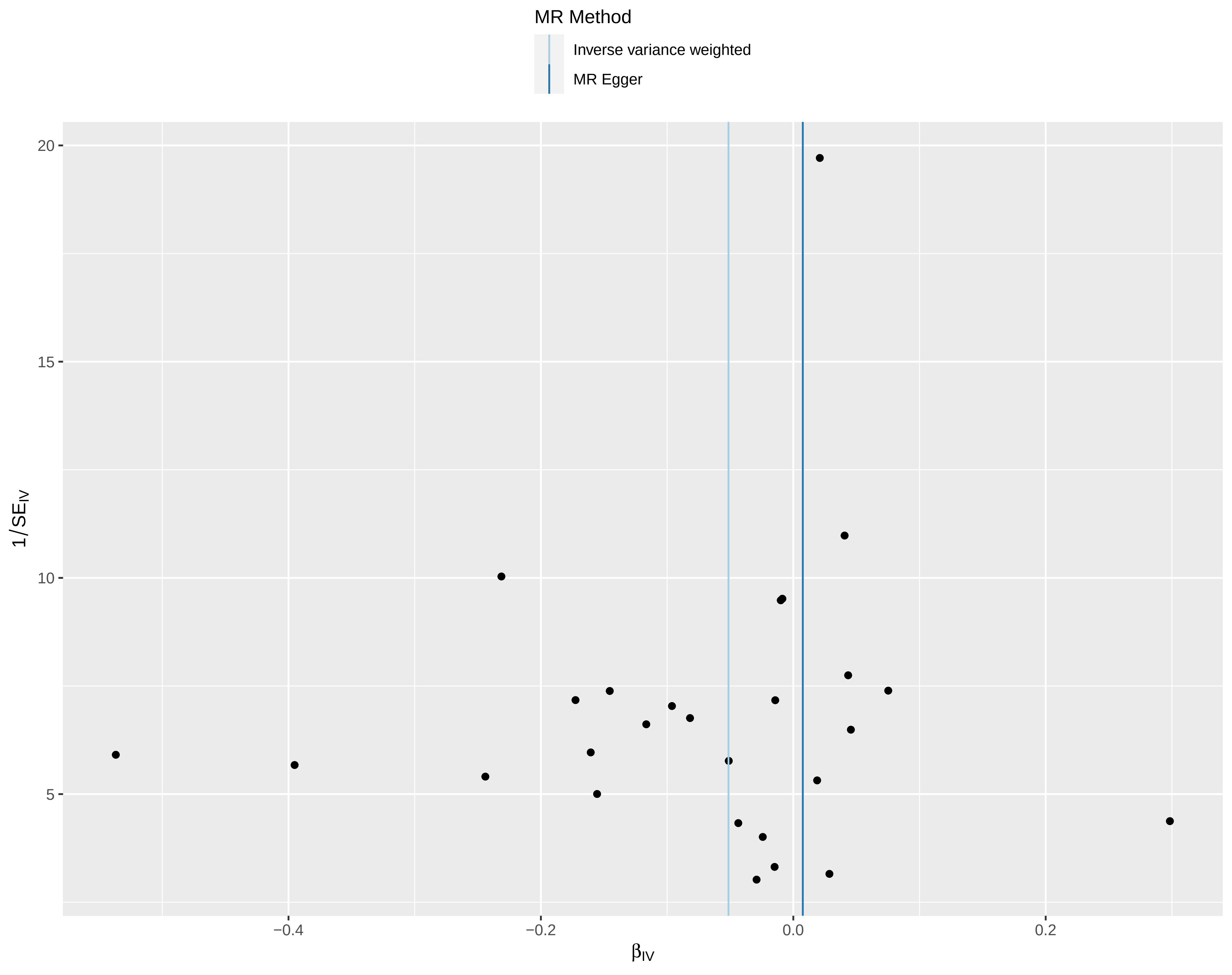
MR Method
Inverse variance weighted
MR Egger
20
15
10
5
1/SE_IV
−0.4
−0.2
0.0
0.2
β_IV

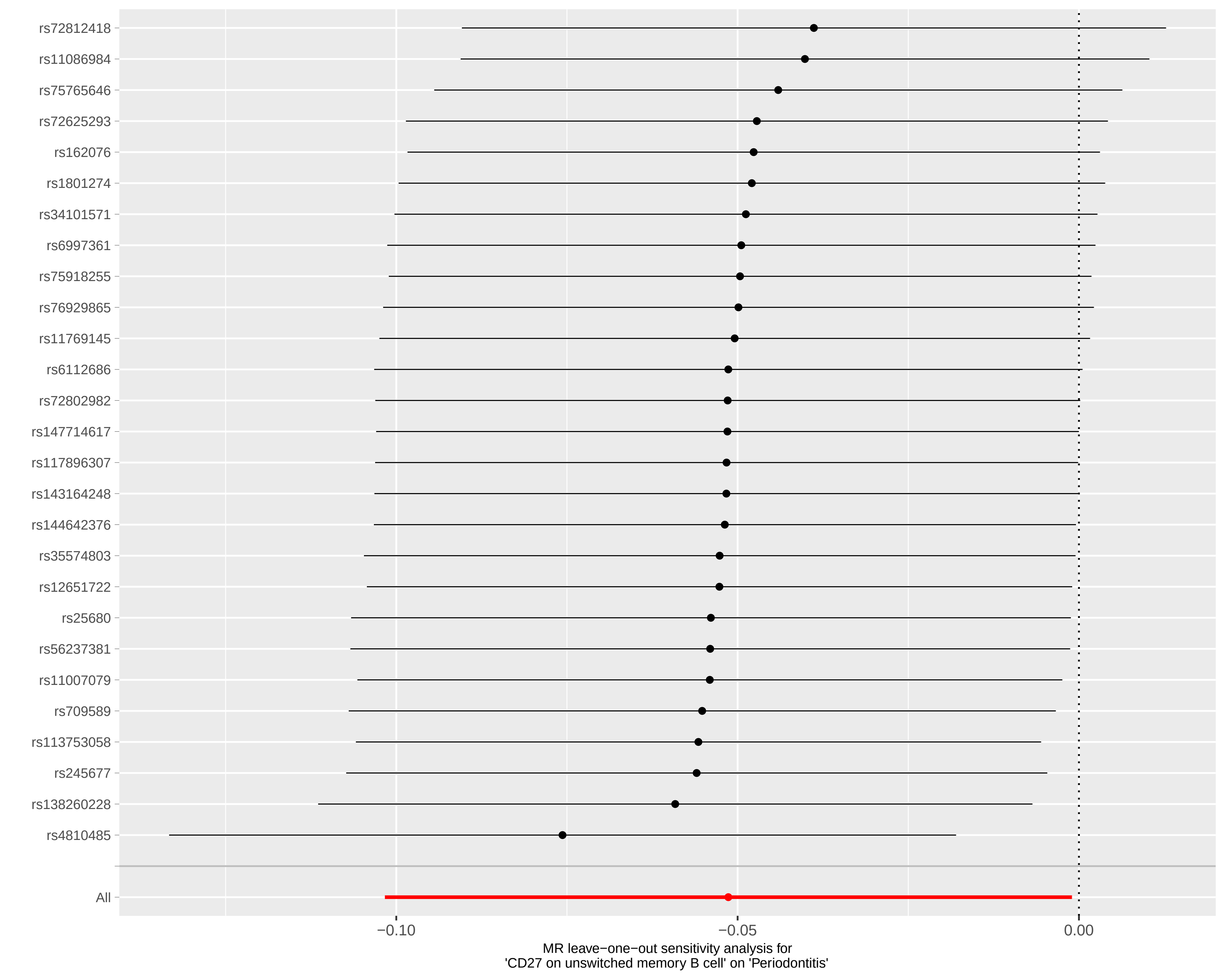
rs72812418
rs11086984
rs75765646
rs72625293
rs162076
rs1801274
rs34101571
rs6997361
rs75918255
rs76929865
rs11769145
rs6112686
rs72802982
rs147714617
rs117896307
rs143164248
rs144642376
rs35574803
rs12651722
rs25680
rs56237381
rs11007079
rs709589
rs113753058
rs245677
rs138260228
rs4810485
All
−0.10
−0.05
0.00
MR leave−one−out sensitivity analysis for
'CD27 on unswitched memory B cell' on 'Periodontitis'

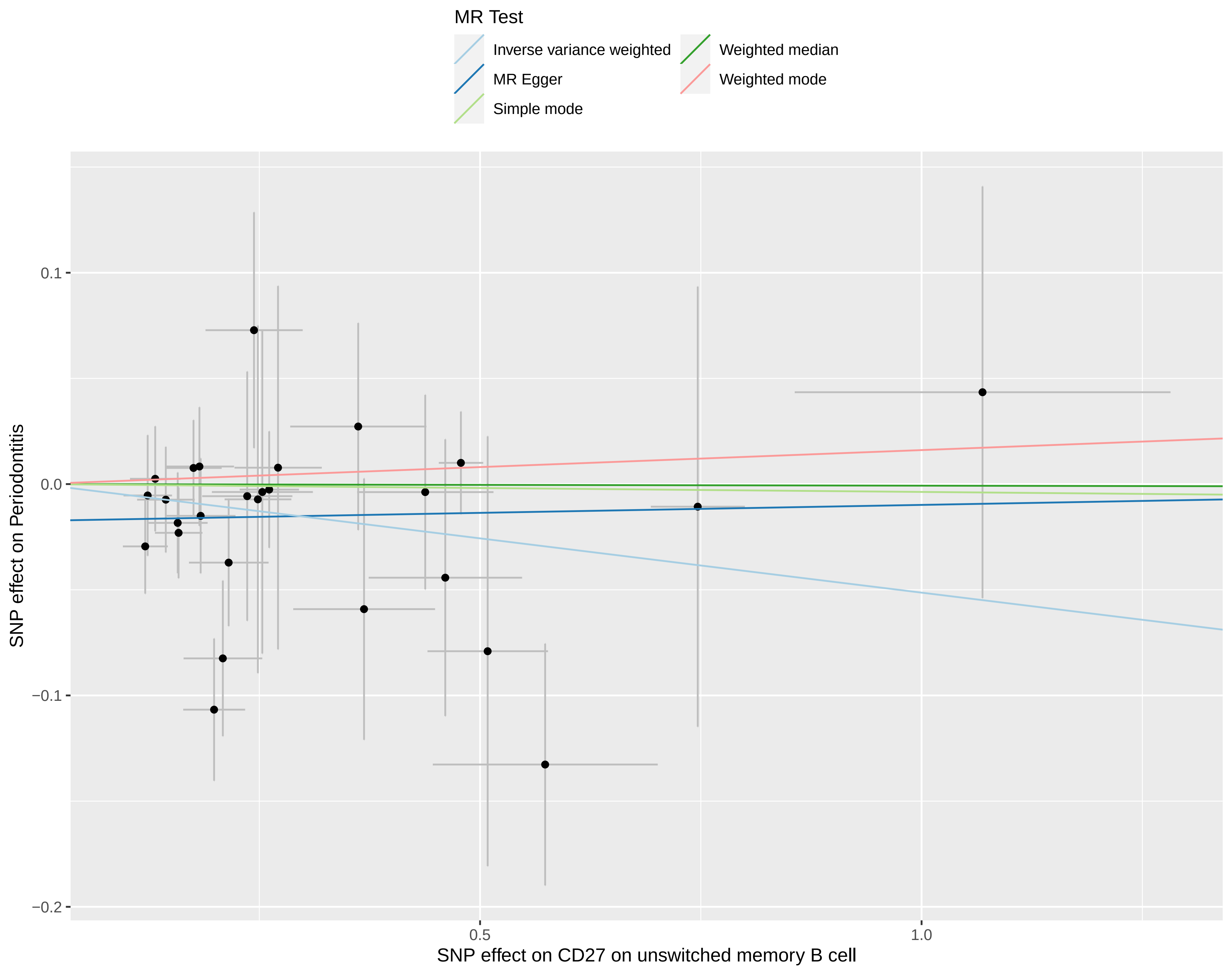
MR Test
Inverse variance weighted
MR Egger
Simple mode
Weighted median
Weighted mode
0.1
0.0
−0.1
−0.2
SNP effect on Periodontitis
0.5
1.0
SNP effect on CD27 on unswitched memory B cell

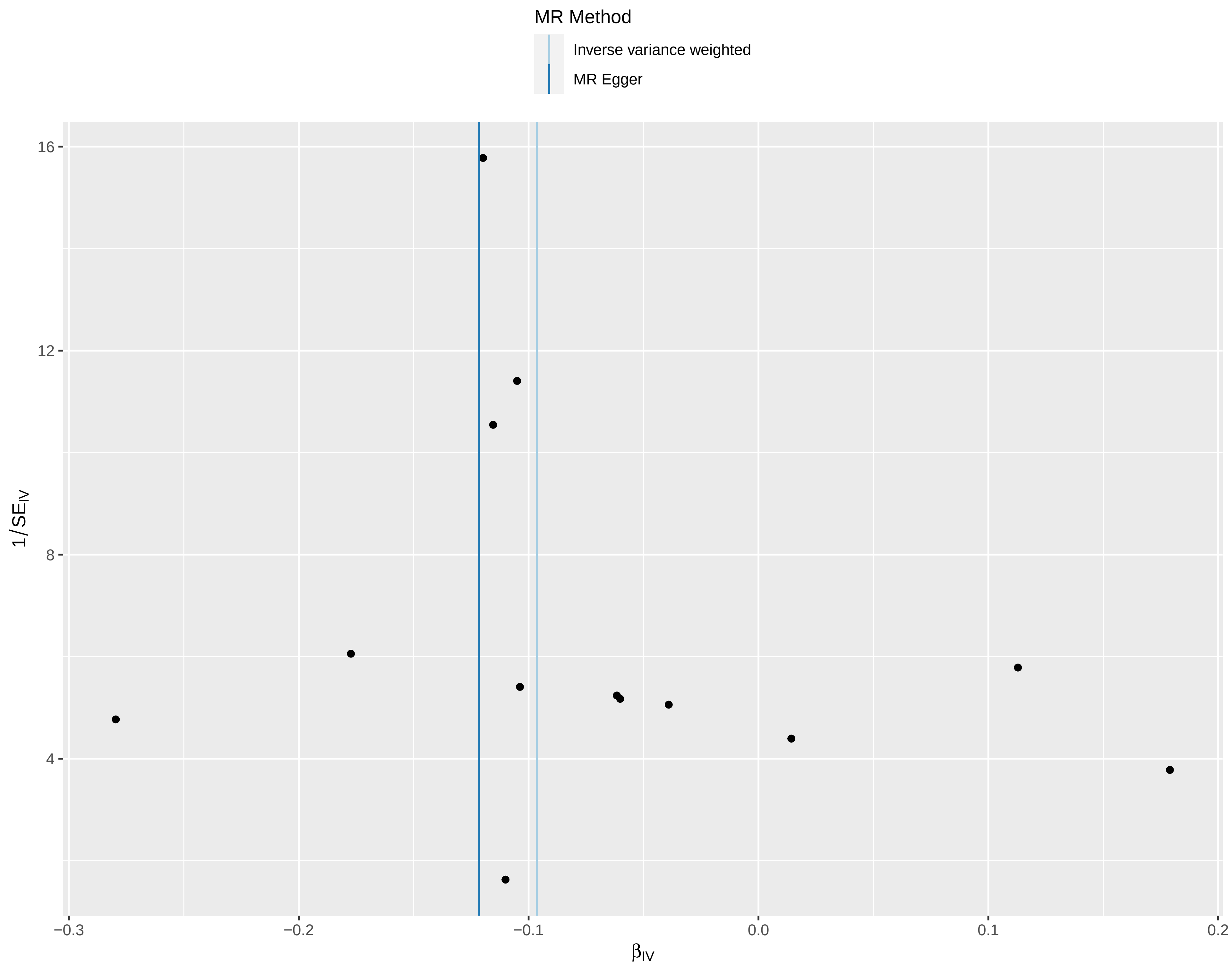

MR Method
Inverse variance weighted
MR Egger
16
12
8
4
1/SE$_{IV}$
−0.3
−0.2
−0.1
0.0
0.1
0.2
β$_{IV}$

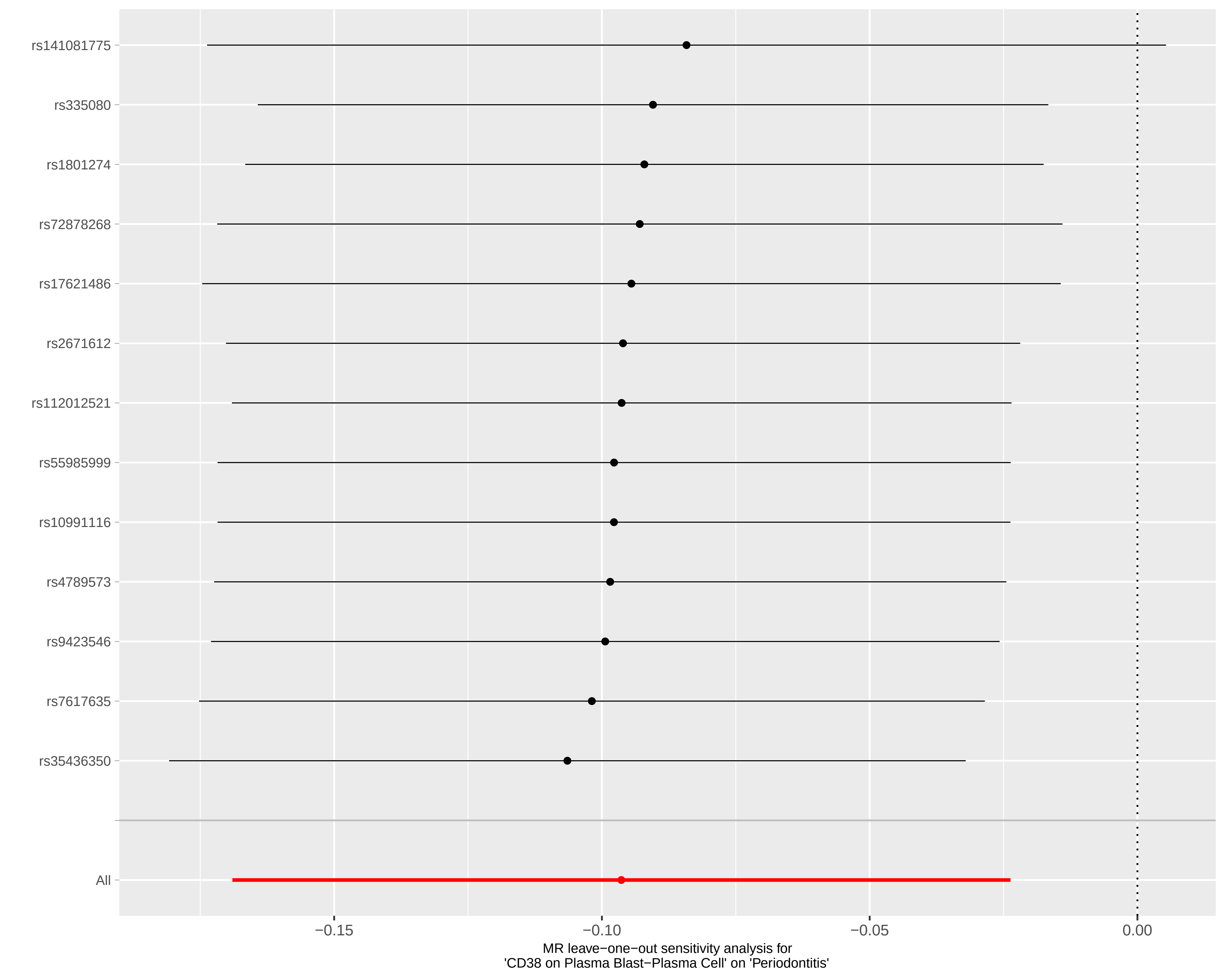
rs141081775
rs335080
rs1801274
rs72878268
rs17621486
rs2671612
rs112012521
rs55985999
rs10991116
rs4789573
rs9423546
rs7617635
rs35436350
All
−0.15
−0.10
−0.05
0.00
MR leave−one−out sensitivity analysis for
'CD38 on Plasma Blast−Plasma Cell' on 'Periodontitis'

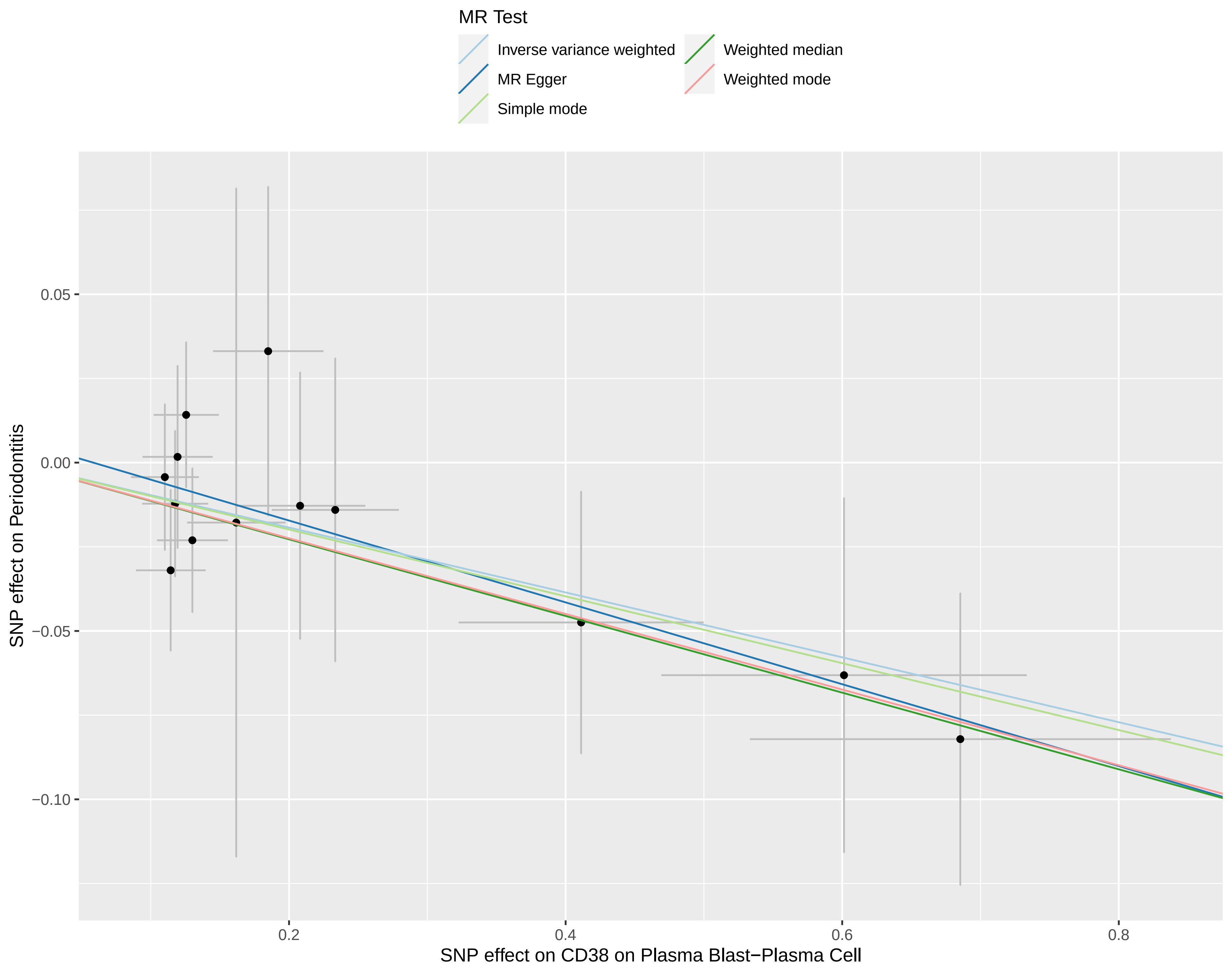
MR Test
Inverse variance weighted
MR Egger
Simple mode
Weighted median
Weighted mode
0.05
0.00
−0.05
−0.10
0.2
0.4
0.6
0.8
SNP effect on Periodontitis
SNP effect on CD38 on Plasma Blast−Plasma Cell

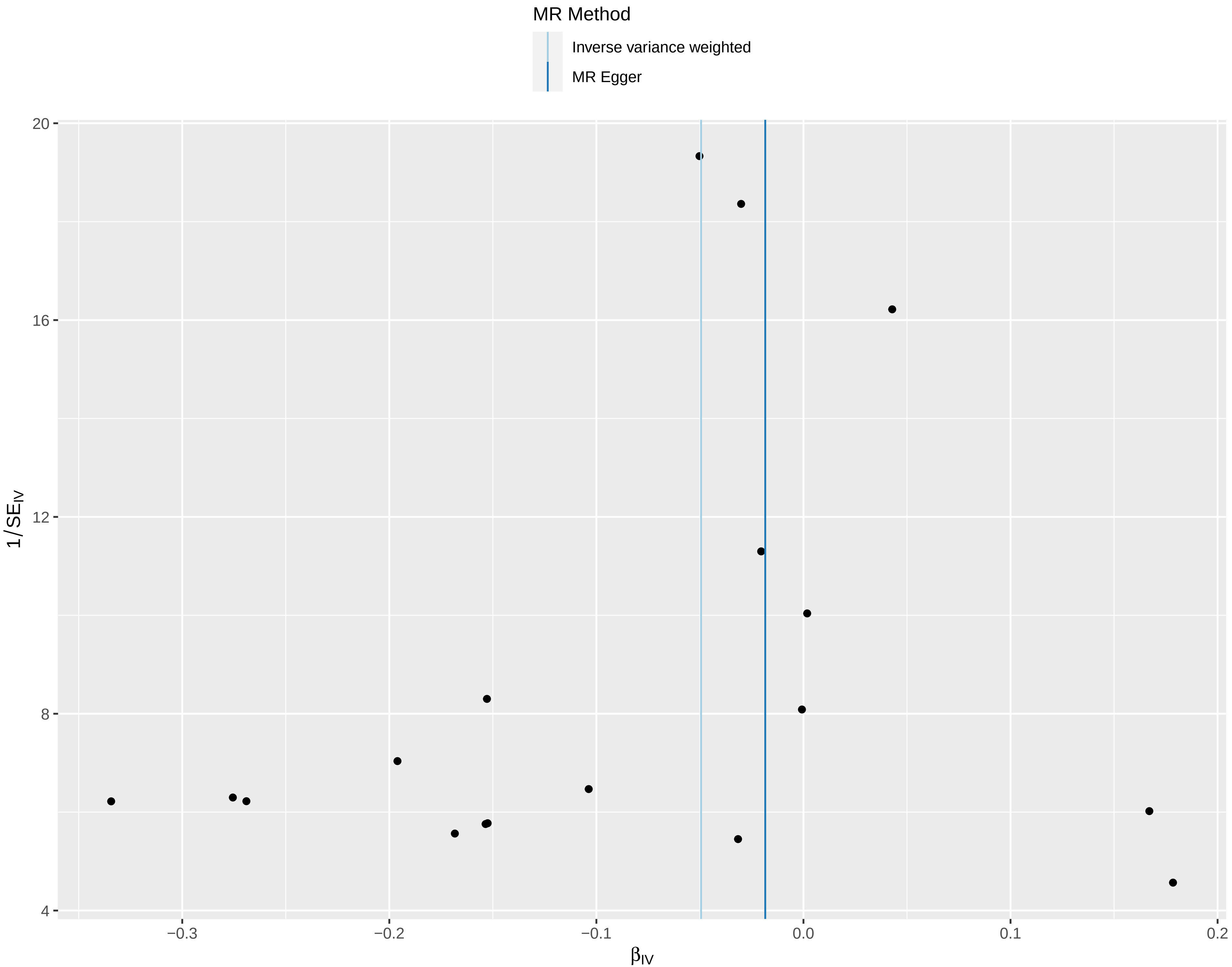
MR Method
Inverse variance weighted
MR Egger
20
16
12
8
4
1/SE$_{IV}$
−0.3
−0.2
−0.1
0.0
0.1
0.2
β$_{IV}$

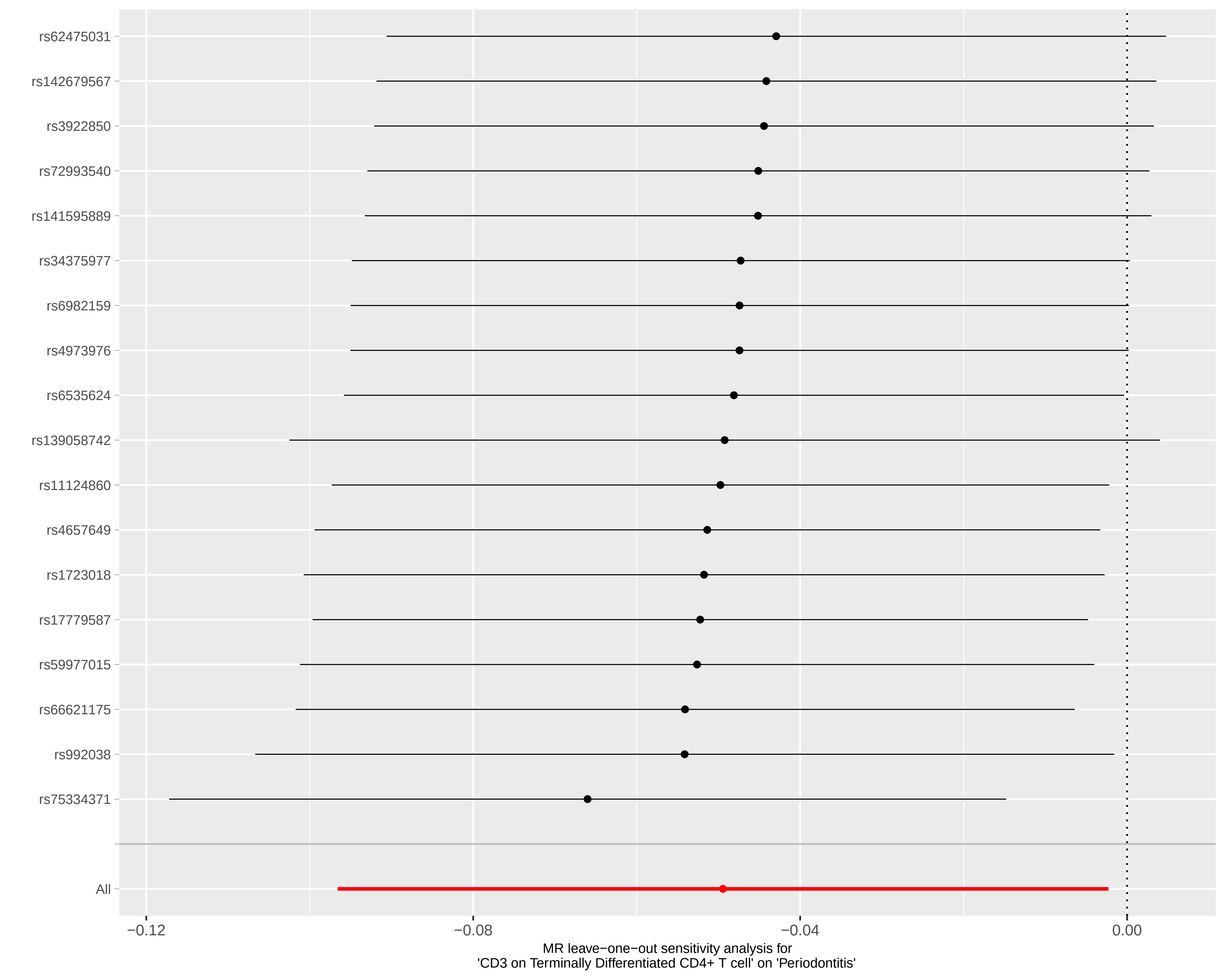
rs62475031
rs142679567
rs3922850
rs72993540
rs141595889
rs34375977
rs6982159
rs4973976
rs6535624
rs139058742
rs11124860
rs4657649
rs1723018
rs17779587
rs59977015
rs66621175
rs992038
rs75334371
All
−0.12
−0.08
−0.04
0.00
MR leave−one−out sensitivity analysis for
'CD3 on Terminally Differentiated CD4+ T cell' on 'Periodontitis'

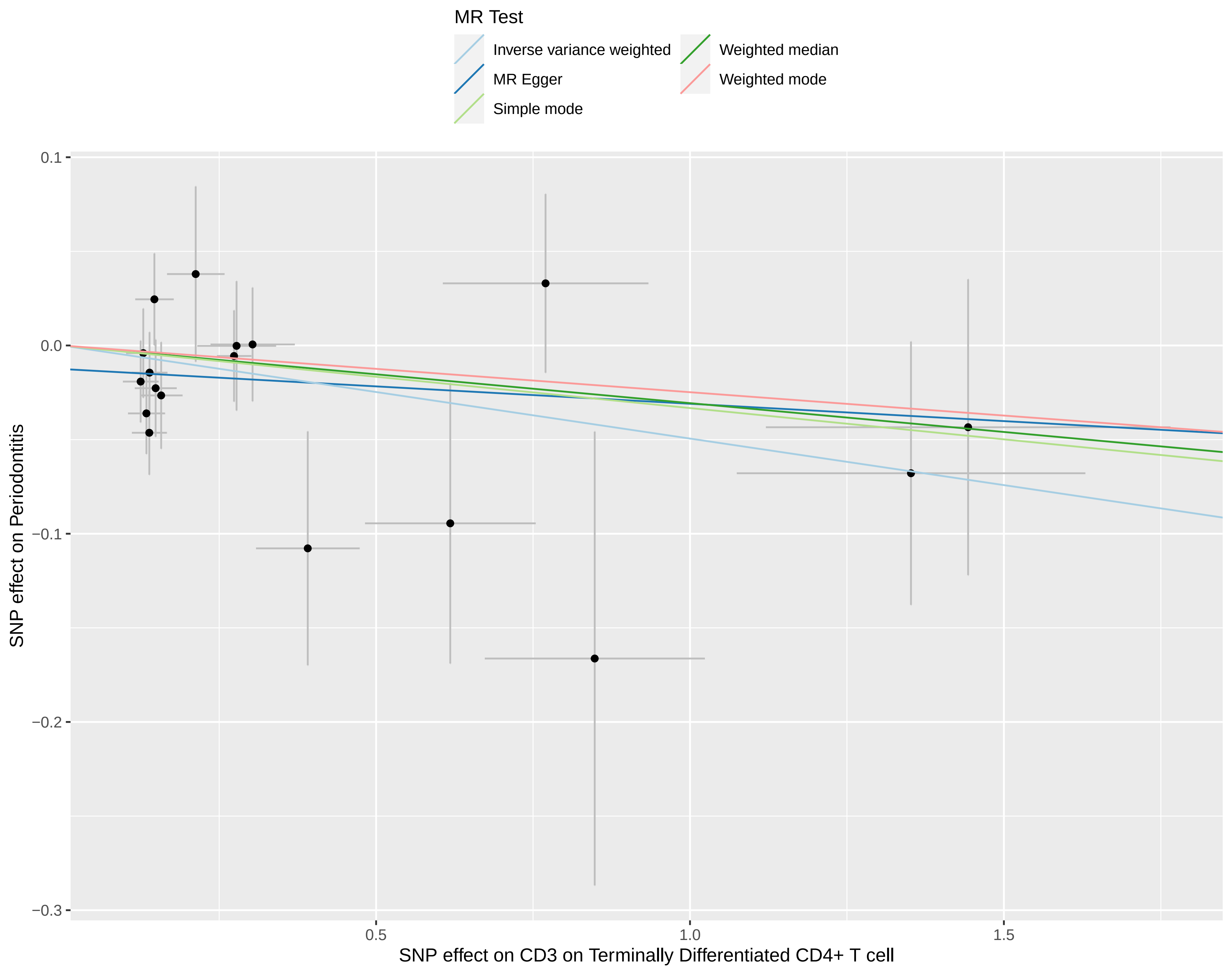

MR Test
Inverse variance weighted
MR Egger
Simple mode
Weighted median
Weighted mode
0.1
0.0
−0.1
−0.2
−0.3
0.5
1.0
1.5
SNP effect on Periodontitis
SNP effect on CD3 on Terminally Differentiated CD4+ T cell

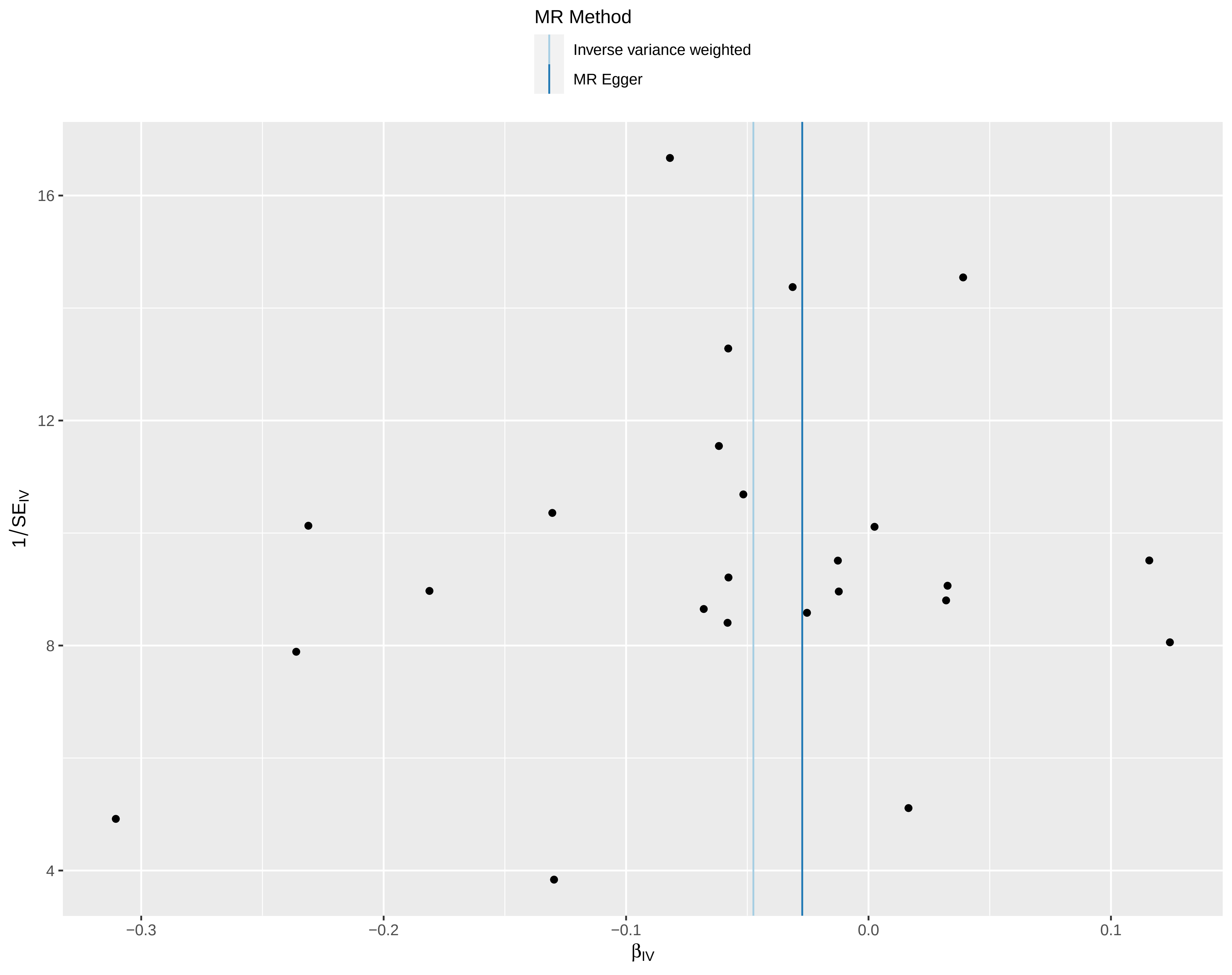
MR Method
Inverse variance weighted
MR Egger
16
12
8
4
−0.3
−0.2
−0.1
0.0
0.1
$1/SE_{IV}$
β_{IV}

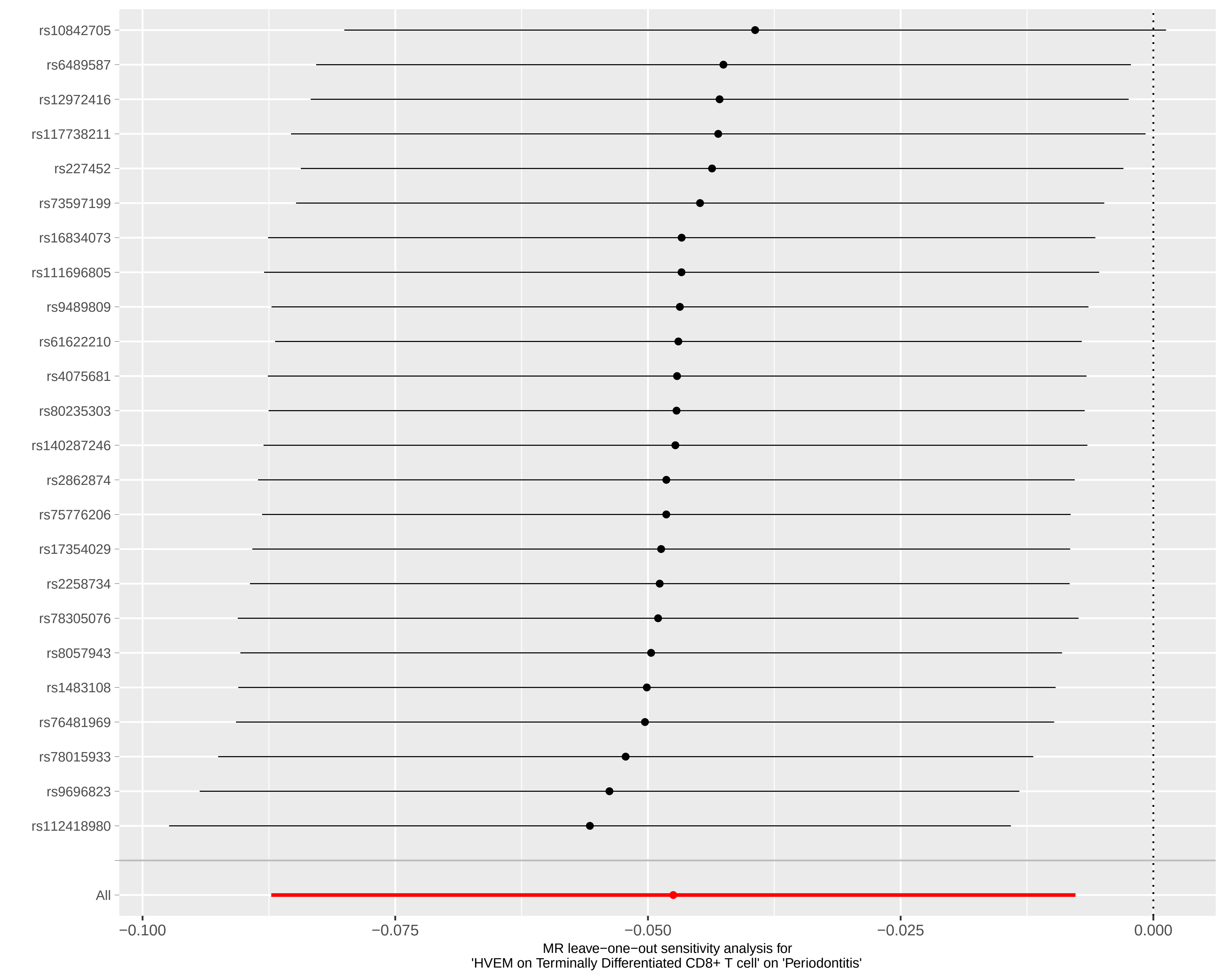

rs10842705
rs6489587
rs12972416
rs117738211
rs227452
rs73597199
rs16834073
rs111696805
rs9489809
rs61622210
rs4075681
rs80235303
rs140287246
rs2862874
rs75776206
rs17354029
rs2258734
rs78305076
rs8057943
rs1483108
rs76481969
rs78015933
rs9696823
rs112418980
All
−0.100
−0.075
−0.050
−0.025
0.000
MR leave−one−out sensitivity analysis for
'HVEM on Terminally Differentiated CD8+ T cell' on 'Periodontitis'

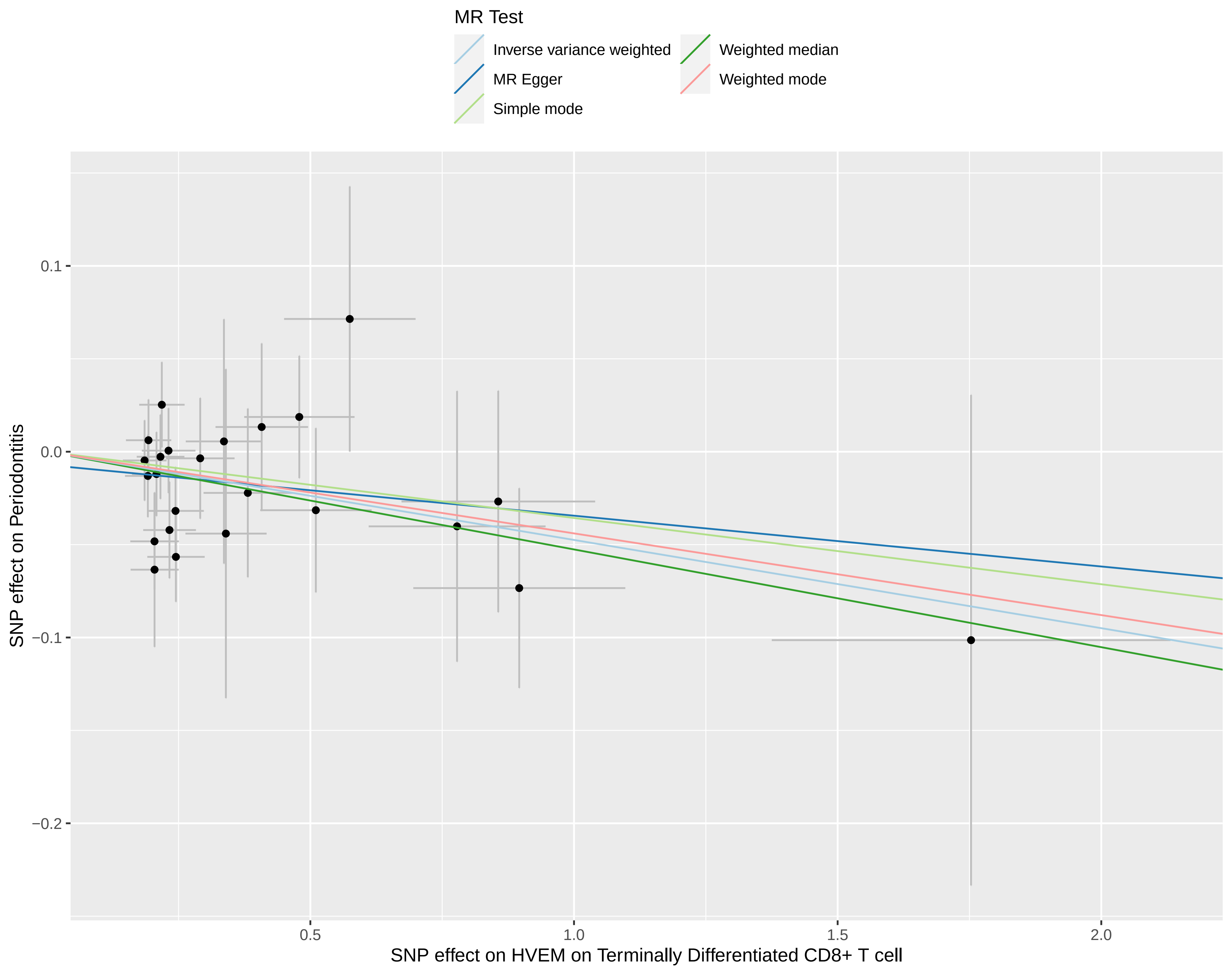
MR Test
Inverse variance weighted
MR Egger
Simple mode
Weighted median
Weighted mode
0.1
0.0
−0.1
−0.2
SNP effect on Periodontitis
0.5
1.0
1.5
2.0
SNP effect on HVEM on Terminally Differentiated CD8+ T cell

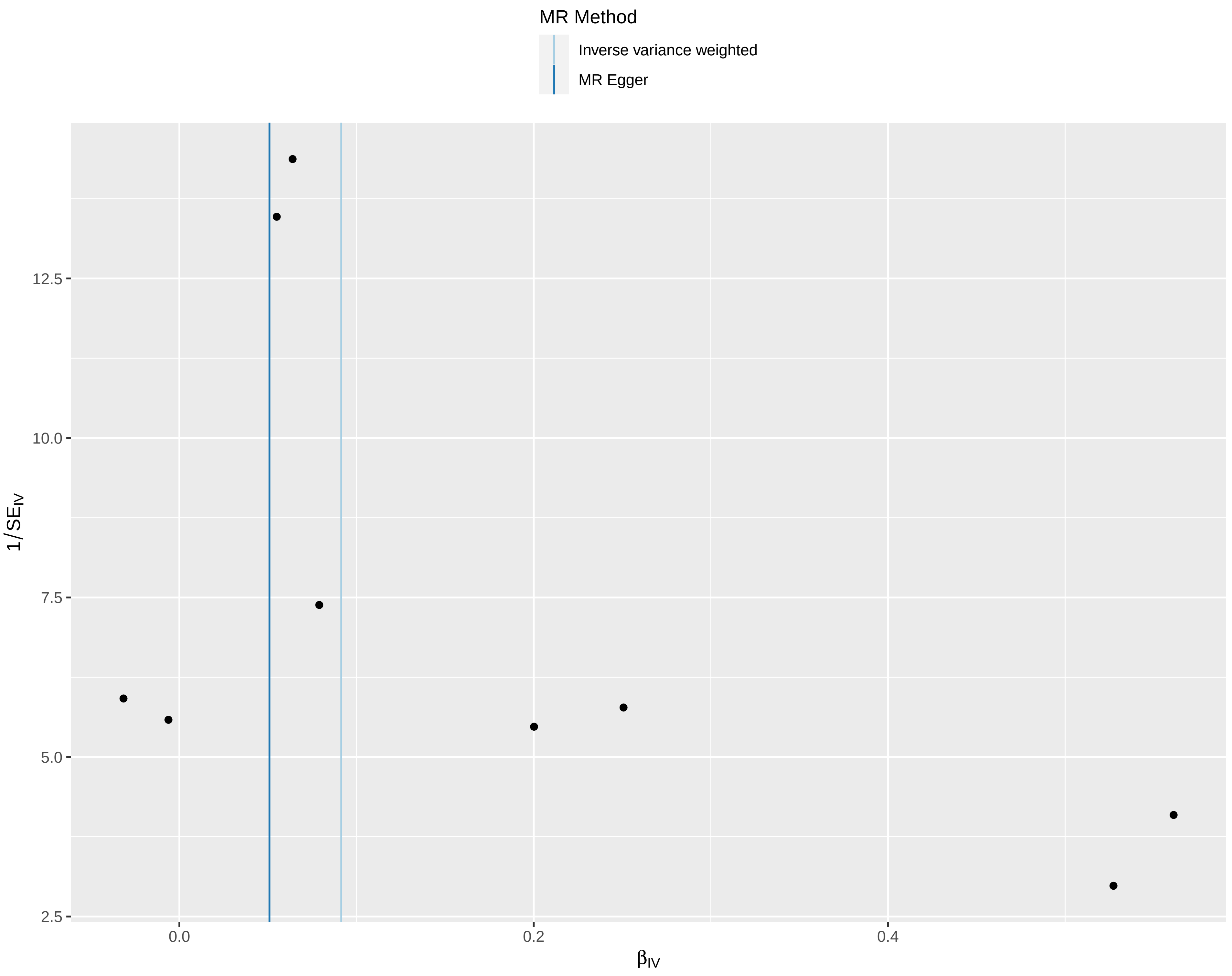
MR Method
Inverse variance weighted
MR Egger
12.5
10.0
7.5
5.0
2.5
1/SE_IV
0.0
0.2
0.4
β_IV

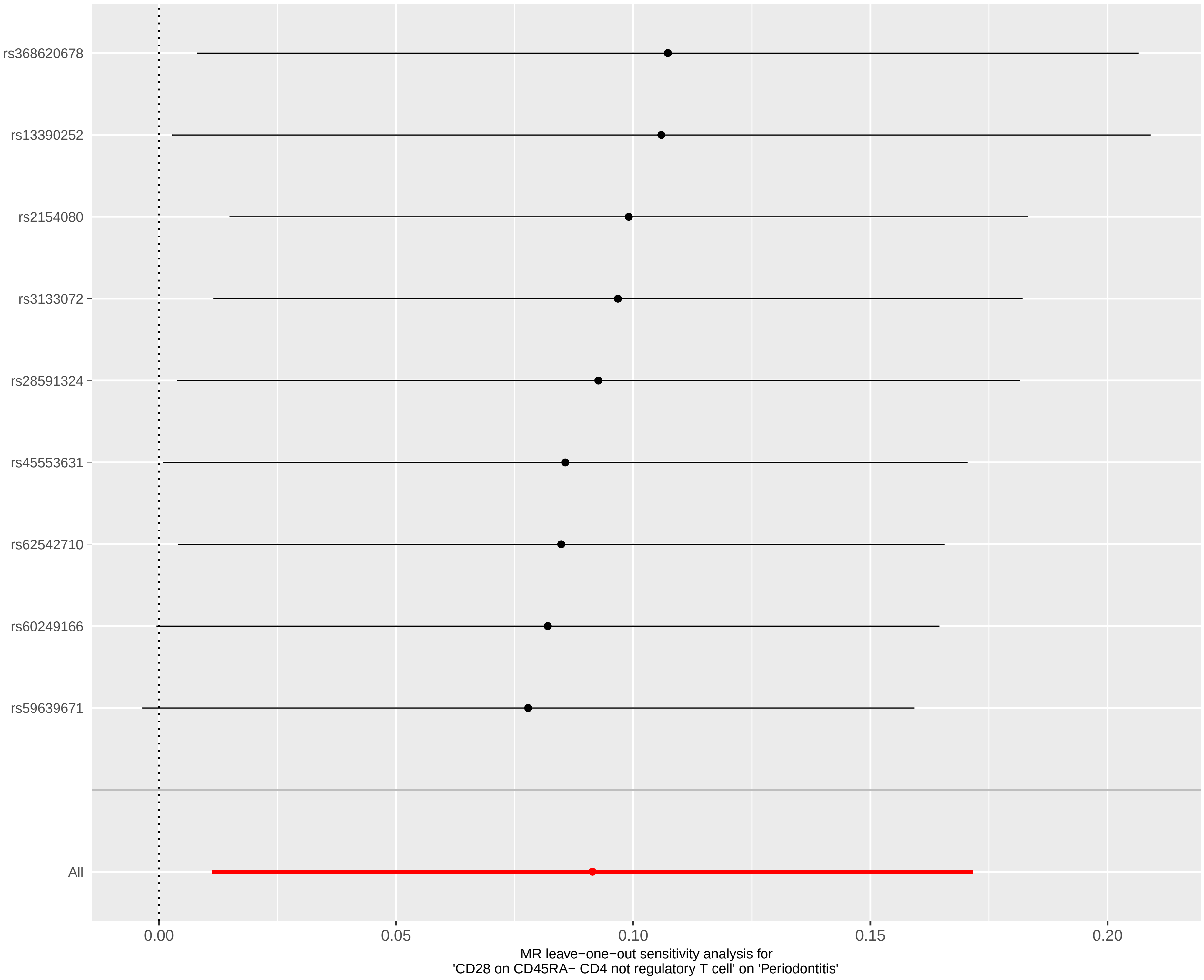
rs368620678
rs13390252
rs2154080
rs3133072
rs28591324
rs45553631
rs62542710
rs60249166
rs59639671
All
0.00
0.05
0.10
0.15
0.20
MR leave−one−out sensitivity analysis for
'CD28 on CD45RA− CD4 not regulatory T cell' on 'Periodontitis'

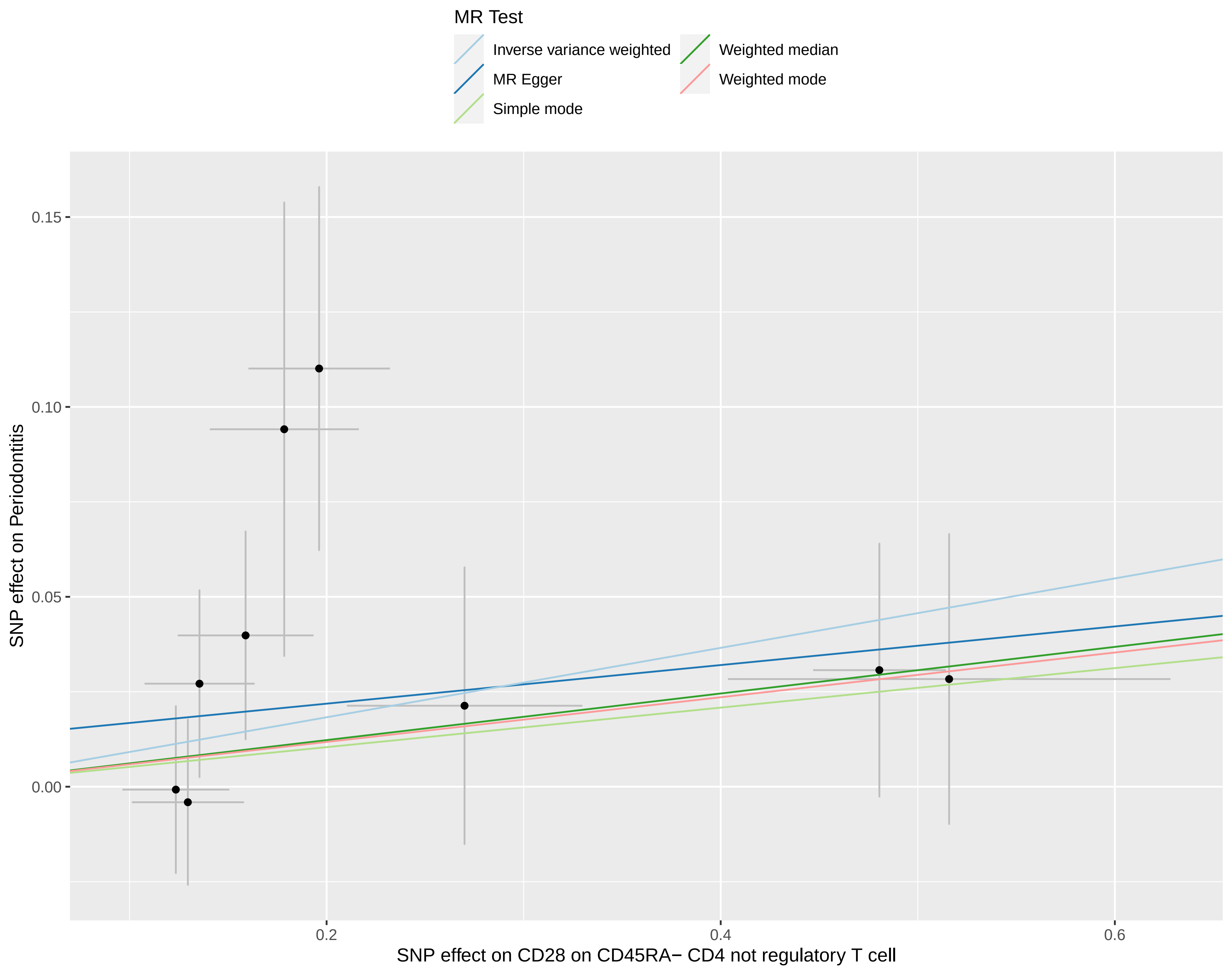

MR Test
Inverse variance weighted
MR Egger
Simple mode
Weighted median
Weighted mode
0.15
0.10
0.05
0.00
0.2
0.4
0.6
SNP effect on Periodontitis
SNP effect on CD28 on CD45RA− CD4 not regulatory T cell

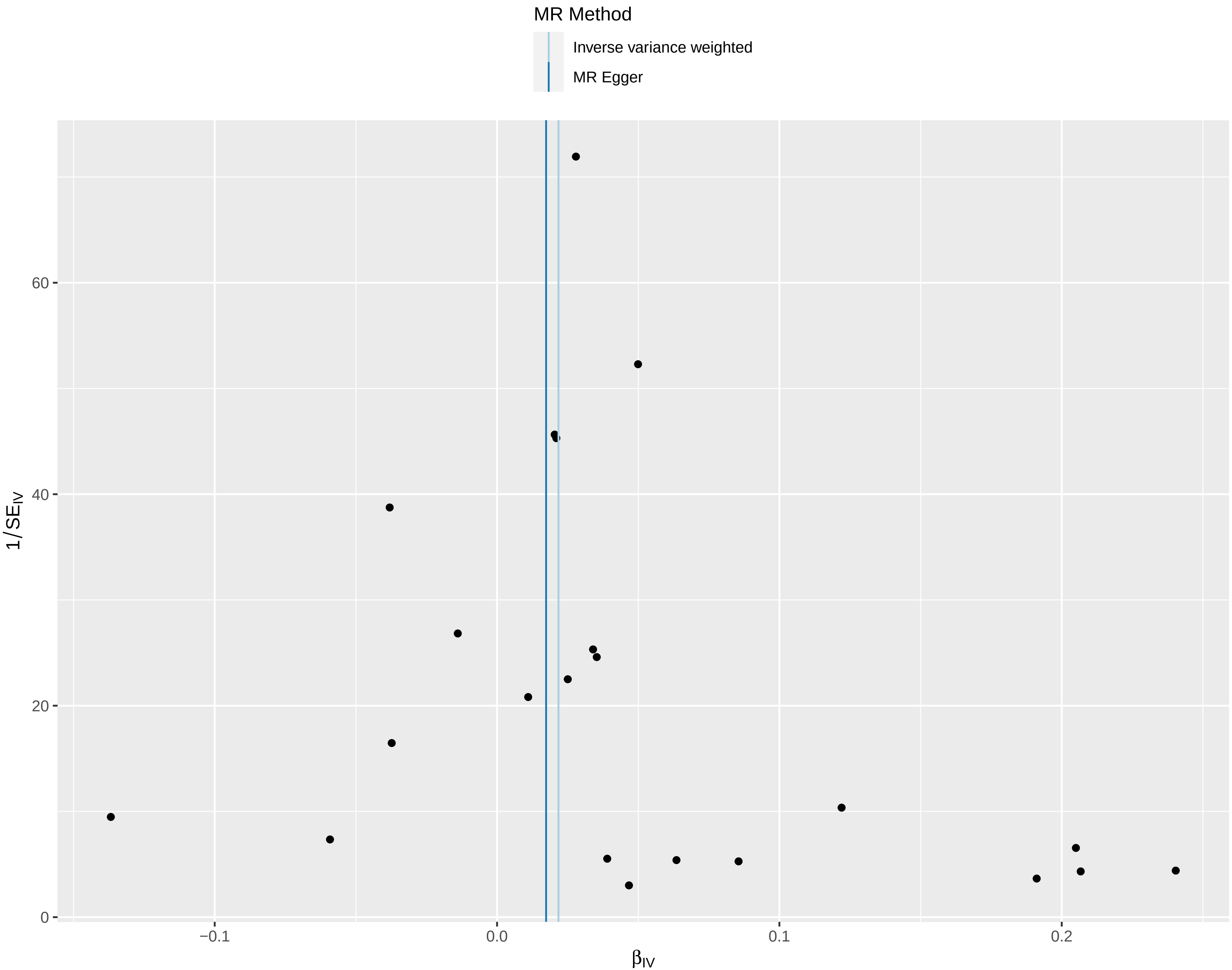

MR Method
Inverse variance weighted
MR Egger
60
40
20
0
1/SE$_{IV}$
−0.1
0.0
0.1
0.2
β$_{IV}$

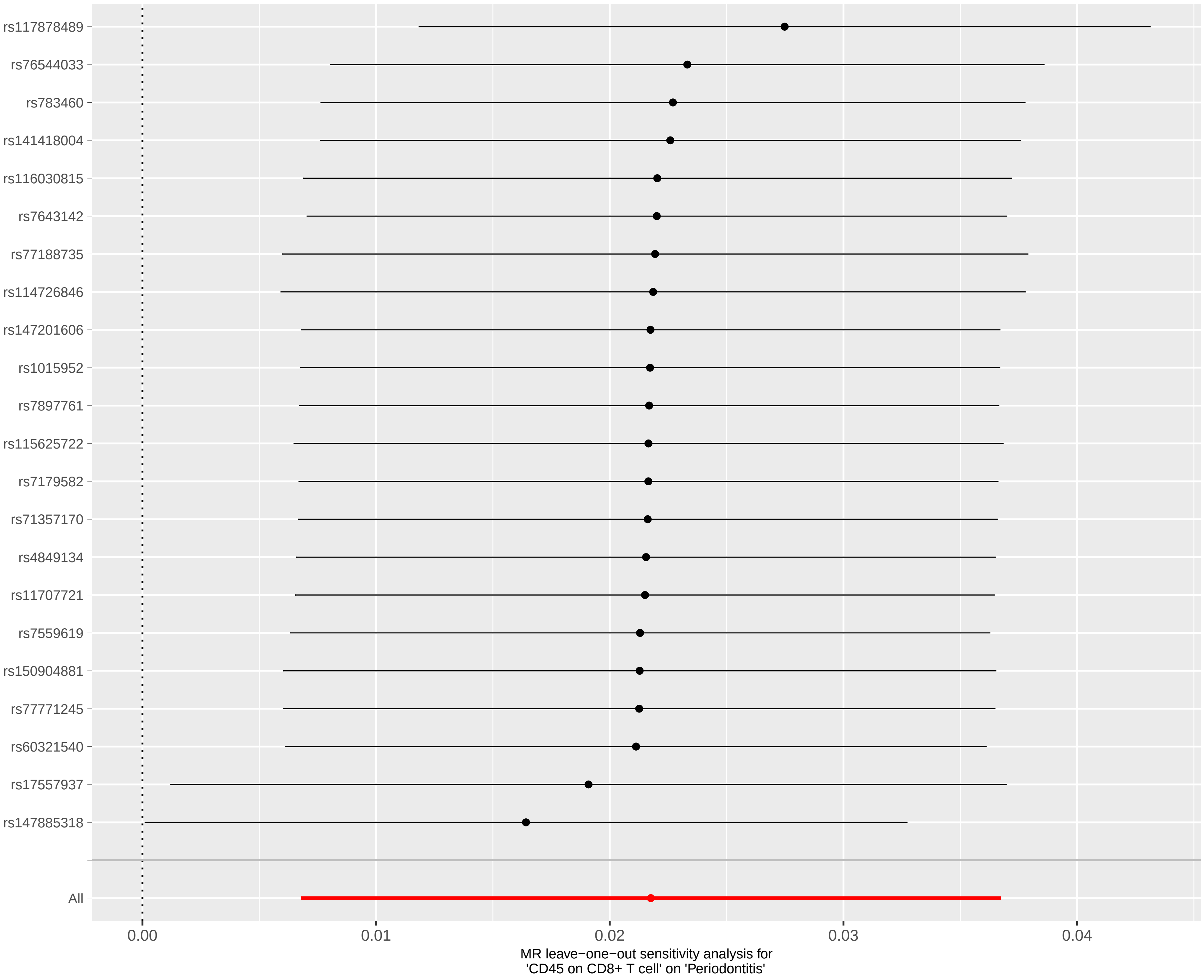
rs117878489
rs76544033
rs783460
rs141418004
rs116030815
rs7643142
rs77188735
rs114726846
rs147201606
rs1015952
rs7897761
rs115625722
rs7179582
rs71357170
rs4849134
rs11707721
rs7559619
rs150904881
rs77771245
rs60321540
rs17557937
rs147885318
All
0.00
0.01
0.02
0.03
0.04
MR leave−one−out sensitivity analysis for
'CD45 on CD8+ T cell' on 'Periodontitis'

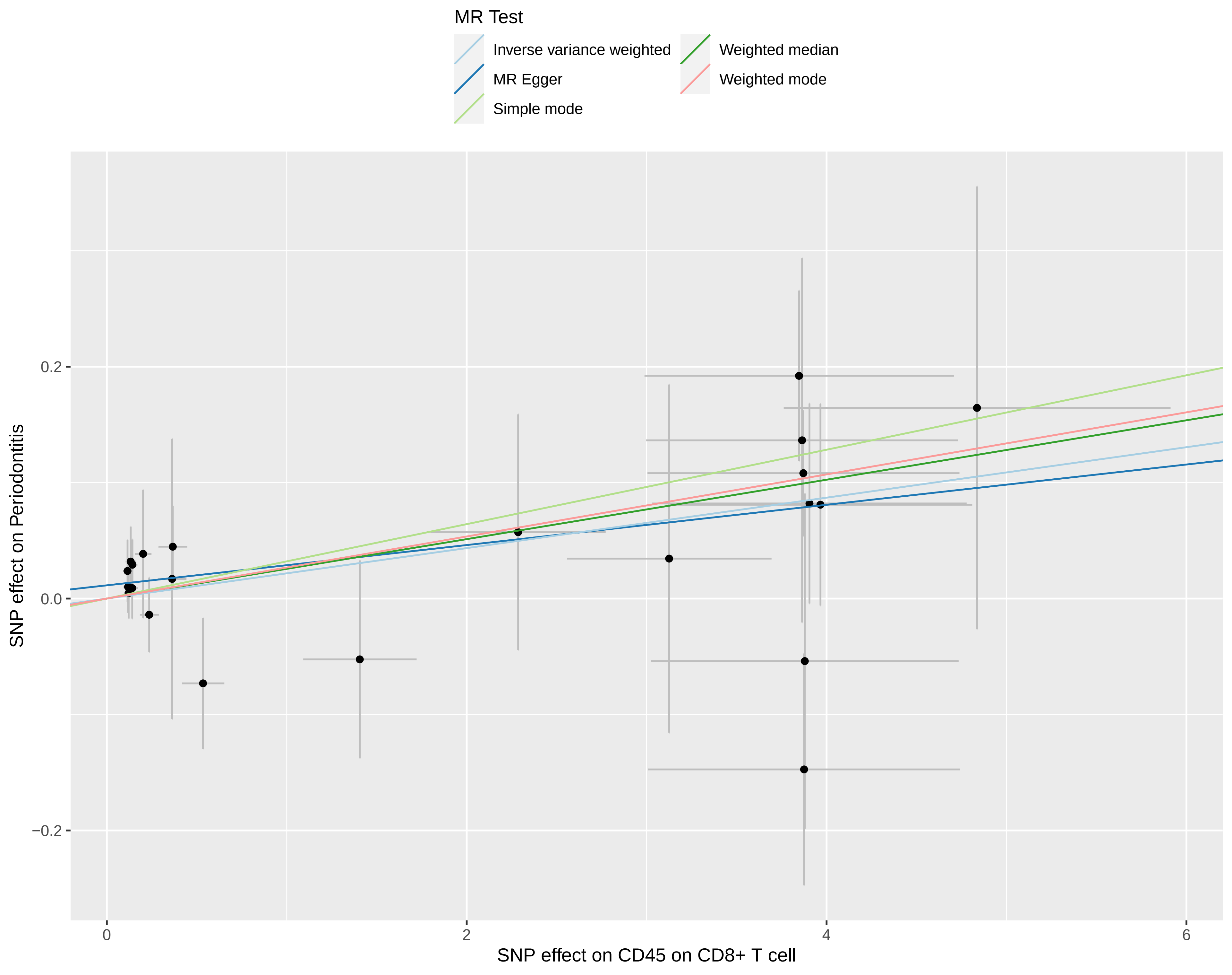
MR Test
Inverse variance weighted
MR Egger
Simple mode
Weighted median
Weighted mode
0.2
0.0
−0.2
0
2
4
6
SNP effect on Periodontitis
SNP effect on CD45 on CD8+ T cell

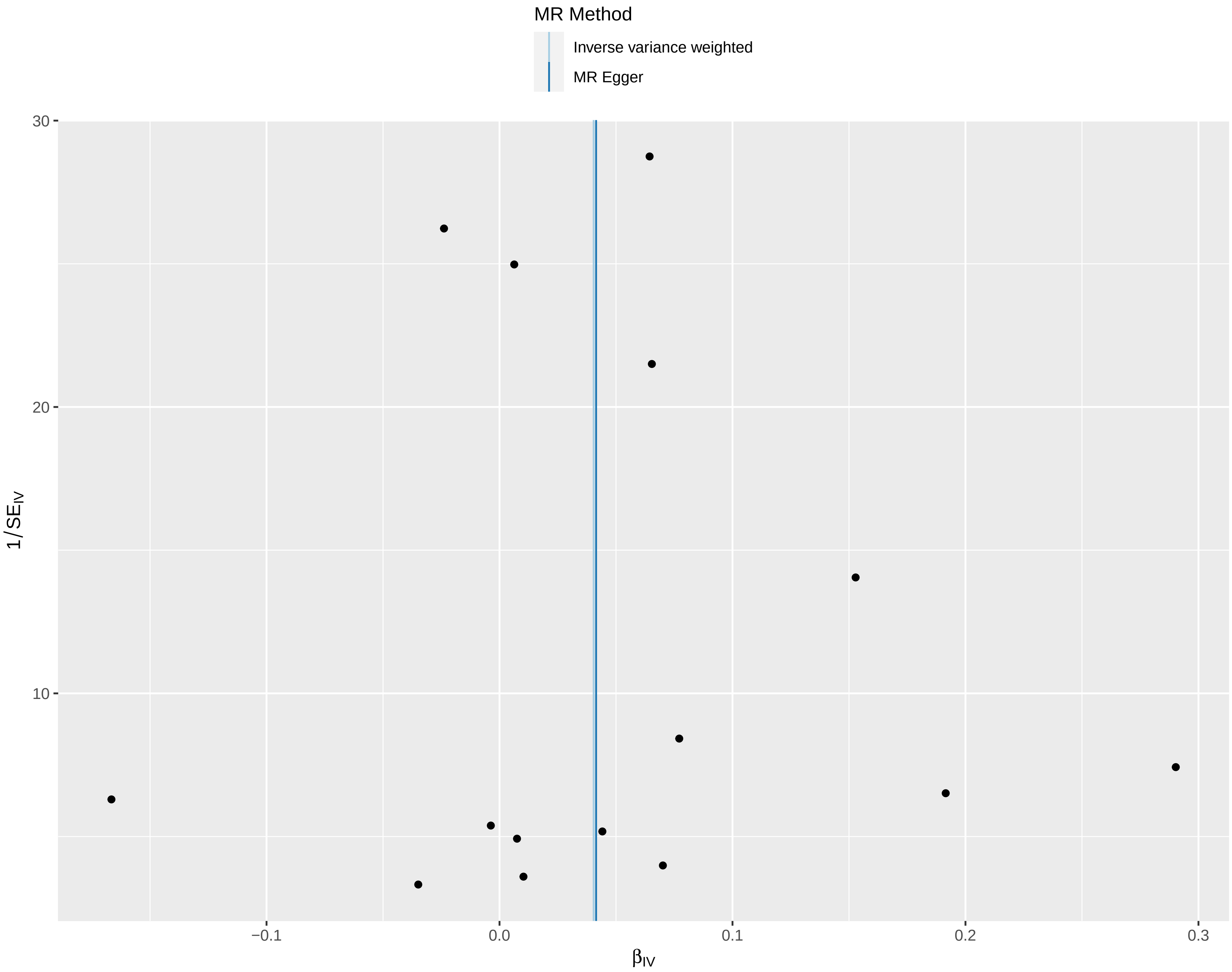
MR Method
Inverse variance weighted
MR Egger
30
20
10
1/SE_IV
−0.1
0.0
0.1
0.2
0.3
β_IV

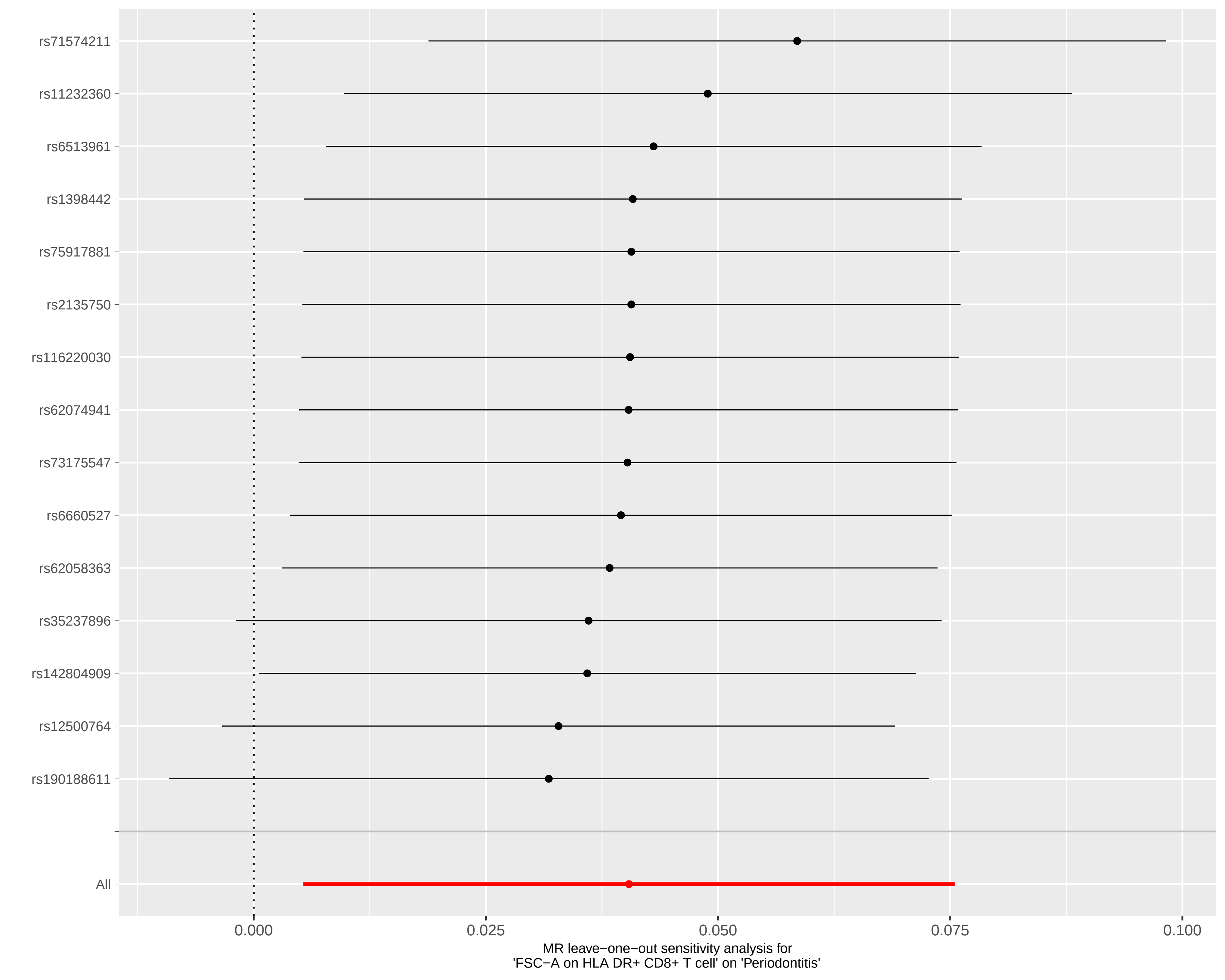
rs71574211
rs11232360
rs6513961
rs1398442
rs75917881
rs2135750
rs116220030
rs62074941
rs73175547
rs6660527
rs62058363
rs35237896
rs142804909
rs12500764
rs190188611
All
0.000
0.025
0.050
0.075
0.100
MR leave−one−out sensitivity analysis for
'FSC−A on HLA DR+ CD8+ T cell' on 'Periodontitis'

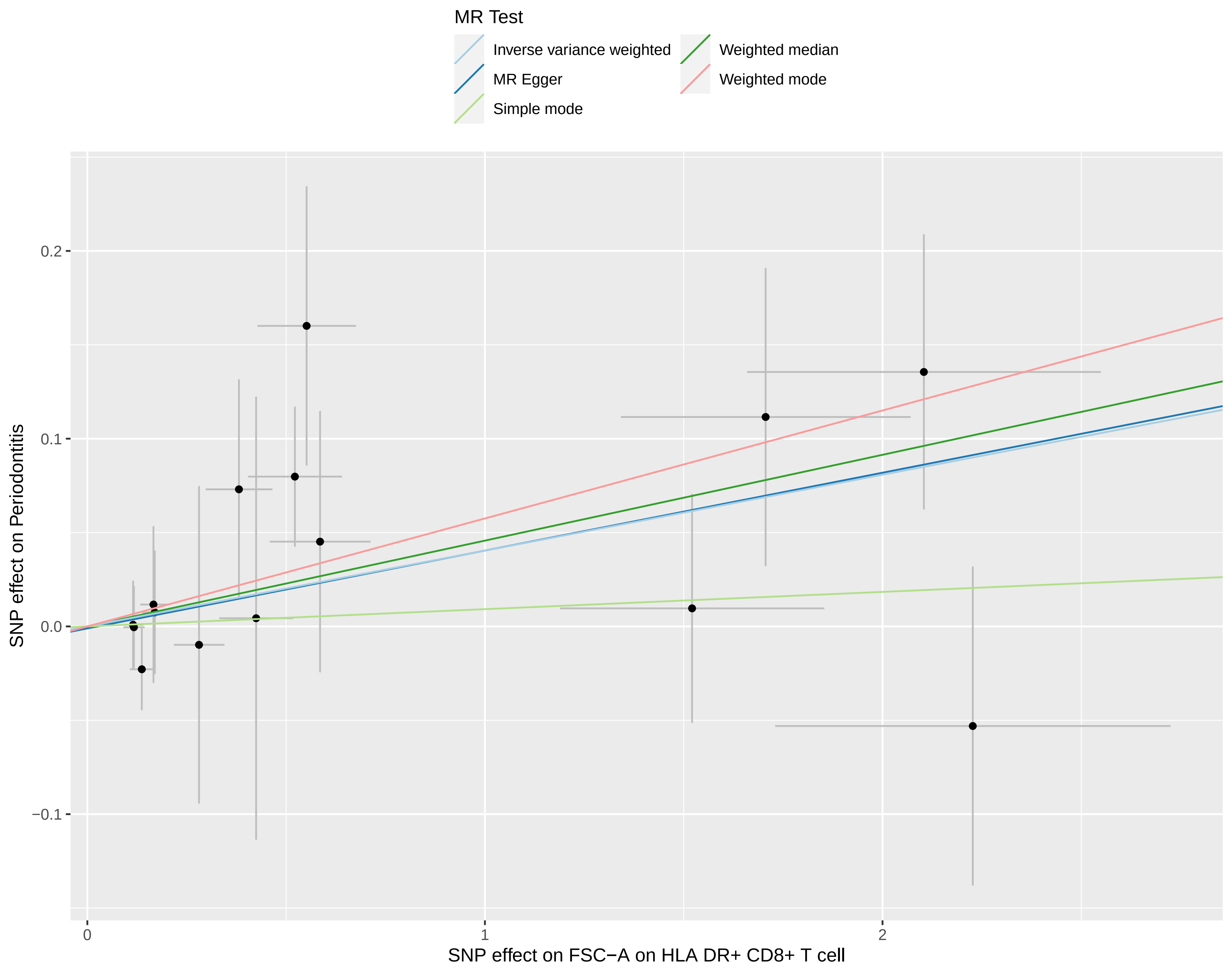
MR Test
Inverse variance weighted
MR Egger
Simple mode
Weighted median
Weighted mode
0.2
0.1
0.0
−0.1
0
1
2
SNP effect on Periodontitis
SNP effect on FSC−A on HLA DR+ CD8+ T cell

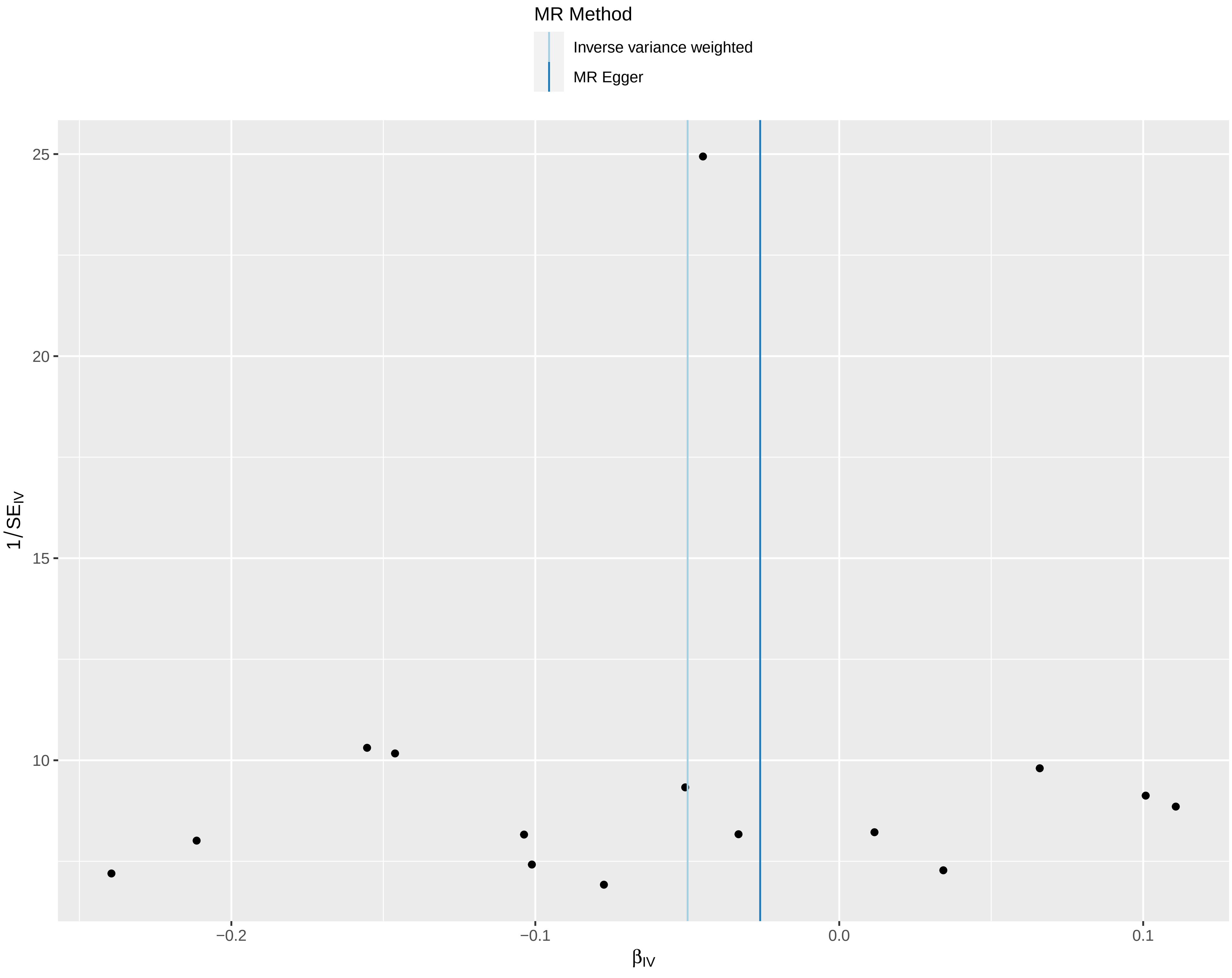
MR Method
Inverse variance weighted
MR Egger
25
20
15
10
1/SE_IV
−0.2
−0.1
0.0
0.1
β_IV

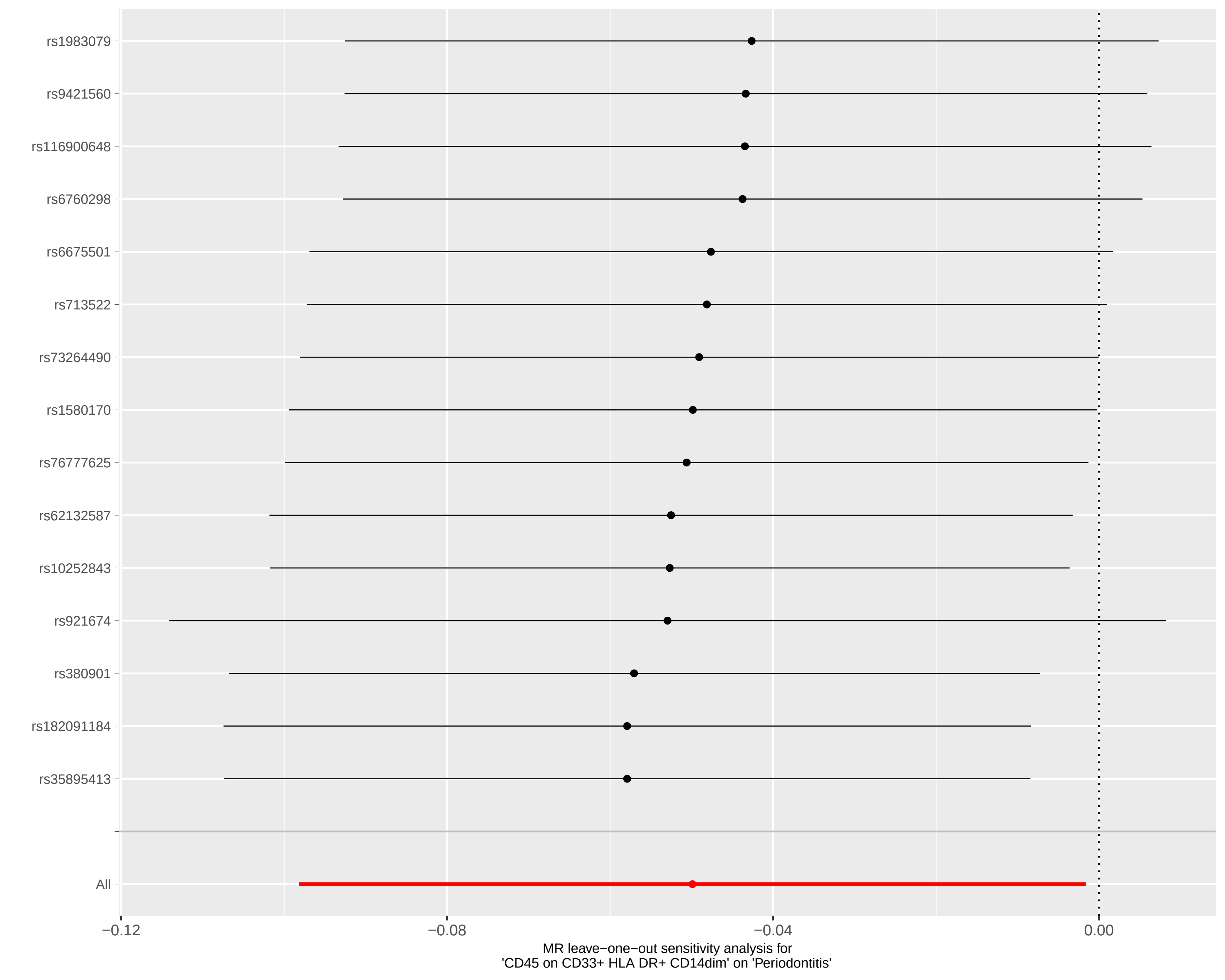
rs1983079
rs9421560
rs116900648
rs6760298
rs6675501
rs713522
rs73264490
rs1580170
rs76777625
rs62132587
rs10252843
rs921674
rs380901
rs182091184
rs35895413
All
−0.12
−0.08
−0.04
0.00
MR leave−one−out sensitivity analysis for
'CD45 on CD33+ HLA DR+ CD14dim' on 'Periodontitis'

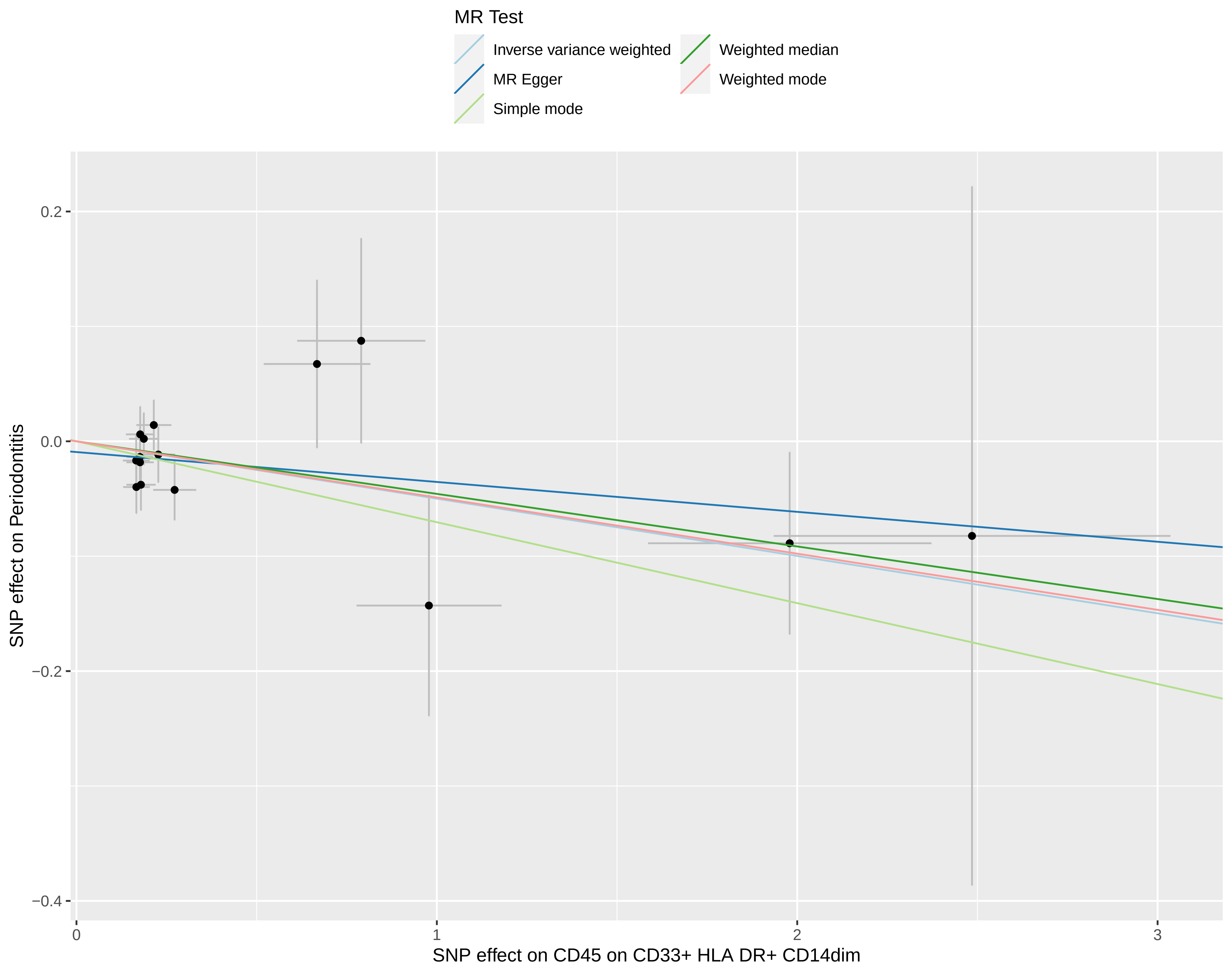
MR Test
Inverse variance weighted
MR Egger
Simple mode
Weighted median
Weighted mode
0.2
0.0
−0.2
−0.4
0
1
2
3
SNP effect on Periodontitis
SNP effect on CD45 on CD33+ HLA DR+ CD14dim

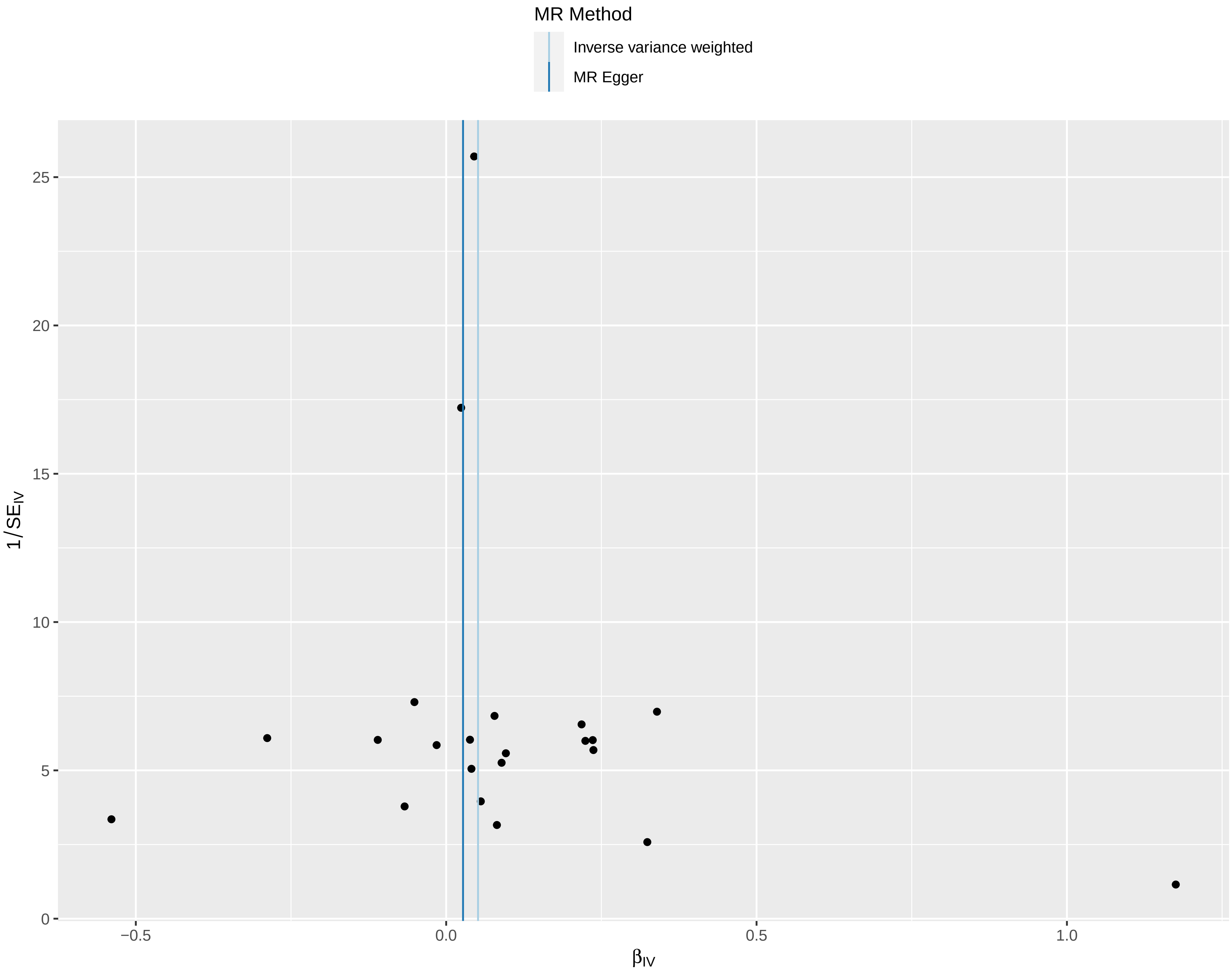

MR Method
Inverse variance weighted
MR Egger
1/SE_IV
β_IV
25
20
15
10
5
0
−0.5
0.0
0.5
1.0

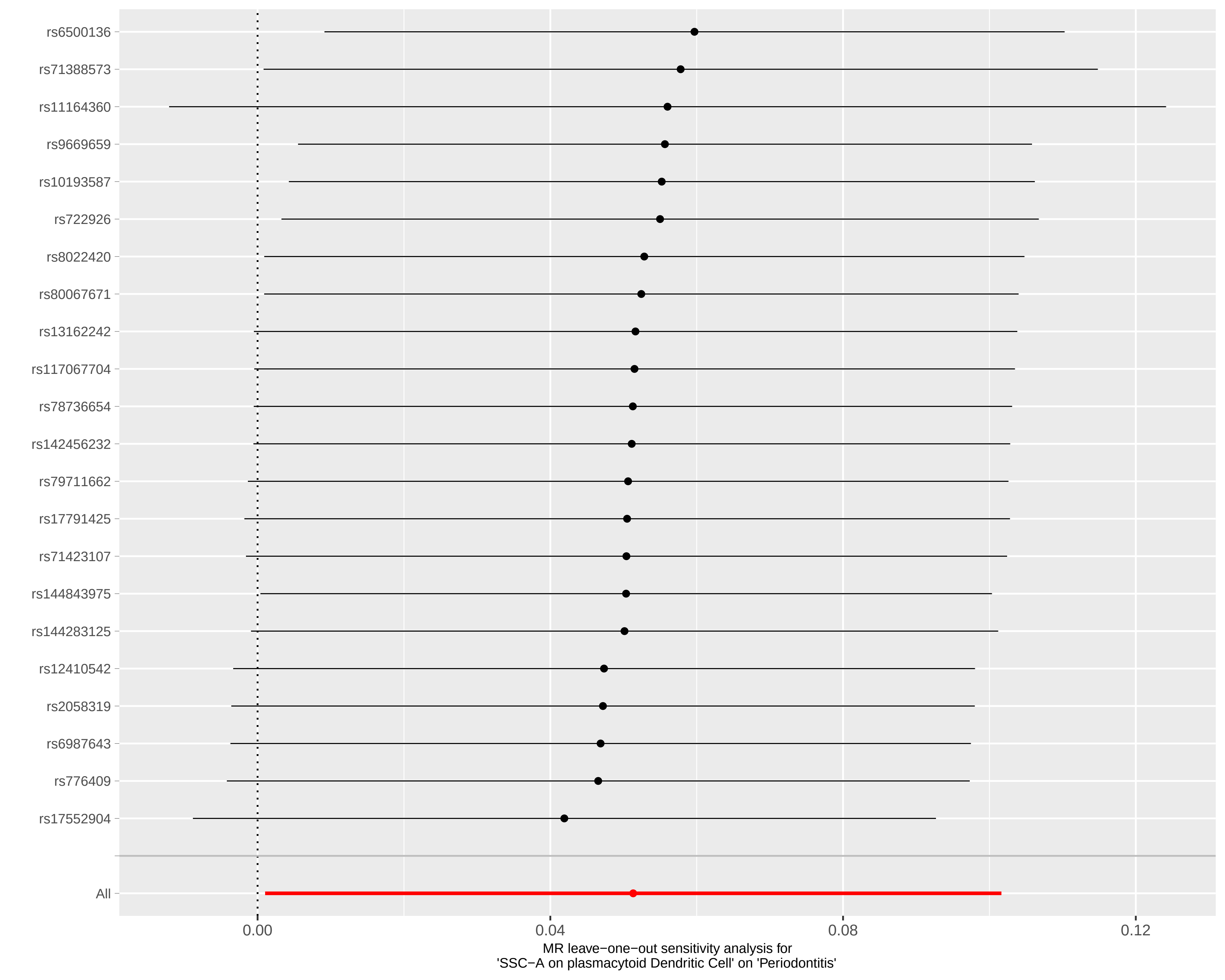
rs6500136
rs71388573
rs11164360
rs9669659
rs10193587
rs722926
rs8022420
rs80067671
rs13162242
rs117067704
rs78736654
rs142456232
rs79711662
rs17791425
rs71423107
rs144843975
rs144283125
rs12410542
rs2058319
rs6987643
rs776409
rs17552904
All
0.00
0.04
0.08
0.12
MR leave−one−out sensitivity analysis for
'SSC−A on plasmacytoid Dendritic Cell' on 'Periodontitis'

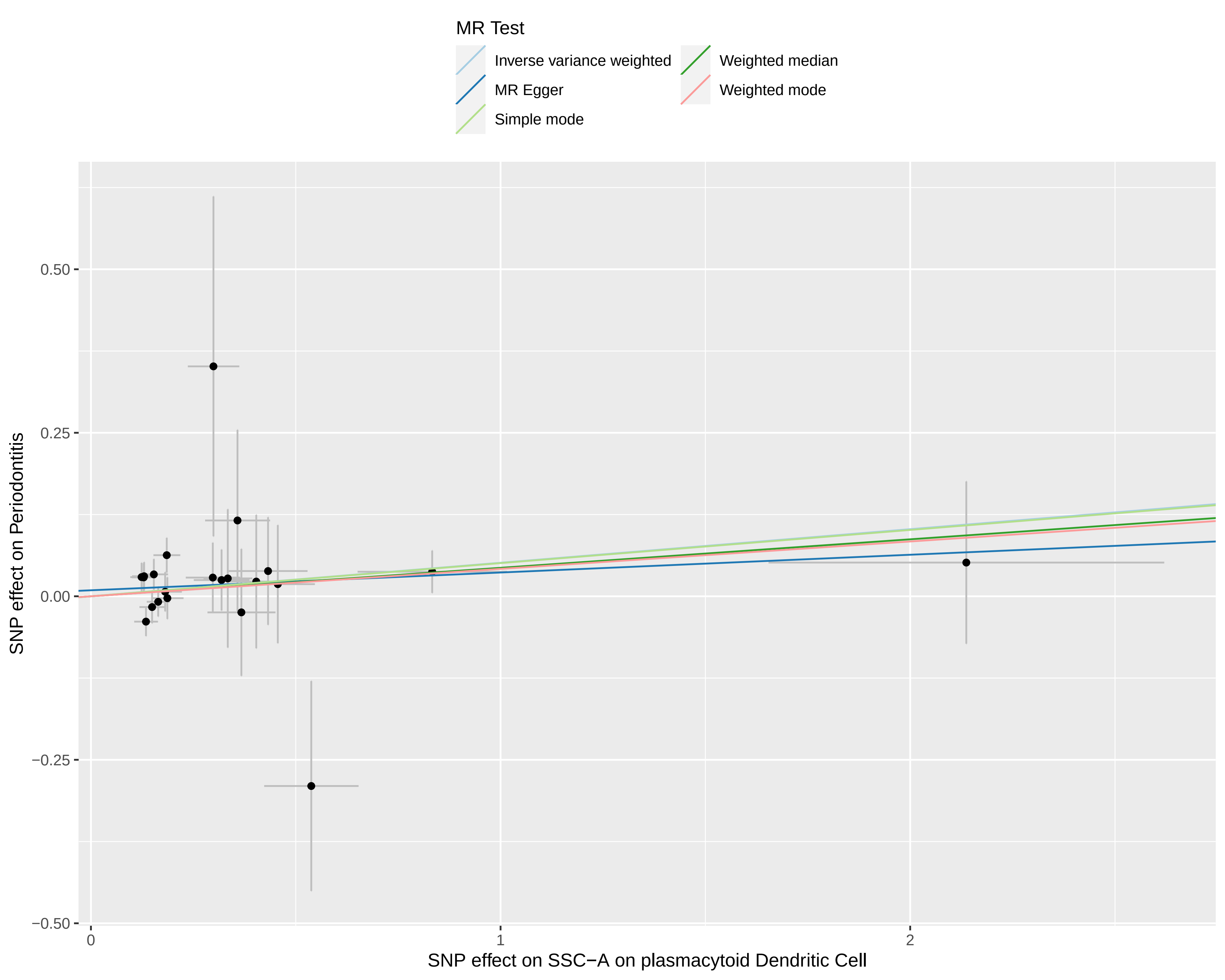
MR Test
Inverse variance weighted
MR Egger
Simple mode
Weighted median
Weighted mode
0.50
0.25
0.00
−0.25
−0.50
SNP effect on Periodontitis
0
1
2
SNP effect on SSC−A on plasmacytoid Dendritic Cell

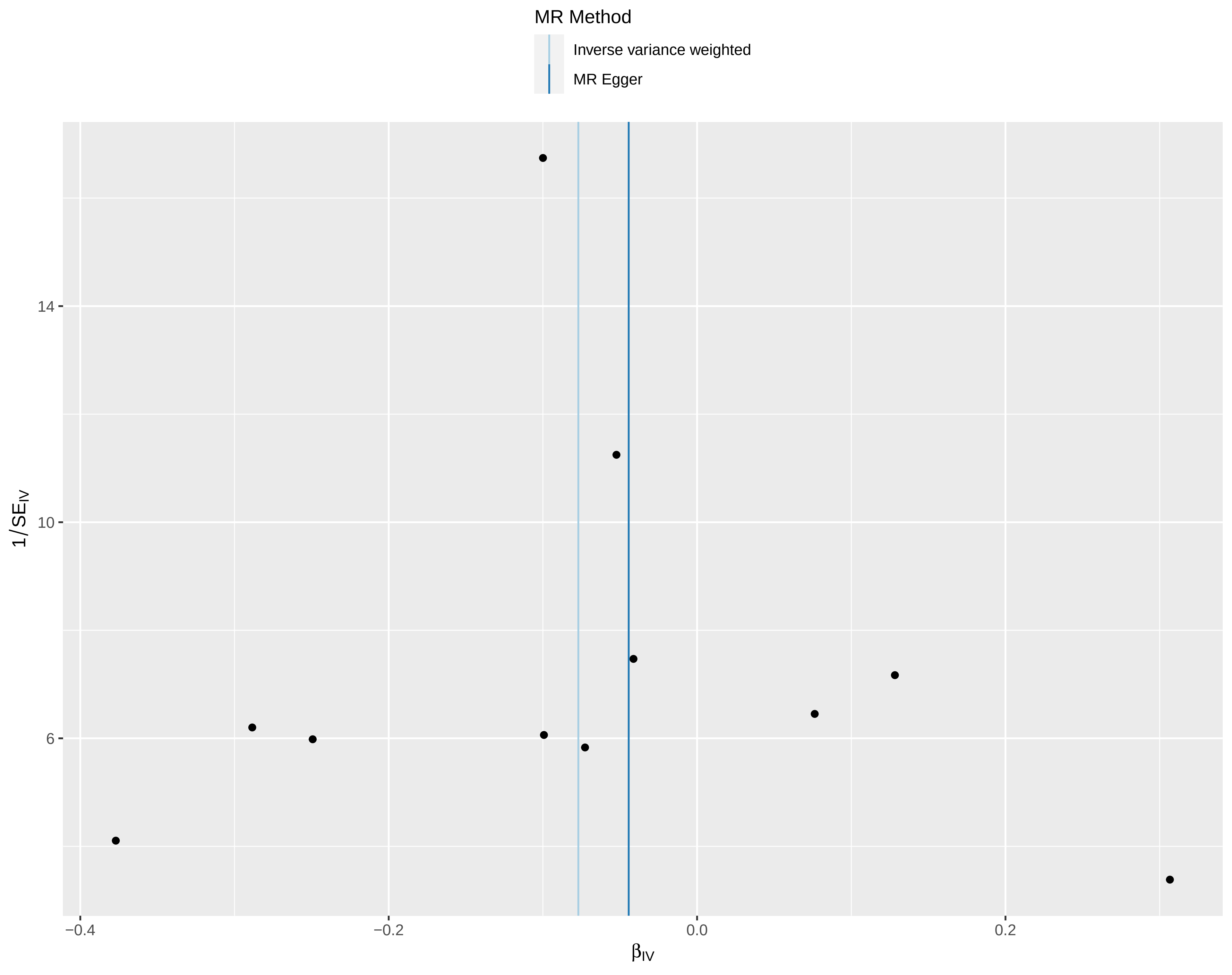
MR Method
Inverse variance weighted
MR Egger
14
10
6
$1/SE_{IV}$
−0.4
−0.2
0.0
0.2
β_{IV}

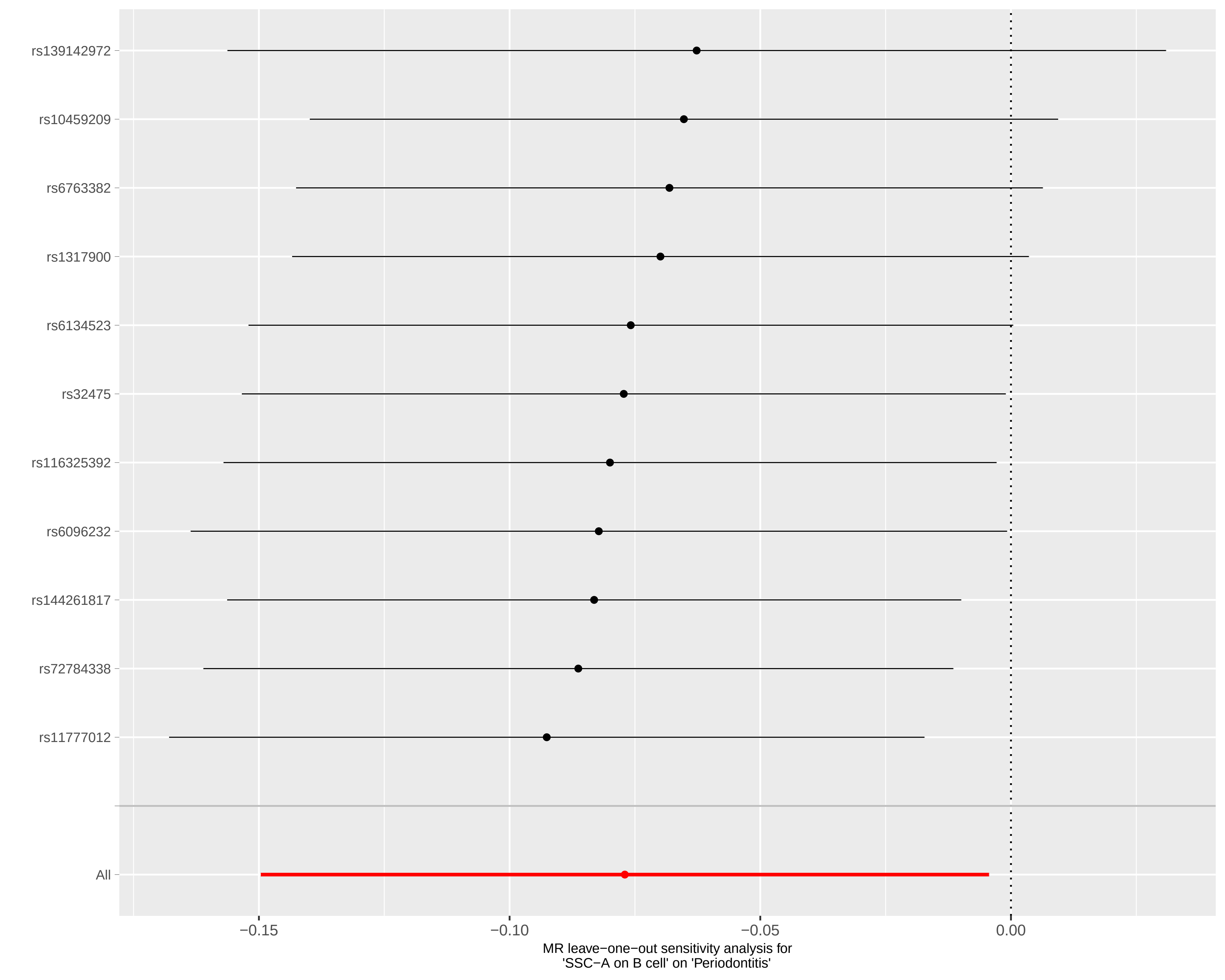

rs139142972
rs10459209
rs6763382
rs1317900
rs6134523
rs32475
rs116325392
rs6096232
rs144261817
rs72784338
rs11777012
All
−0.15
−0.10
−0.05
0.00
MR leave−one−out sensitivity analysis for
'SSC−A on B cell' on 'Periodontitis'

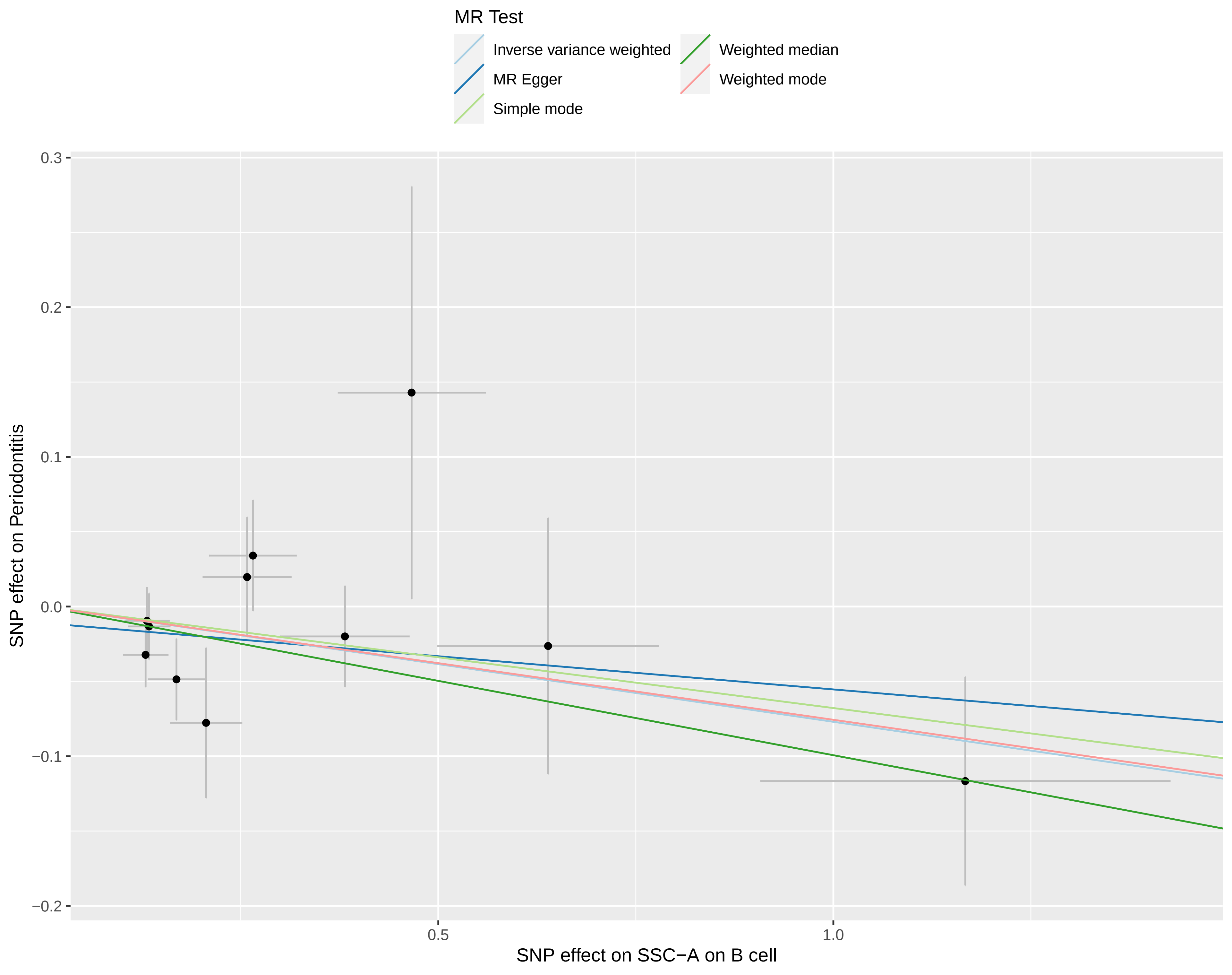
MR Test
Inverse variance weighted
MR Egger
Simple mode
Weighted median
Weighted mode
0.3
0.2
0.1
0.0
−0.1
−0.2
0.5
1.0
SNP effect on Periodontitis
SNP effect on SSC−A on B cell
