# Supplementary material for: Causal role of immune cells in chronic periodontitis: a bidirectional Mendelian randomization study
Source: BMC Oral Health. 2024 Jul 16;24:806. doi: 10.1186/s12903-024-04592-0 (PMC11253491; doi:10.1186/s12903-024-04592-0)
Supplement: Supplementary file 3 — Supplementary Material 3. [file 12903_2024_4592_MOESM3_ESM.pdf]

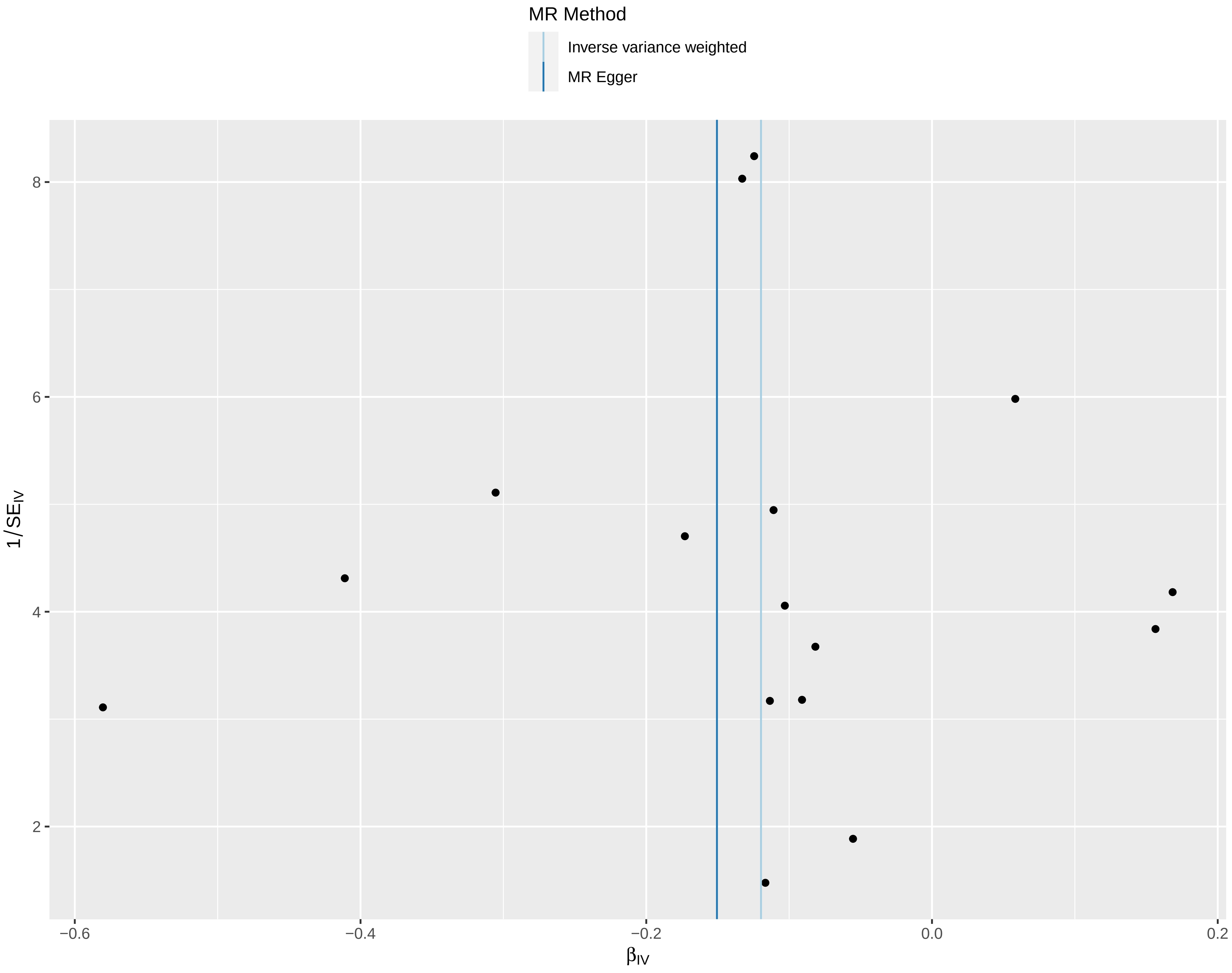
MR Method
Inverse variance weighted
MR Egger
1/SE_IV
8
6
4
2
−0.6
−0.4
−0.2
0.0
0.2
β_IV

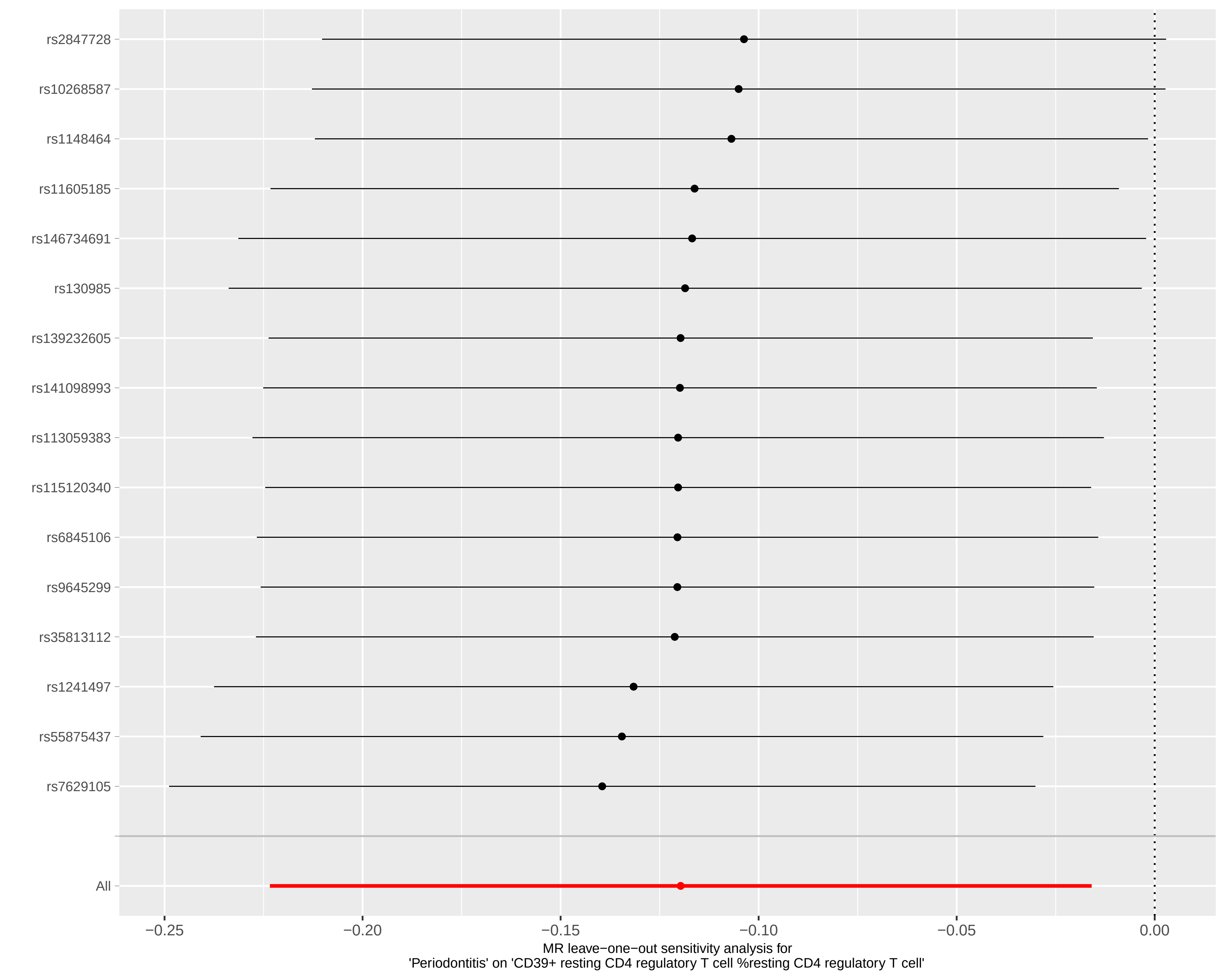
rs2847728
rs10268587
rs1148464
rs11605185
rs146734691
rs130985
rs139232605
rs141098993
rs113059383
rs115120340
rs6845106
rs9645299
rs35813112
rs1241497
rs55875437
rs7629105
All
−0.25
−0.20
−0.15
−0.10
−0.05
0.00
MR leave−one−out sensitivity analysis for
'Periodontitis' on 'CD39+ resting CD4 regulatory T cell %resting CD4 regulatory T cell'

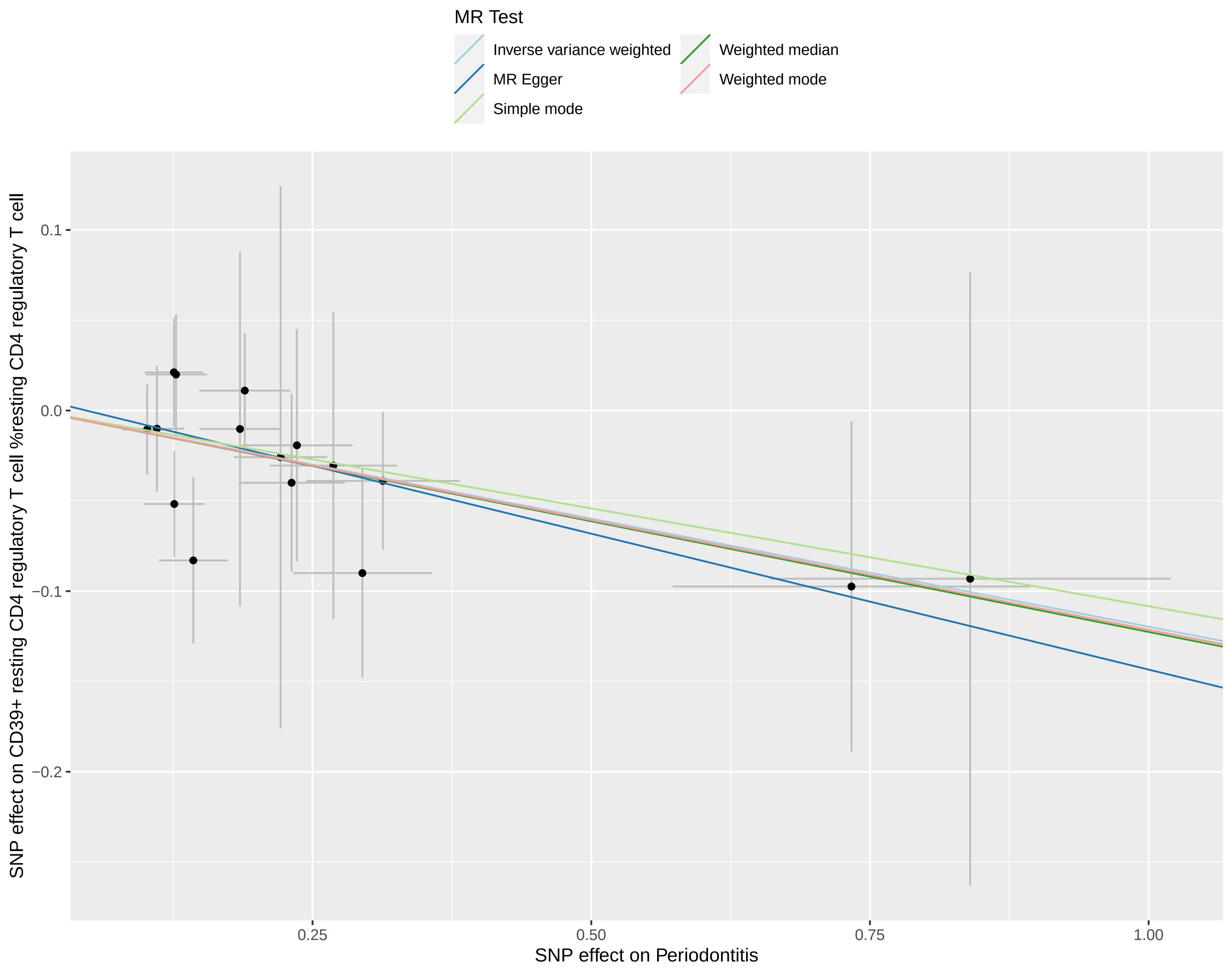

MR Test
Inverse variance weighted
MR Egger
Simple mode
Weighted median
Weighted mode
0.1
0.0
−0.1
−0.2
0.25
0.50
0.75
1.00
SNP effect on CD39+ resting CD4 regulatory T cell %resting CD4 regulatory T cell
SNP effect on Periodontitis

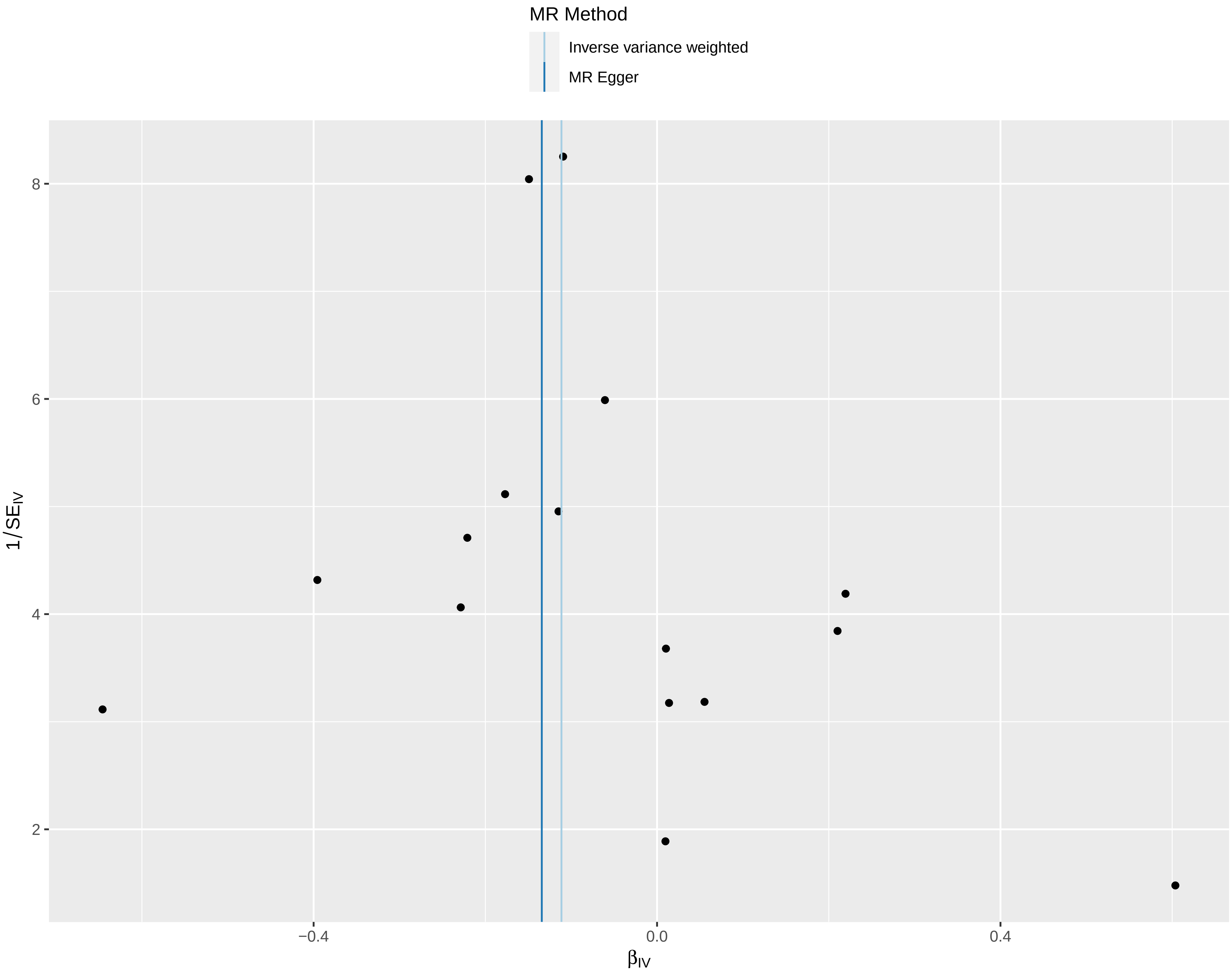

MR Method
Inverse variance weighted
MR Egger
8
6
4
2
−0.4
0.0
0.4
$1/SE_{IV}$
β_{IV}

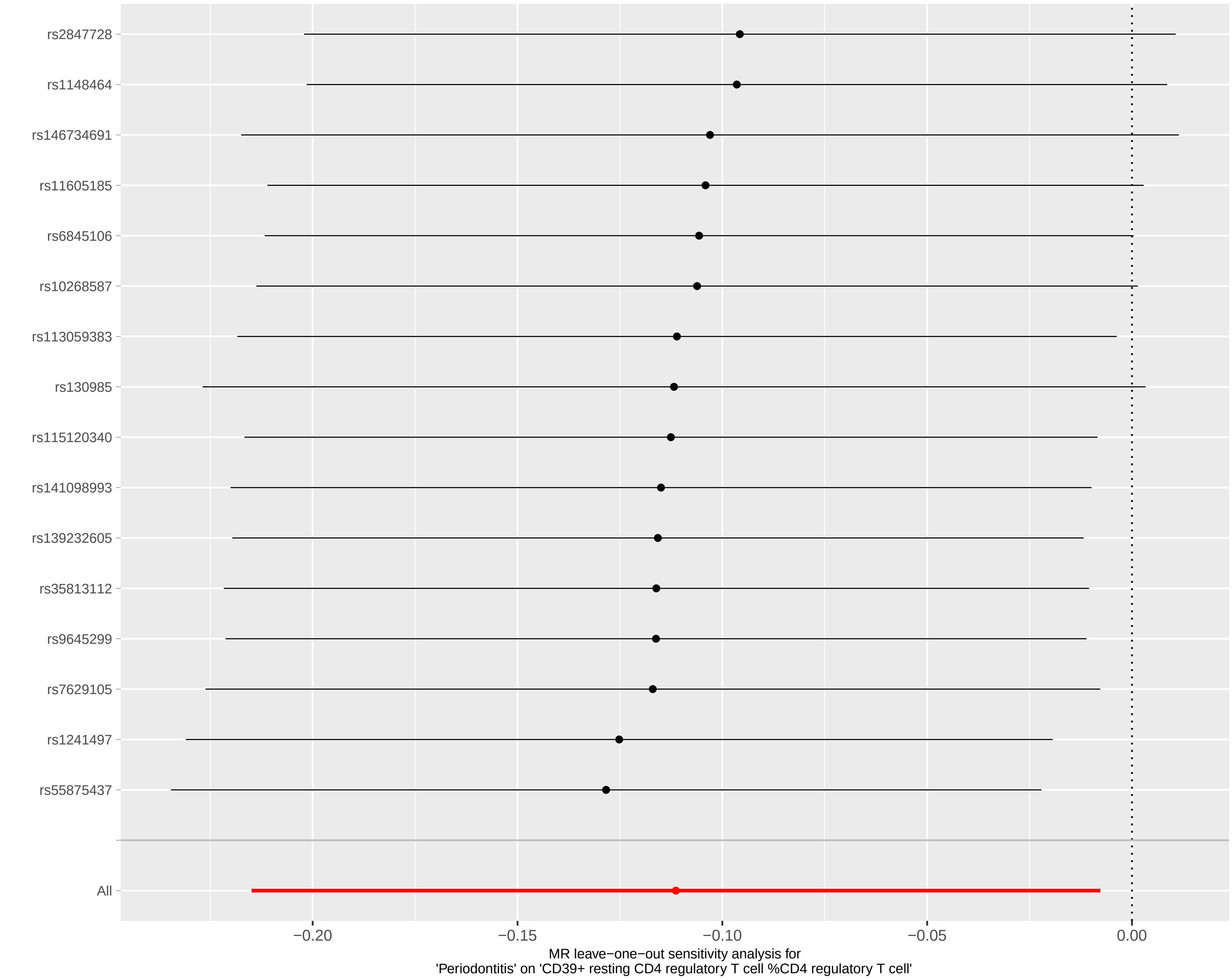
rs2847728
rs1148464
rs146734691
rs11605185
rs6845106
rs10268587
rs113059383
rs130985
rs115120340
rs141098993
rs139232605
rs35813112
rs9645299
rs7629105
rs1241497
rs55875437
All
−0.20
−0.15
−0.10
−0.05
0.00
MR leave−one−out sensitivity analysis for
'Periodontitis' on 'CD39+ resting CD4 regulatory T cell %CD4 regulatory T cell'

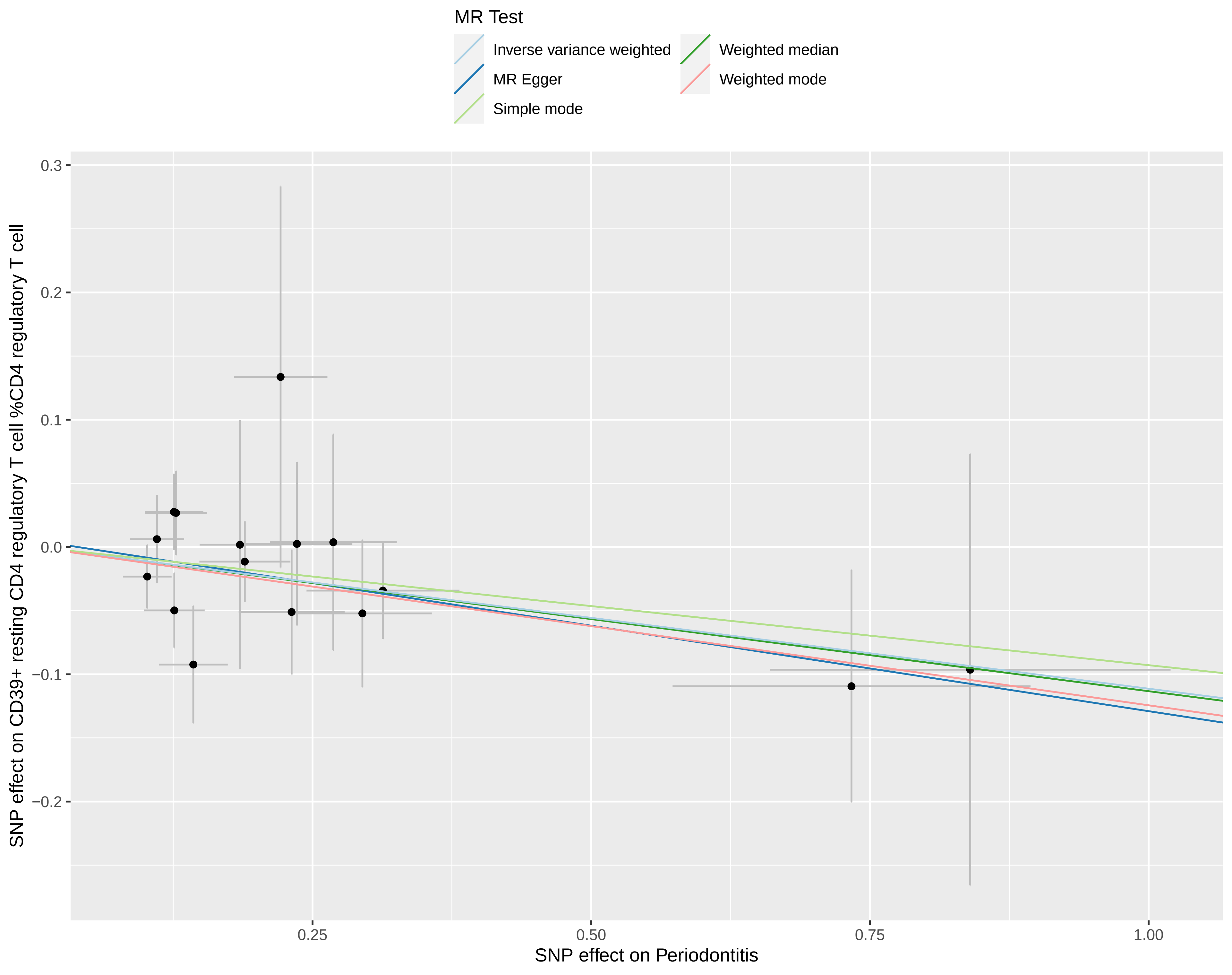
MR Test
Inverse variance weighted
MR Egger
Simple mode
Weighted median
Weighted mode
0.3
0.2
0.1
0.0
−0.1
−0.2
SNP effect on CD39+ resting CD4 regulatory T cell %CD4 regulatory T cell
0.25
0.50
0.75
1.00
SNP effect on Periodontitis

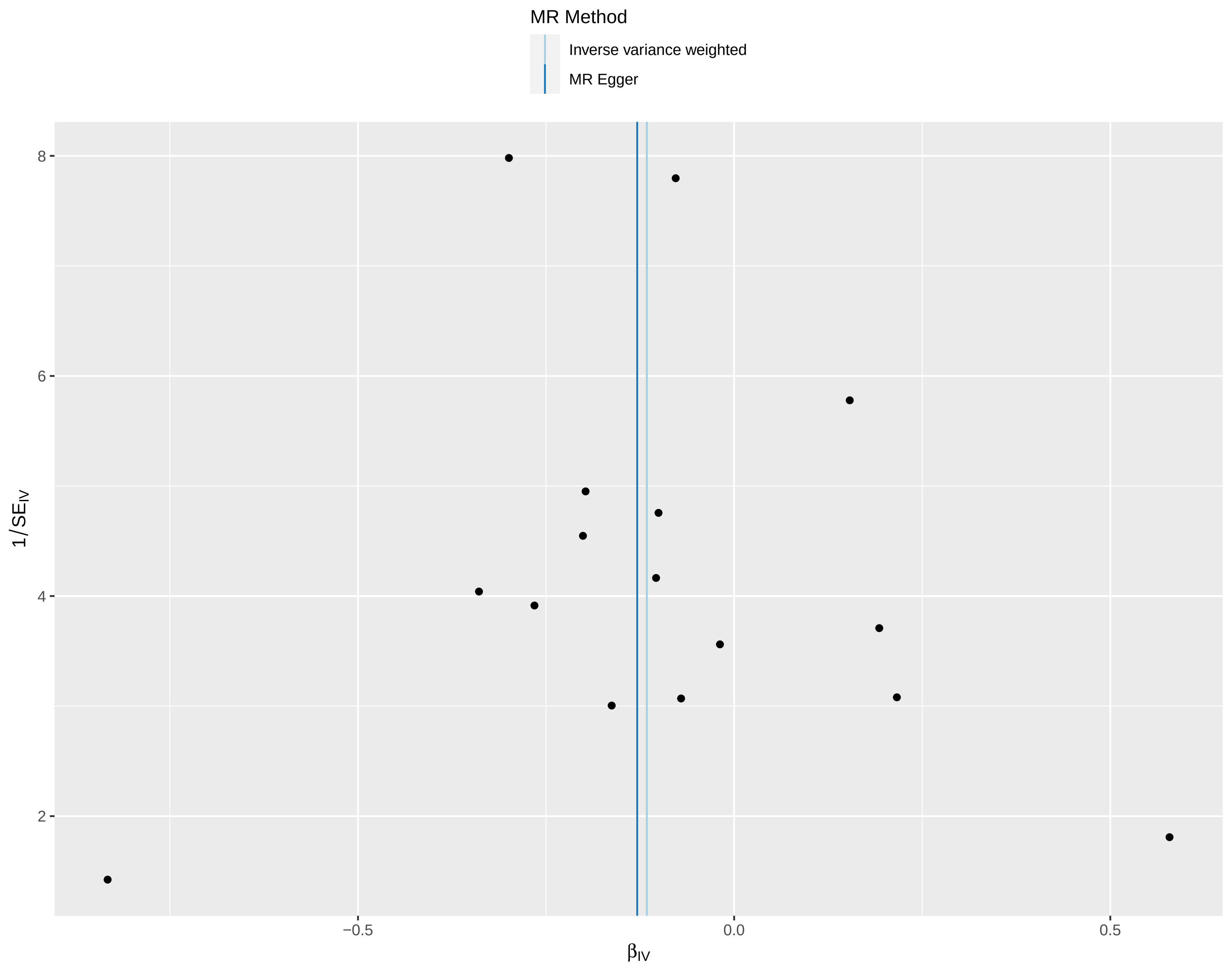

MR Method
Inverse variance weighted
MR Egger
8
6
4
2
1/SE_IV
−0.5
0.0
0.5
β_IV

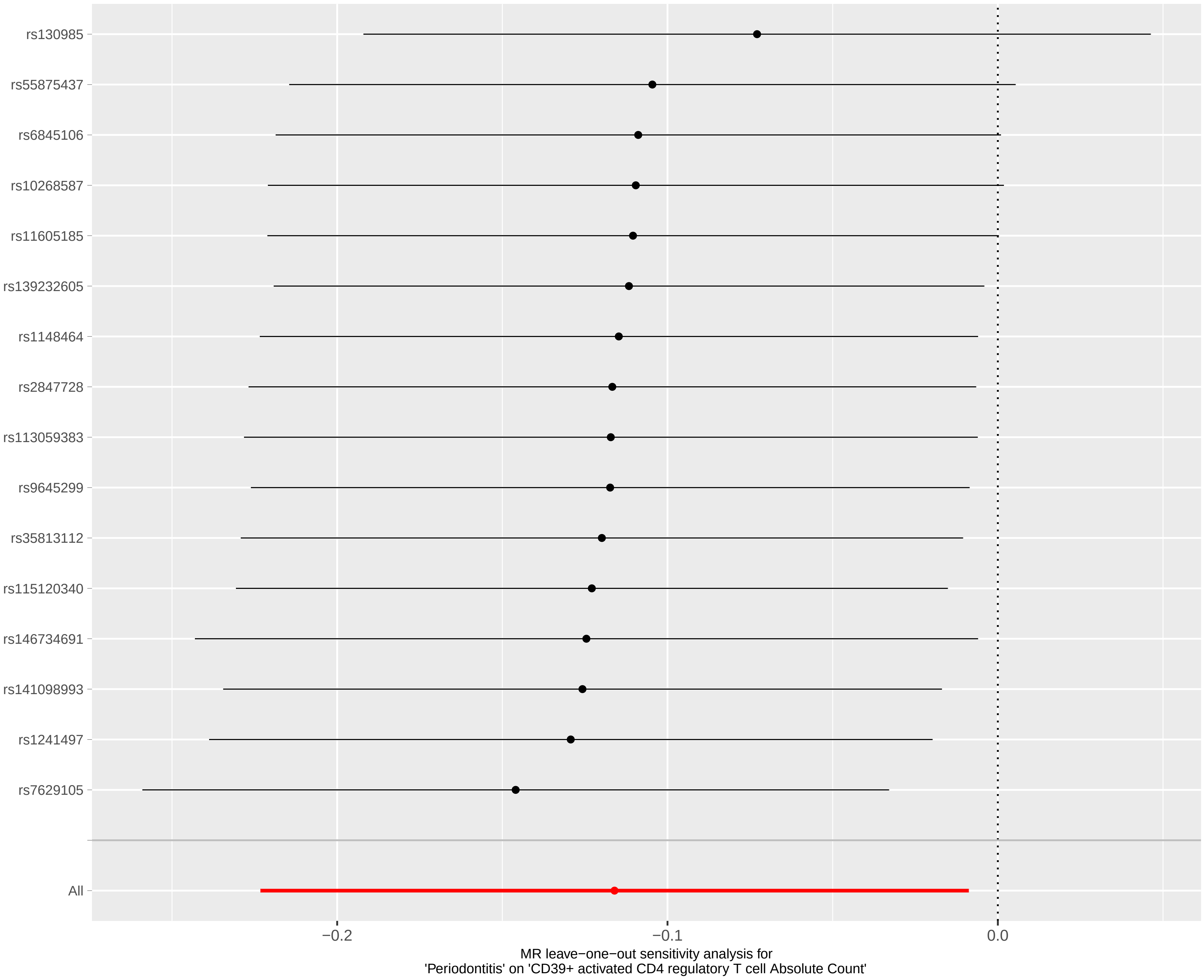
rs130985
rs55875437
rs6845106
rs10268587
rs11605185
rs139232605
rs1148464
rs2847728
rs113059383
rs9645299
rs35813112
rs115120340
rs146734691
rs141098993
rs1241497
rs7629105
All
−0.2
−0.1
0.0
MR leave−one−out sensitivity analysis for
'Periodontitis' on 'CD39+ activated CD4 regulatory T cell Absolute Count'

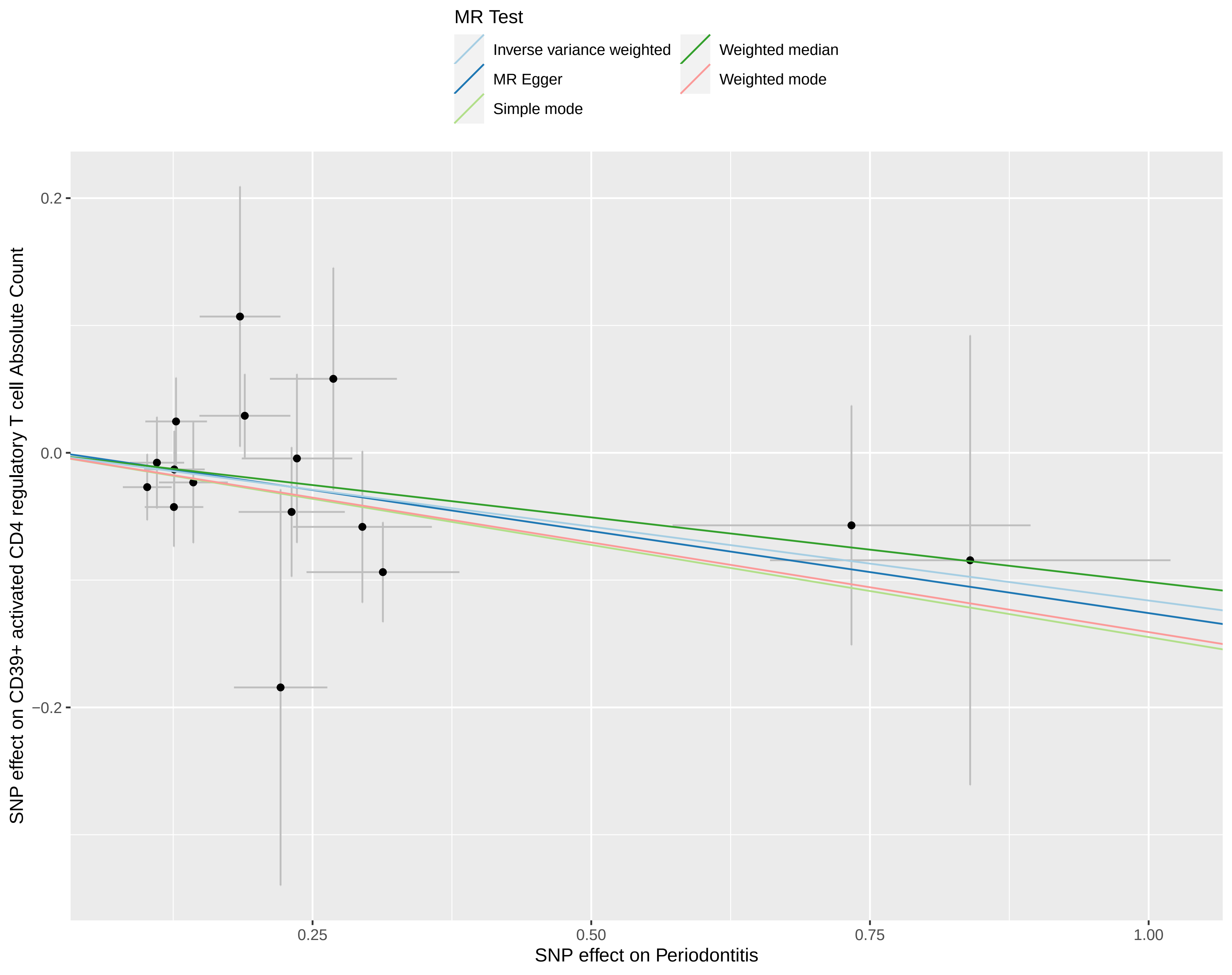

MR Test
Inverse variance weighted
MR Egger
Simple mode
Weighted median
Weighted mode
0.2
0.0
−0.2
0.25
0.50
0.75
1.00
SNP effect on Periodontitis
SNP effect on CD39+ activated CD4 regulatory T cell Absolute Count

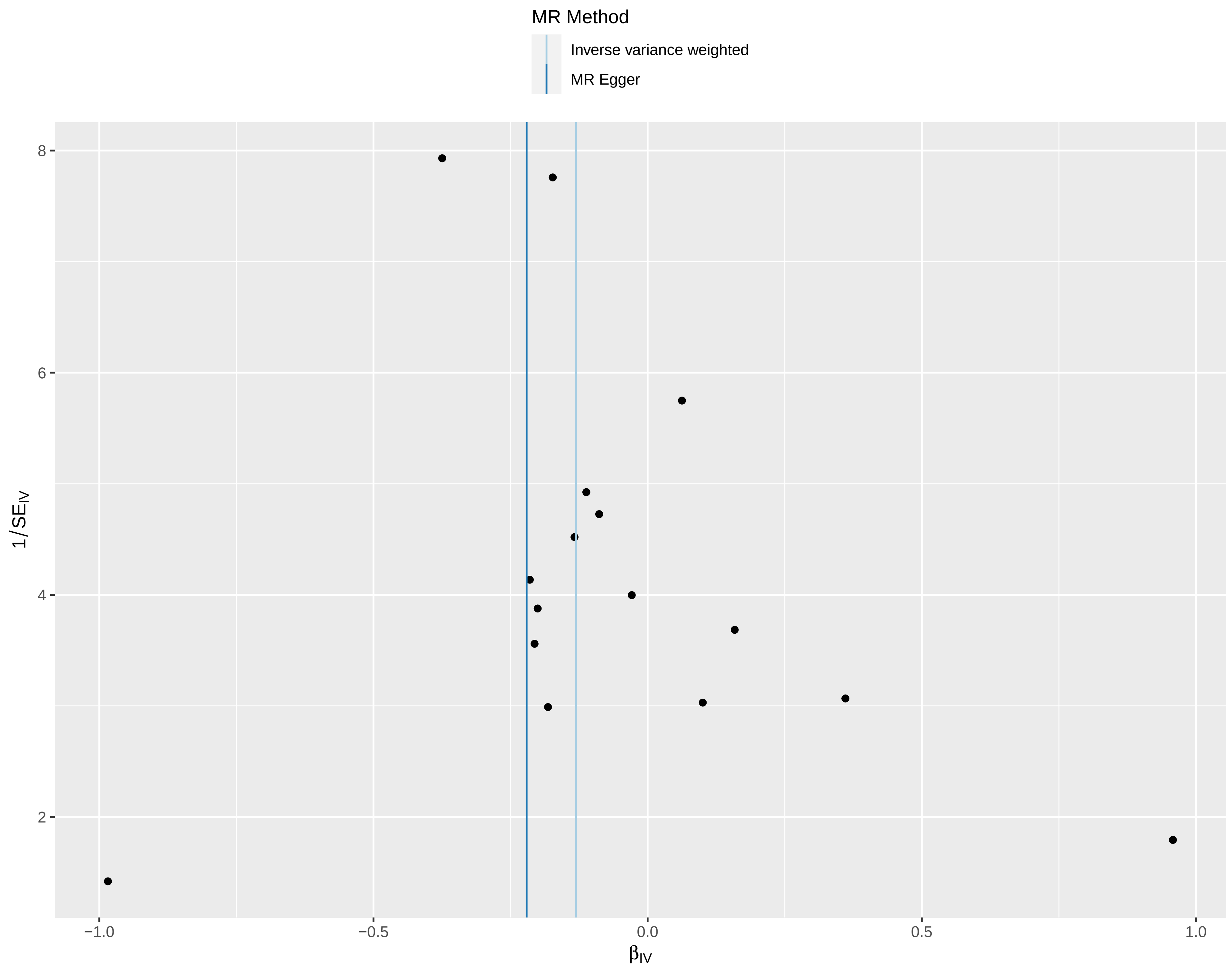

MR Method
Inverse variance weighted
MR Egger
8
6
4
2
1/SE_IV
−1.0
−0.5
0.0
0.5
1.0
β_IV

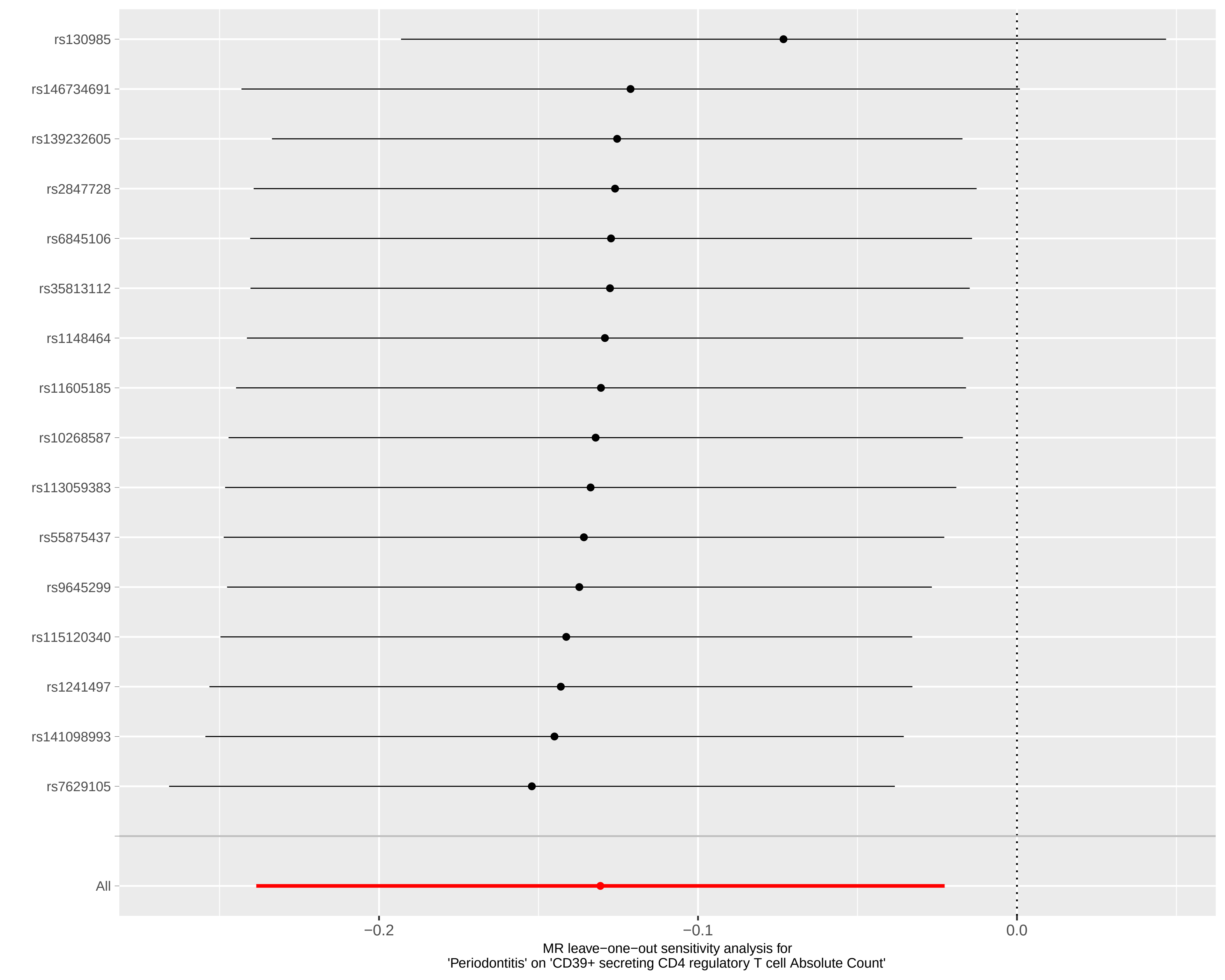
rs130985
rs146734691
rs139232605
rs2847728
rs6845106
rs35813112
rs1148464
rs11605185
rs10268587
rs113059383
rs55875437
rs9645299
rs115120340
rs1241497
rs141098993
rs7629105
All
−0.2
−0.1
0.0
MR leave−one−out sensitivity analysis for
'Periodontitis' on 'CD39+ secreting CD4 regulatory T cell Absolute Count'

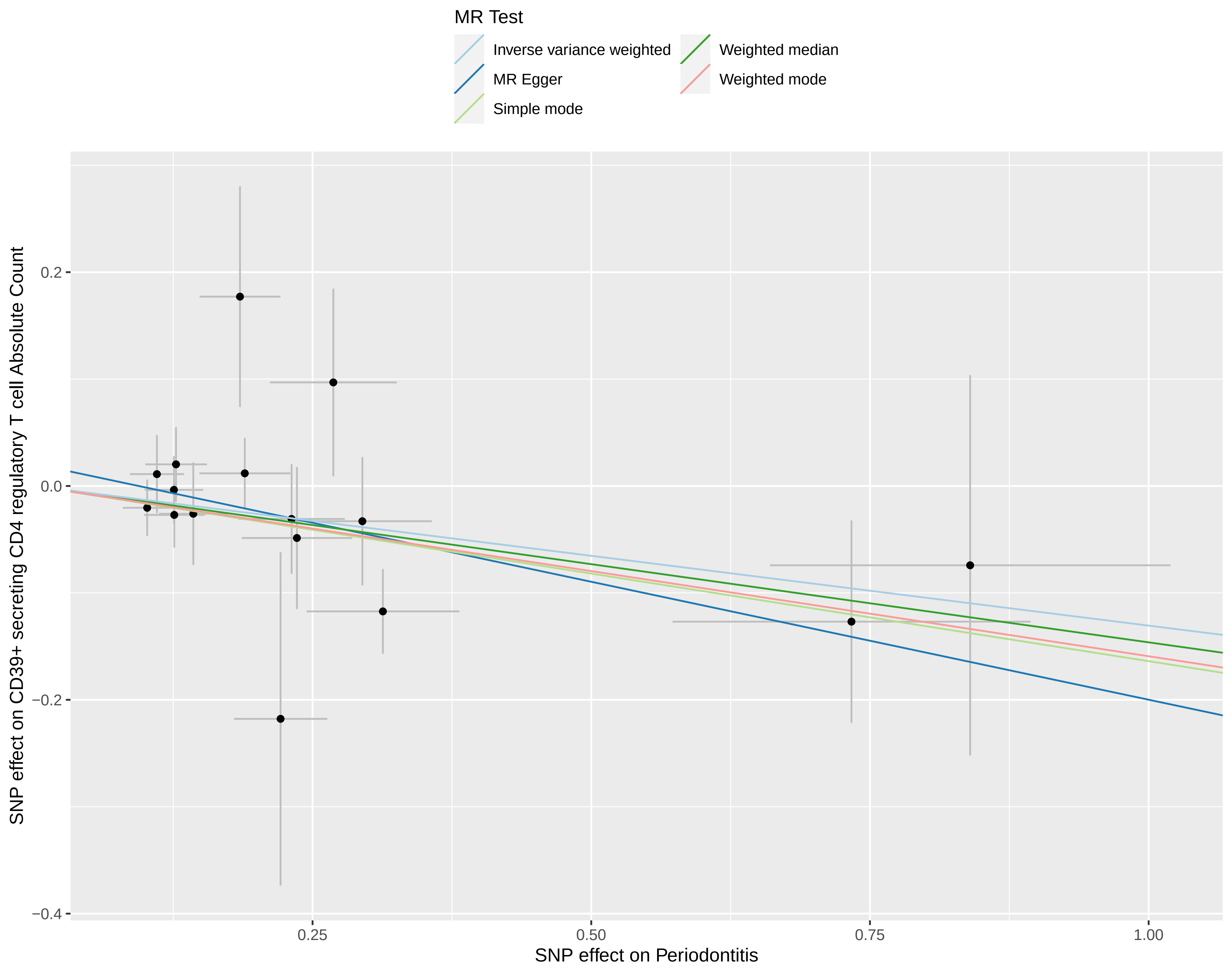

MR Test
Inverse variance weighted
MR Egger
Simple mode
Weighted median
Weighted mode
0.2
0.0
−0.2
−0.4
0.25
0.50
0.75
1.00
SNP effect on CD39+ secreting CD4 regulatory T cell Absolute Count
SNP effect on Periodontitis

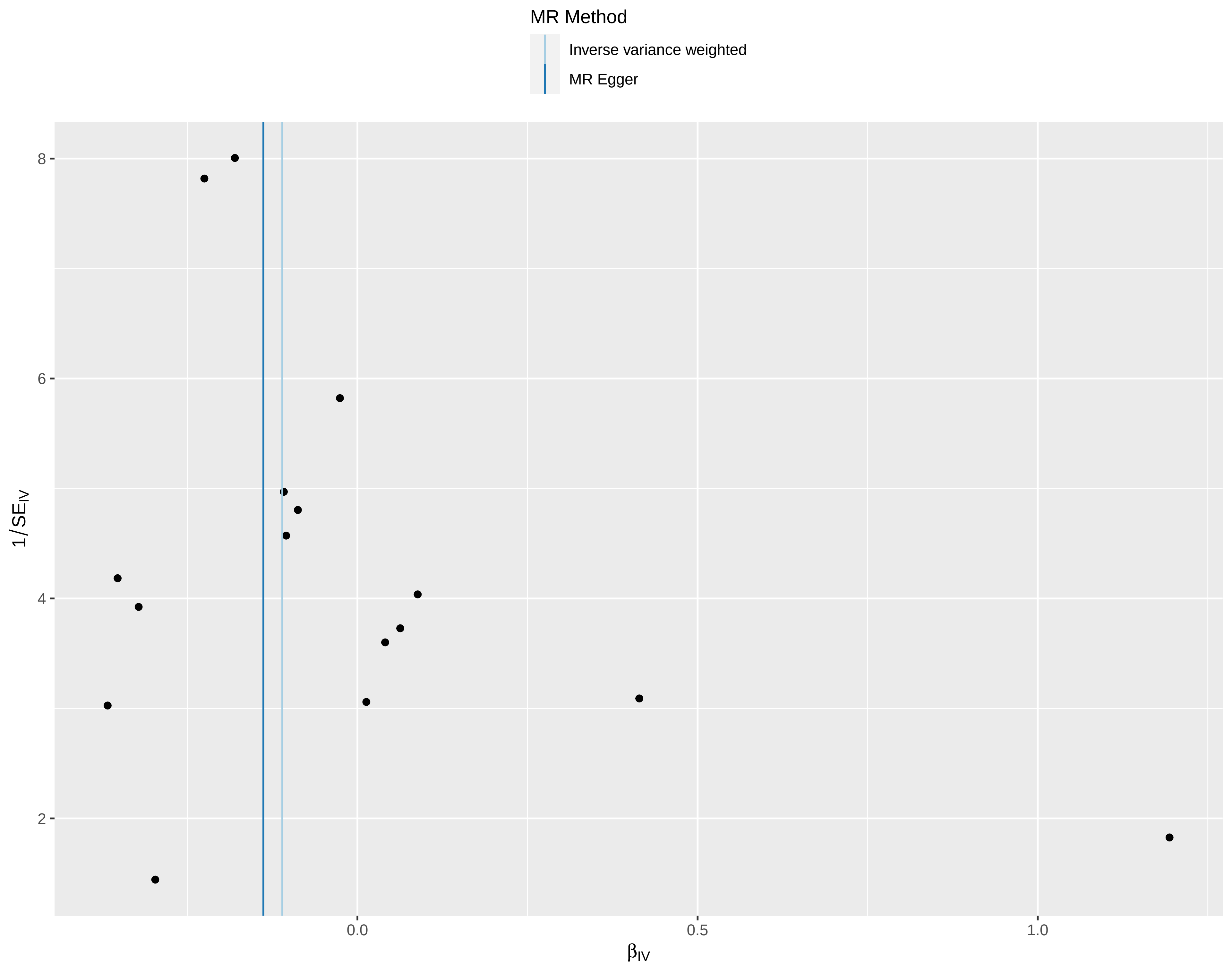
MR Method
Inverse variance weighted
MR Egger
8
6
4
2
1/SE_IV
0.0
0.5
1.0
β_IV

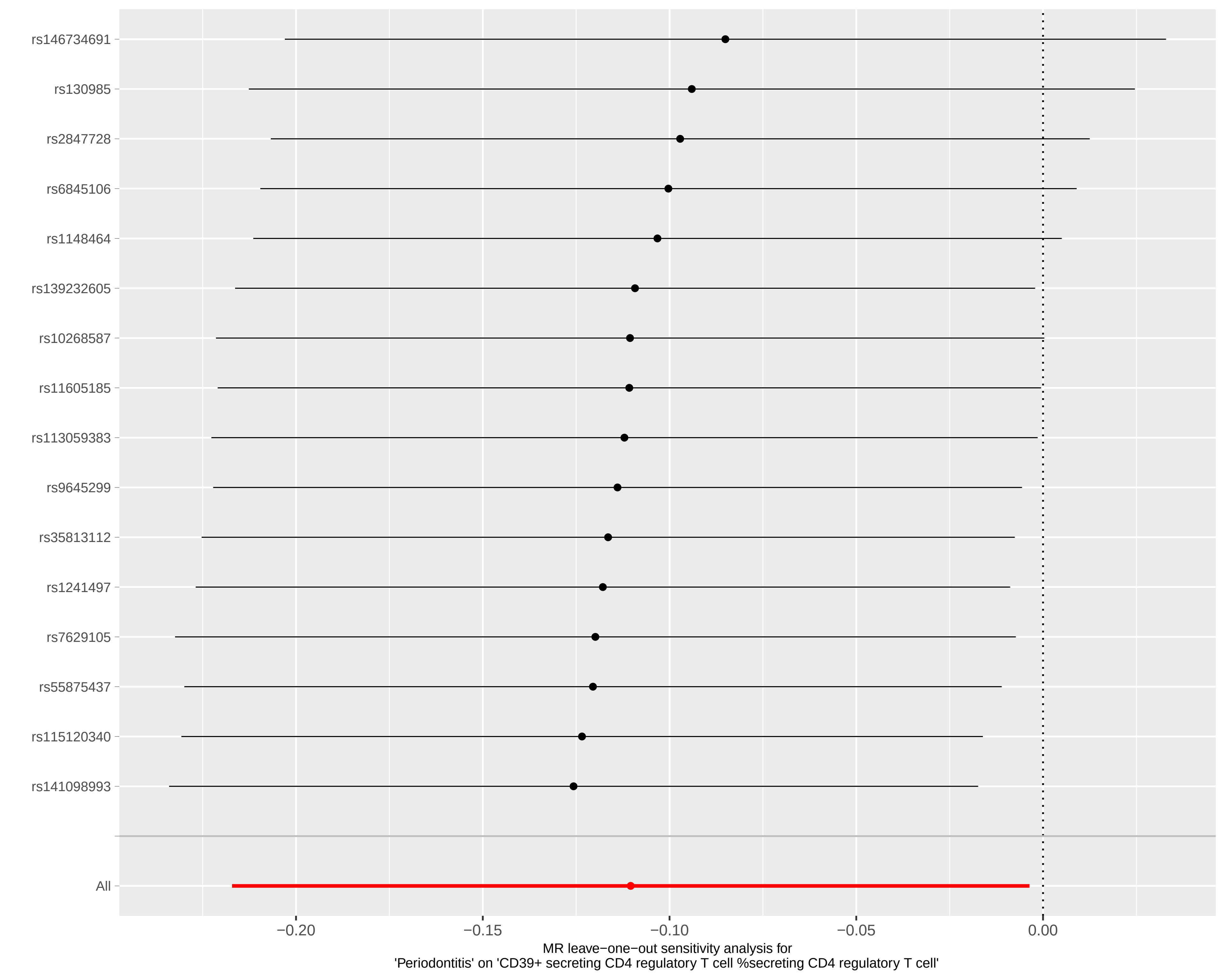

rs146734691
rs130985
rs2847728
rs6845106
rs1148464
rs139232605
rs10268587
rs11605185
rs113059383
rs9645299
rs35813112
rs1241497
rs7629105
rs55875437
rs115120340
rs141098993
All
−0.20
−0.15
−0.10
−0.05
0.00
MR leave−one−out sensitivity analysis for
'Periodontitis' on 'CD39+ secreting CD4 regulatory T cell %secreting CD4 regulatory T cell'

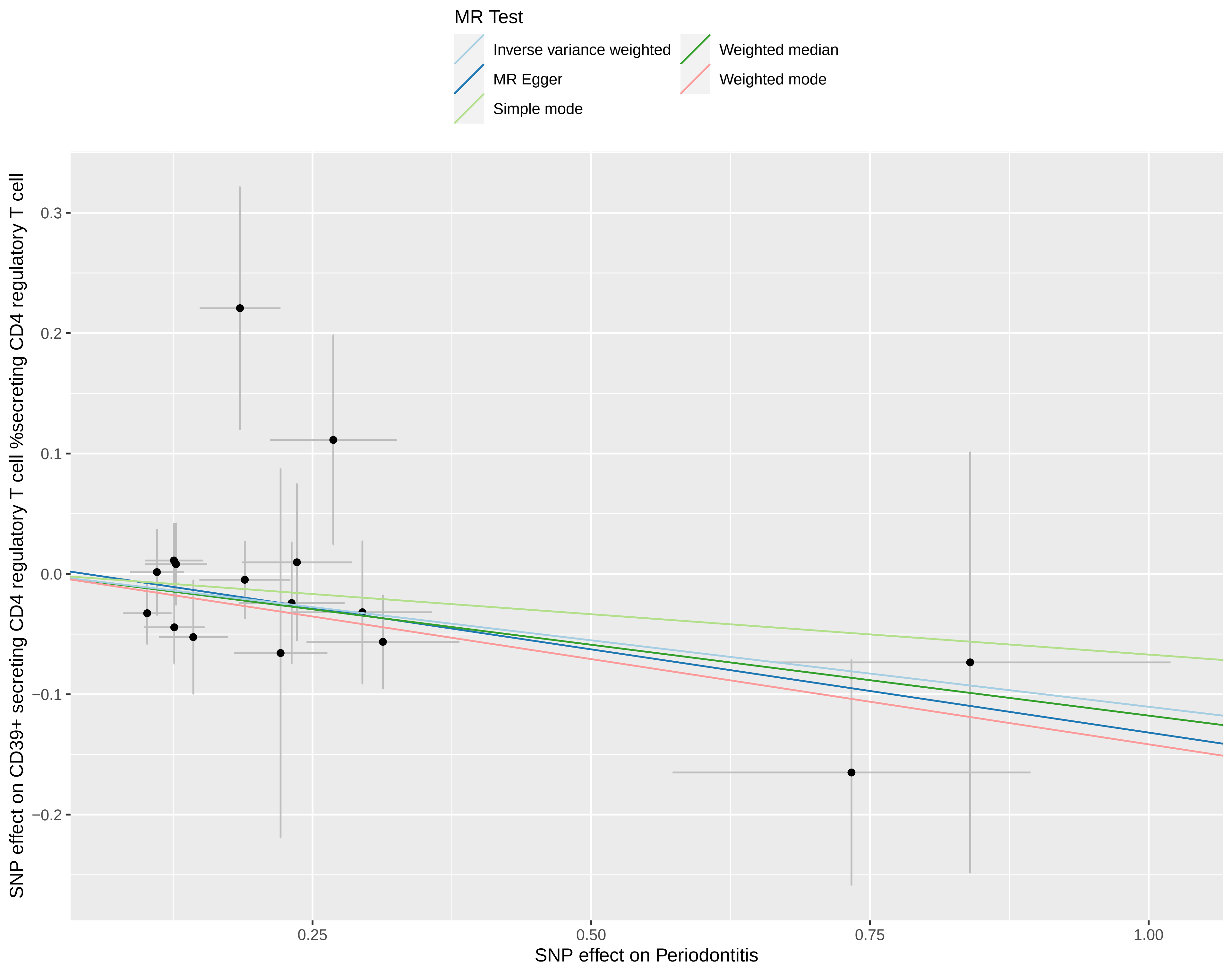

MR Test
Inverse variance weighted
MR Egger
Simple mode
Weighted median
Weighted mode
SNP effect on CD39+ secreting CD4 regulatory T cell %secreting CD4 regulatory T cell
0.3
0.2
0.1
0.0
−0.1
−0.2
0.25
0.50
0.75
1.00
SNP effect on Periodontitis

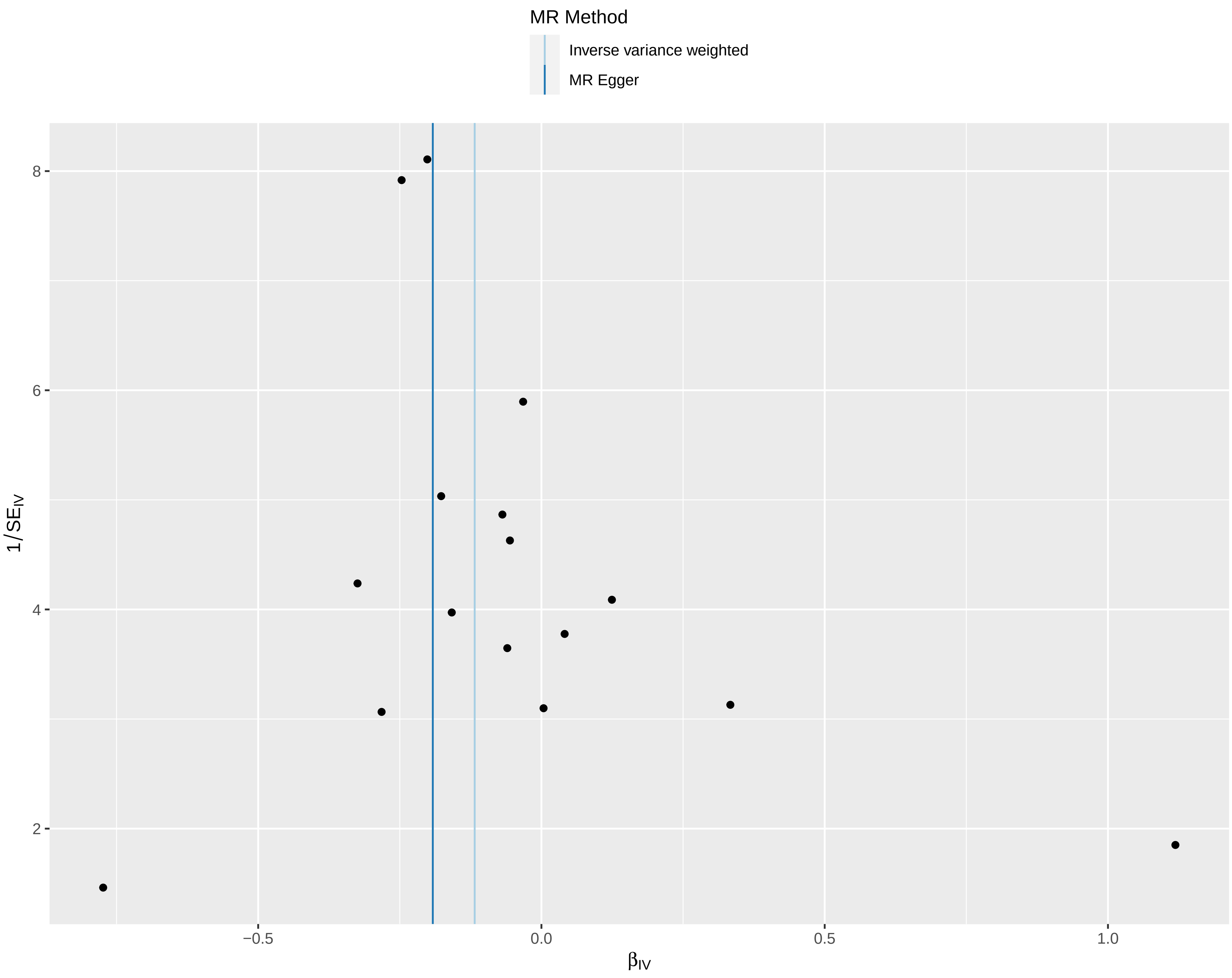

MR Method
Inverse variance weighted
MR Egger
8
6
4
2
1/SE_IV
−0.5
0.0
0.5
1.0
β_IV

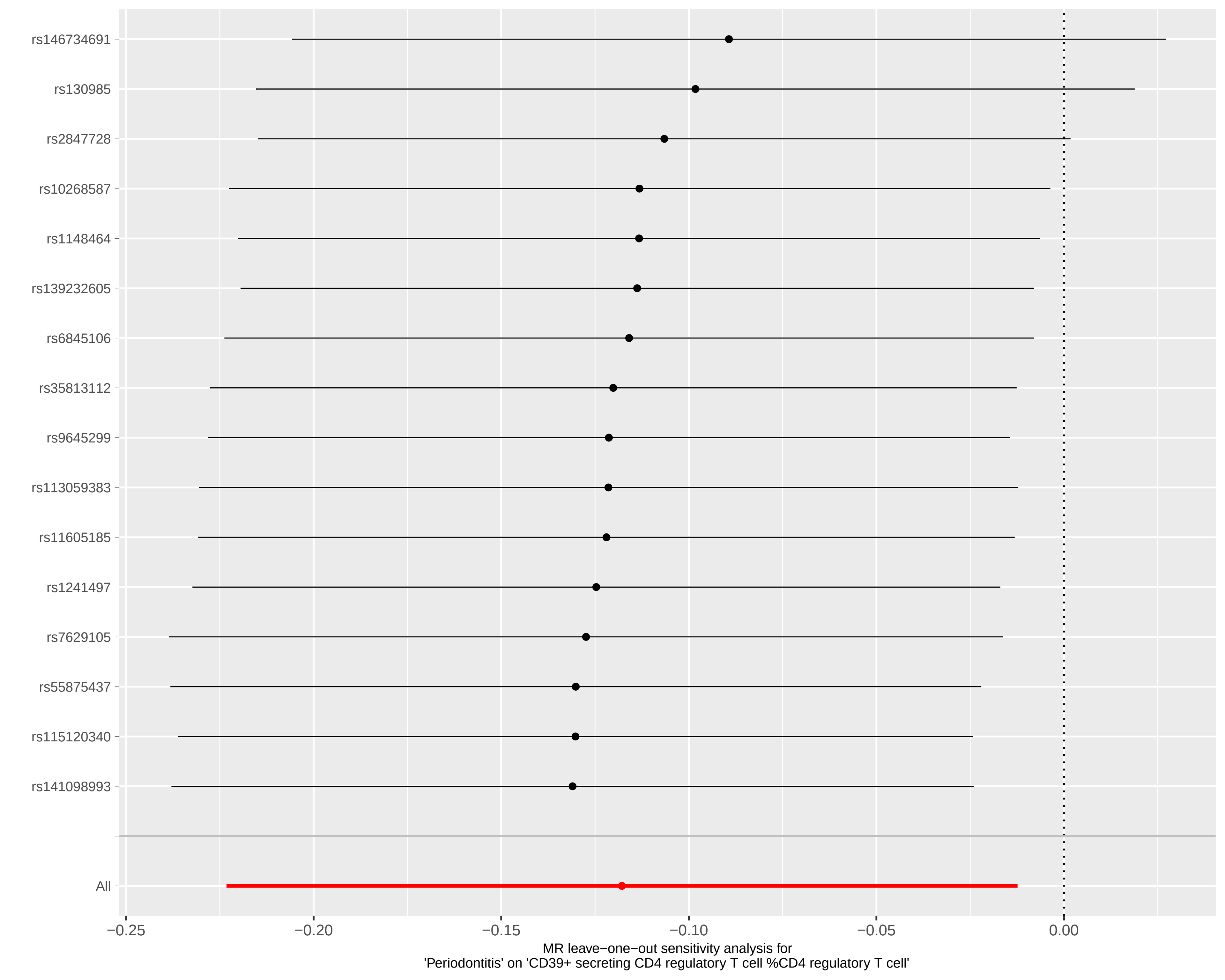
rs146734691
rs130985
rs2847728
rs10268587
rs1148464
rs139232605
rs6845106
rs35813112
rs9645299
rs113059383
rs11605185
rs1241497
rs7629105
rs55875437
rs115120340
rs141098993
All
−0.25
−0.20
−0.15
−0.10
−0.05
0.00
MR leave−one−out sensitivity analysis for
'Periodontitis' on 'CD39+ secreting CD4 regulatory T cell %CD4 regulatory T cell'

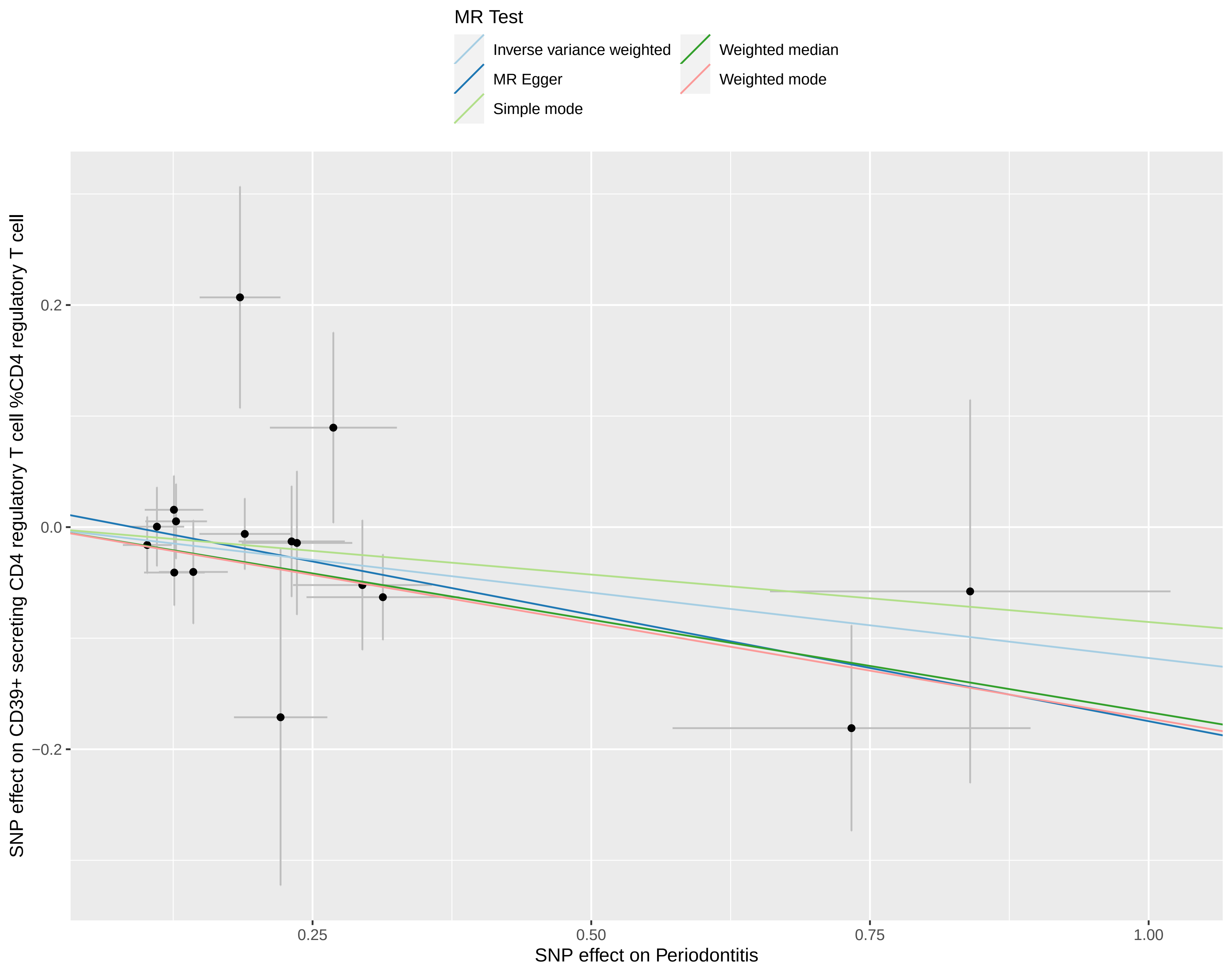

MR Test
Inverse variance weighted
MR Egger
Simple mode
Weighted median
Weighted mode
0.2
0.0
−0.2
0.25
0.50
0.75
1.00
SNP effect on Periodontitis
SNP effect on CD39+ secreting CD4 regulatory T cell %CD4 regulatory T cell

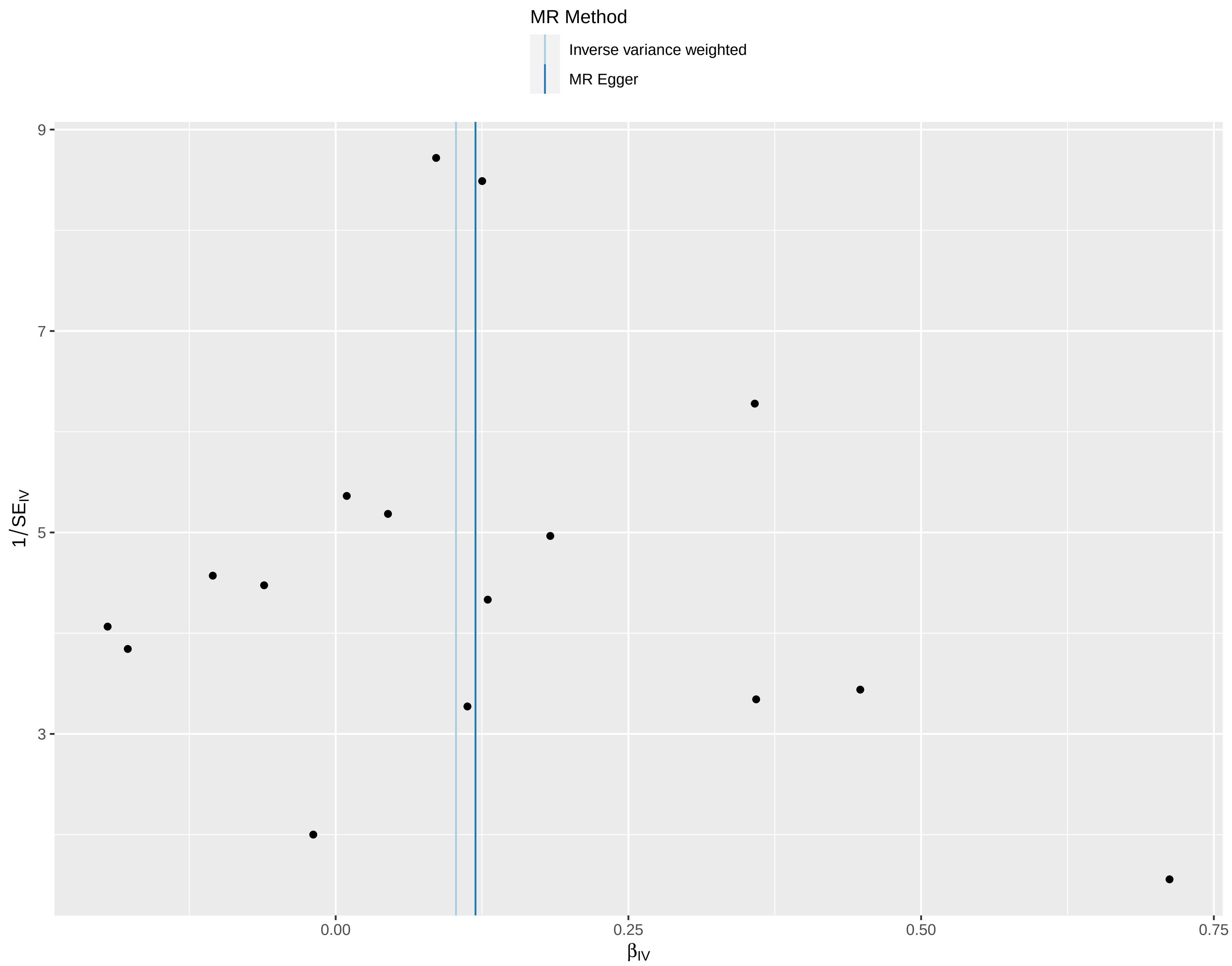
MR Method
Inverse variance weighted
MR Egger
9
7
5
3
1/SE_IV
0.00
0.25
0.50
0.75
β_IV

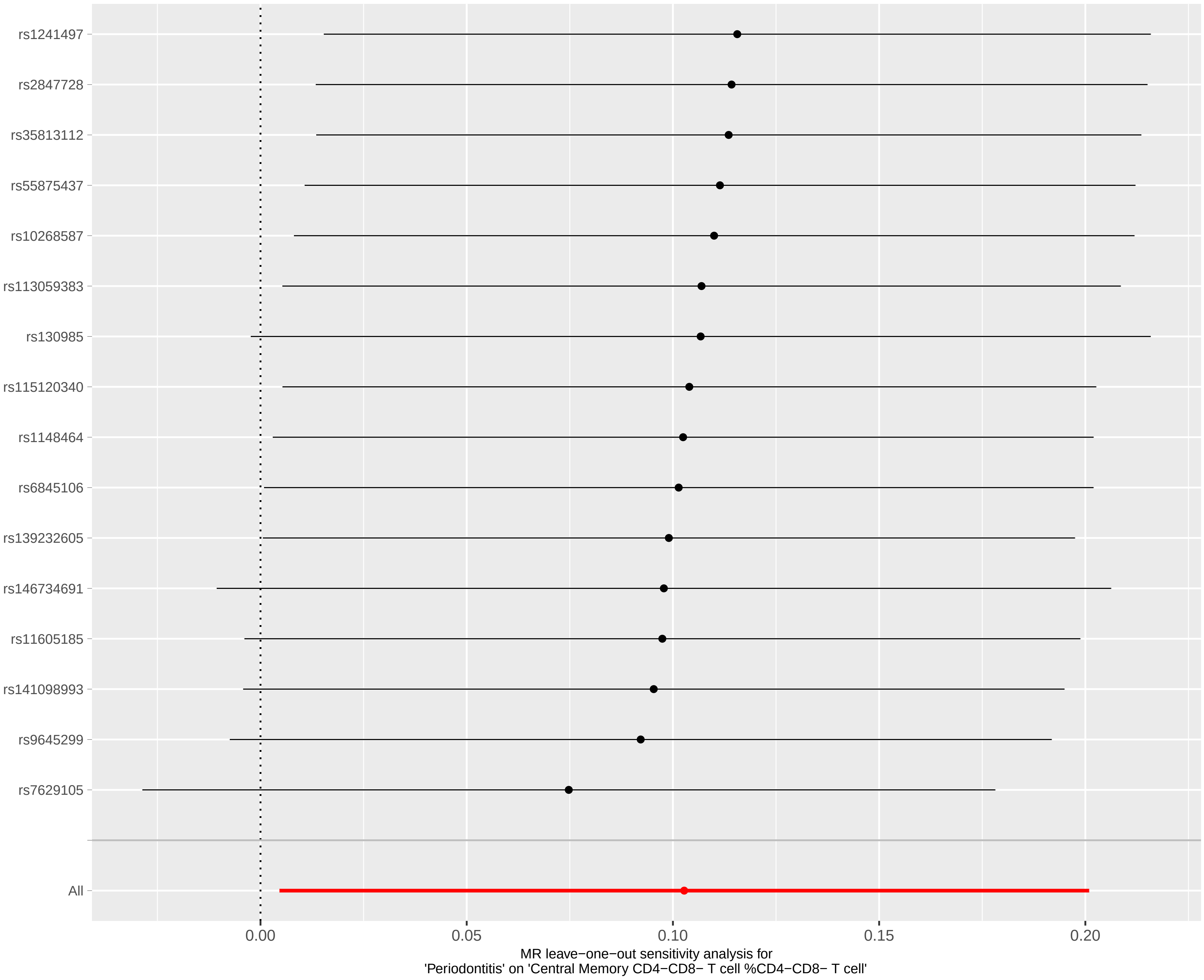

rs1241497
rs2847728
rs35813112
rs55875437
rs10268587
rs113059383
rs130985
rs115120340
rs1148464
rs6845106
rs139232605
rs146734691
rs11605185
rs141098993
rs9645299
rs7629105
All
0.00
0.05
0.10
0.15
0.20
MR leave−one−out sensitivity analysis for
'Periodontitis' on 'Central Memory CD4−CD8− T cell %CD4−CD8− T cell'

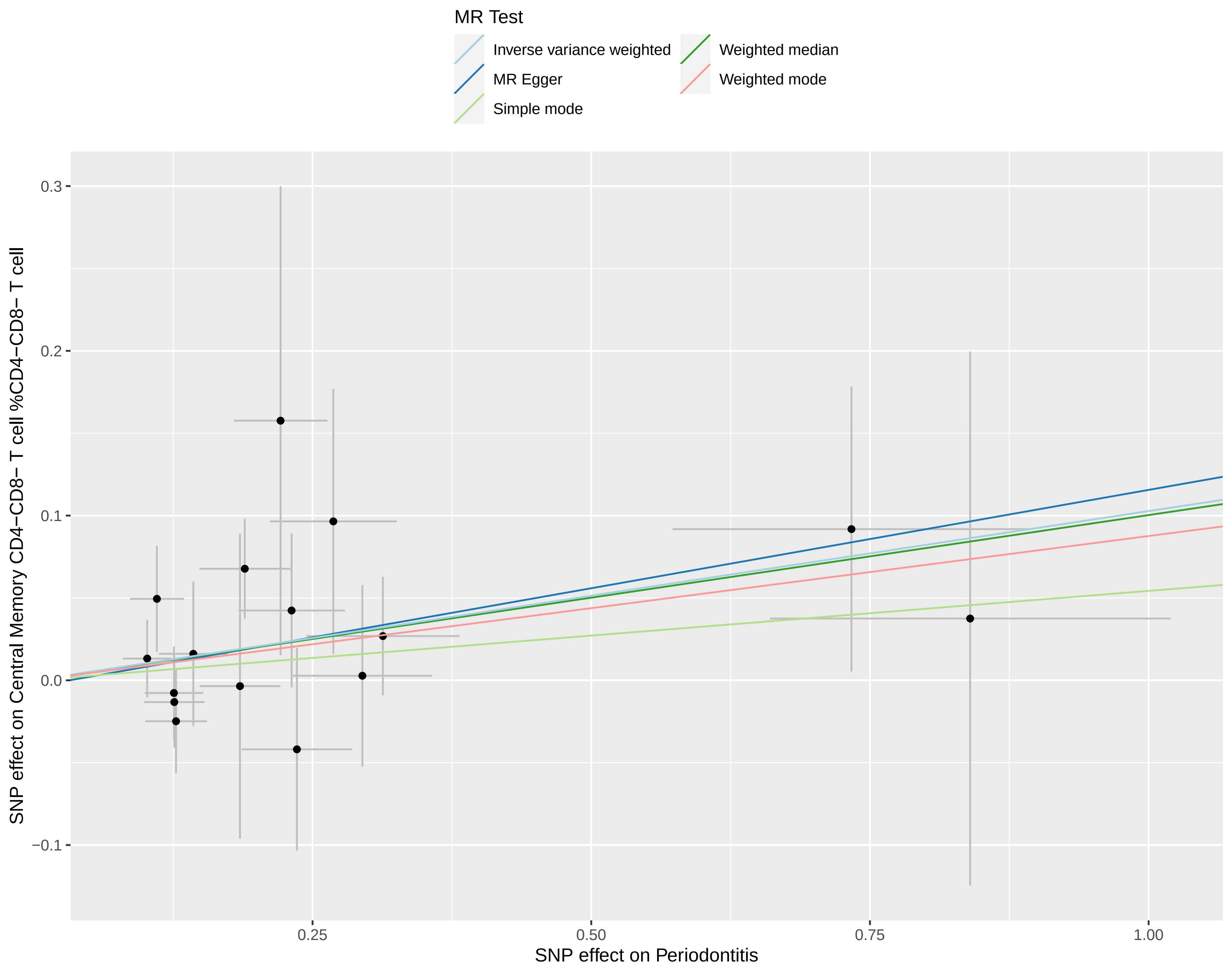

MR Test
Inverse variance weighted
MR Egger
Simple mode
Weighted median
Weighted mode
SNP effect on Central Memory CD4−CD8− T cell %CD4−CD8− T cell
0.3
0.2
0.1
0.0
−0.1
0.25
0.50
0.75
1.00
SNP effect on Periodontitis

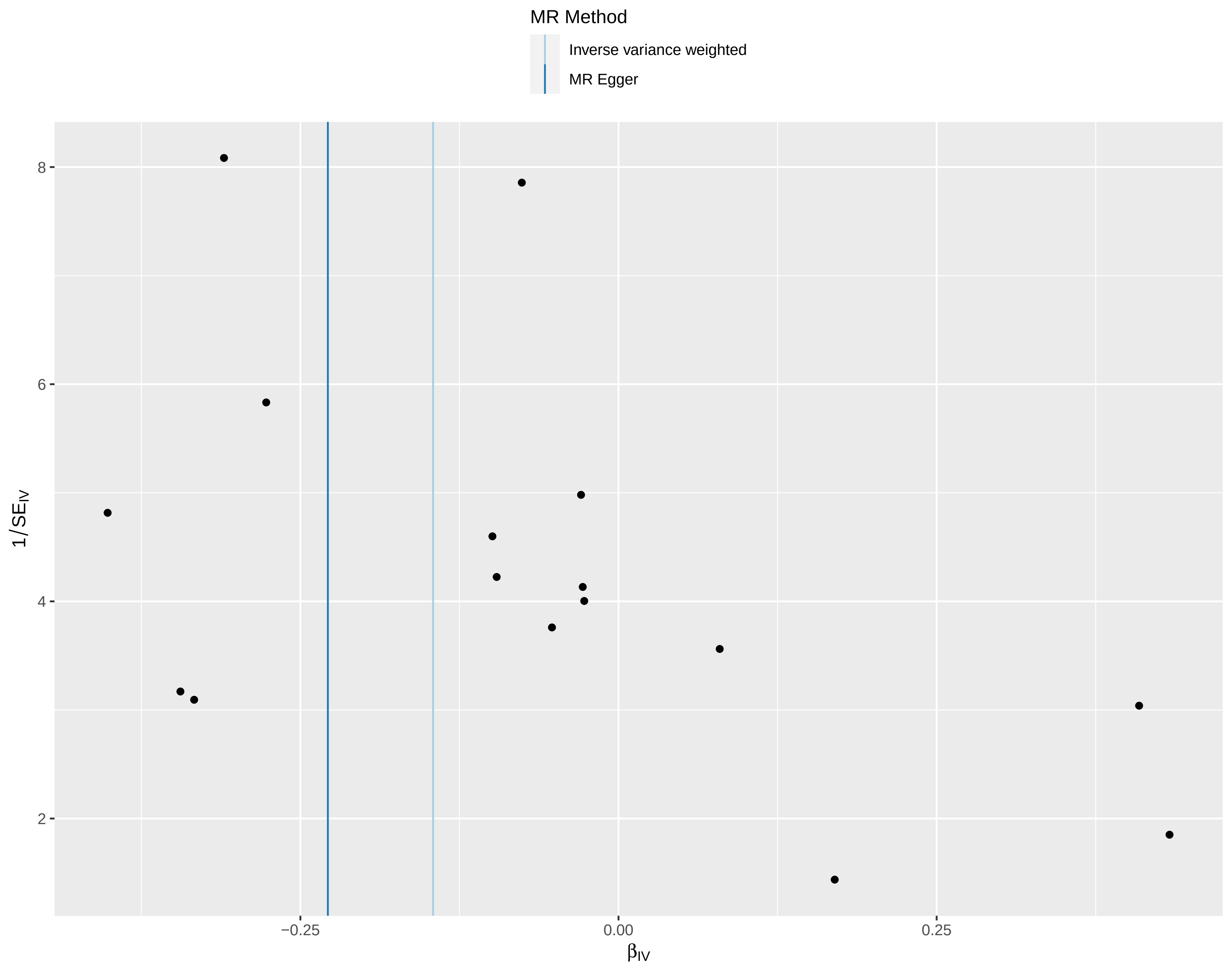
MR Method
Inverse variance weighted
MR Egger
8
6
4
2
1/SE_IV
−0.25
0.00
0.25
β_IV

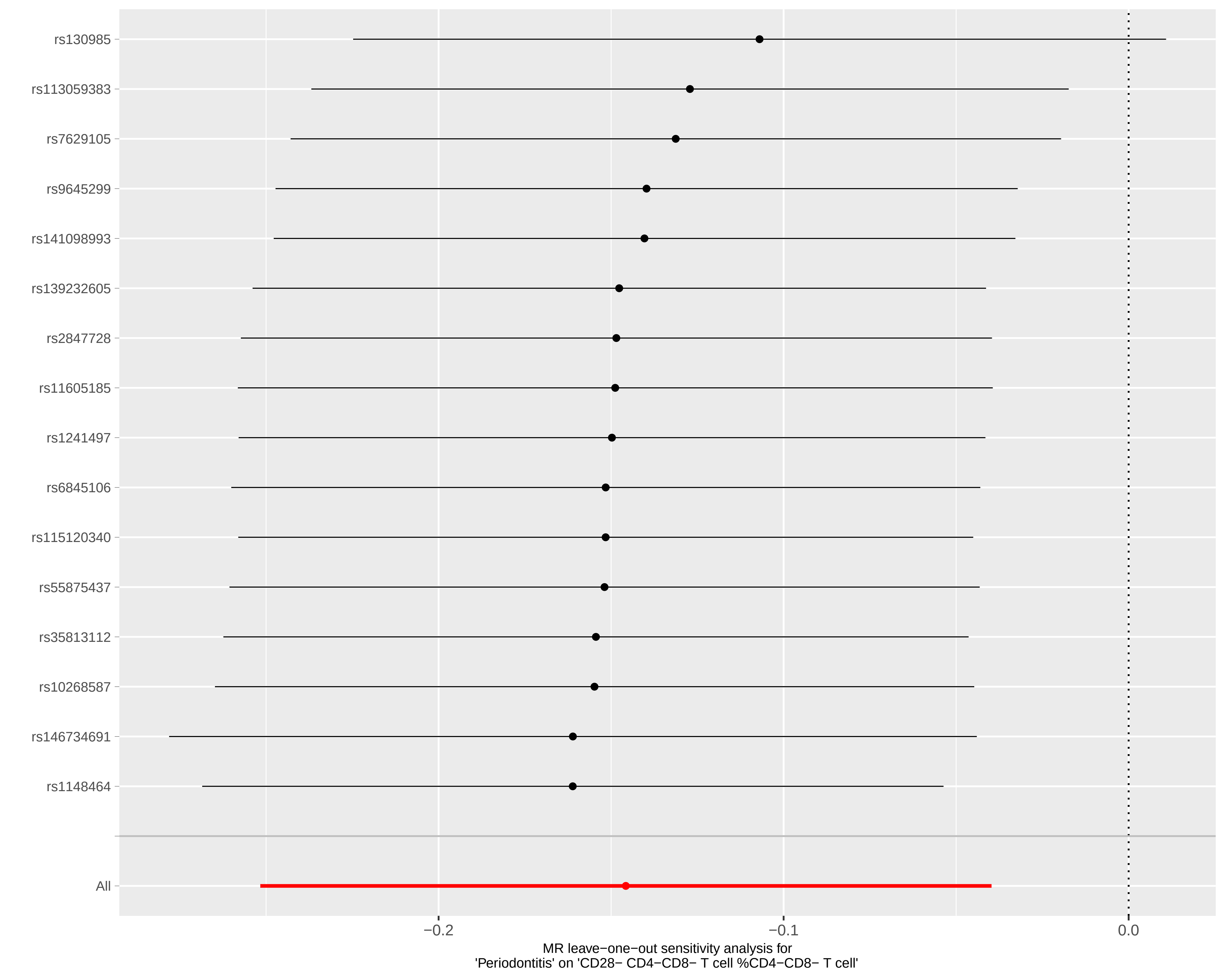
rs130985
rs113059383
rs7629105
rs9645299
rs141098993
rs139232605
rs2847728
rs11605185
rs1241497
rs6845106
rs115120340
rs55875437
rs35813112
rs10268587
rs146734691
rs1148464
All
−0.2
−0.1
0.0
MR leave−one−out sensitivity analysis for
'Periodontitis' on 'CD28− CD4−CD8− T cell %CD4−CD8− T cell'

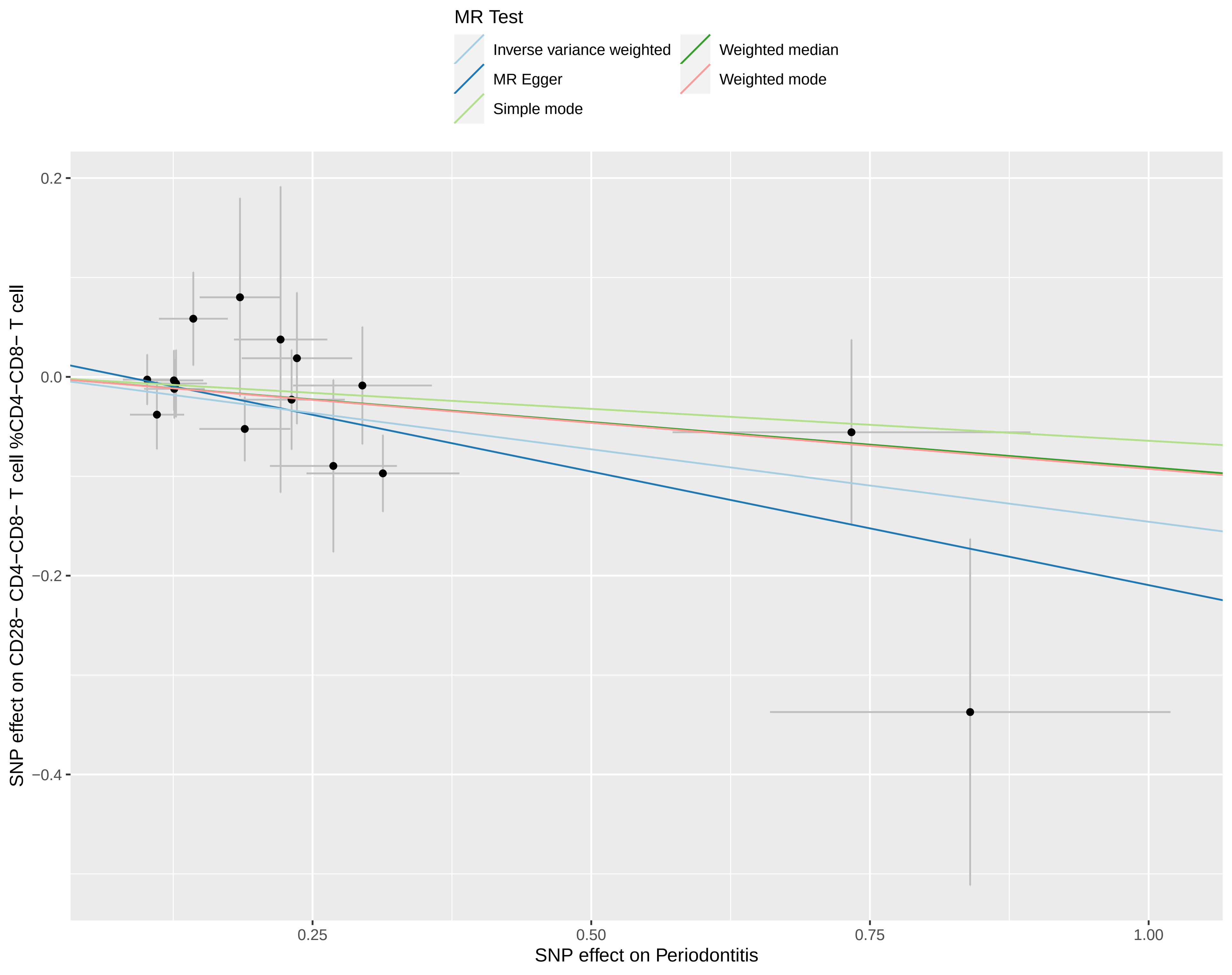

MR Test
Inverse variance weighted
MR Egger
Simple mode
Weighted median
Weighted mode
0.2
0.0
−0.2
−0.4
0.25
0.50
0.75
1.00
SNP effect on CD28− CD4−CD8− T cell %CD4−CD8− T cell
SNP effect on Periodontitis

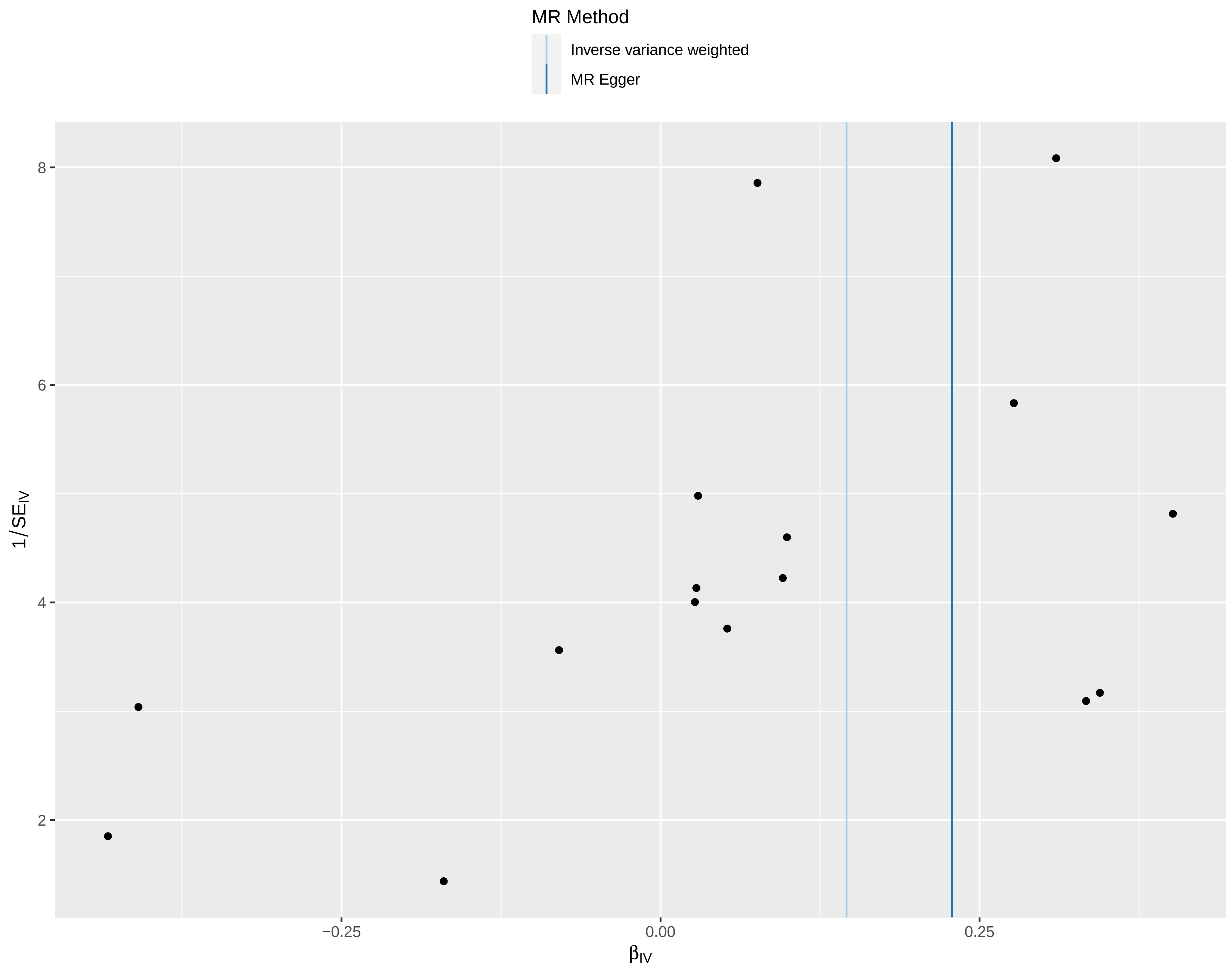
MR Method
Inverse variance weighted
MR Egger
8
6
4
2
1/SE$_{IV}$
−0.25
0.00
0.25
β$_{IV}$

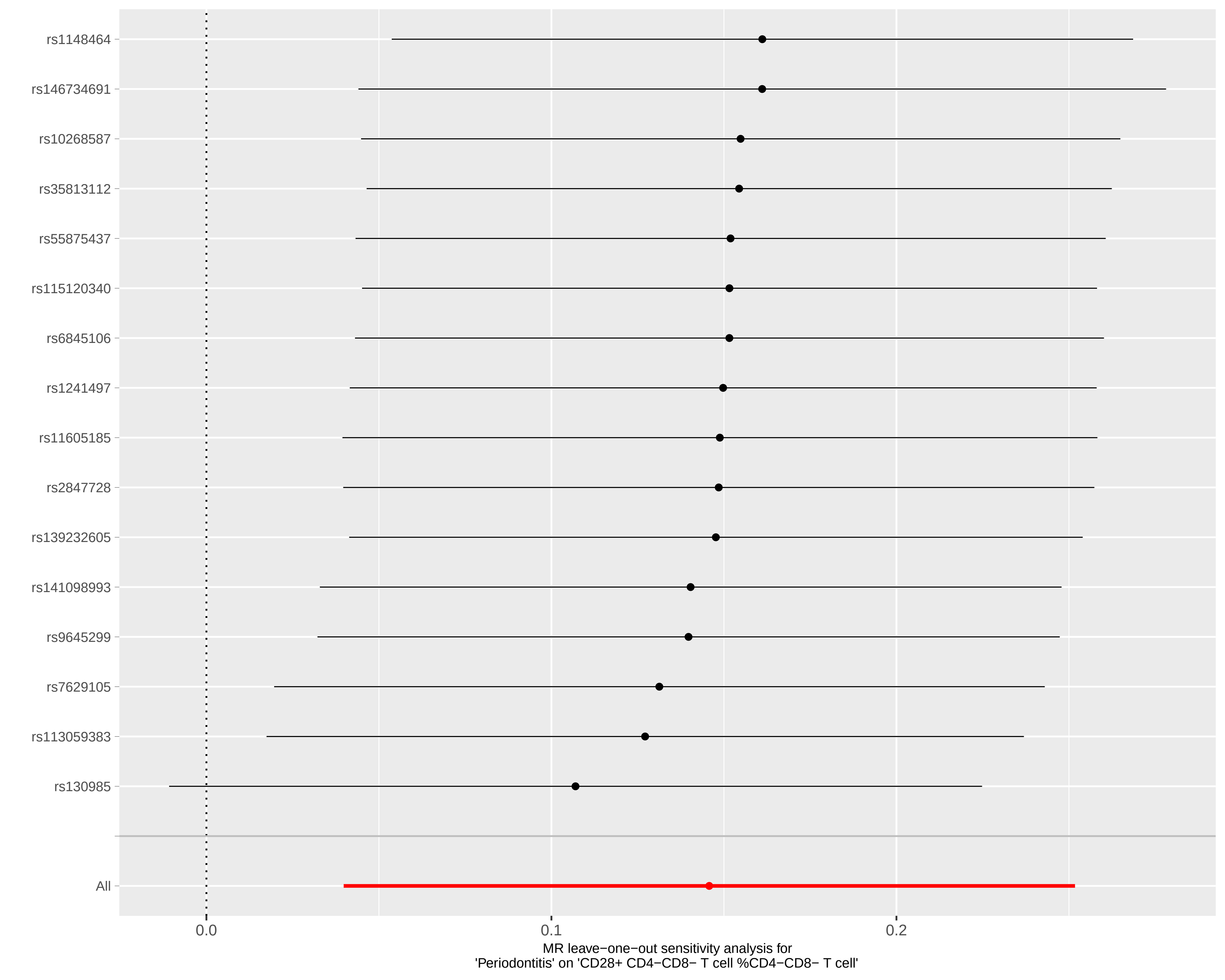

rs1148464
rs146734691
rs10268587
rs35813112
rs55875437
rs115120340
rs6845106
rs1241497
rs11605185
rs2847728
rs139232605
rs141098993
rs9645299
rs7629105
rs113059383
rs130985
All
0.0
0.1
0.2
MR leave−one−out sensitivity analysis for
'Periodontitis' on 'CD28+ CD4−CD8− T cell %CD4−CD8− T cell'

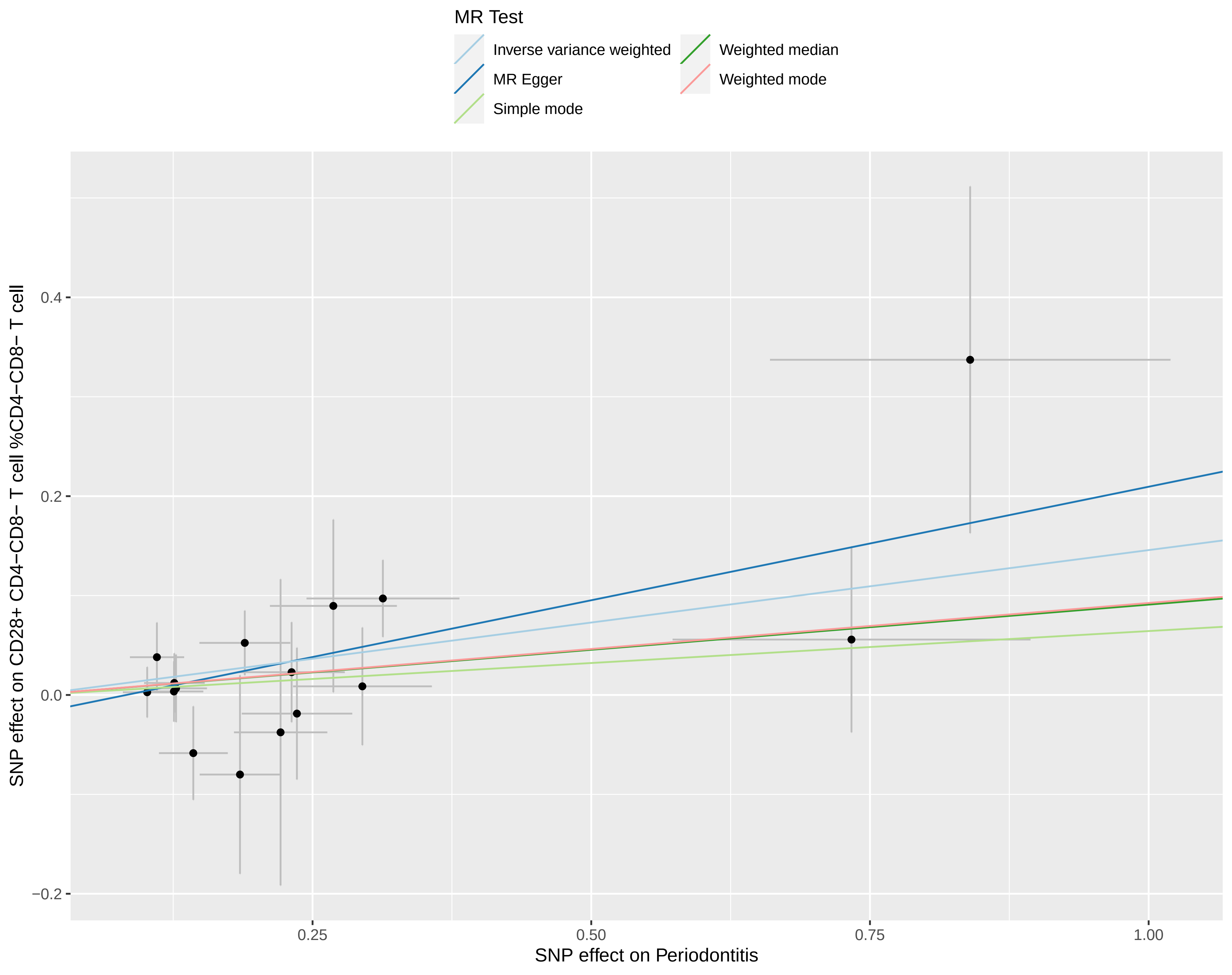

MR Test
Inverse variance weighted
MR Egger
Simple mode
Weighted median
Weighted mode
0.4
0.2
0.0
−0.2
0.25
0.50
0.75
1.00
SNP effect on Periodontitis
SNP effect on CD28+ CD4−CD8− T cell %CD4−CD8− T cell

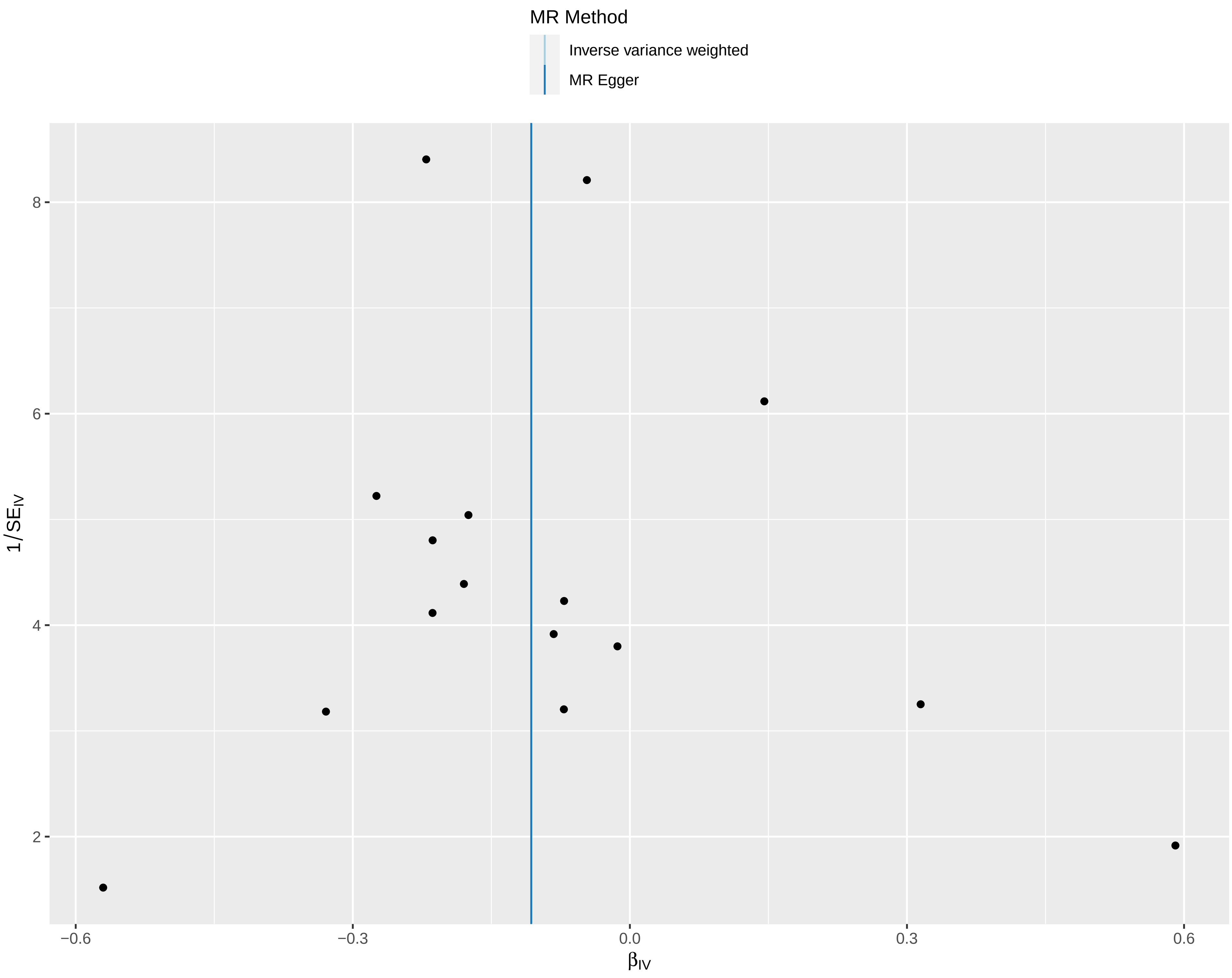
MR Method
Inverse variance weighted
MR Egger
8
6
4
2
1/SE_IV
−0.6
−0.3
0.0
0.3
0.6
β_IV

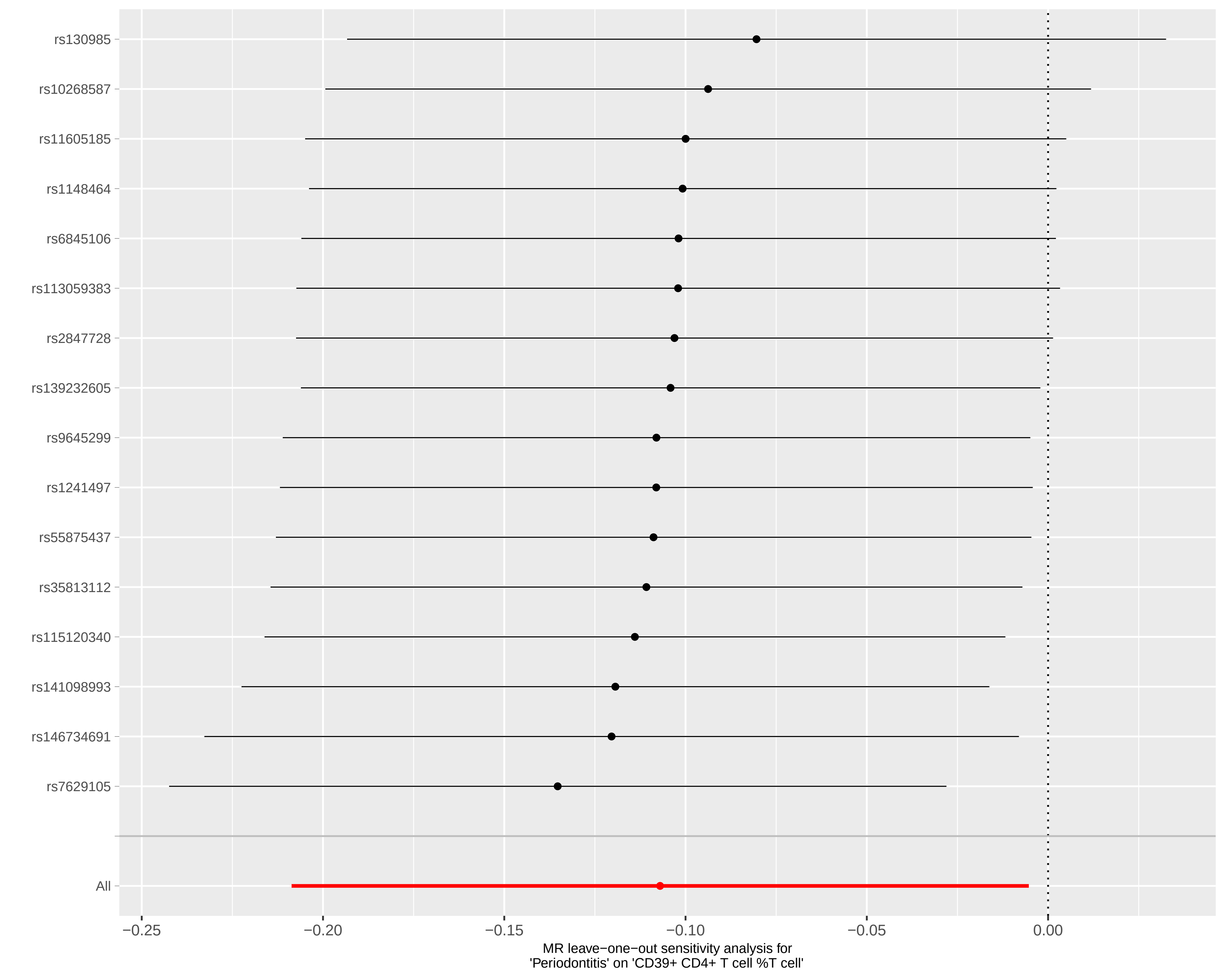
rs130985
rs10268587
rs11605185
rs1148464
rs6845106
rs113059383
rs2847728
rs139232605
rs9645299
rs1241497
rs55875437
rs35813112
rs115120340
rs141098993
rs146734691
rs7629105
All
−0.25
−0.20
−0.15
−0.10
−0.05
0.00
MR leave−one−out sensitivity analysis for
'Periodontitis' on 'CD39+ CD4+ T cell %T cell'

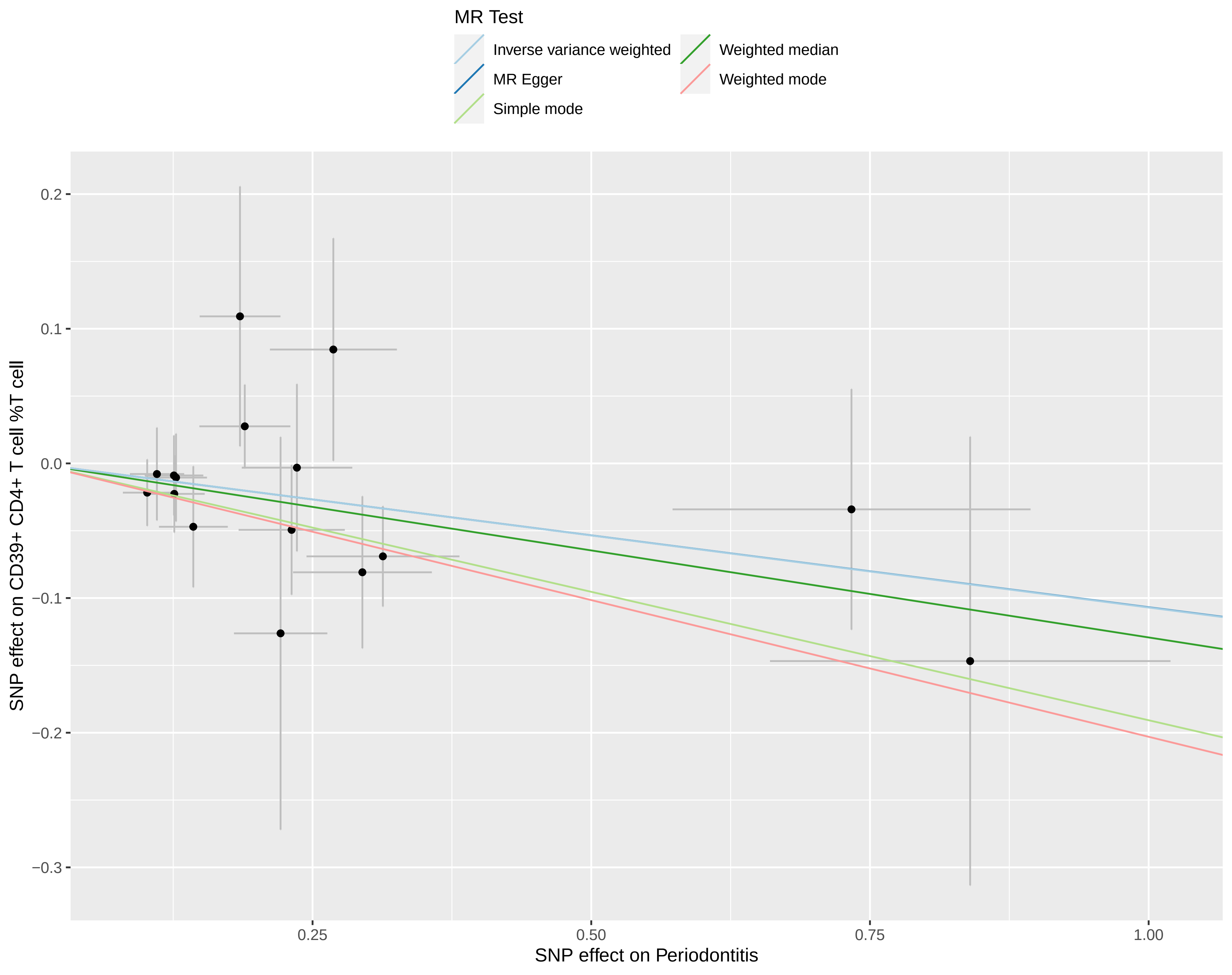

MR Test
Inverse variance weighted
MR Egger
Simple mode
Weighted median
Weighted mode
0.2
0.1
0.0
−0.1
−0.2
−0.3
SNP effect on CD39+ CD4+ T cell %T cell
0.25
0.50
0.75
1.00
SNP effect on Periodontitis

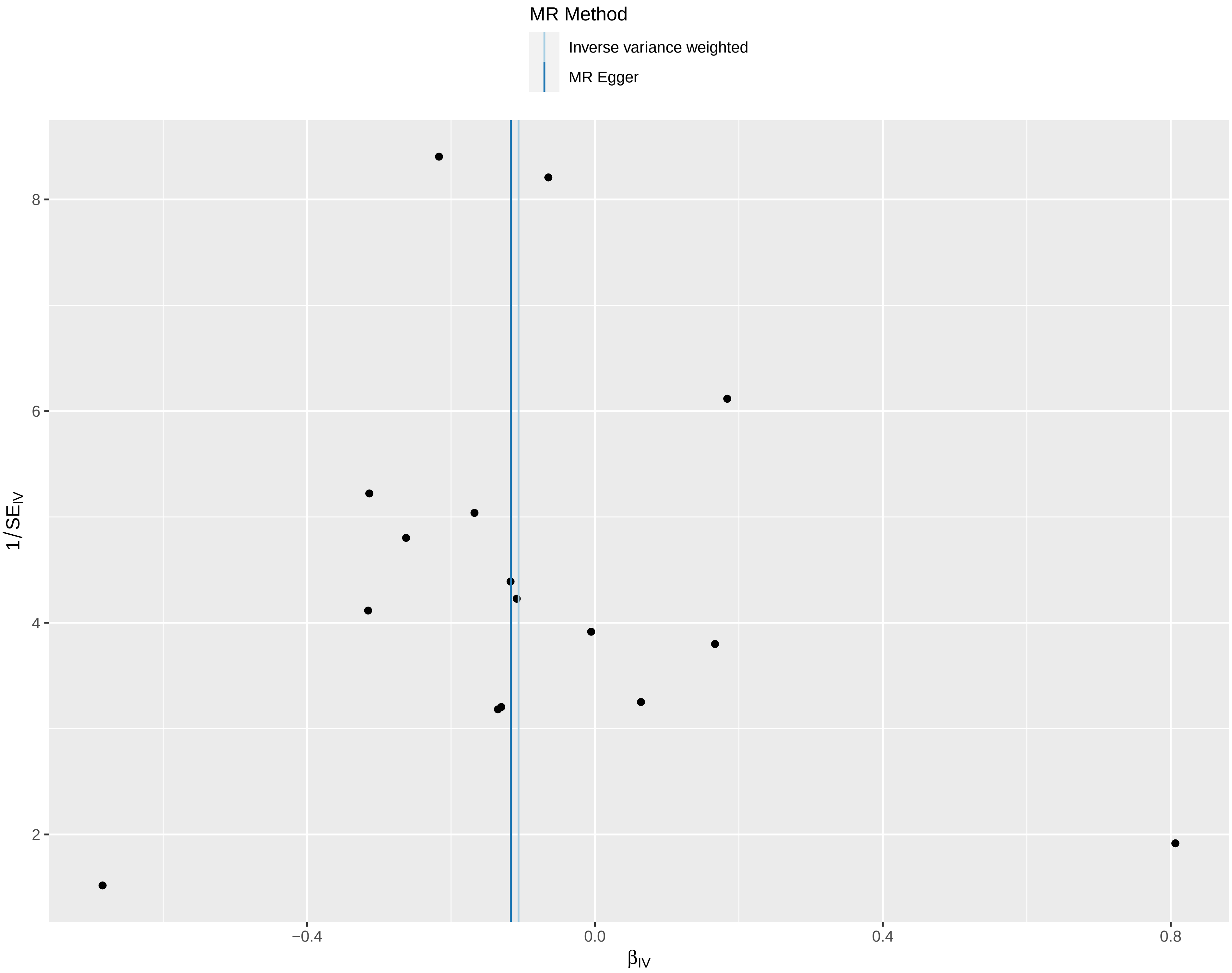
MR Method
Inverse variance weighted
MR Egger
1/SE_IV
β_IV
8
6
4
2
−0.4
0.0
0.4
0.8

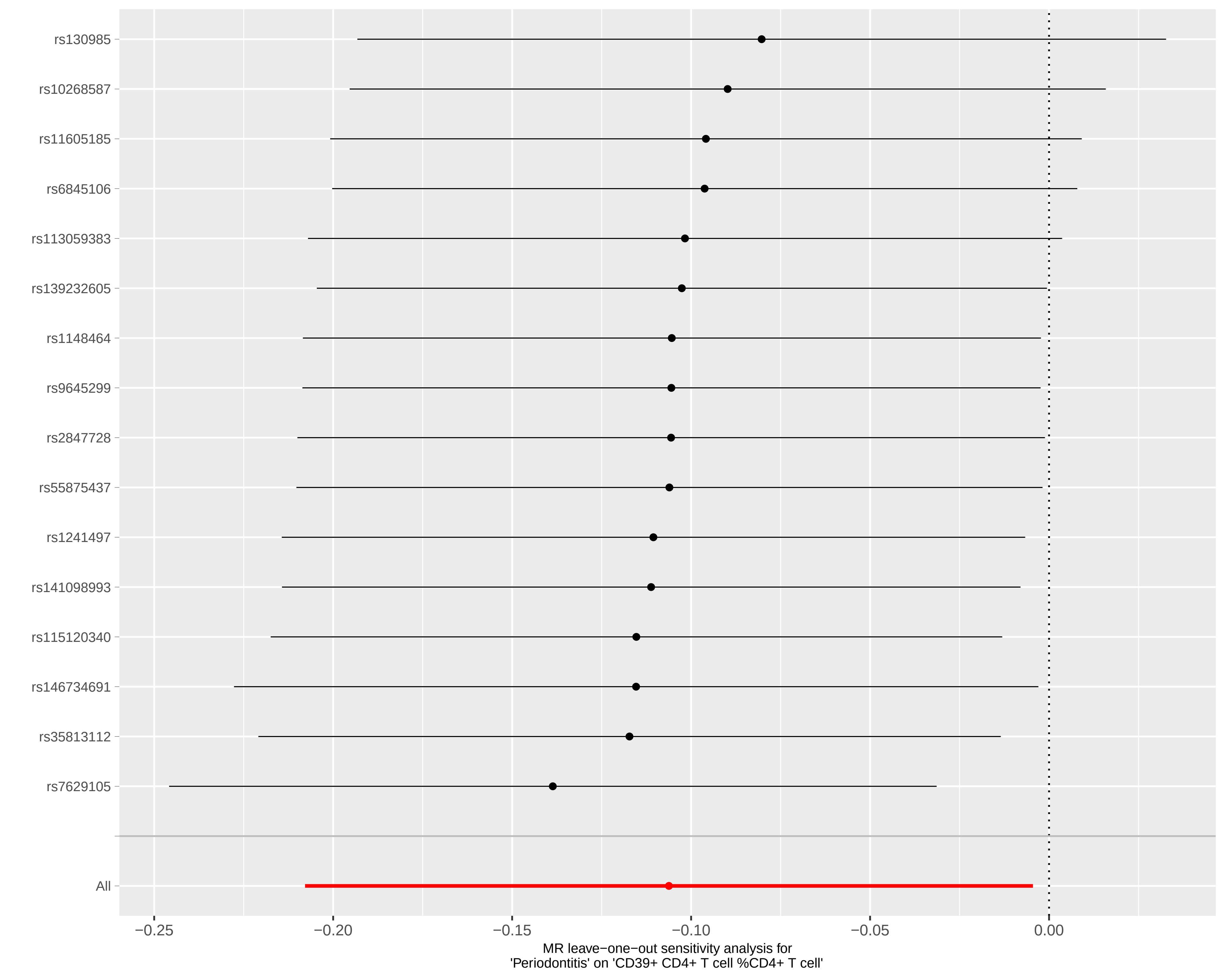

rs130985
rs10268587
rs11605185
rs6845106
rs113059383
rs139232605
rs1148464
rs9645299
rs2847728
rs55875437
rs1241497
rs141098993
rs115120340
rs146734691
rs35813112
rs7629105
All
−0.25
−0.20
−0.15
−0.10
−0.05
0.00
MR leave−one−out sensitivity analysis for
'Periodontitis' on 'CD39+ CD4+ T cell %CD4+ T cell'

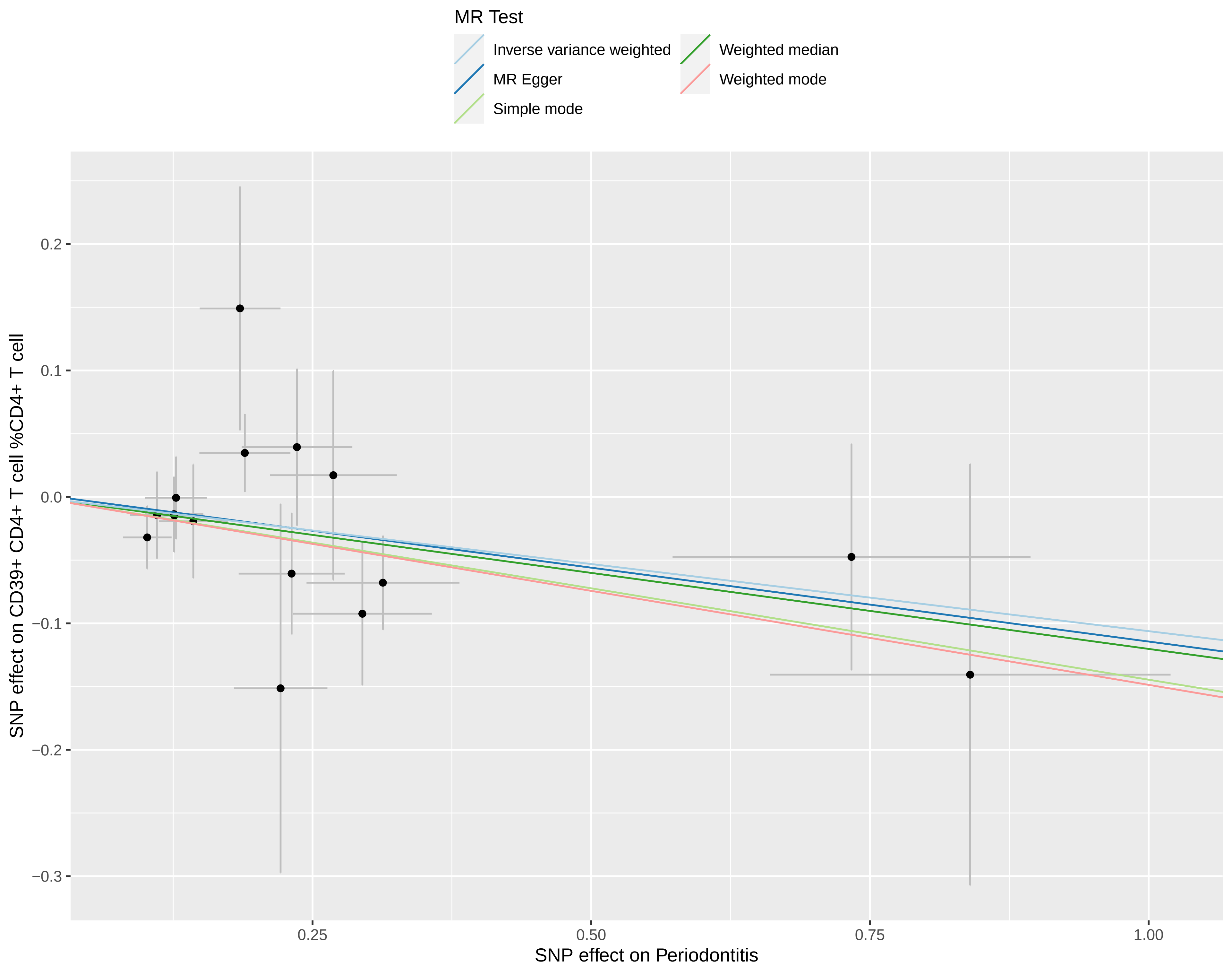
MR Test
Inverse variance weighted
MR Egger
Simple mode
Weighted median
Weighted mode
SNP effect on CD39+ CD4+ T cell %CD4+ T cell
0.2
0.1
0.0
−0.1
−0.2
−0.3
0.25
0.50
0.75
1.00
SNP effect on Periodontitis

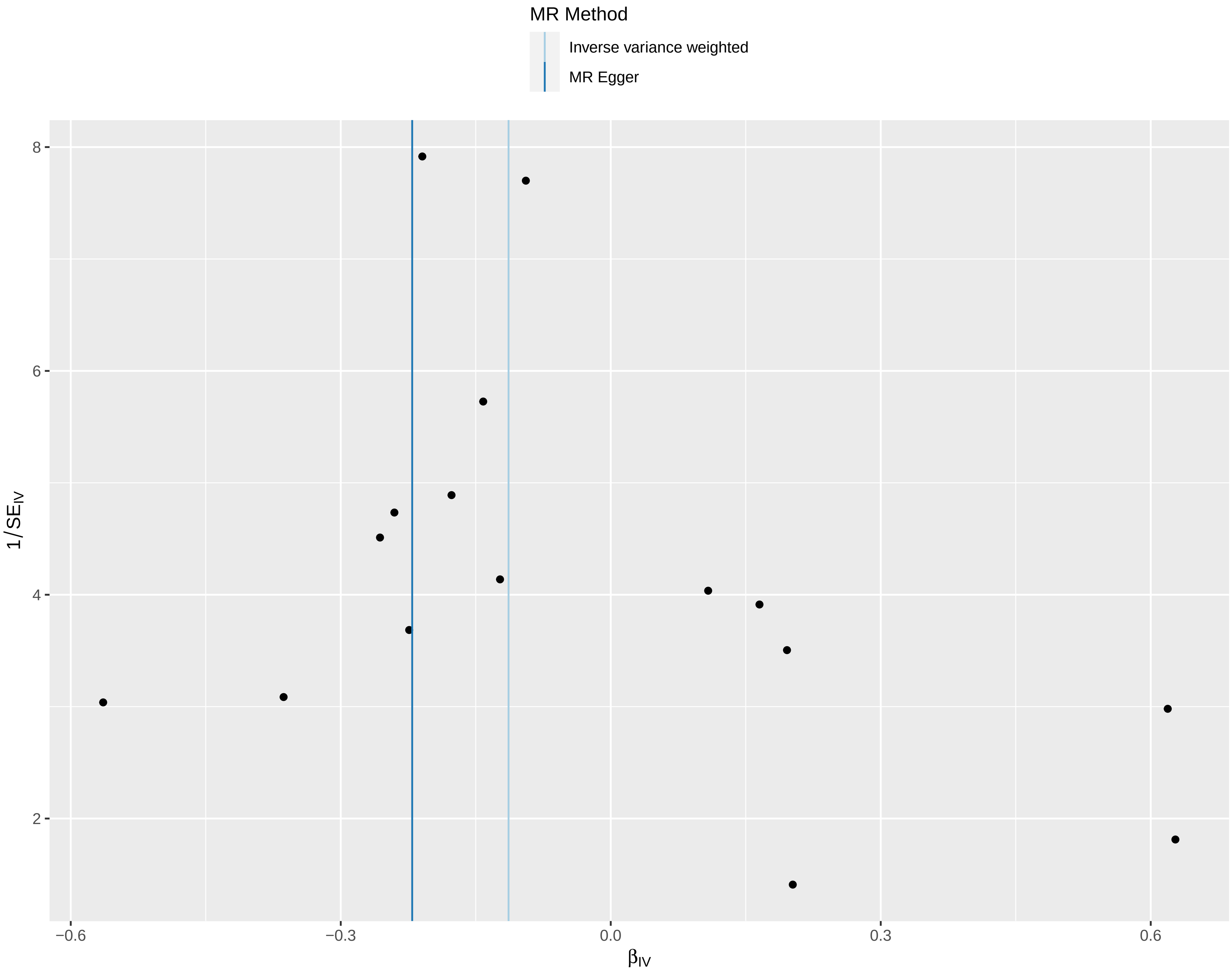
MR Method
Inverse variance weighted
MR Egger
1/SE_IV
8
6
4
2
−0.6
−0.3
0.0
0.3
0.6
β_IV

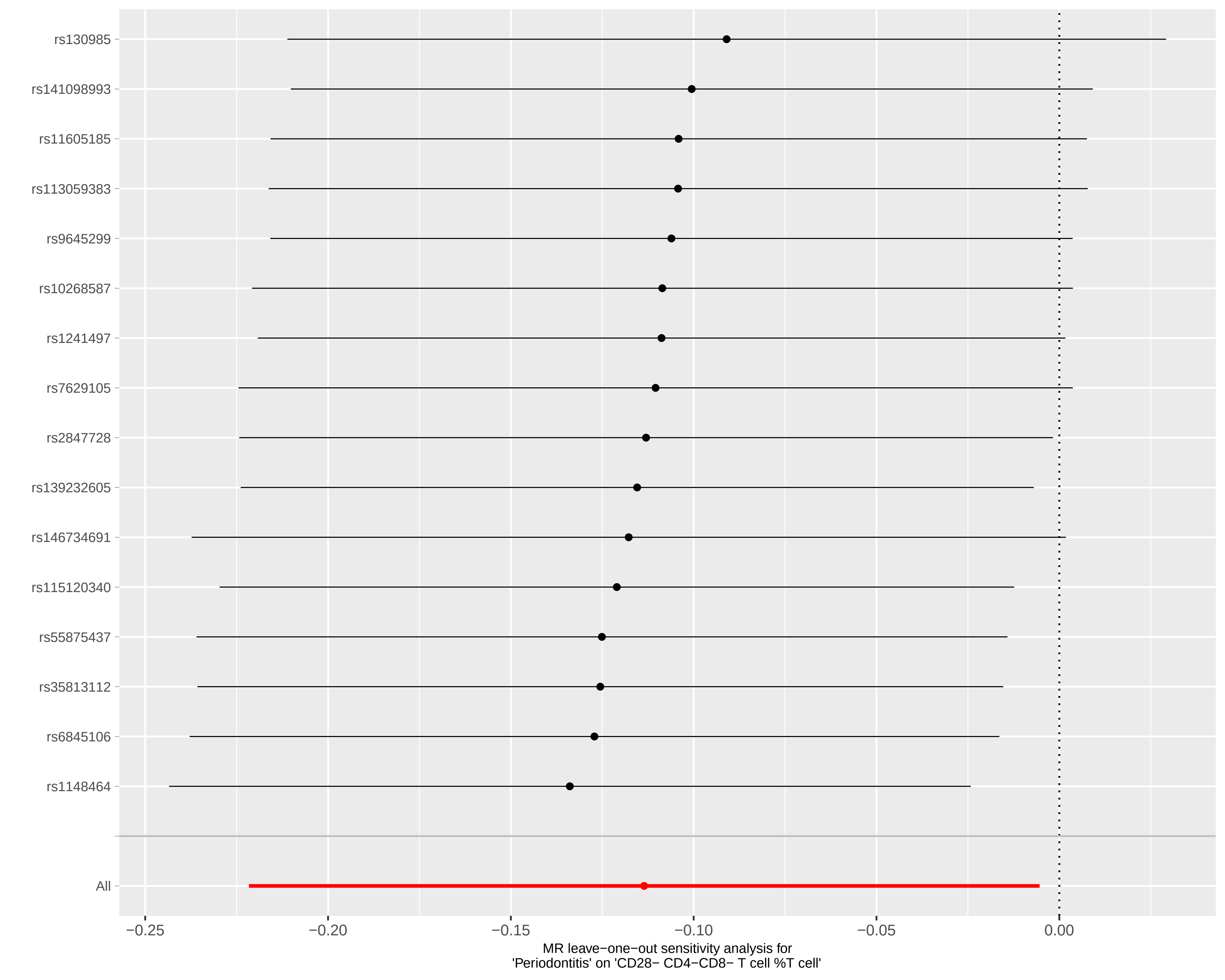
rs130985
rs141098993
rs11605185
rs113059383
rs9645299
rs10268587
rs1241497
rs7629105
rs2847728
rs139232605
rs146734691
rs115120340
rs55875437
rs35813112
rs6845106
rs1148464
All
−0.25
−0.20
−0.15
−0.10
−0.05
0.00
MR leave−one−out sensitivity analysis for
'Periodontitis' on 'CD28− CD4−CD8− T cell %T cell'

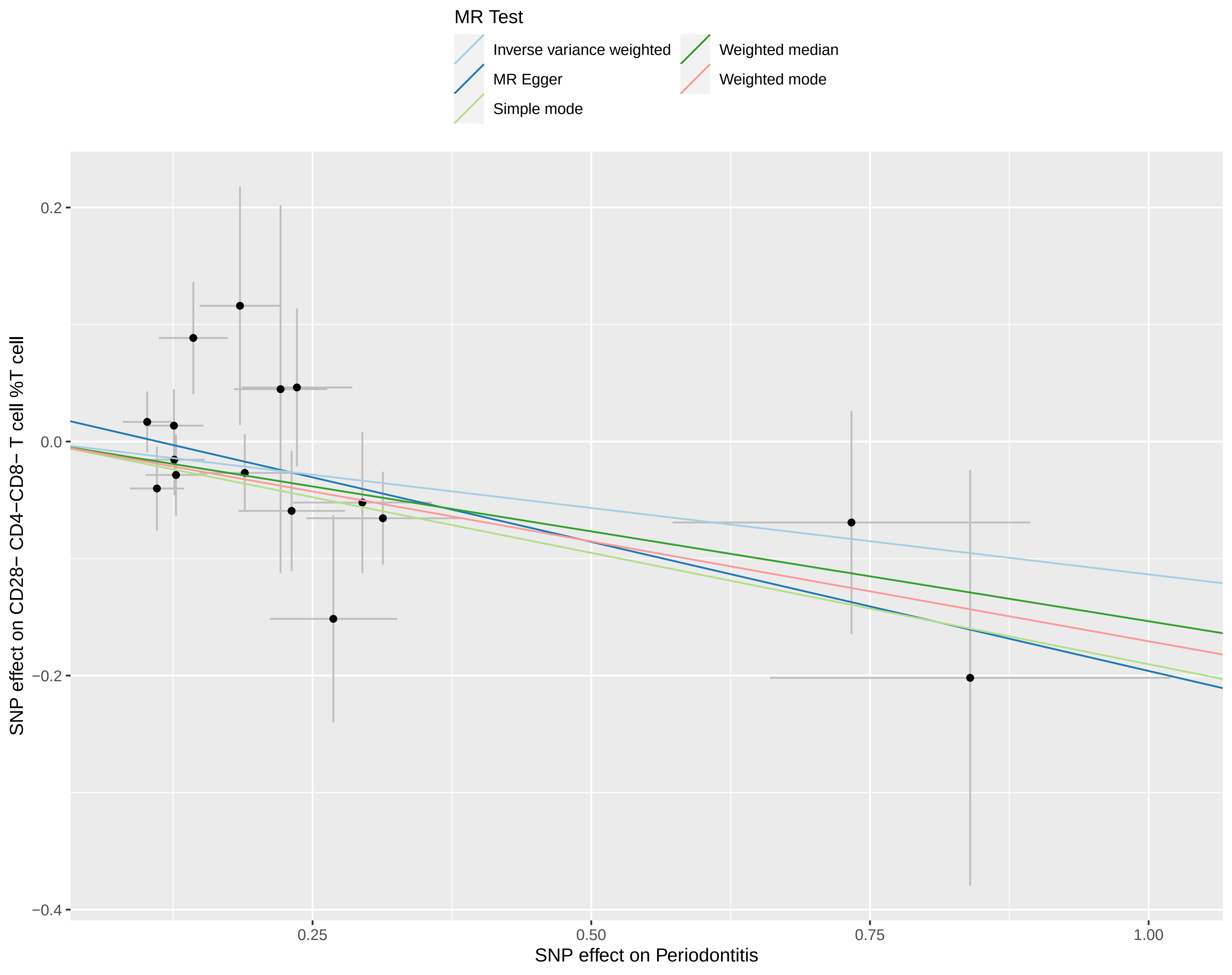

MR Test
Inverse variance weighted
MR Egger
Simple mode
Weighted median
Weighted mode
0.2
0.0
−0.2
−0.4
0.25
0.50
0.75
1.00
SNP effect on CD28− CD4−CD8− T cell %T cell
SNP effect on Periodontitis

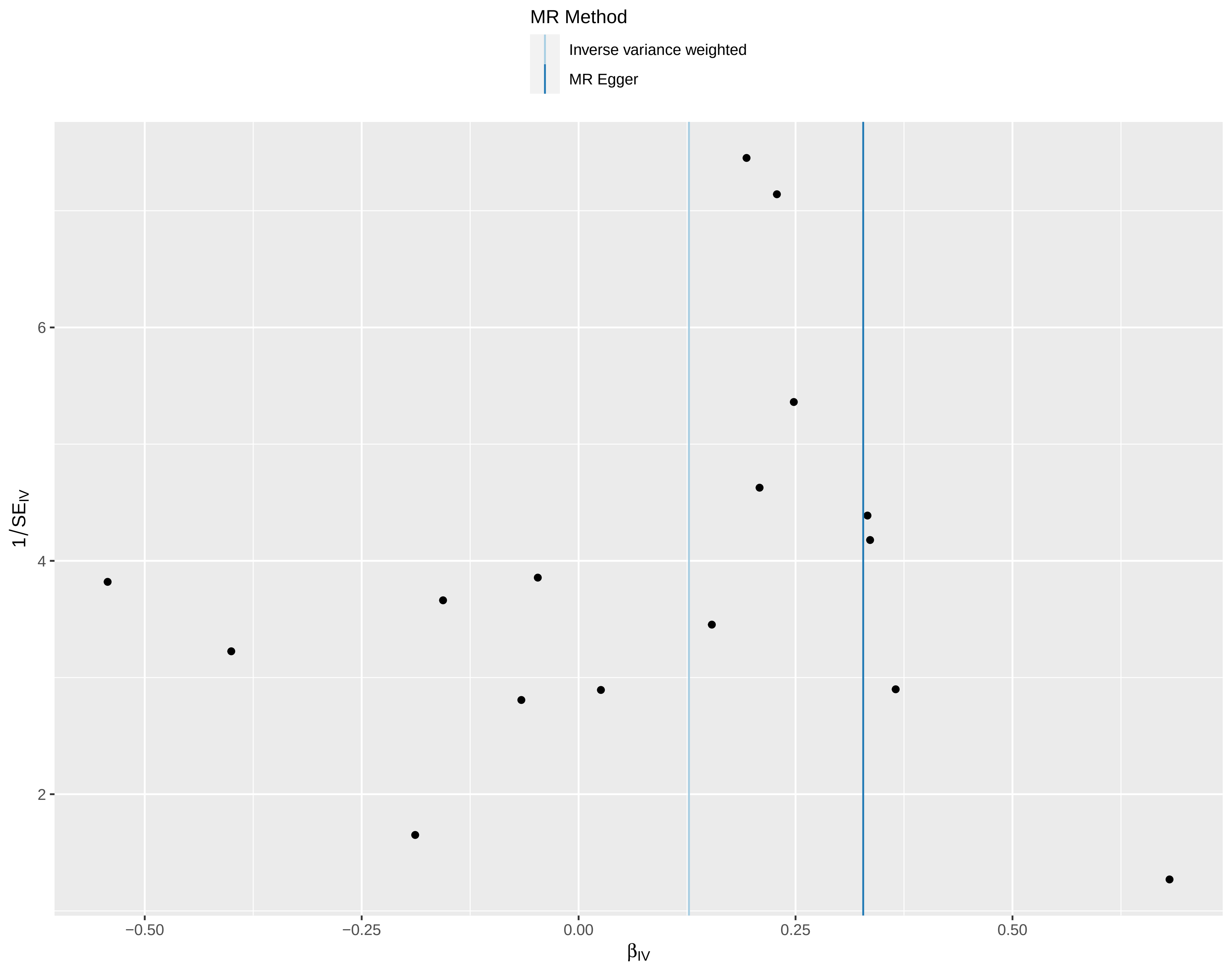
MR Method
Inverse variance weighted
MR Egger
6
4
2
1/SE$_{IV}$
−0.50
−0.25
0.00
0.25
0.50
β$_{IV}$

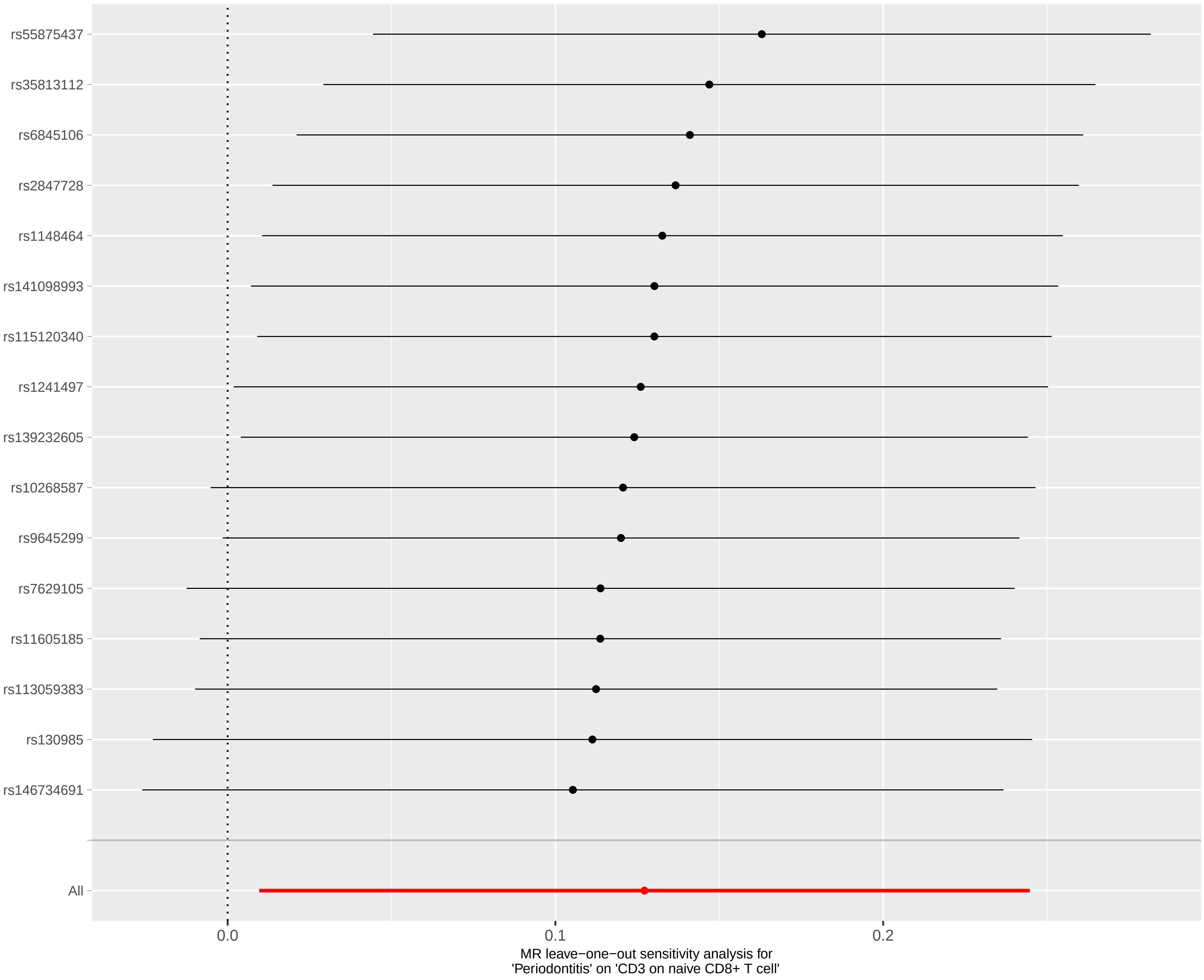

rs55875437
rs35813112
rs6845106
rs2847728
rs1148464
rs141098993
rs115120340
rs1241497
rs139232605
rs10268587
rs9645299
rs7629105
rs11605185
rs113059383
rs130985
rs146734691
All
0.0
0.1
0.2
MR leave−one−out sensitivity analysis for
'Periodontitis' on 'CD3 on naive CD8+ T cell'

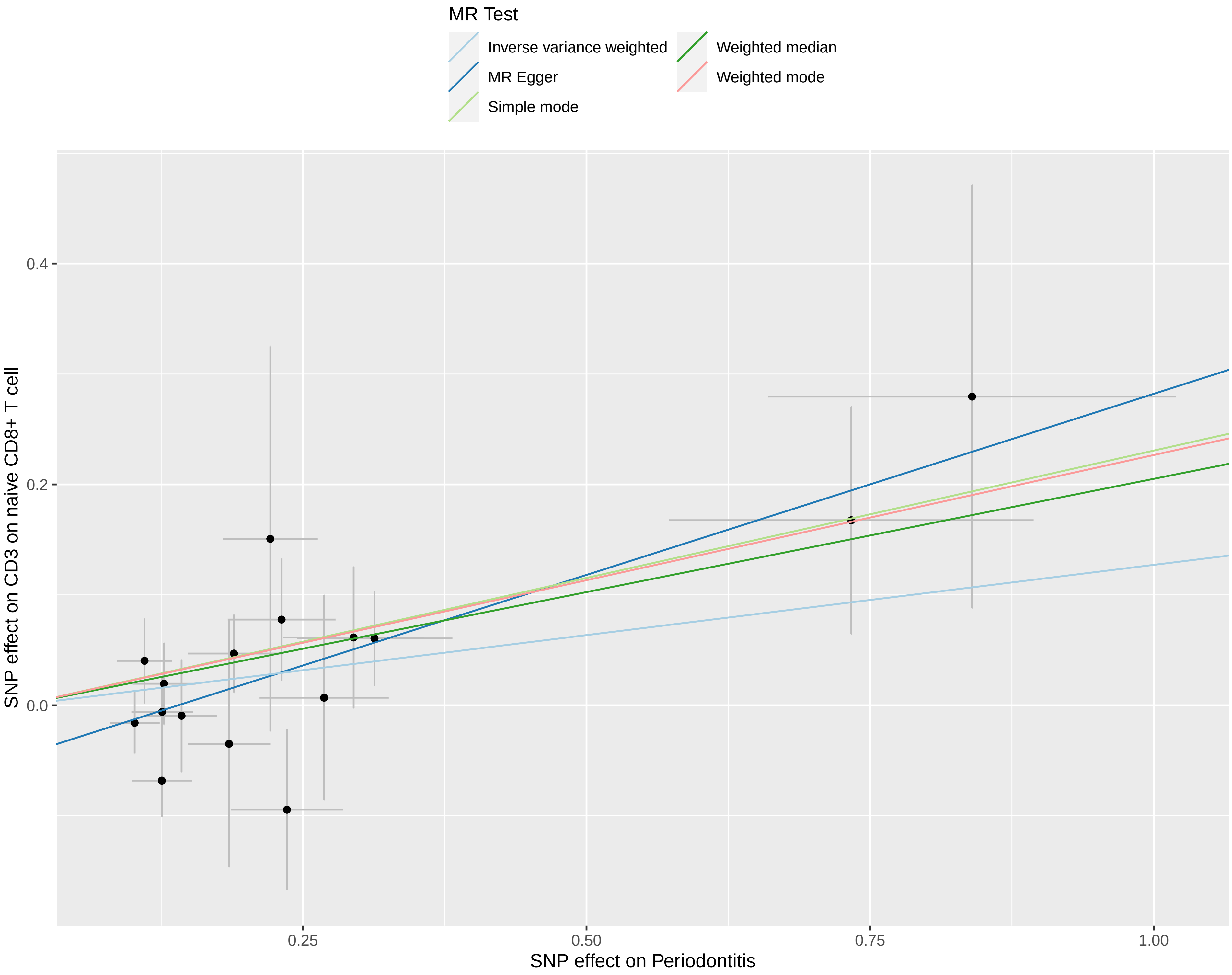
MR Test
Inverse variance weighted
MR Egger
Simple mode
Weighted median
Weighted mode
SNP effect on CD3 on naive CD8+ T cell
0.4
0.2
0.0
0.25
0.50
0.75
1.00
SNP effect on Periodontitis

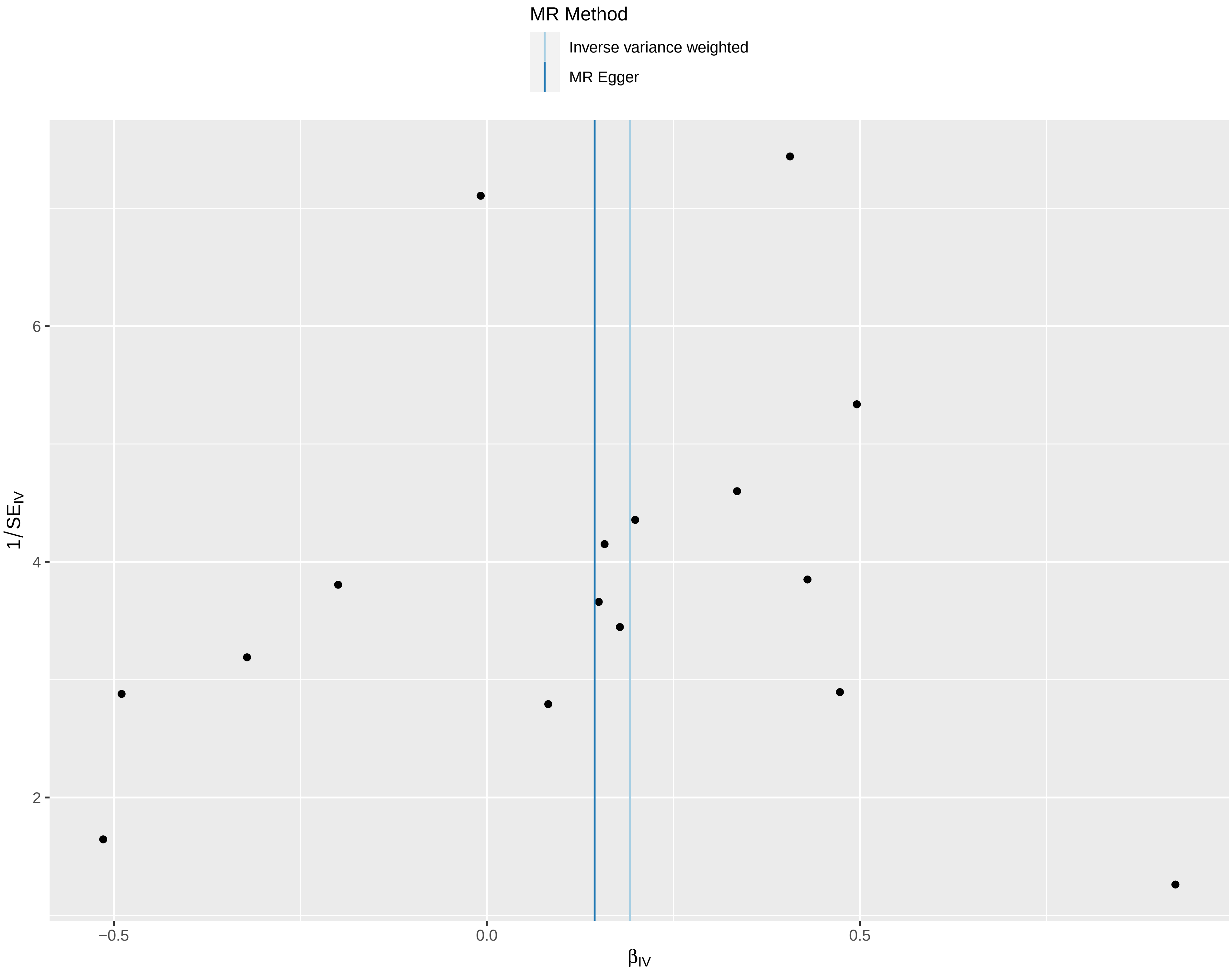

MR Method
Inverse variance weighted
MR Egger
1/SE$_{IV}$
6
4
2
−0.5
0.0
0.5
β$_{IV}$

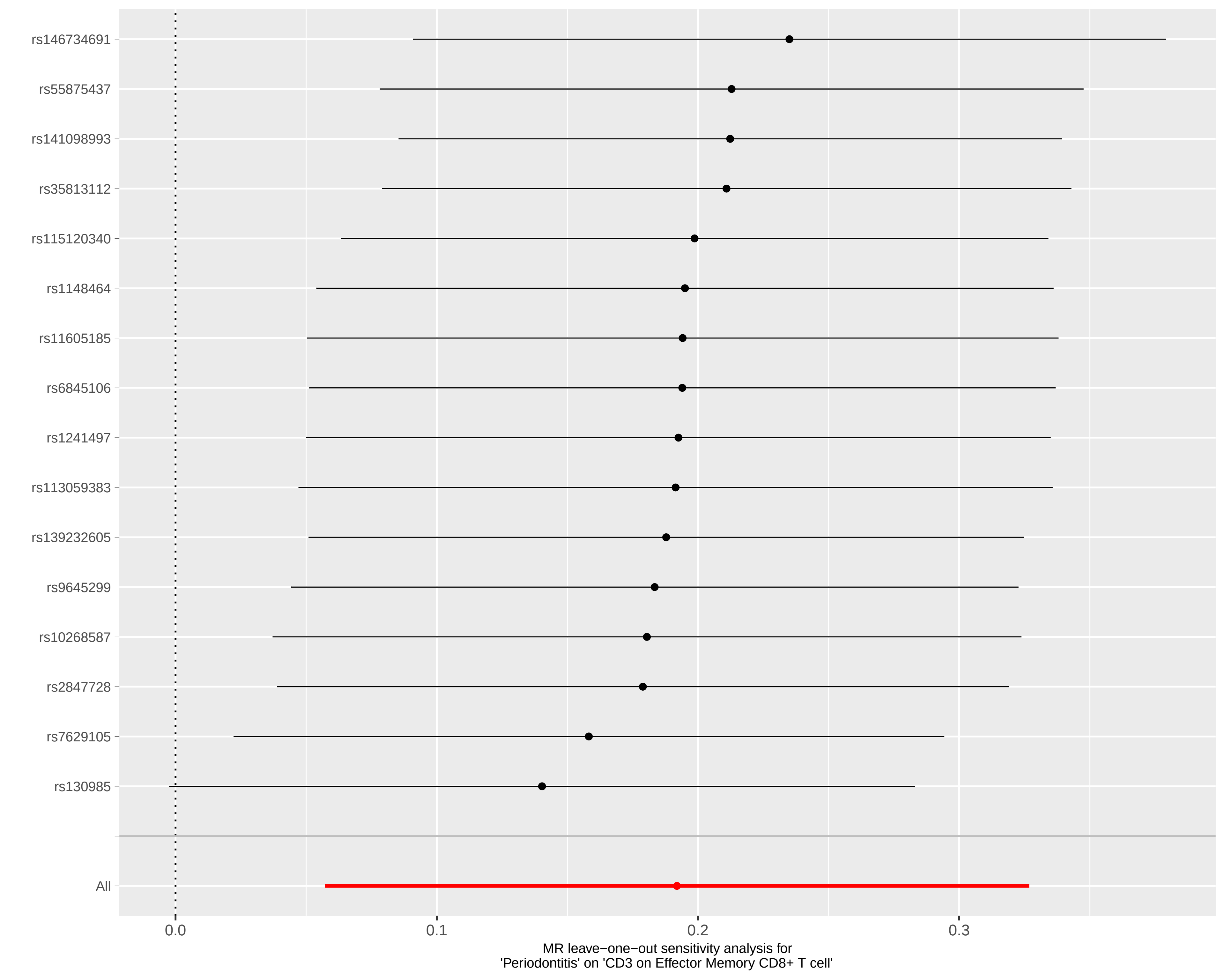
rs146734691
rs55875437
rs141098993
rs35813112
rs115120340
rs1148464
rs11605185
rs6845106
rs1241497
rs113059383
rs139232605
rs9645299
rs10268587
rs2847728
rs7629105
rs130985
All
0.0
0.1
0.2
0.3
MR leave−one−out sensitivity analysis for
'Periodontitis' on 'CD3 on Effector Memory CD8+ T cell'

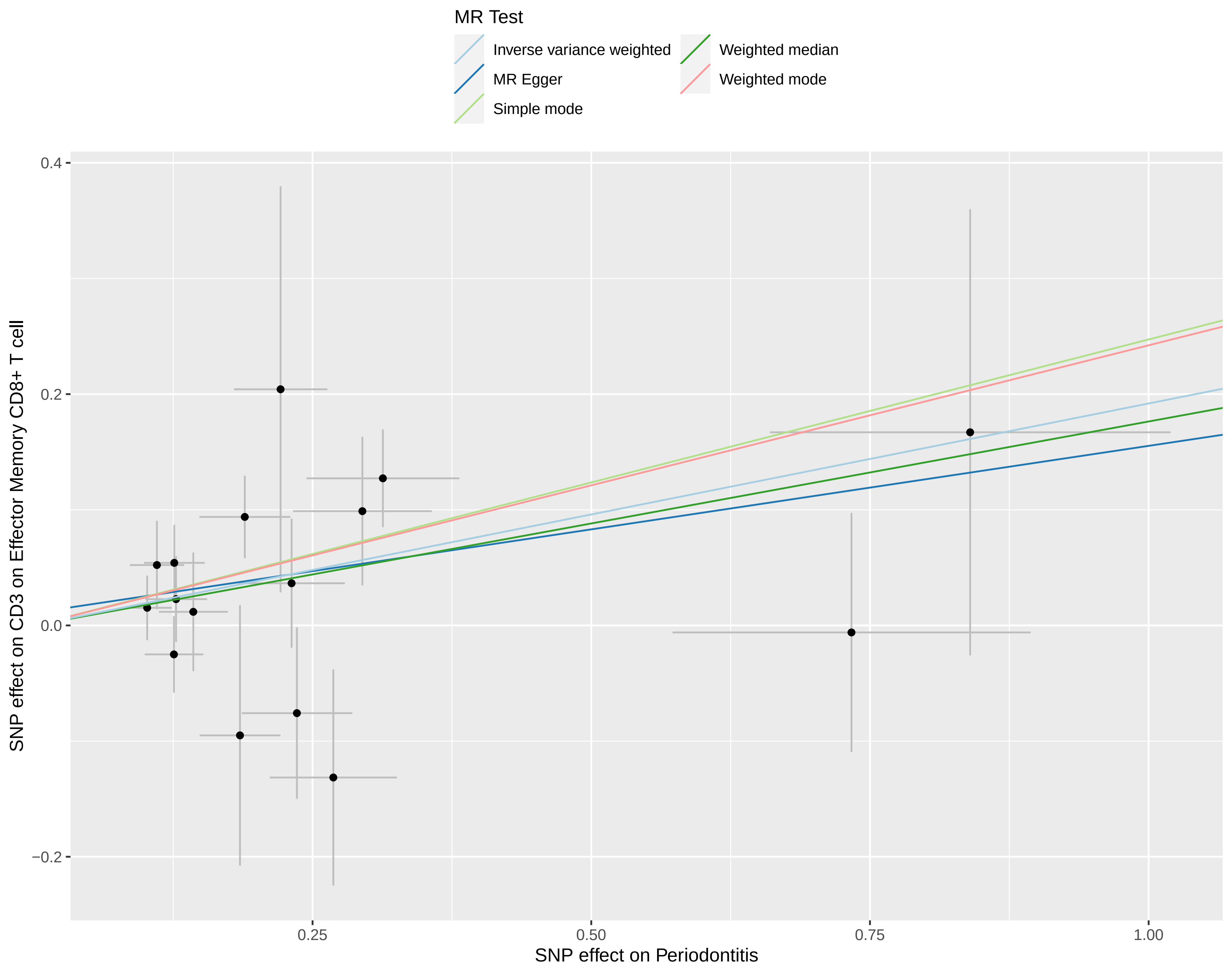

MR Test
Inverse variance weighted
MR Egger
Simple mode
Weighted median
Weighted mode
0.4
0.2
0.0
−0.2
SNP effect on CD3 on Effector Memory CD8+ T cell
0.25
0.50
0.75
1.00
SNP effect on Periodontitis

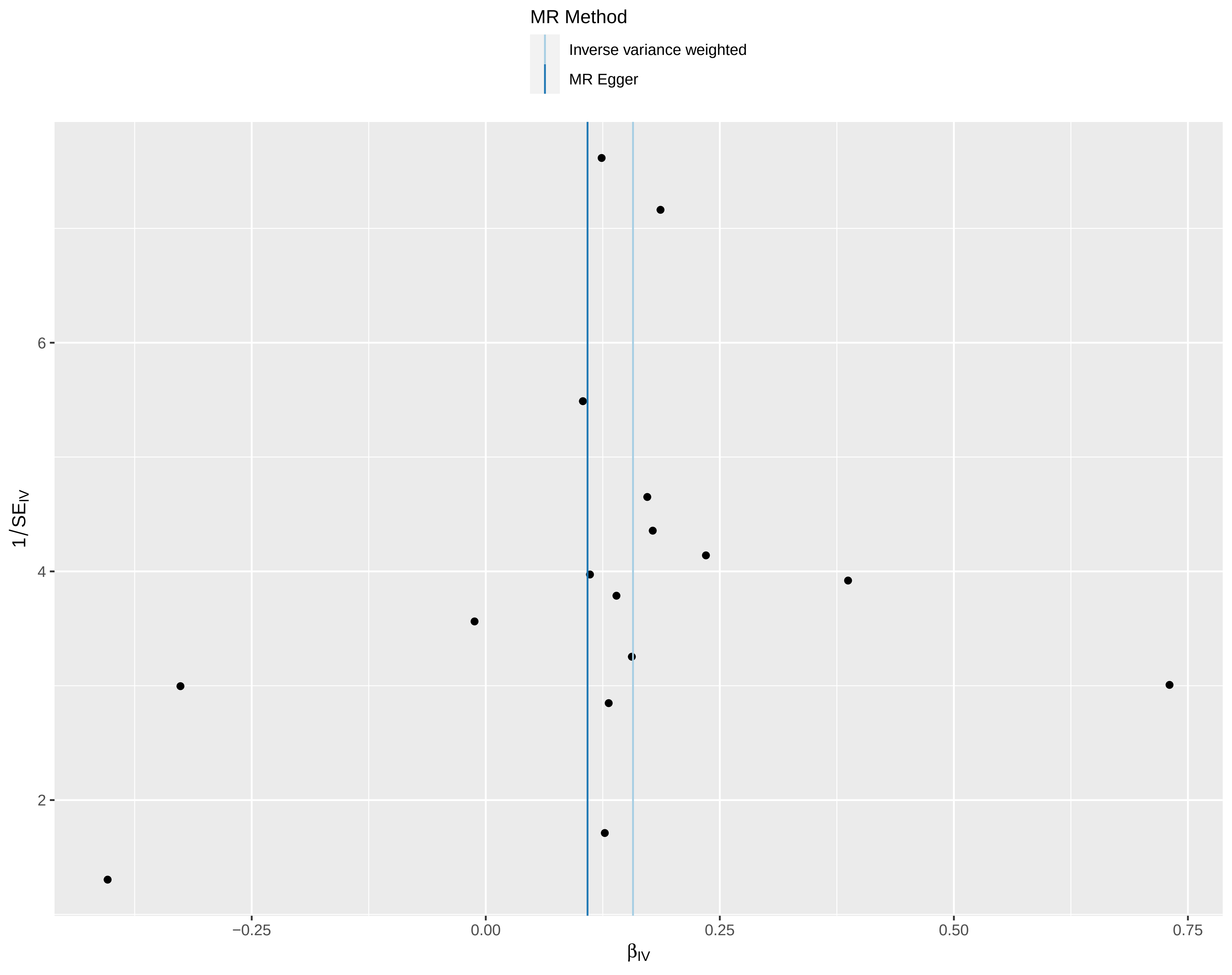
MR Method
Inverse variance weighted
MR Egger
6
4
2
1/SE_IV
−0.25
0.00
0.25
0.50
0.75
β_IV

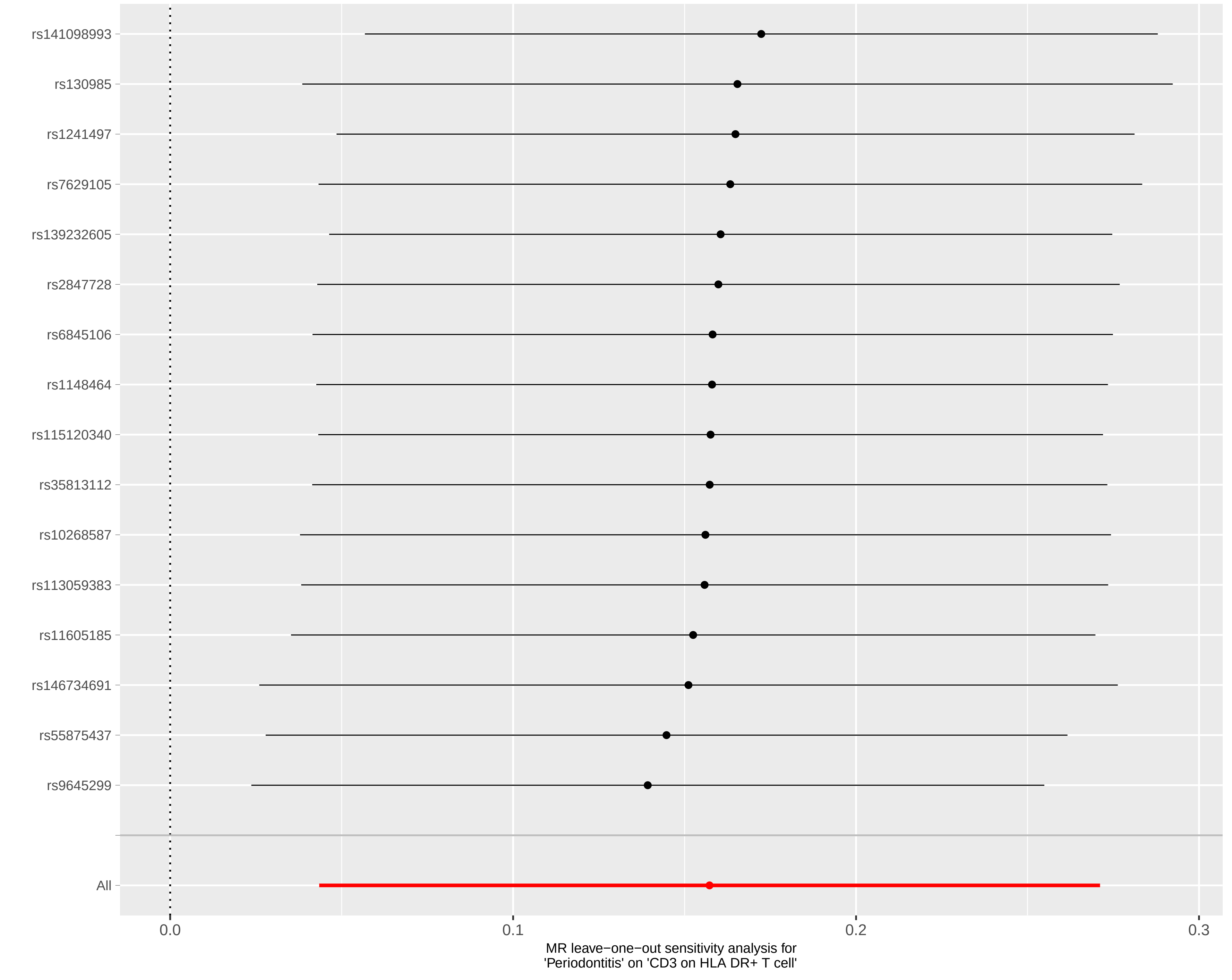
rs141098993
rs130985
rs1241497
rs7629105
rs139232605
rs2847728
rs6845106
rs1148464
rs115120340
rs35813112
rs10268587
rs113059383
rs11605185
rs146734691
rs55875437
rs9645299
All
0.0
0.1
0.2
0.3
MR leave−one−out sensitivity analysis for
'Periodontitis' on 'CD3 on HLA DR+ T cell'

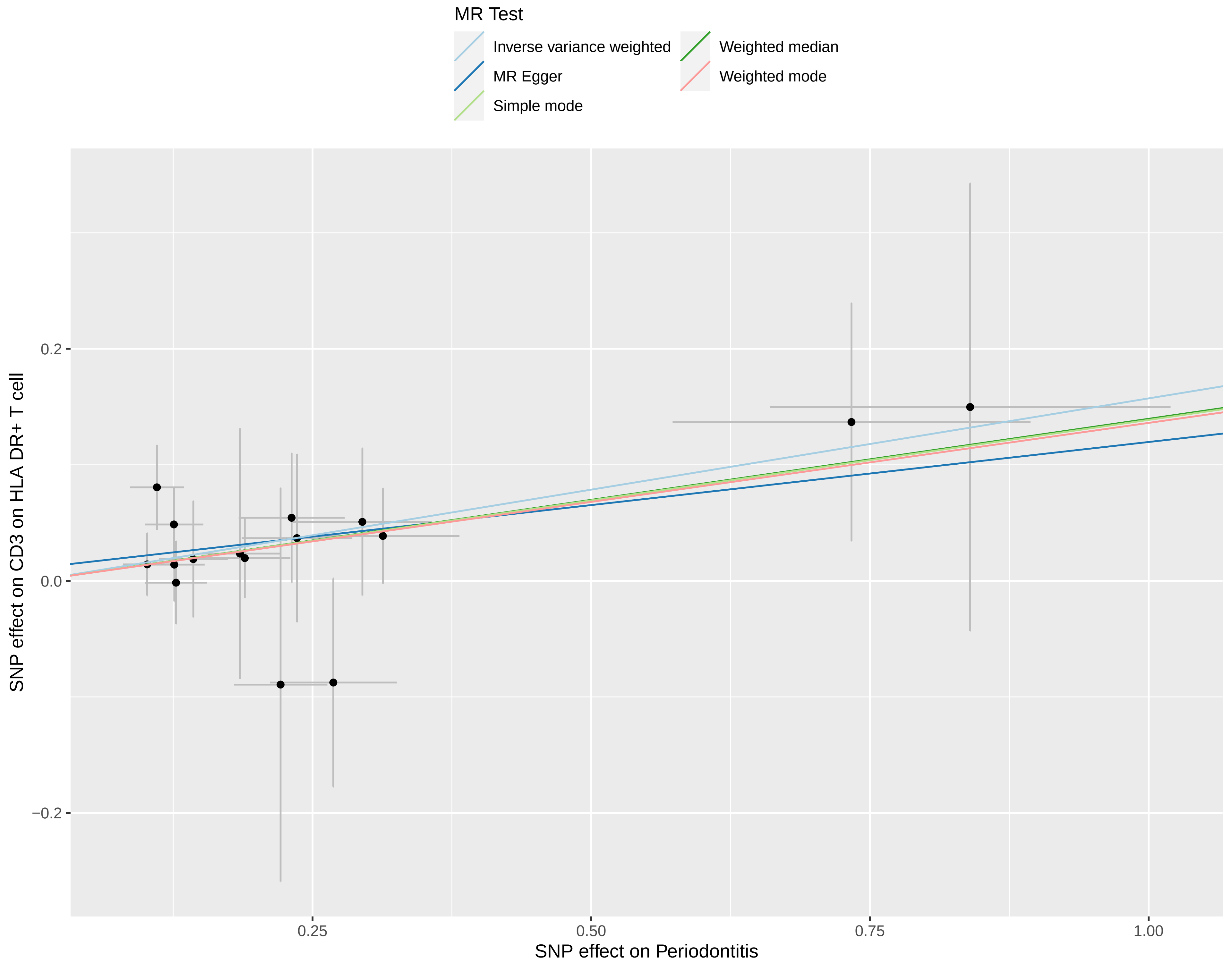

MR Test
Inverse variance weighted
MR Egger
Simple mode
Weighted median
Weighted mode
0.2
0.0
−0.2
0.25
0.50
0.75
1.00
SNP effect on CD3 on HLA DR+ T cell
SNP effect on Periodontitis

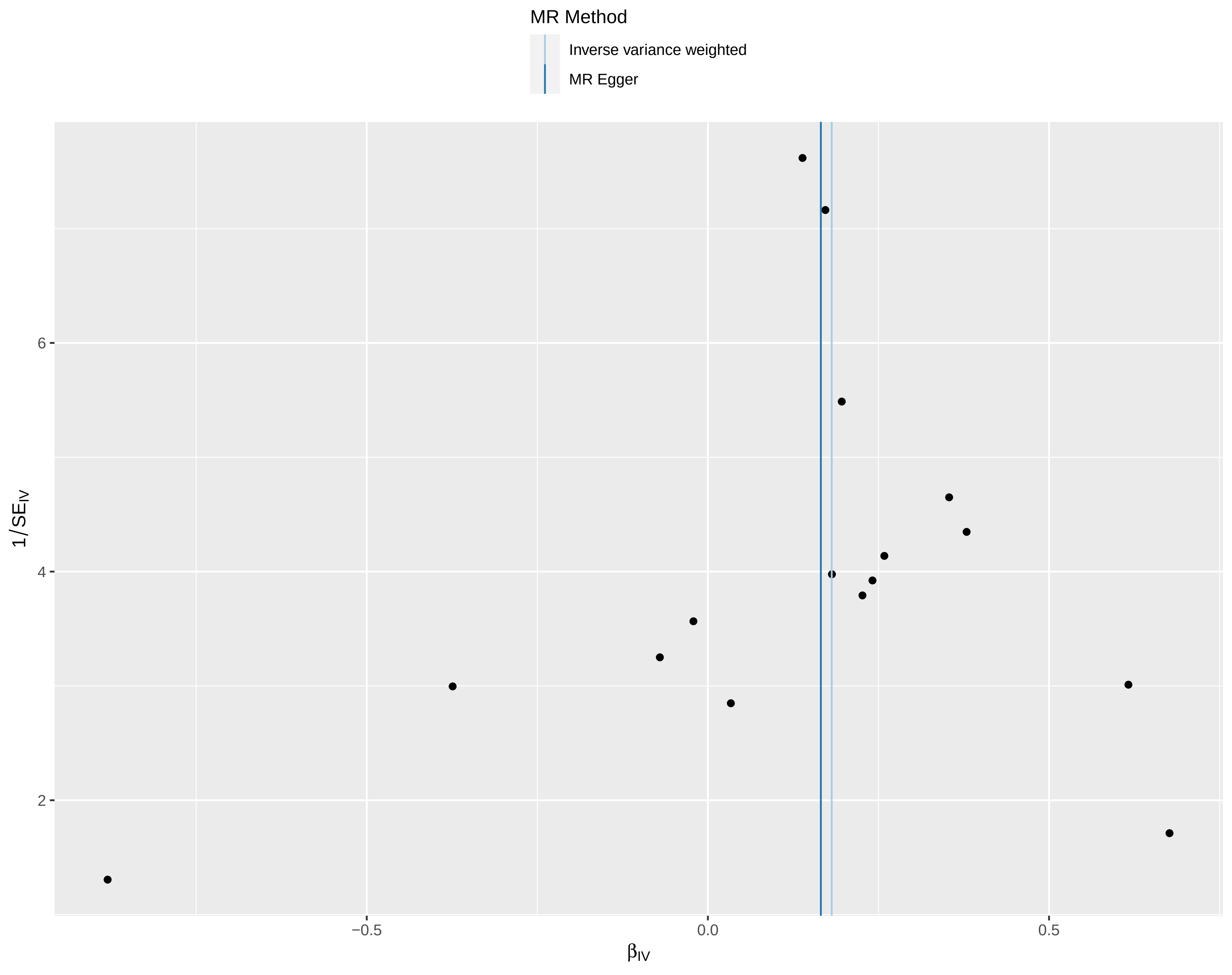
MR Method
Inverse variance weighted
MR Egger
6
4
2
1/SE_IV
−0.5
0.0
0.5
β_IV

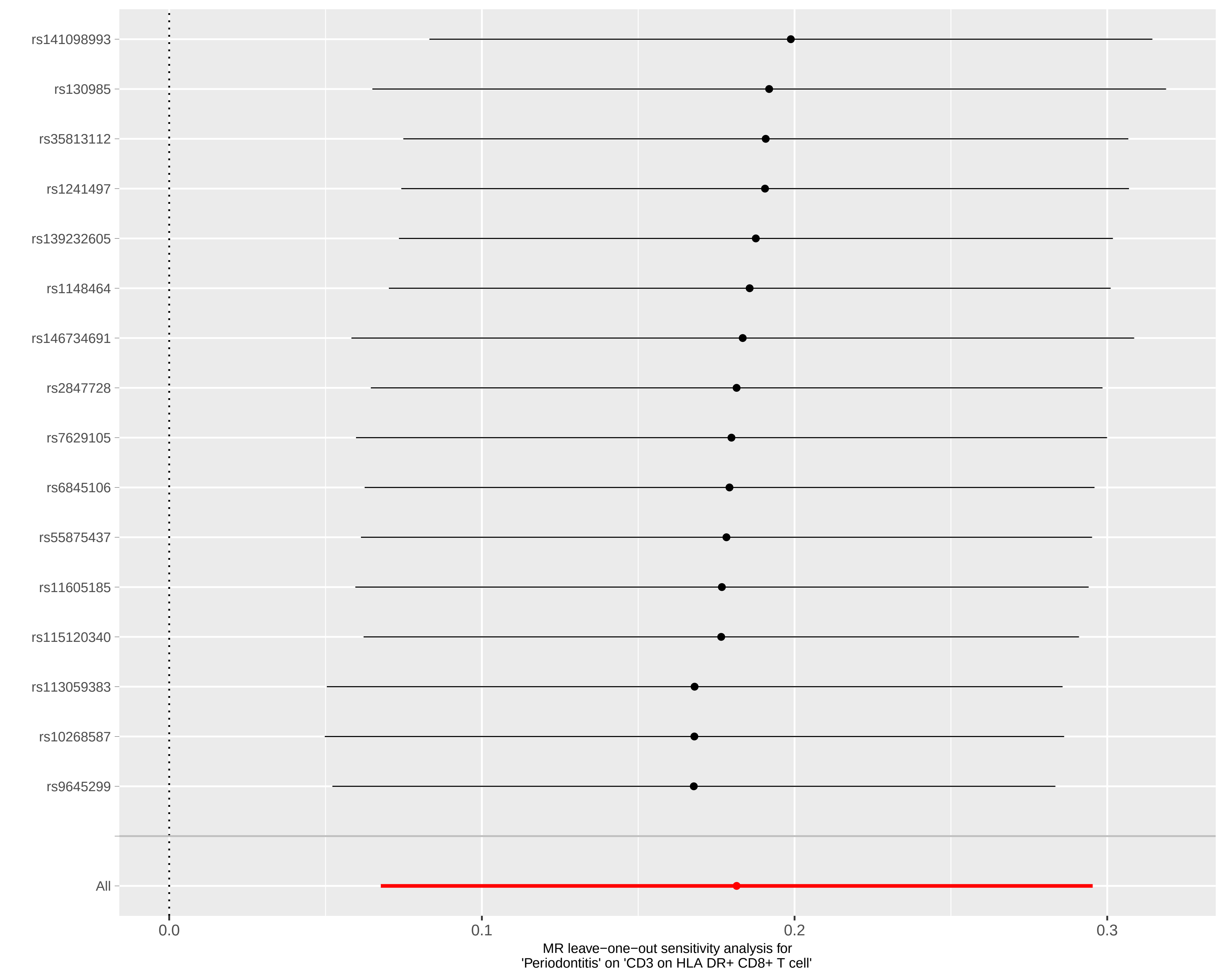
rs141098993
rs130985
rs35813112
rs1241497
rs139232605
rs1148464
rs146734691
rs2847728
rs7629105
rs6845106
rs55875437
rs11605185
rs115120340
rs113059383
rs10268587
rs9645299
All
0.0
0.1
0.2
0.3
MR leave−one−out sensitivity analysis for
'Periodontitis' on 'CD3 on HLA DR+ CD8+ T cell'

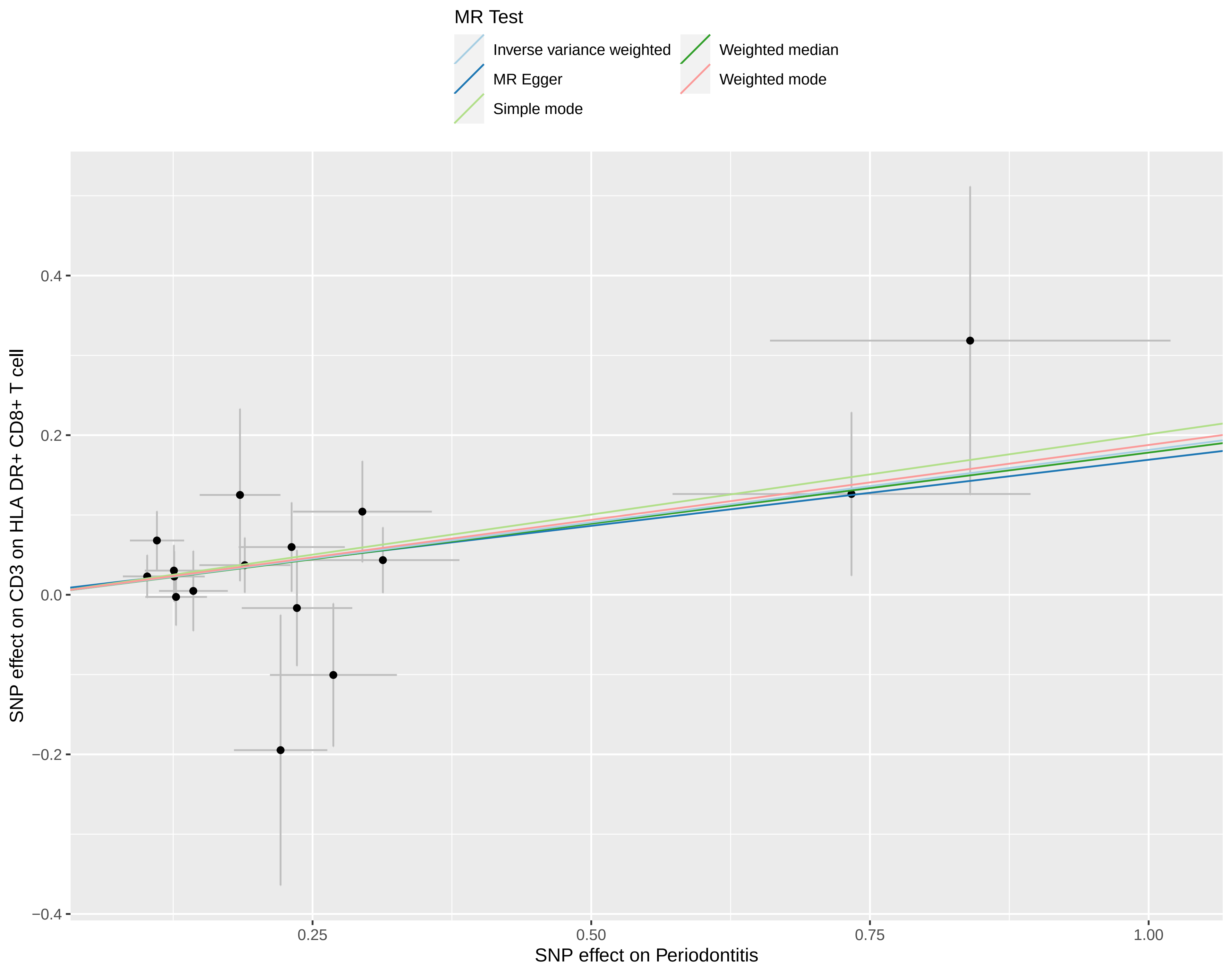

MR Test
Inverse variance weighted
MR Egger
Simple mode
Weighted median
Weighted mode
SNP effect on CD3 on HLA DR+ CD8+ T cell
0.4
0.2
0.0
−0.2
−0.4
0.25
0.50
0.75
1.00
SNP effect on Periodontitis

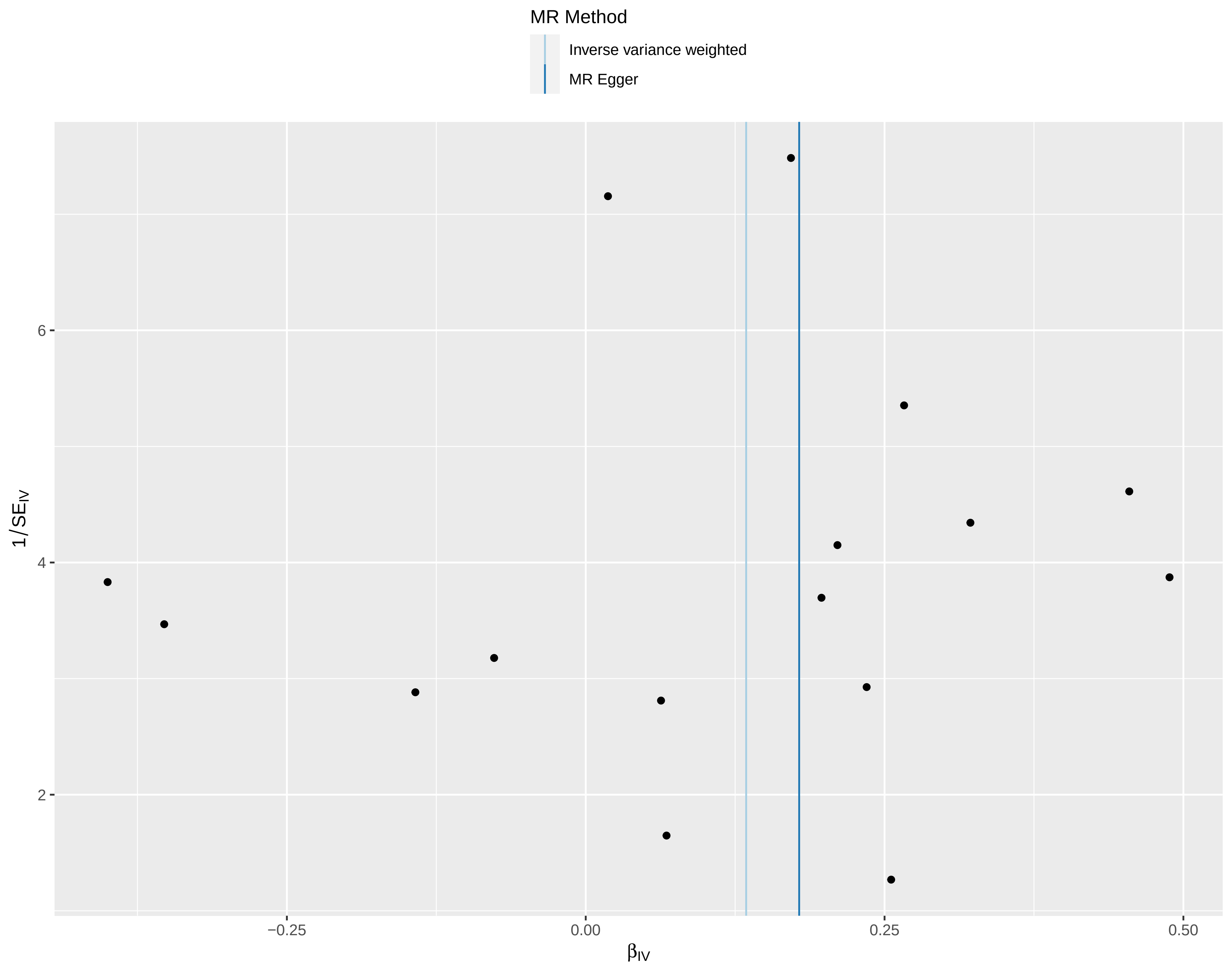
MR Method
Inverse variance weighted
MR Egger
6
4
2
$1/SE_{IV}$
−0.25
0.00
0.25
0.50
β_{IV}

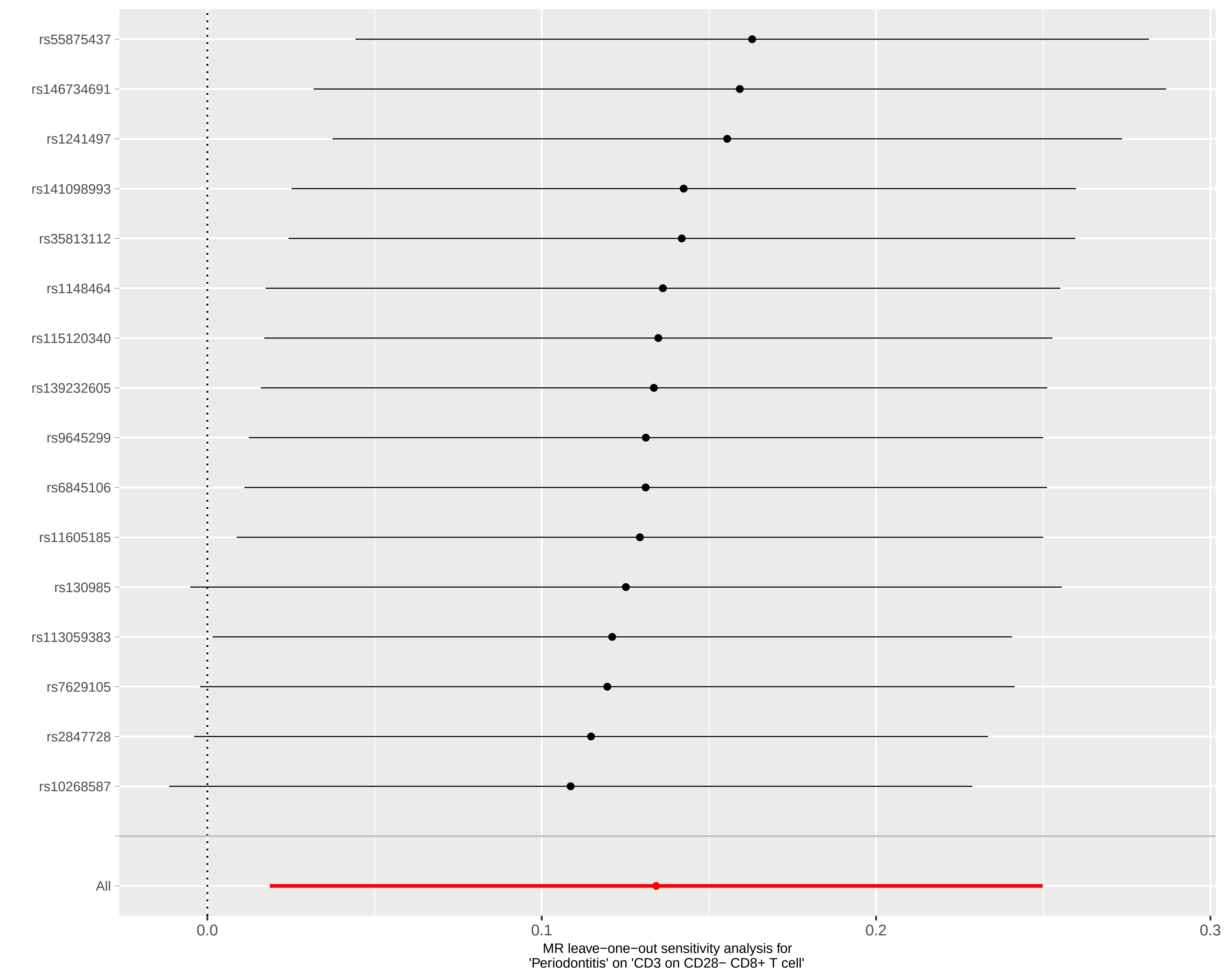

rs55875437
rs146734691
rs1241497
rs141098993
rs35813112
rs1148464
rs115120340
rs139232605
rs9645299
rs6845106
rs11605185
rs130985
rs113059383
rs7629105
rs2847728
rs10268587
All
0.0
0.1
0.2
0.3
MR leave−one−out sensitivity analysis for
'Periodontitis' on 'CD3 on CD28− CD8+ T cell'

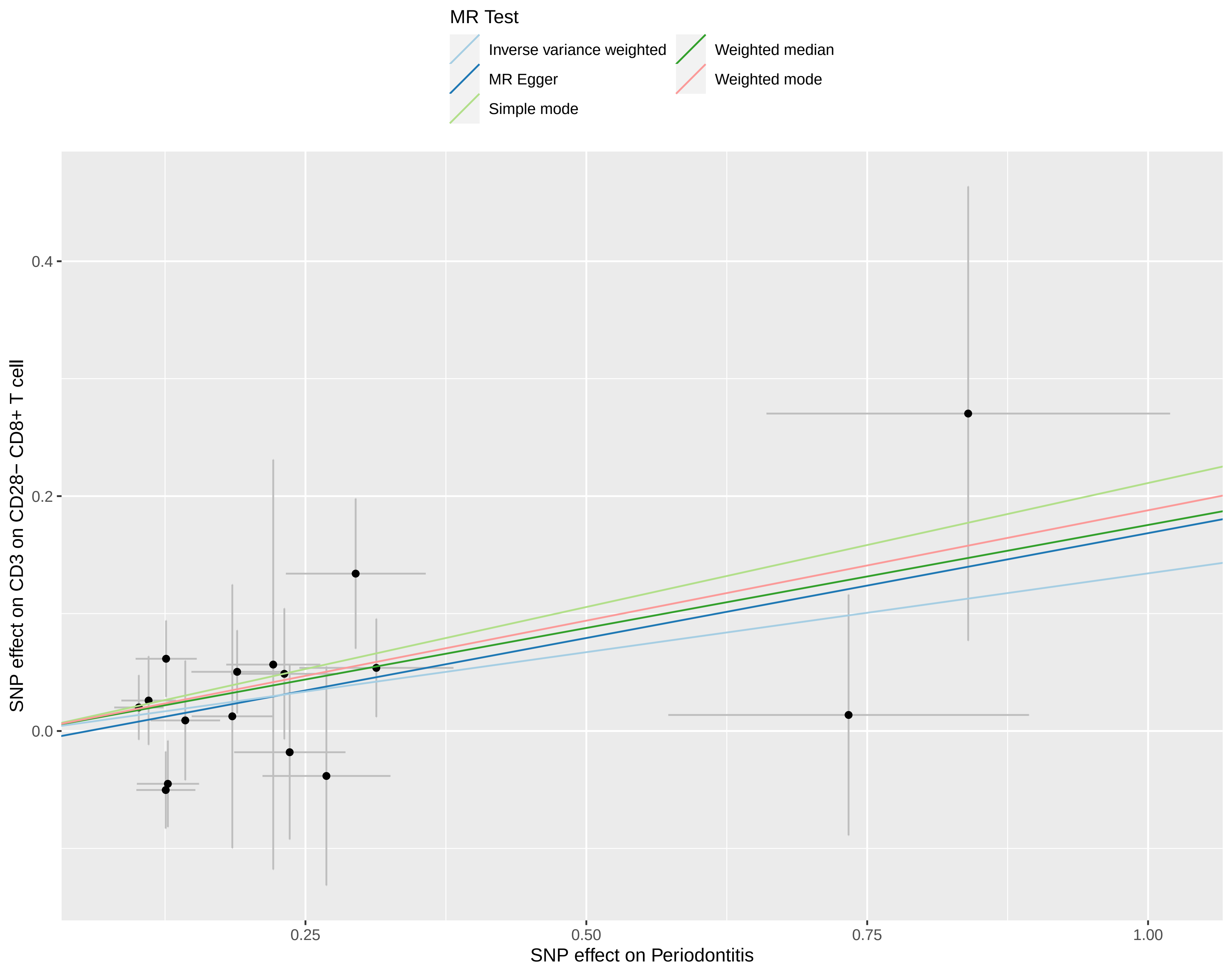
MR Test
Inverse variance weighted
MR Egger
Simple mode
Weighted median
Weighted mode
0.4
0.2
0.0
SNP effect on CD3 on CD28− CD8+ T cell
0.25
0.50
0.75
1.00
SNP effect on Periodontitis

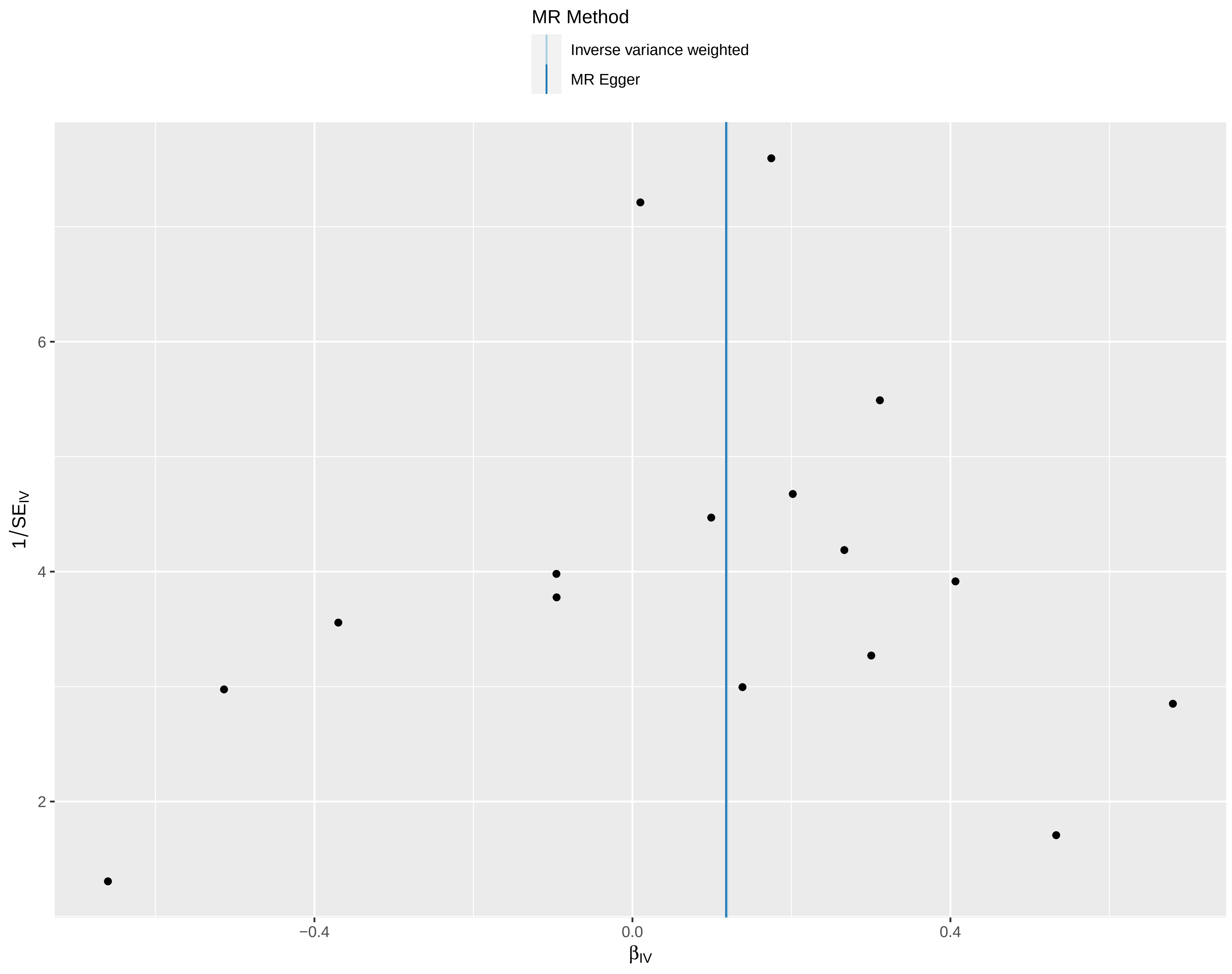

MR Method
Inverse variance weighted
MR Egger
1/SE_IV
6
4
2
−0.4
0.0
0.4
β_IV

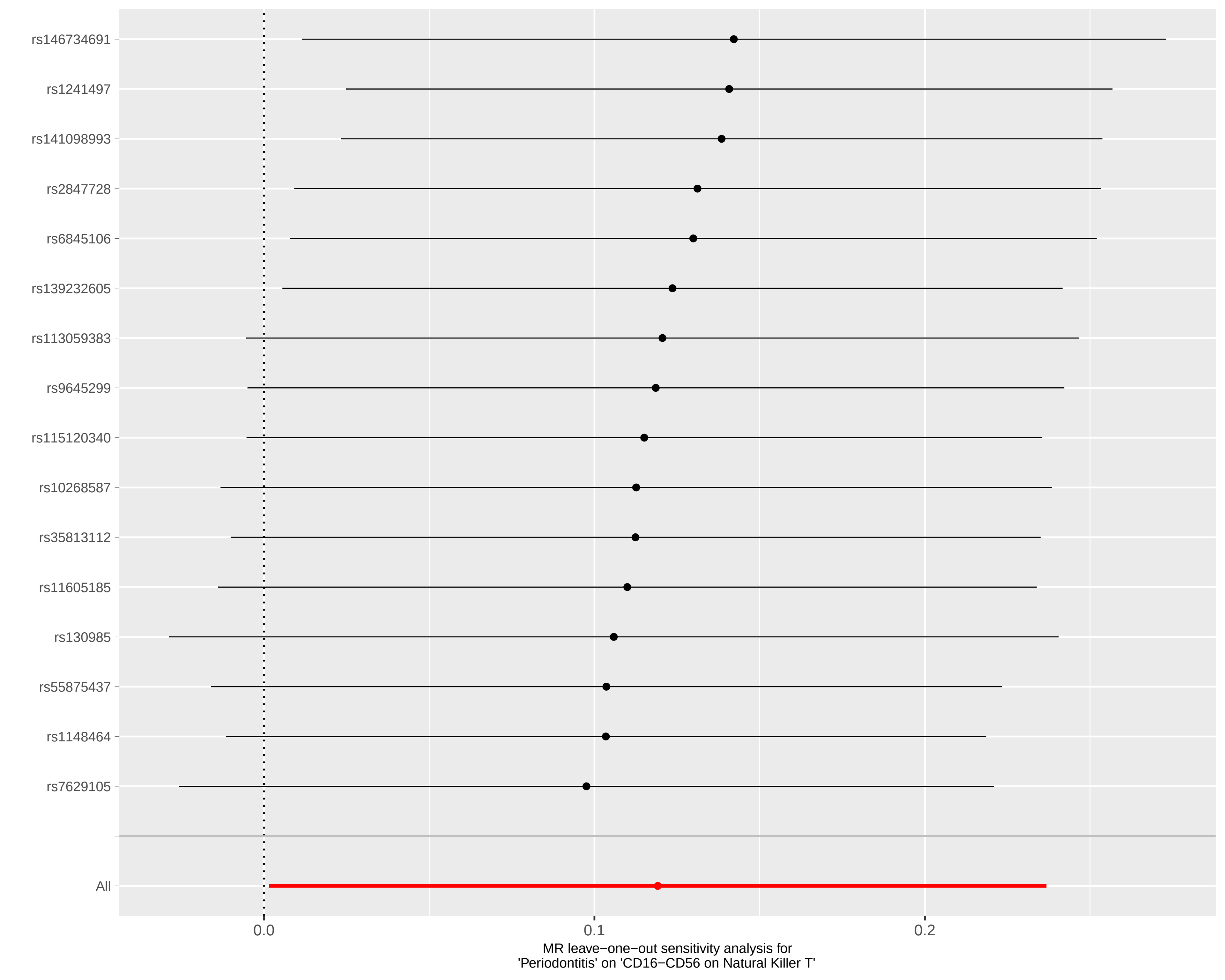

rs146734691
rs1241497
rs141098993
rs2847728
rs6845106
rs139232605
rs113059383
rs9645299
rs115120340
rs10268587
rs35813112
rs11605185
rs130985
rs55875437
rs1148464
rs7629105
All
0.0
0.1
0.2
MR leave−one−out sensitivity analysis for
'Periodontitis' on 'CD16−CD56 on Natural Killer T'

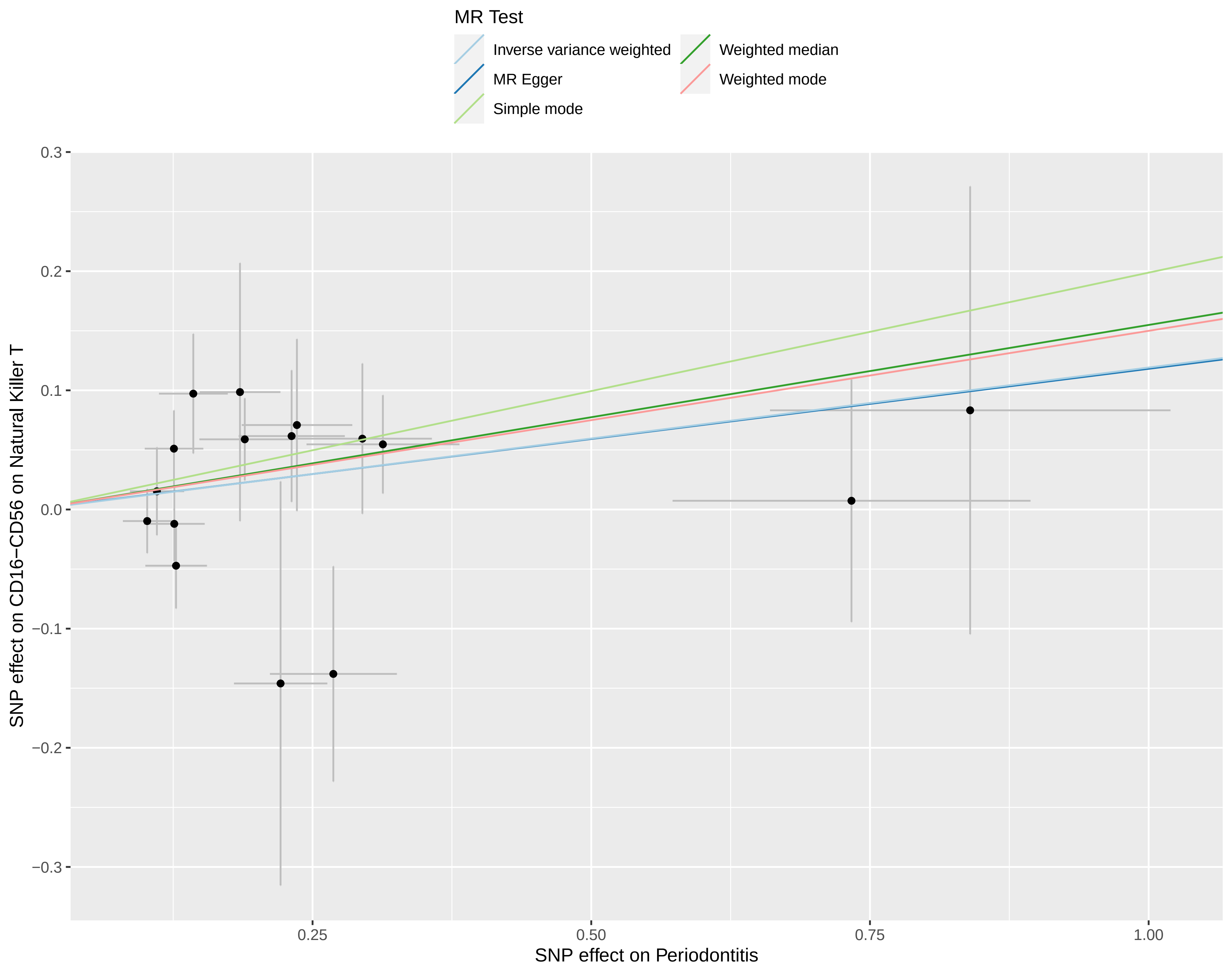

MR Test
Inverse variance weighted
MR Egger
Simple mode
Weighted median
Weighted mode
0.3
0.2
0.1
0.0
−0.1
−0.2
−0.3
0.25
0.50
0.75
1.00
SNP effect on CD16−CD56 on Natural Killer T
SNP effect on Periodontitis

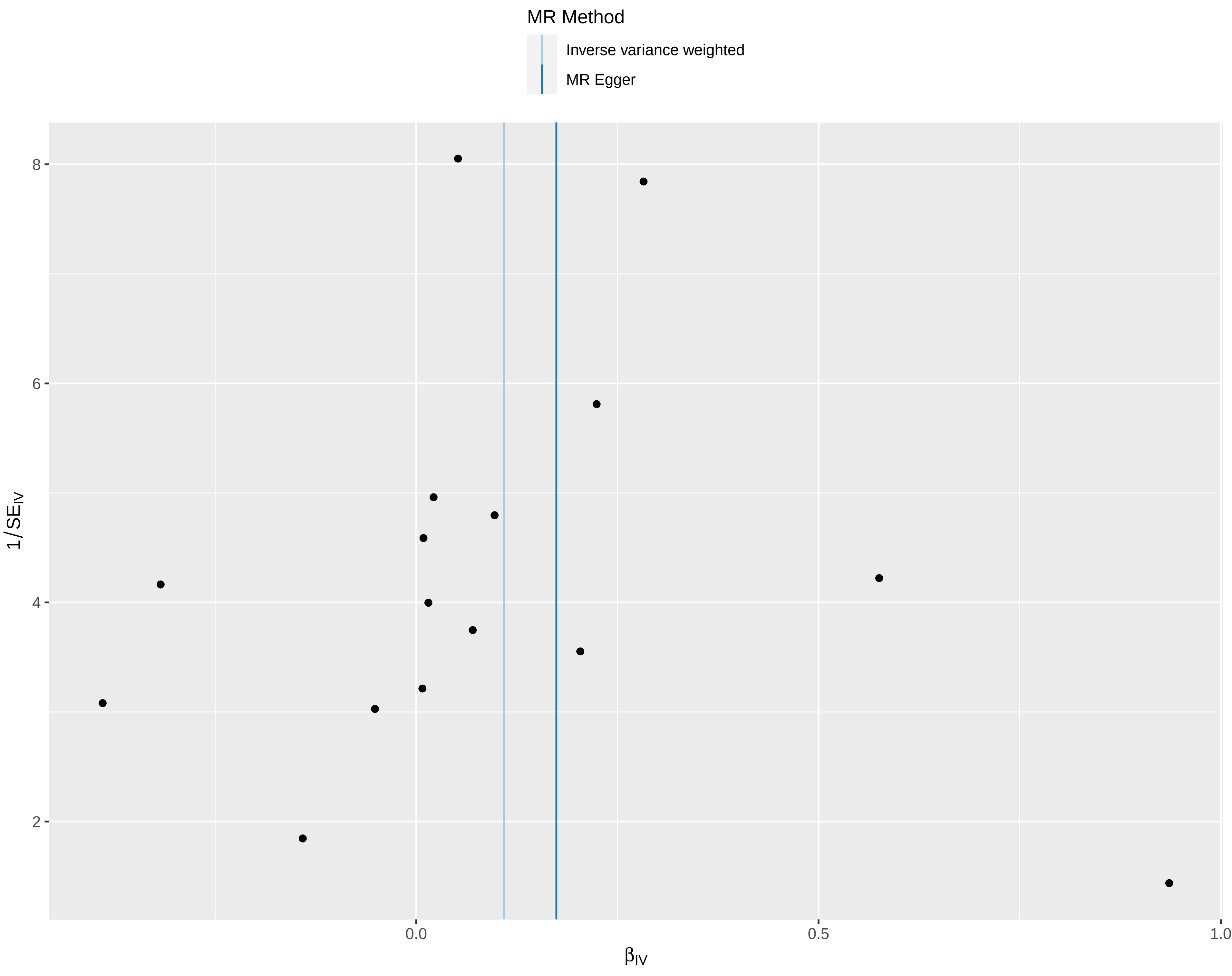

MR Method
Inverse variance weighted
MR Egger
8
6
4
2
1/SE_IV
0.0
0.5
1.0
β_IV

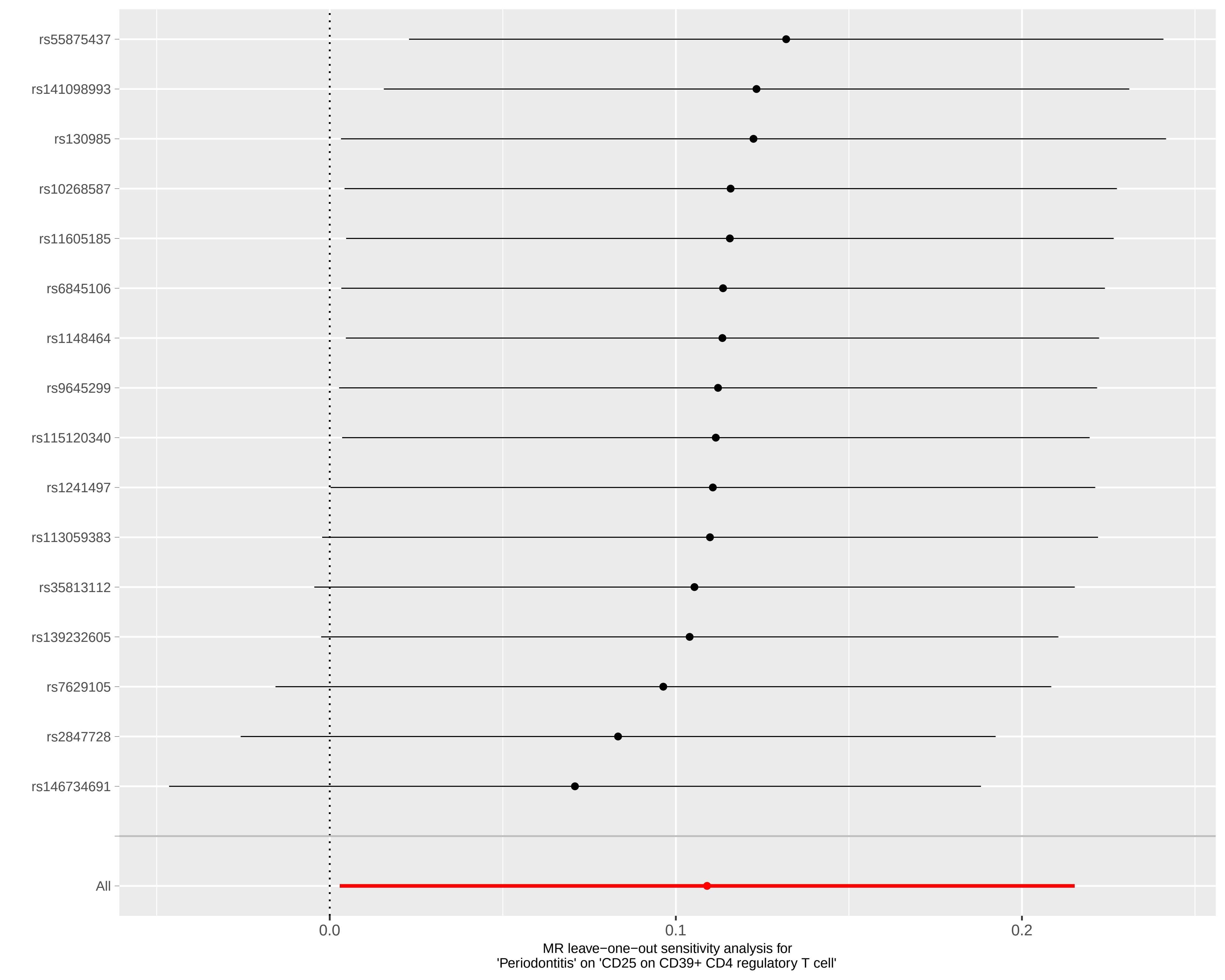
rs55875437
rs141098993
rs130985
rs10268587
rs11605185
rs6845106
rs1148464
rs9645299
rs115120340
rs1241497
rs113059383
rs35813112
rs139232605
rs7629105
rs2847728
rs146734691
All
0.0
0.1
0.2
MR leave−one−out sensitivity analysis for
'Periodontitis' on 'CD25 on CD39+ CD4 regulatory T cell'

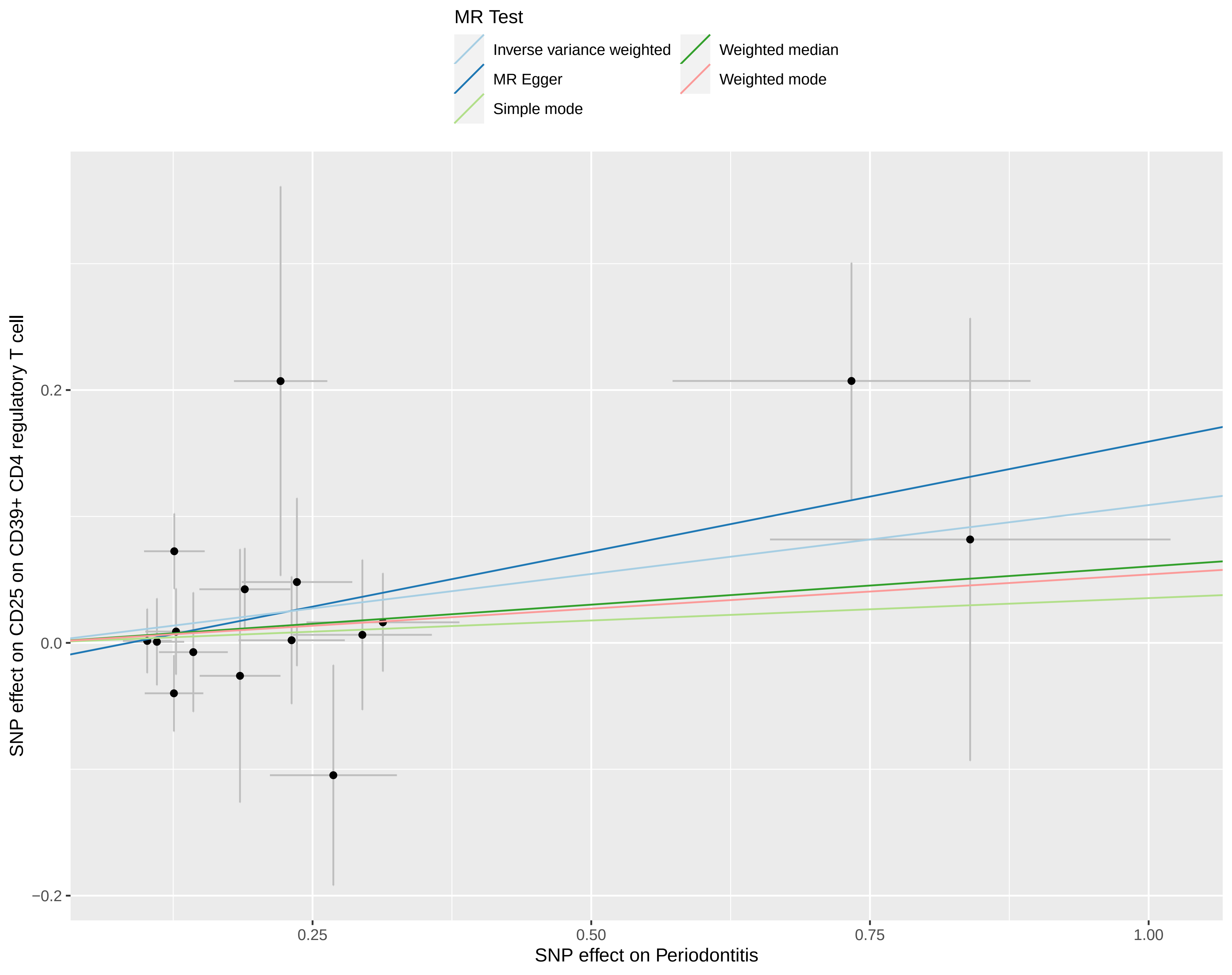
MR Test
Inverse variance weighted
MR Egger
Simple mode
Weighted median
Weighted mode
SNP effect on CD25 on CD39+ CD4 regulatory T cell
0.2
0.0
−0.2
0.25
0.50
0.75
1.00
SNP effect on Periodontitis

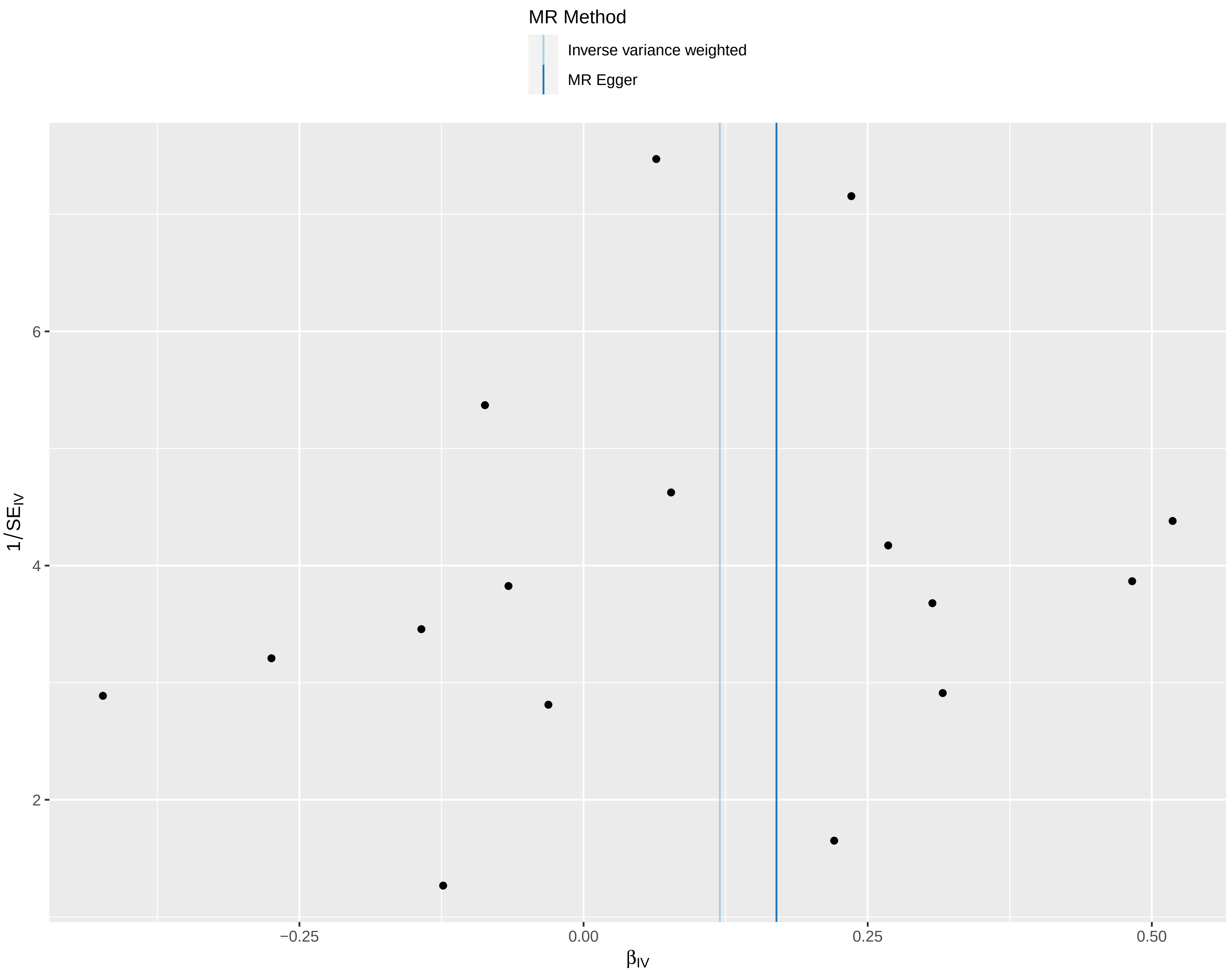

MR Method
Inverse variance weighted
MR Egger
1/SE_IV
β_IV
6
4
2
−0.25
0.00
0.25
0.50

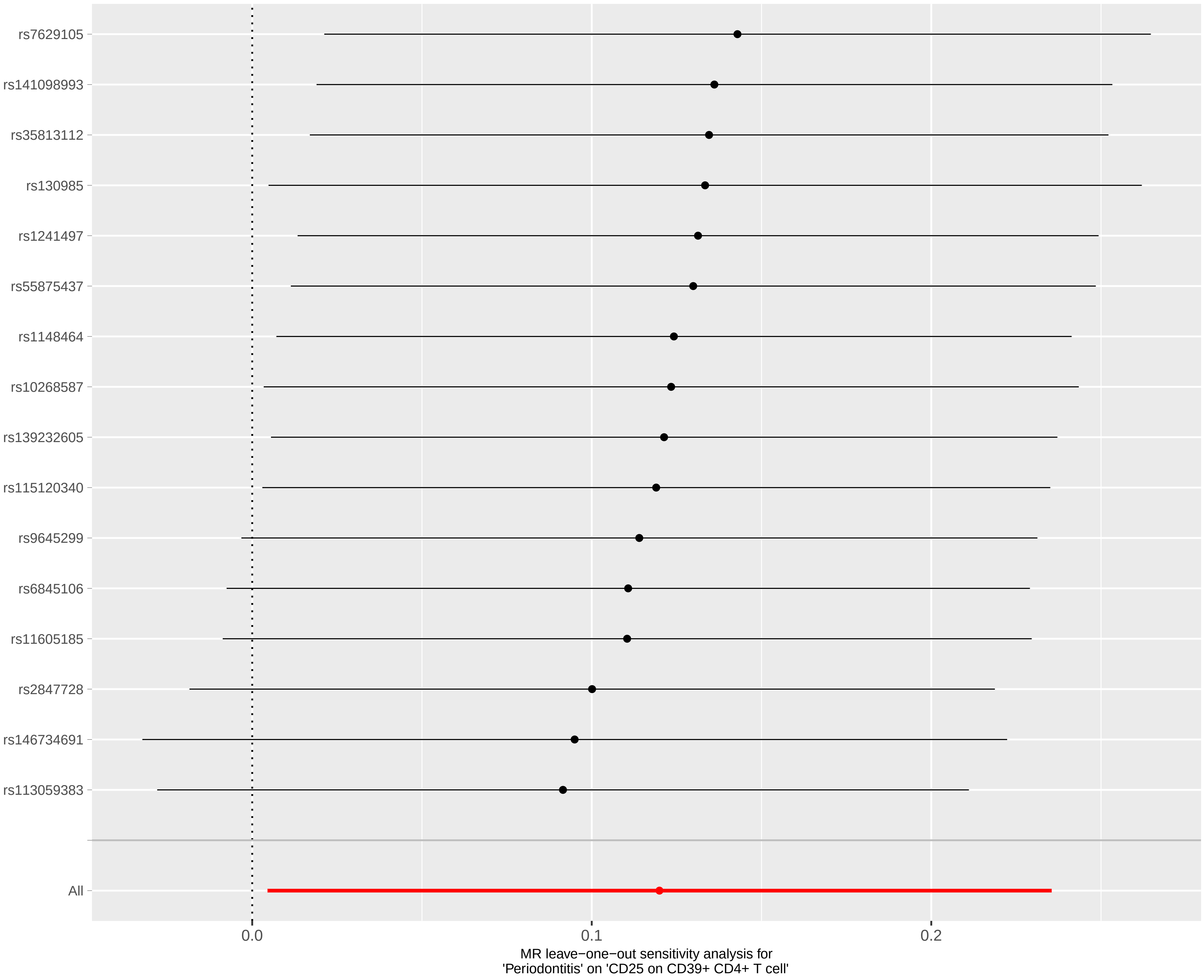
rs7629105
rs141098993
rs35813112
rs130985
rs1241497
rs55875437
rs1148464
rs10268587
rs139232605
rs115120340
rs9645299
rs6845106
rs11605185
rs2847728
rs146734691
rs113059383
All
0.0
0.1
0.2
MR leave−one−out sensitivity analysis for
'Periodontitis' on 'CD25 on CD39+ CD4+ T cell'

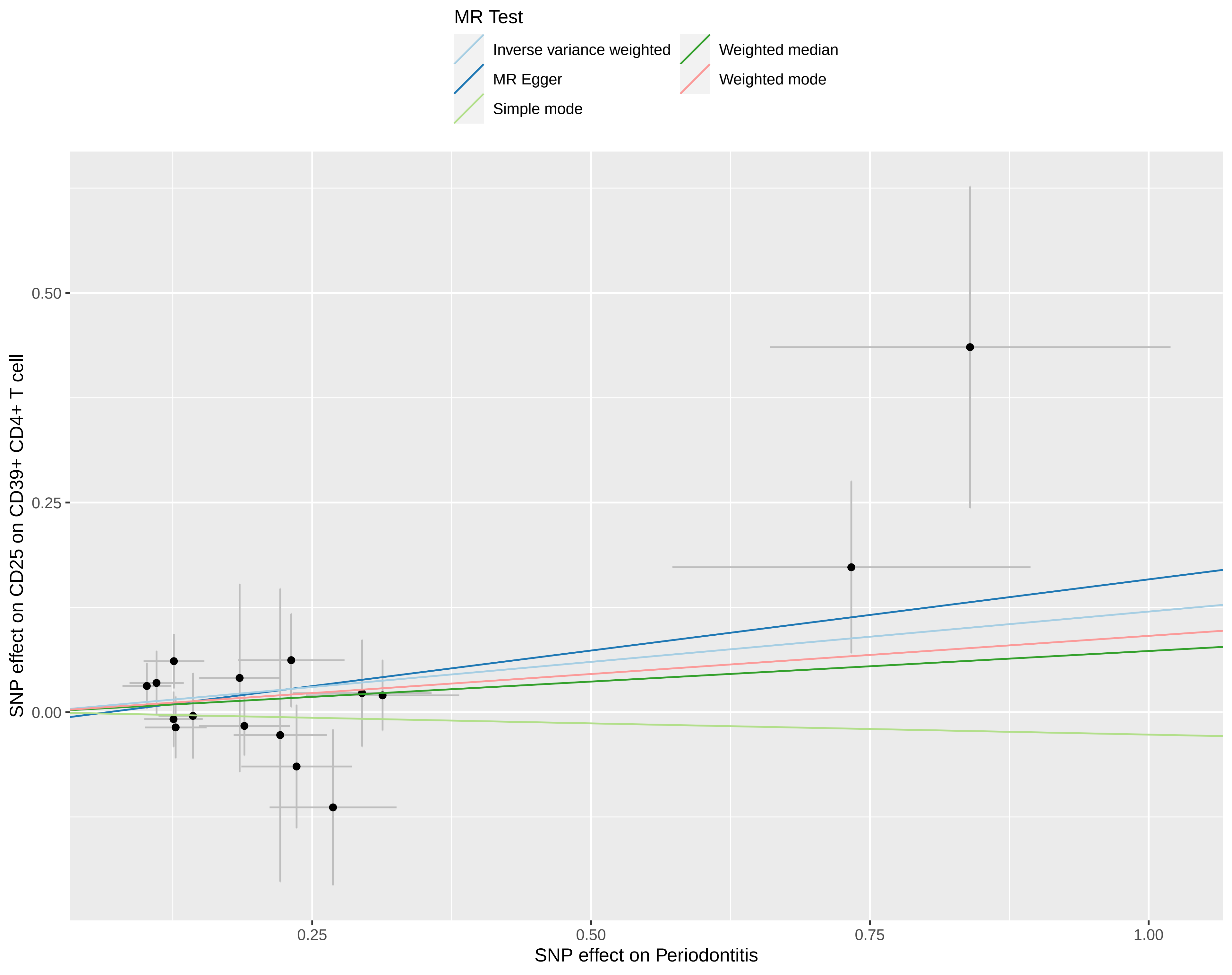
MR Test
Inverse variance weighted
MR Egger
Simple mode
Weighted median
Weighted mode
SNP effect on CD25 on CD39+ CD4+ T cell
0.50
0.25
0.00
SNP effect on Periodontitis
0.25
0.50
0.75
1.00

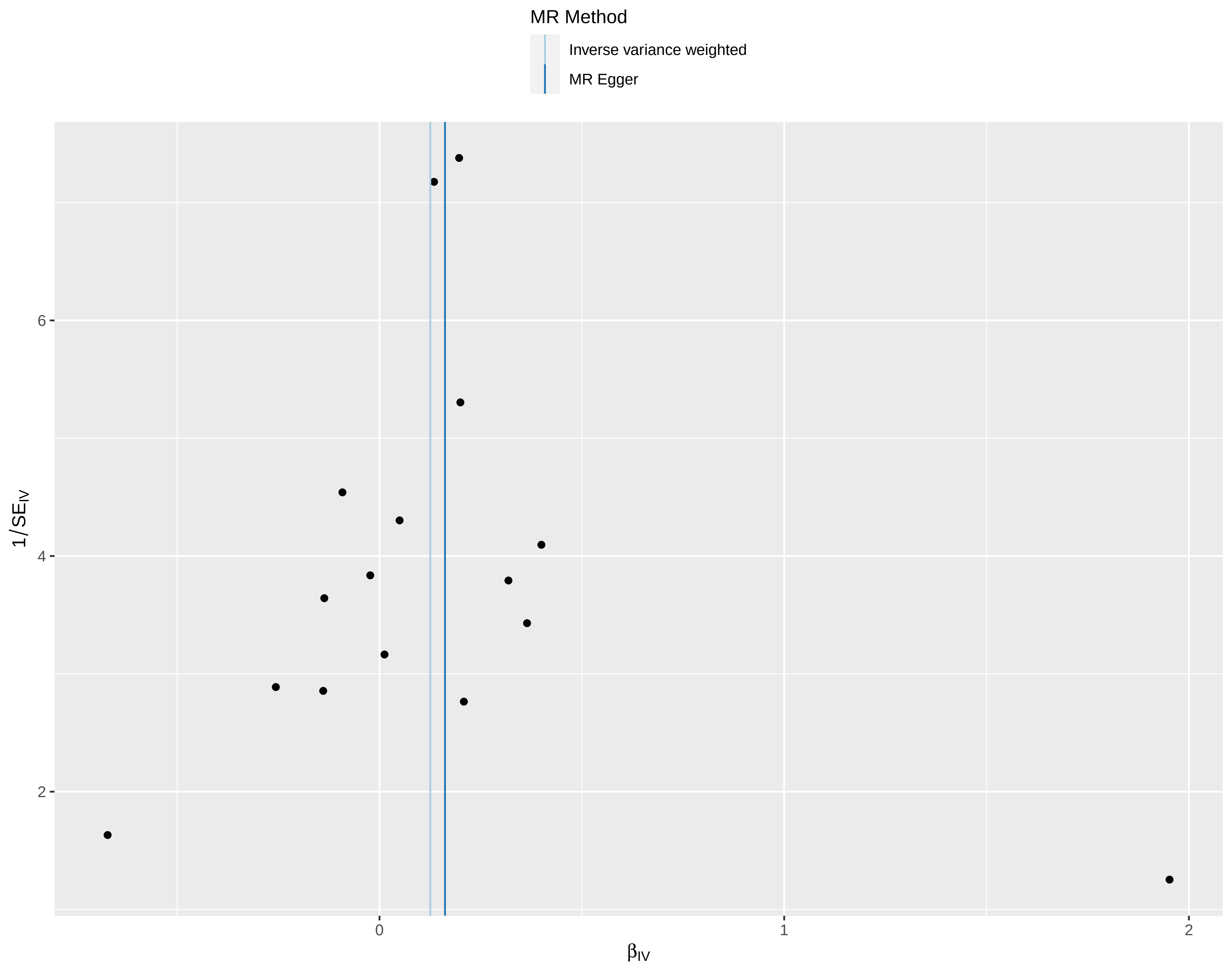

MR Method
Inverse variance weighted
MR Egger
1/SE_IV
6
4
2
0
1
2
β_IV

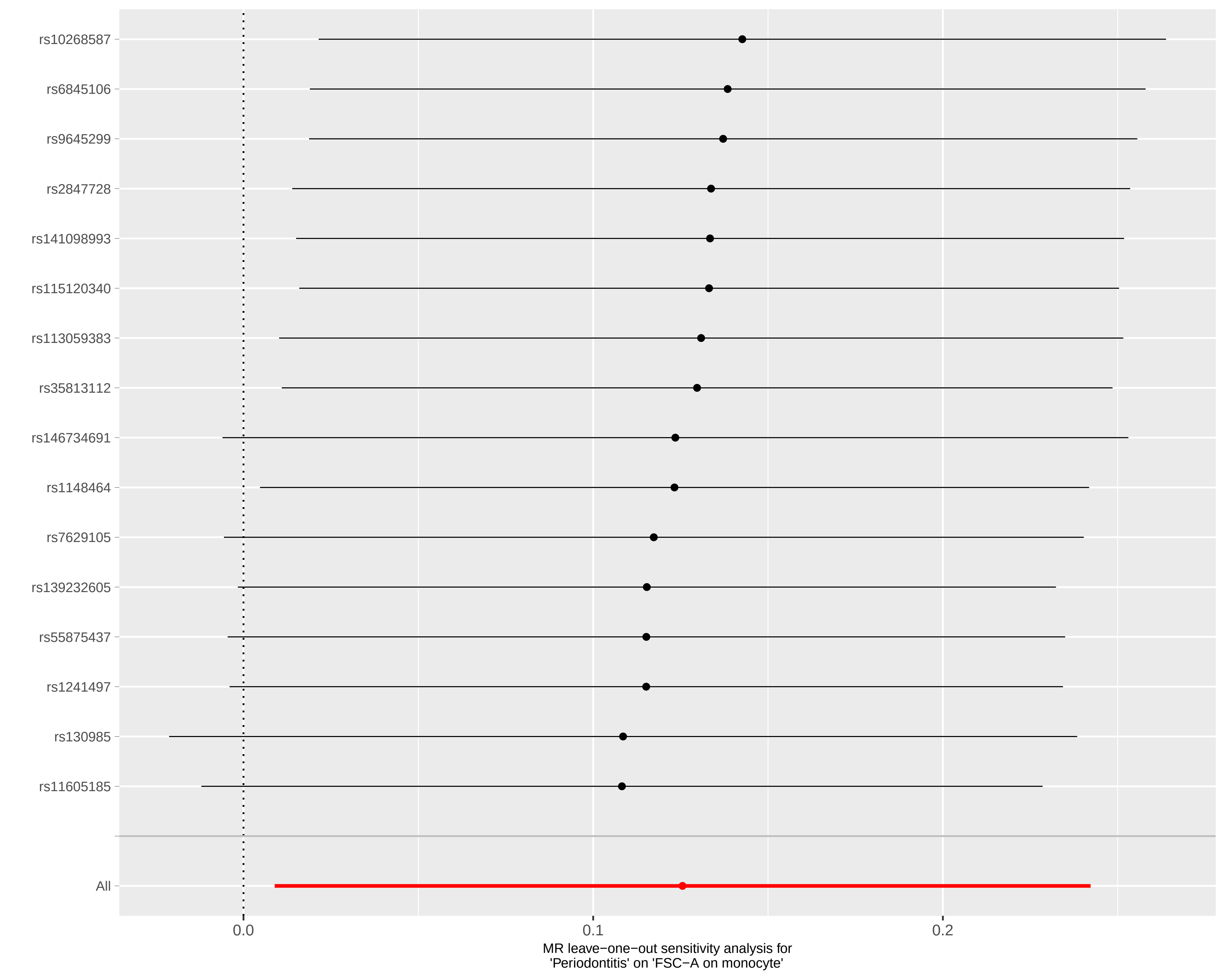
rs10268587
rs6845106
rs9645299
rs2847728
rs141098993
rs115120340
rs113059383
rs35813112
rs146734691
rs1148464
rs7629105
rs139232605
rs55875437
rs1241497
rs130985
rs11605185
All
0.0
0.1
0.2
MR leave−one−out sensitivity analysis for
'Periodontitis' on 'FSC−A on monocyte'

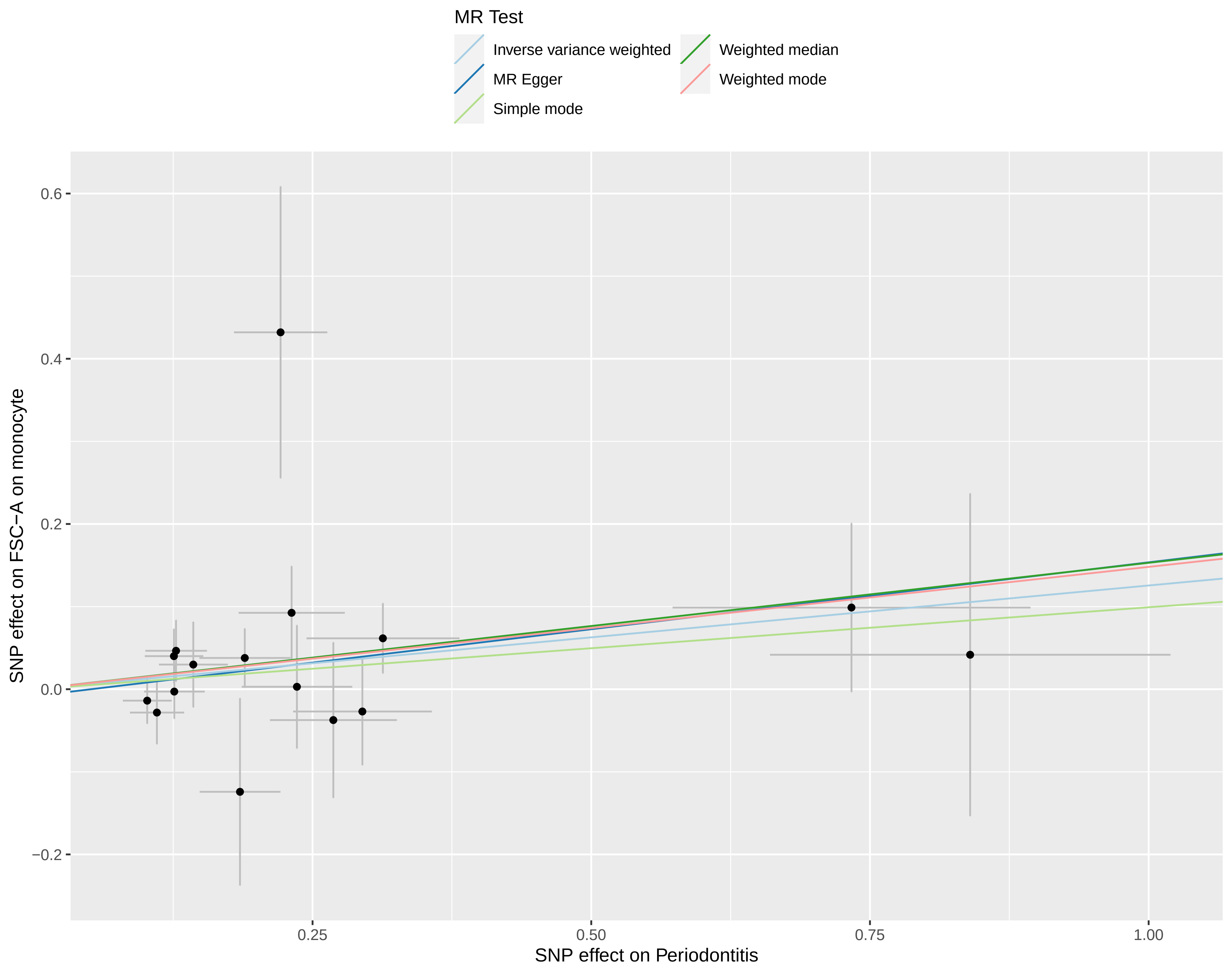
MR Test
Inverse variance weighted
MR Egger
Simple mode
Weighted median
Weighted mode
0.6
0.4
0.2
0.0
−0.2
0.25
0.50
0.75
1.00
SNP effect on FSC−A on monocyte
SNP effect on Periodontitis

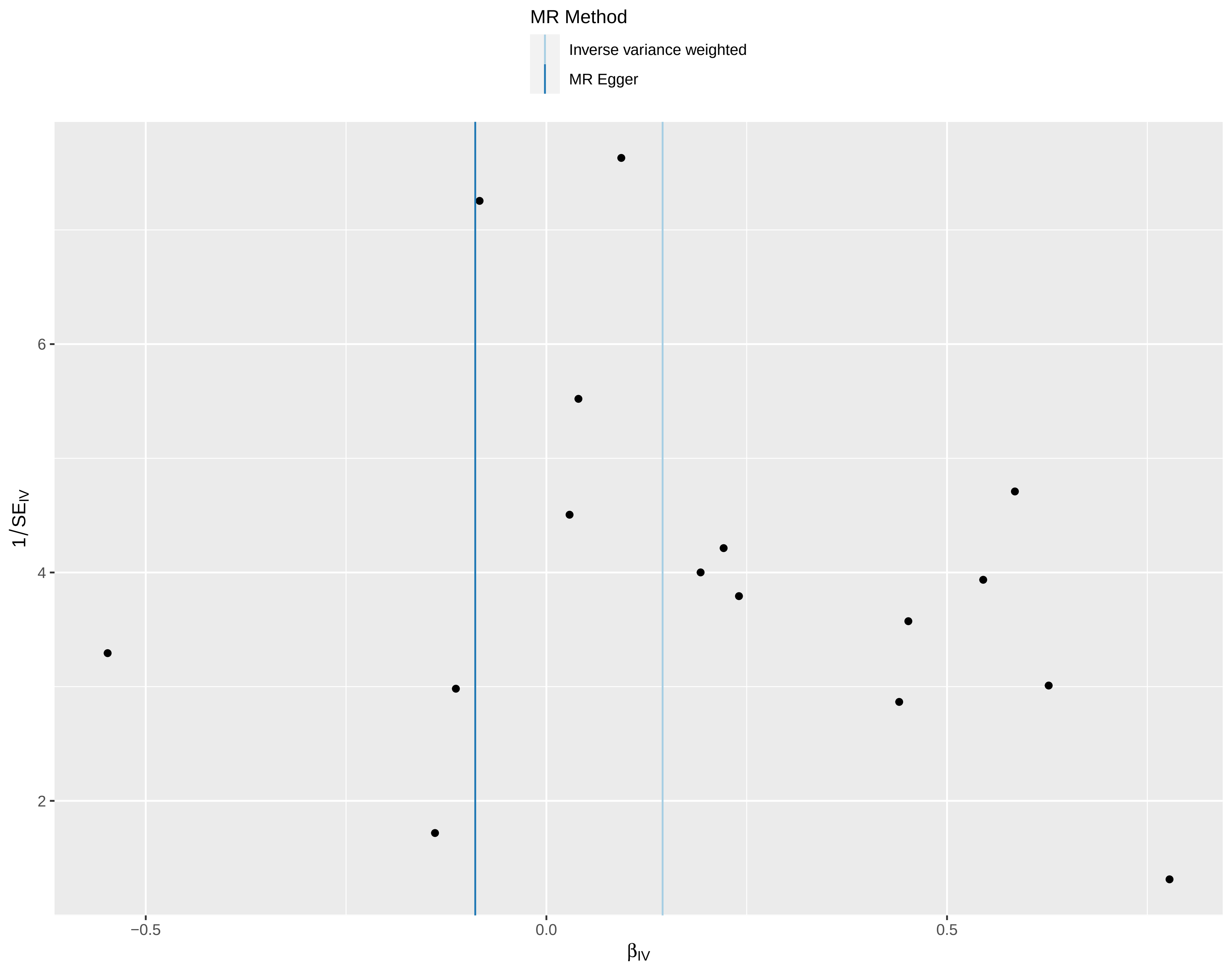
MR Method
Inverse variance weighted
MR Egger
6
4
2
1/SE$_{IV}$
−0.5
0.0
0.5
β$_{IV}$

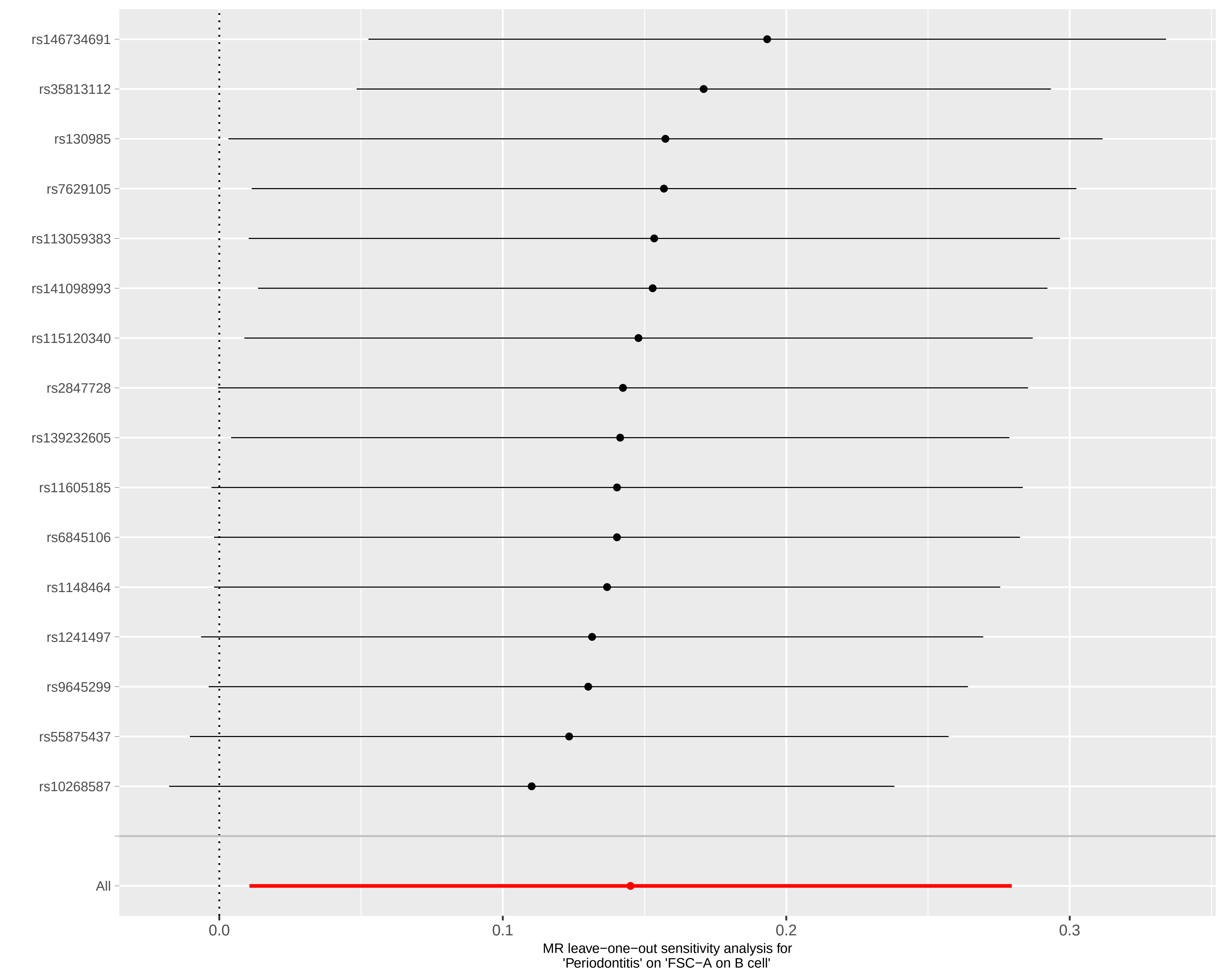

rs146734691
rs35813112
rs130985
rs7629105
rs113059383
rs141098993
rs115120340
rs2847728
rs139232605
rs11605185
rs6845106
rs1148464
rs1241497
rs9645299
rs55875437
rs10268587
All
0.0
0.1
0.2
0.3
MR leave−one−out sensitivity analysis for
'Periodontitis' on 'FSC−A on B cell'

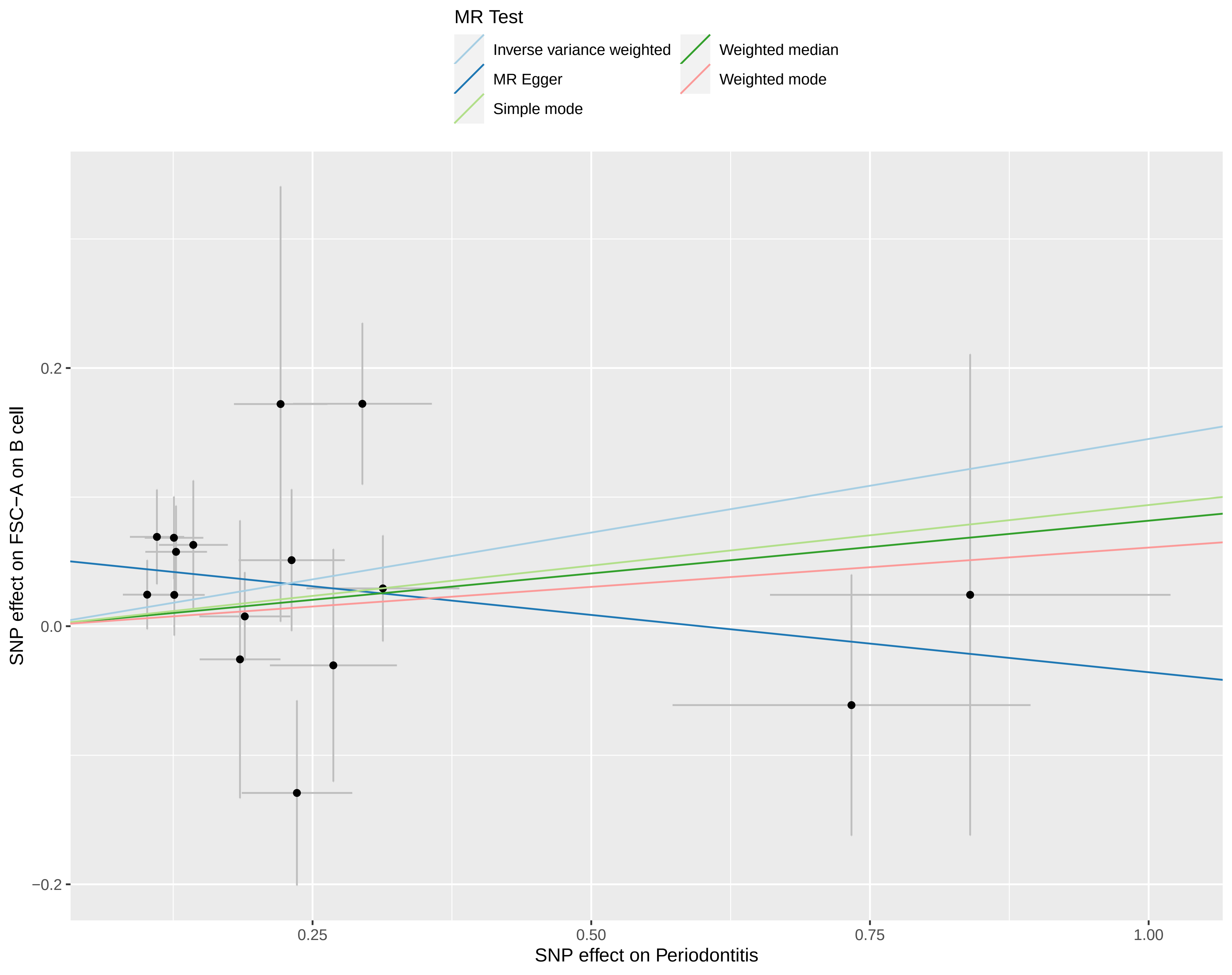
MR Test
Inverse variance weighted
MR Egger
Simple mode
Weighted median
Weighted mode
0.2
0.0
−0.2
0.25
0.50
0.75
1.00
SNP effect on FSC−A on B cell
SNP effect on Periodontitis

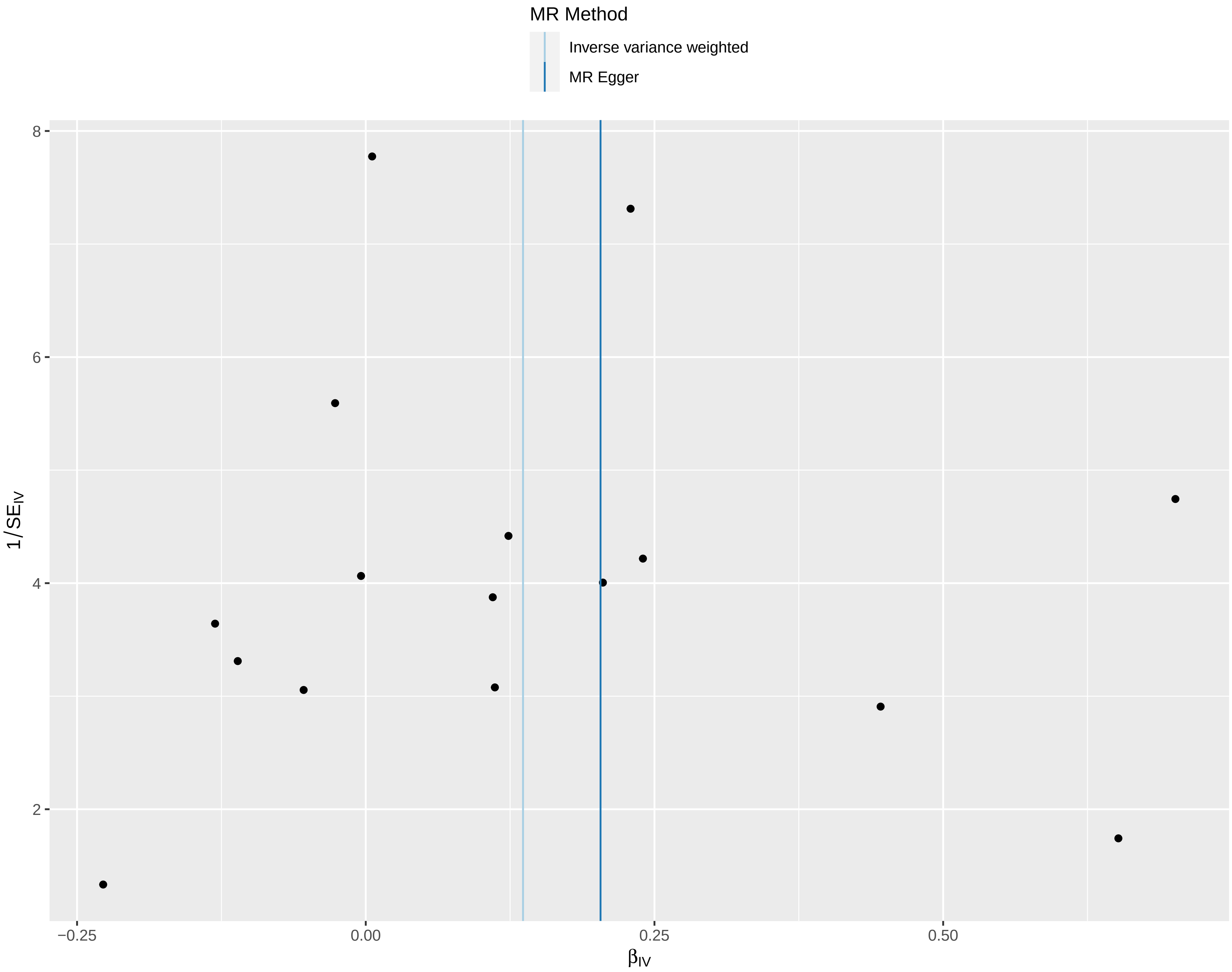

MR Method
Inverse variance weighted
MR Egger
8
6
4
2
$1/SE_{IV}$
−0.25
0.00
0.25
0.50
β_{IV}

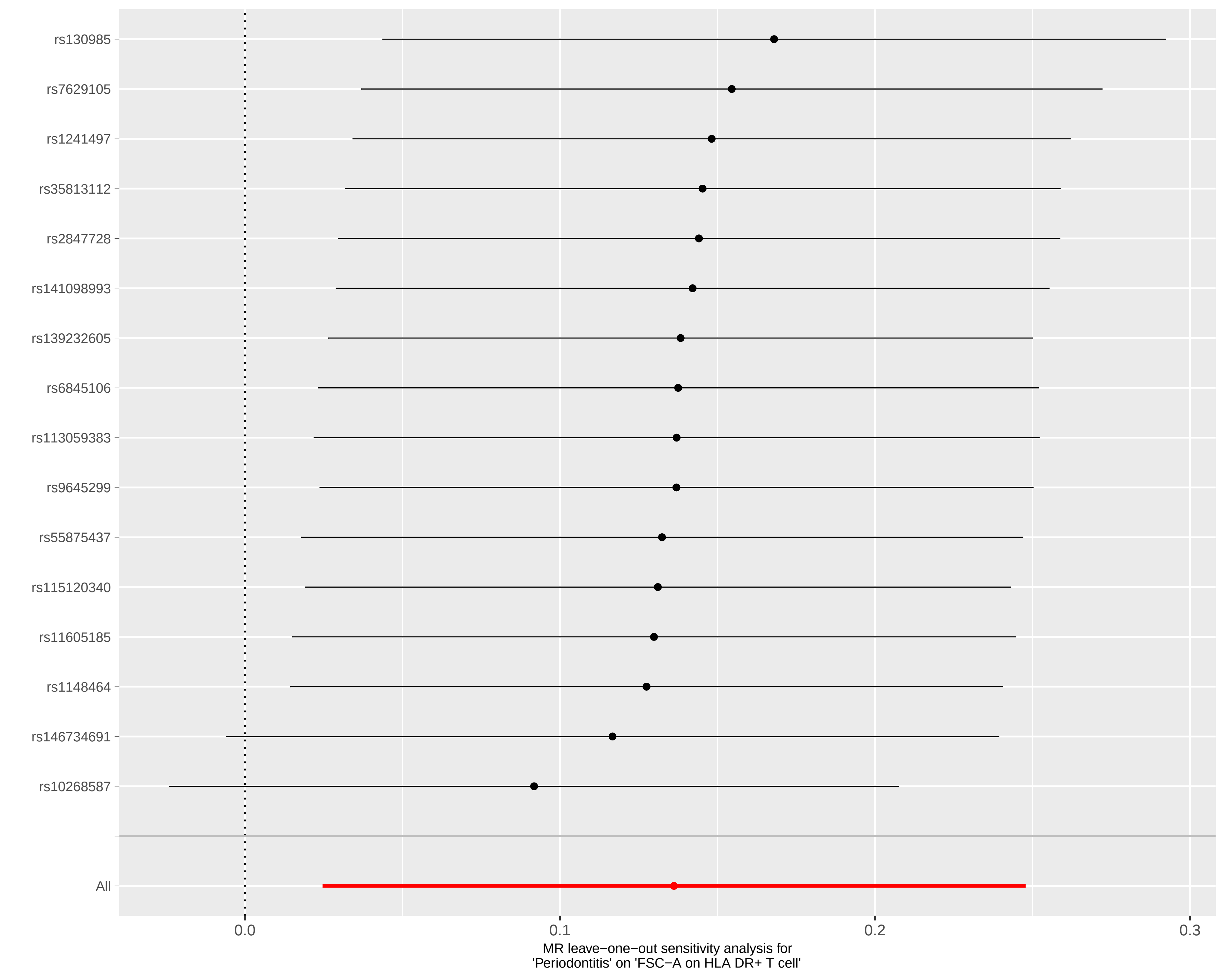
rs130985
rs7629105
rs1241497
rs35813112
rs2847728
rs141098993
rs139232605
rs6845106
rs113059383
rs9645299
rs55875437
rs115120340
rs11605185
rs1148464
rs146734691
rs10268587
All
0.0
0.1
0.2
0.3
MR leave−one−out sensitivity analysis for
'Periodontitis' on 'FSC−A on HLA DR+ T cell'

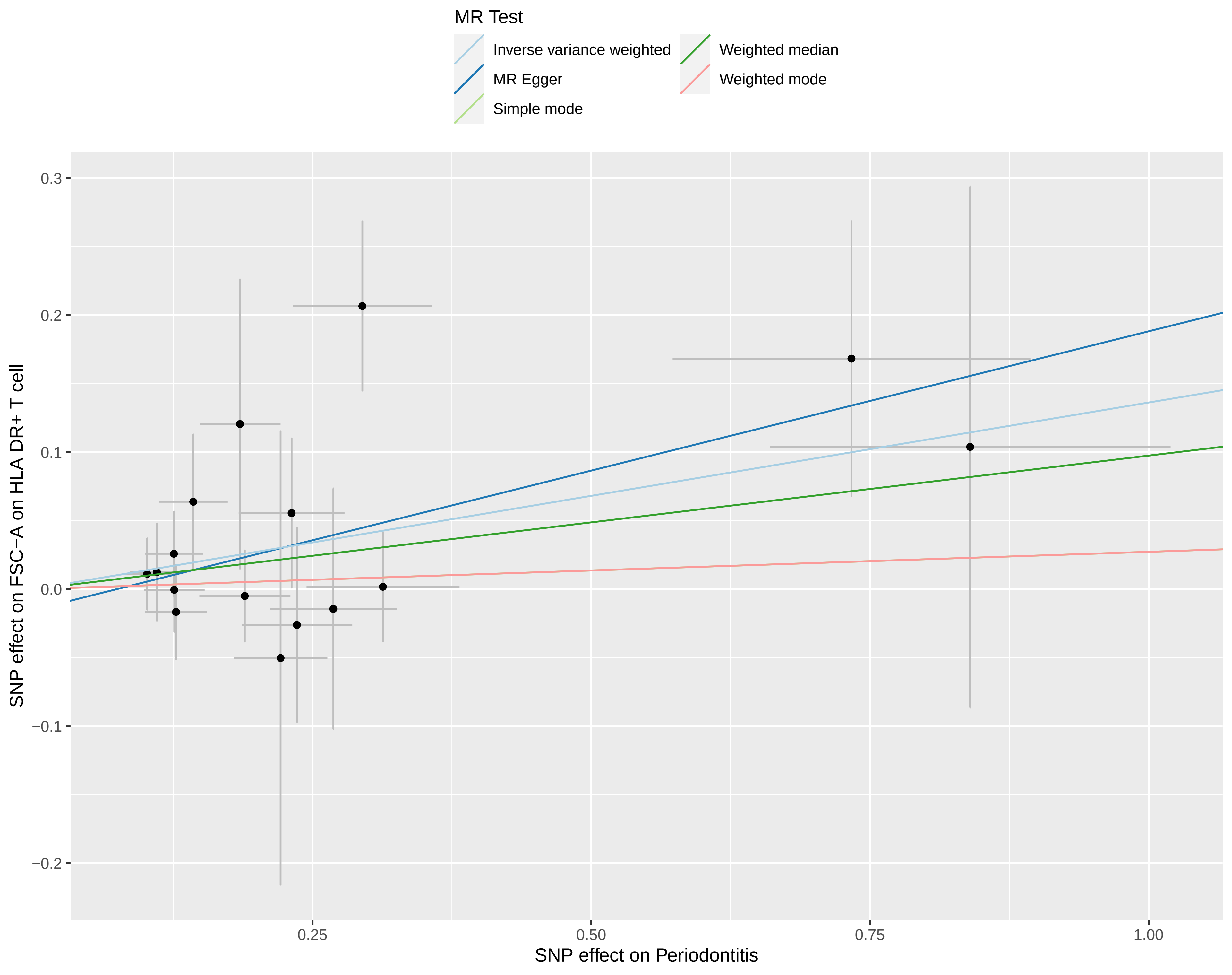

MR Test
Inverse variance weighted
MR Egger
Simple mode
Weighted median
Weighted mode
0.3
0.2
0.1
0.0
−0.1
−0.2
SNP effect on FSC−A on HLA DR+ T cell
0.25
0.50
0.75
1.00
SNP effect on Periodontitis

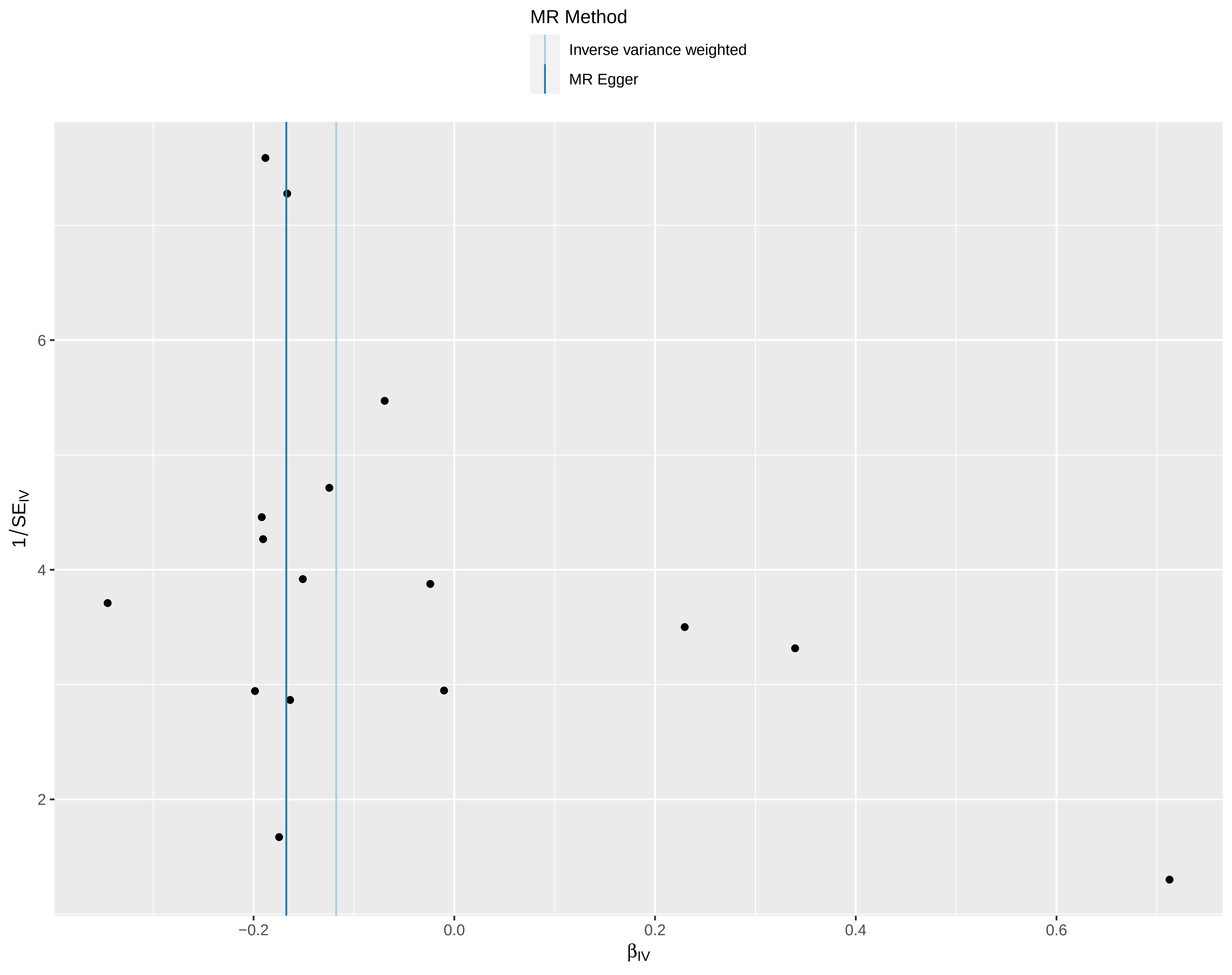

MR Method
Inverse variance weighted
MR Egger
6
4
2
1/SE_IV
−0.2
0.0
0.2
0.4
0.6
β_IV

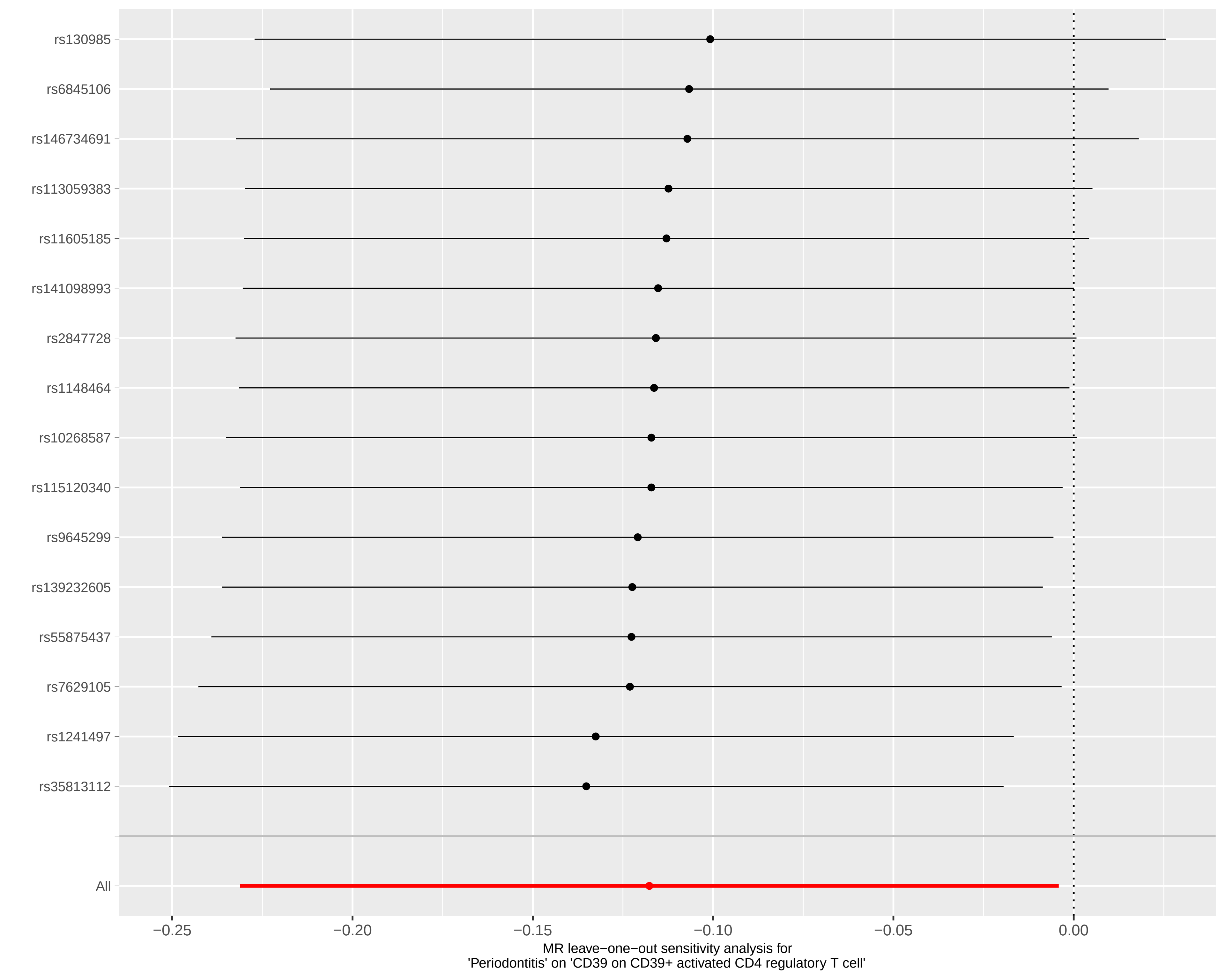
rs130985
rs6845106
rs146734691
rs113059383
rs11605185
rs141098993
rs2847728
rs1148464
rs10268587
rs115120340
rs9645299
rs139232605
rs55875437
rs7629105
rs1241497
rs35813112
All
−0.25
−0.20
−0.15
−0.10
−0.05
0.00
MR leave−one−out sensitivity analysis for
'Periodontitis' on 'CD39 on CD39+ activated CD4 regulatory T cell'

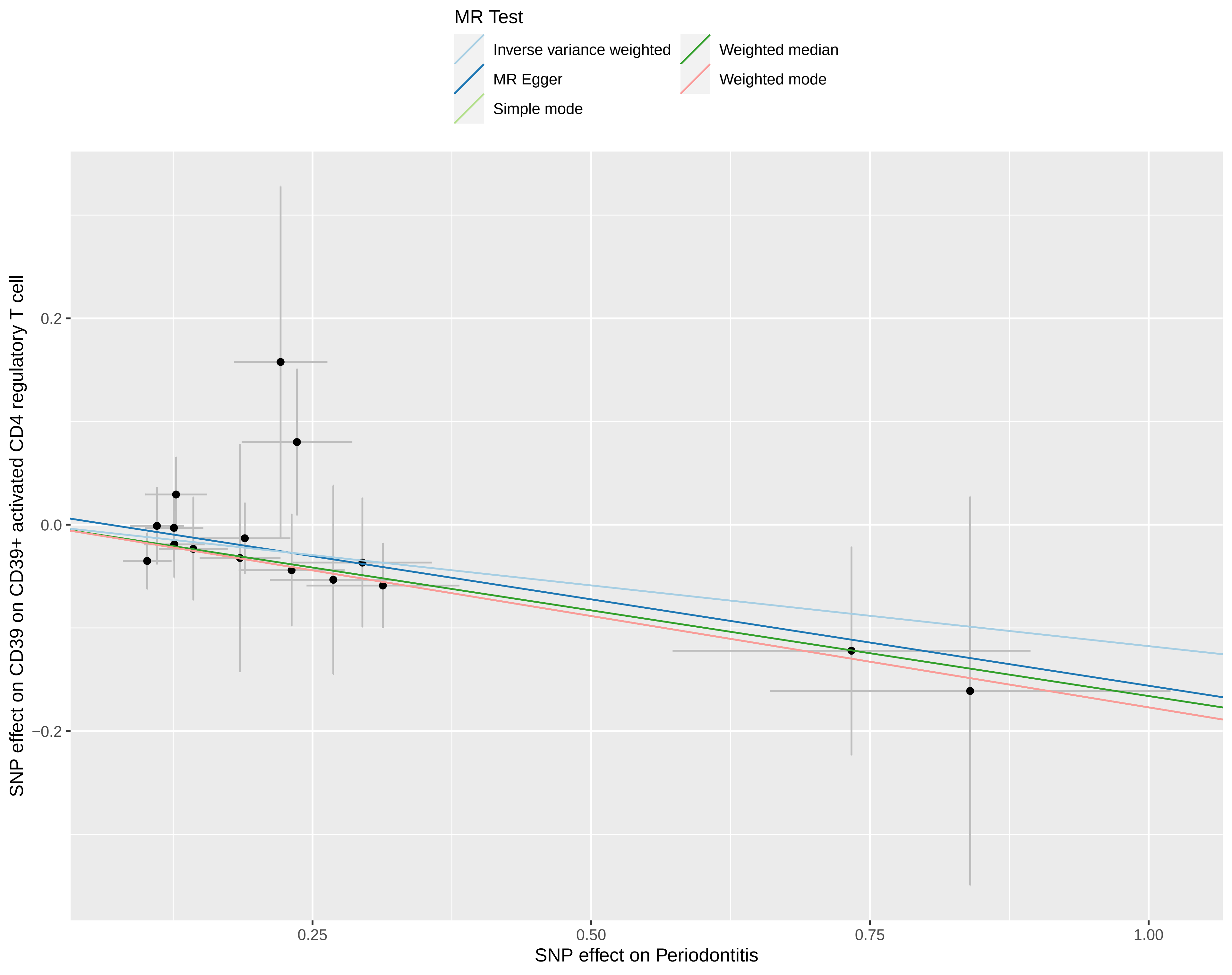

MR Test
Inverse variance weighted
MR Egger
Simple mode
Weighted median
Weighted mode
SNP effect on CD39 on CD39+ activated CD4 regulatory T cell
0.2
0.0
−0.2
SNP effect on Periodontitis
0.25
0.50
0.75
1.00

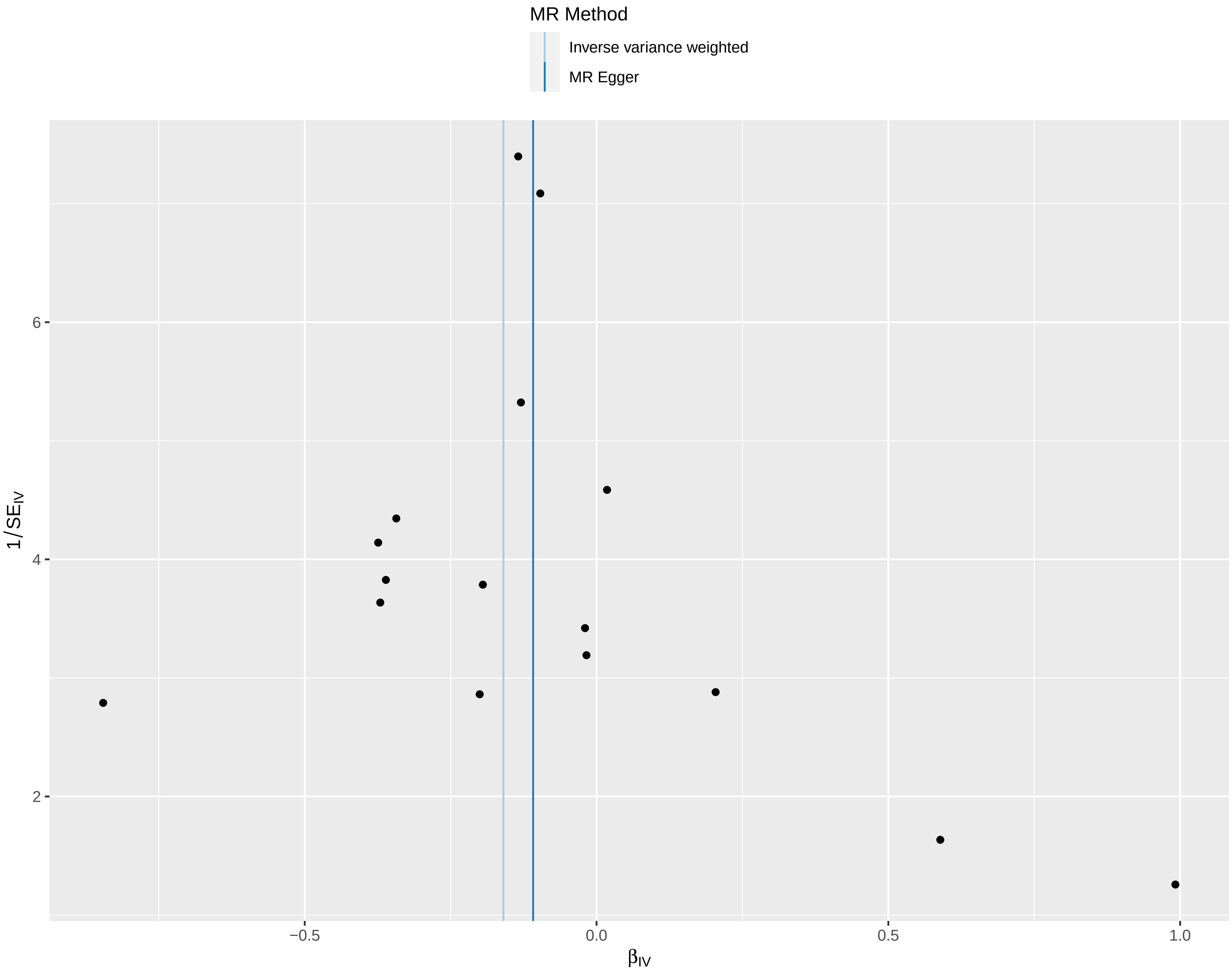

MR Method
Inverse variance weighted
MR Egger
6
4
2
1/SE_IV
−0.5
0.0
0.5
1.0
β_IV

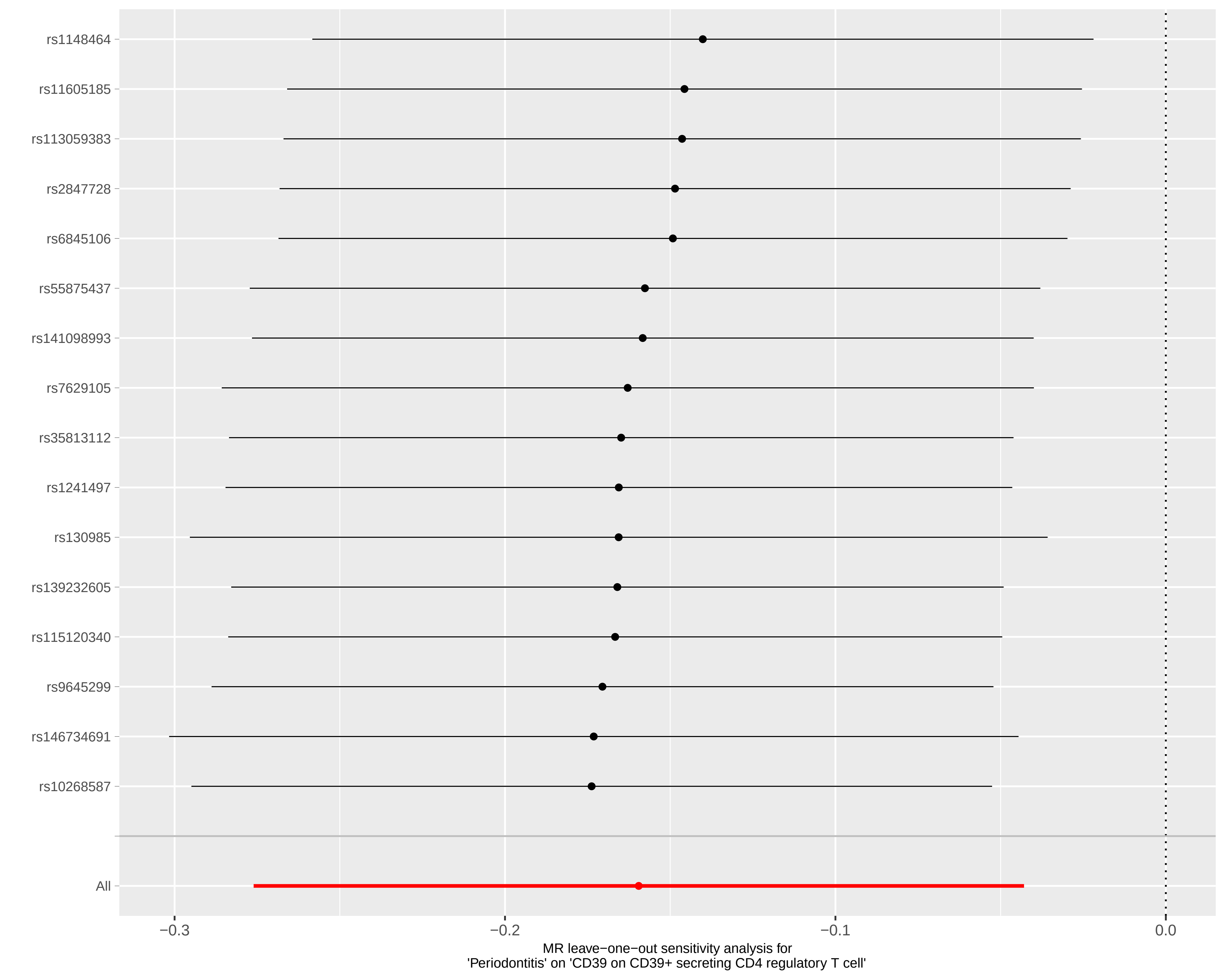
rs1148464
rs11605185
rs113059383
rs2847728
rs6845106
rs55875437
rs141098993
rs7629105
rs35813112
rs1241497
rs130985
rs139232605
rs115120340
rs9645299
rs146734691
rs10268587
All
−0.3
−0.2
−0.1
0.0
MR leave−one−out sensitivity analysis for
'Periodontitis' on 'CD39 on CD39+ secreting CD4 regulatory T cell'

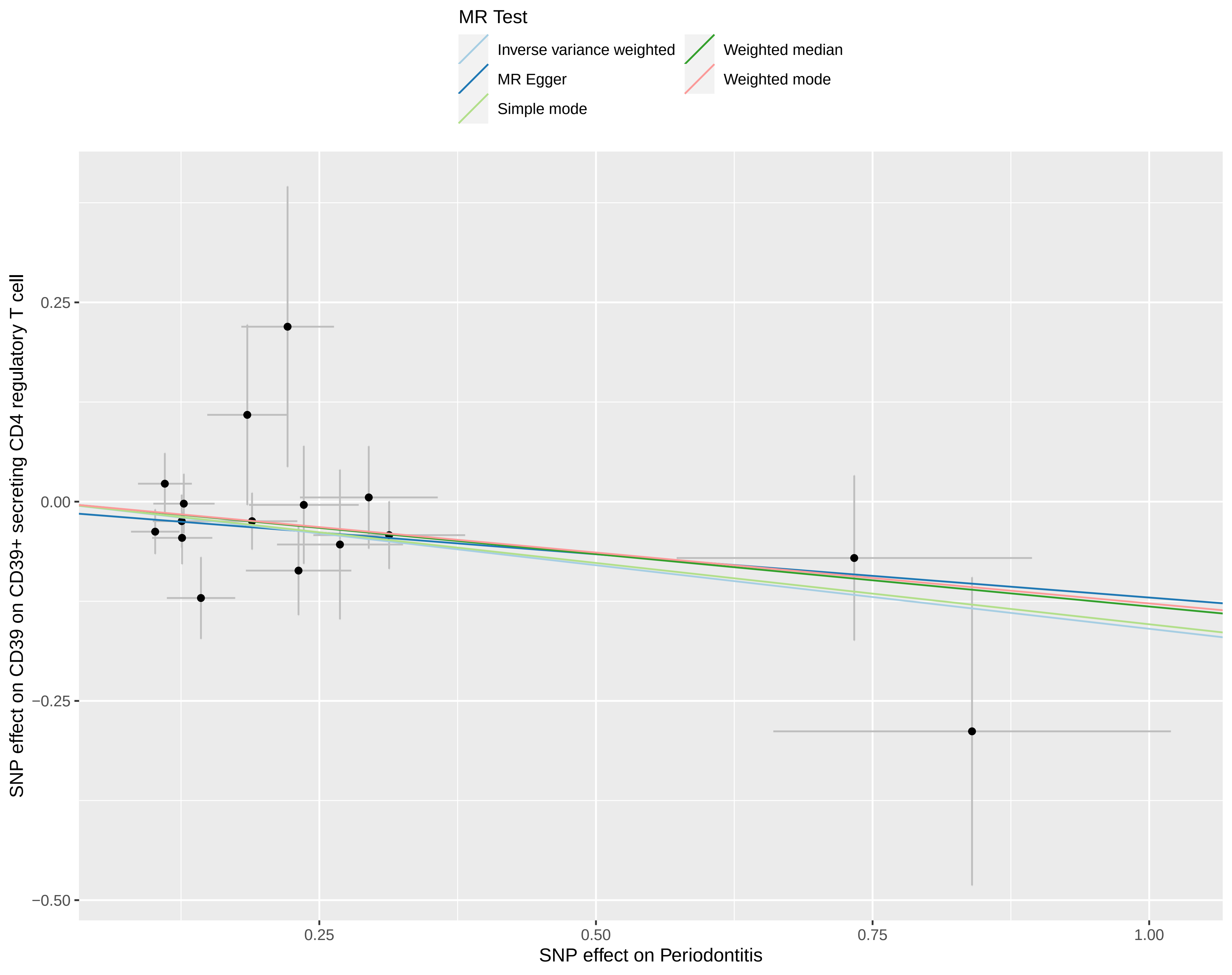
MR Test
Inverse variance weighted
MR Egger
Simple mode
Weighted median
Weighted mode
SNP effect on CD39 on CD39+ secreting CD4 regulatory T cell
0.25
0.00
−0.25
−0.50
0.25
0.50
0.75
1.00
SNP effect on Periodontitis

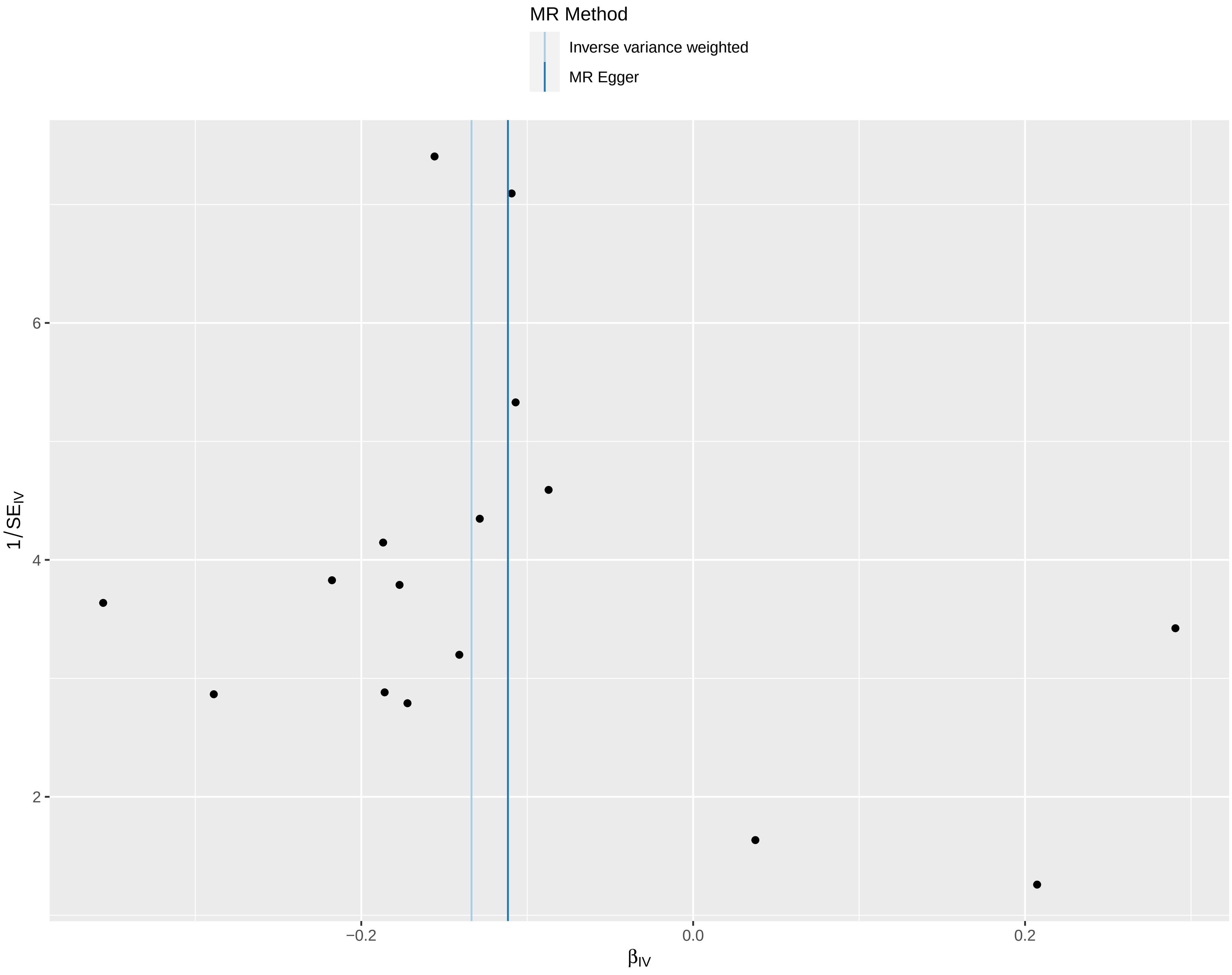
MR Method
Inverse variance weighted
MR Egger
1/SE_IV
6
4
2
−0.2
0.0
0.2
β_IV

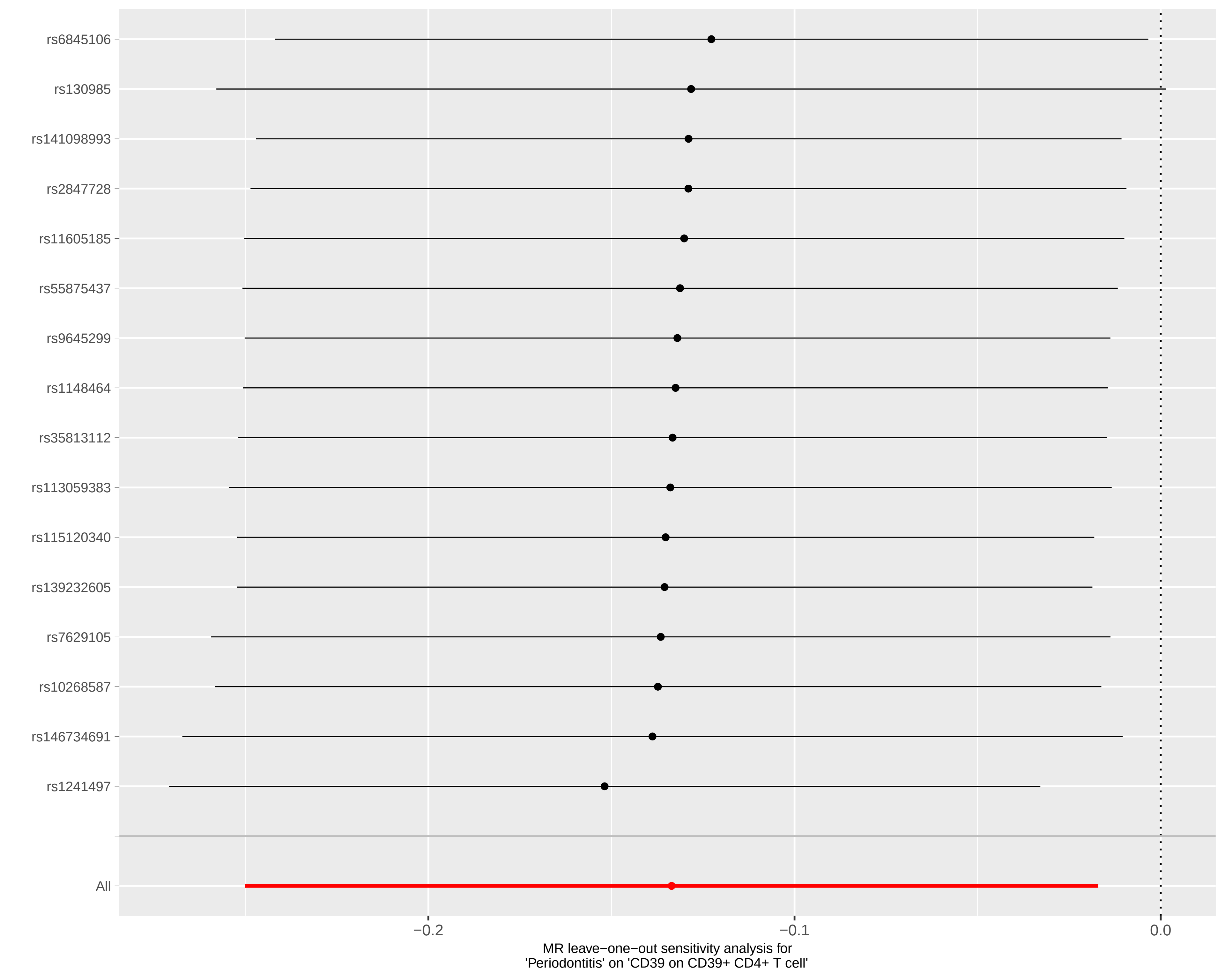

rs6845106
rs130985
rs141098993
rs2847728
rs11605185
rs55875437
rs9645299
rs1148464
rs35813112
rs113059383
rs115120340
rs139232605
rs7629105
rs10268587
rs146734691
rs1241497
All
−0.2
−0.1
0.0
MR leave−one−out sensitivity analysis for
'Periodontitis' on 'CD39 on CD39+ CD4+ T cell'

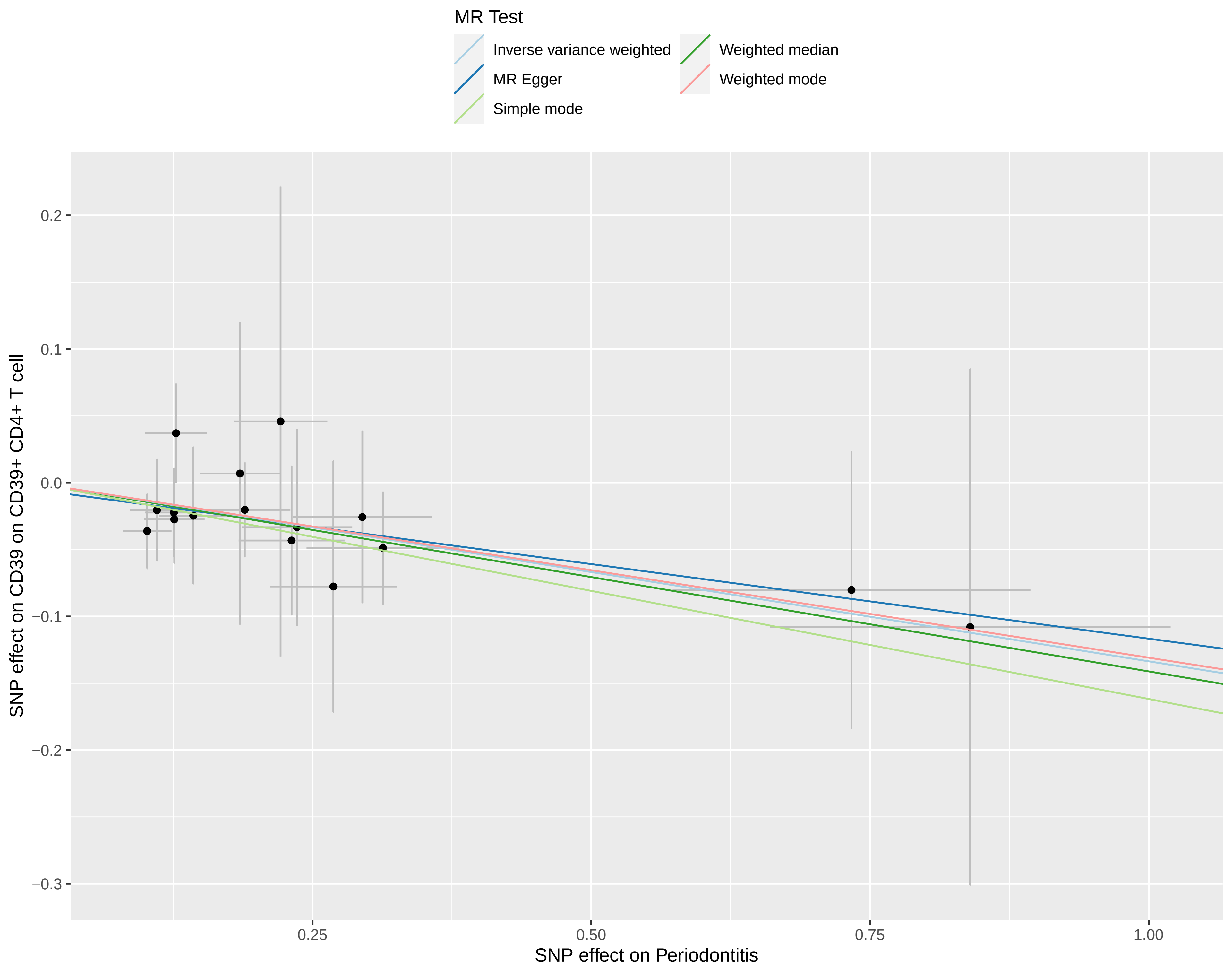
MR Test
Inverse variance weighted
MR Egger
Simple mode
Weighted median
Weighted mode
SNP effect on CD39 on CD39+ CD4+ T cell
SNP effect on Periodontitis
0.2
0.1
0.0
−0.1
−0.2
−0.3
0.25
0.50
0.75
1.00

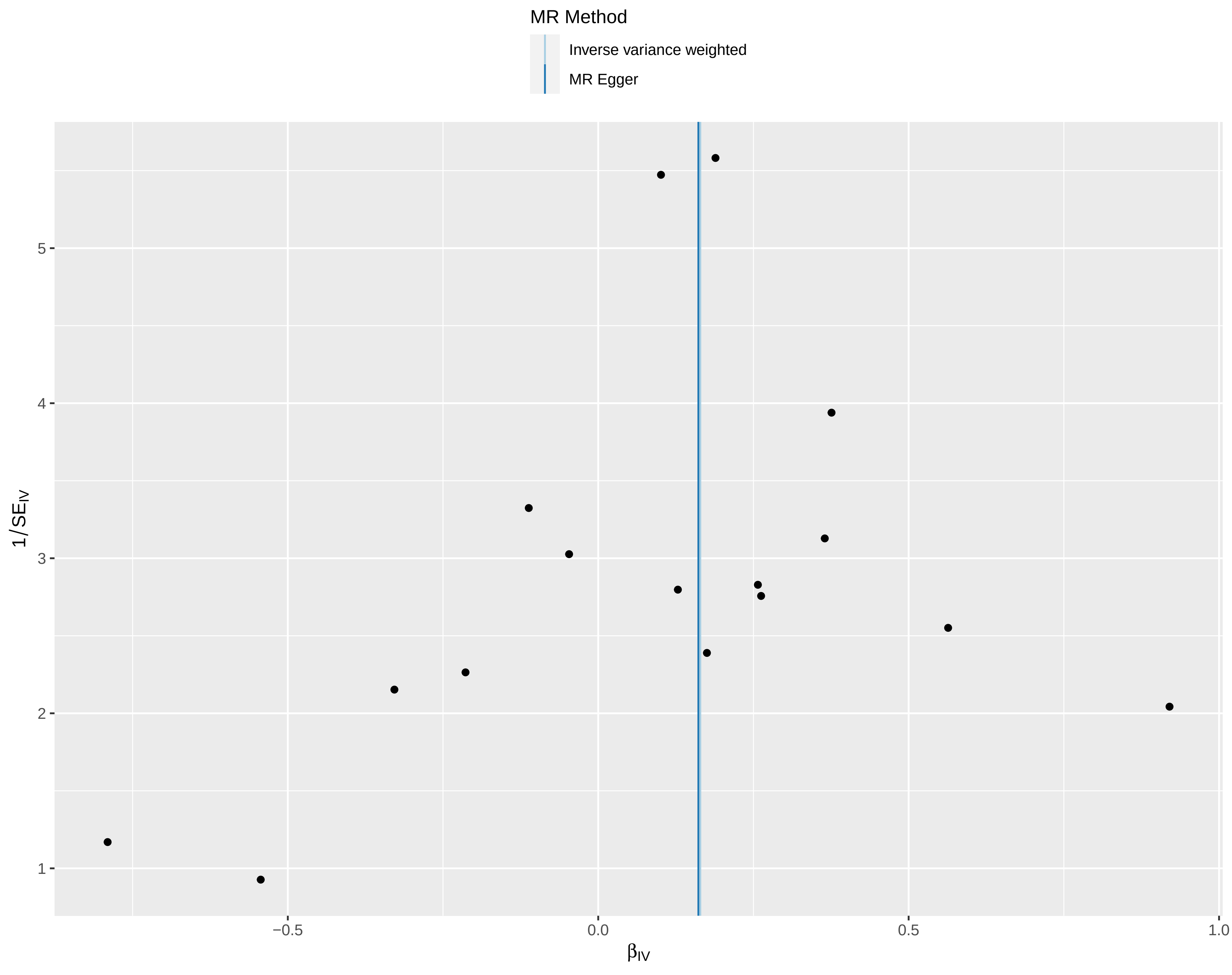
MR Method
Inverse variance weighted
MR Egger
1/SE_IV
β_IV
5
4
3
2
1
−0.5
0.0
0.5
1.0

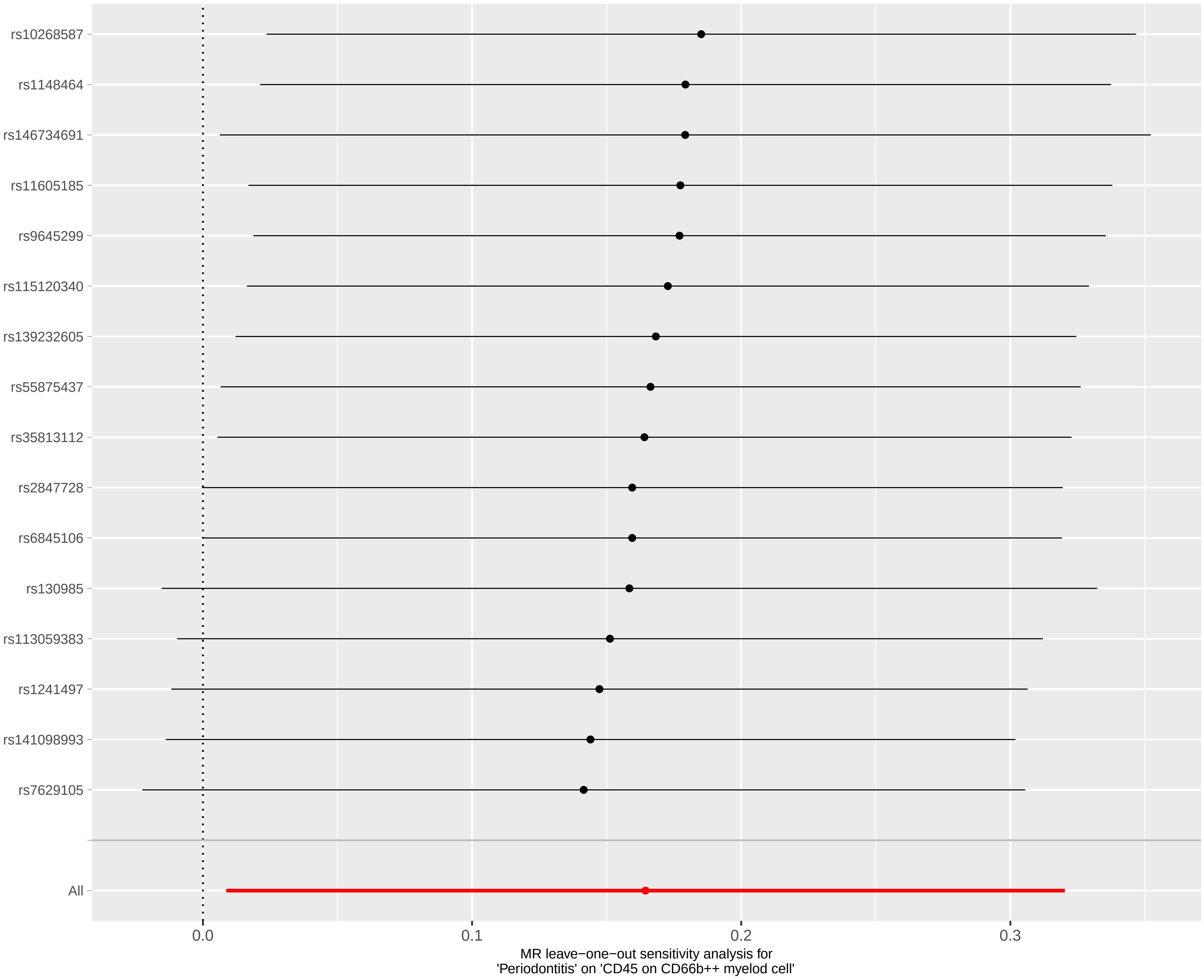

rs10268587
rs1148464
rs146734691
rs11605185
rs9645299
rs115120340
rs139232605
rs55875437
rs35813112
rs2847728
rs6845106
rs130985
rs113059383
rs1241497
rs141098993
rs7629105
All
0.0
0.1
0.2
0.3
MR leave−one−out sensitivity analysis for
'Periodontitis' on 'CD45 on CD66b++ myelod cell'

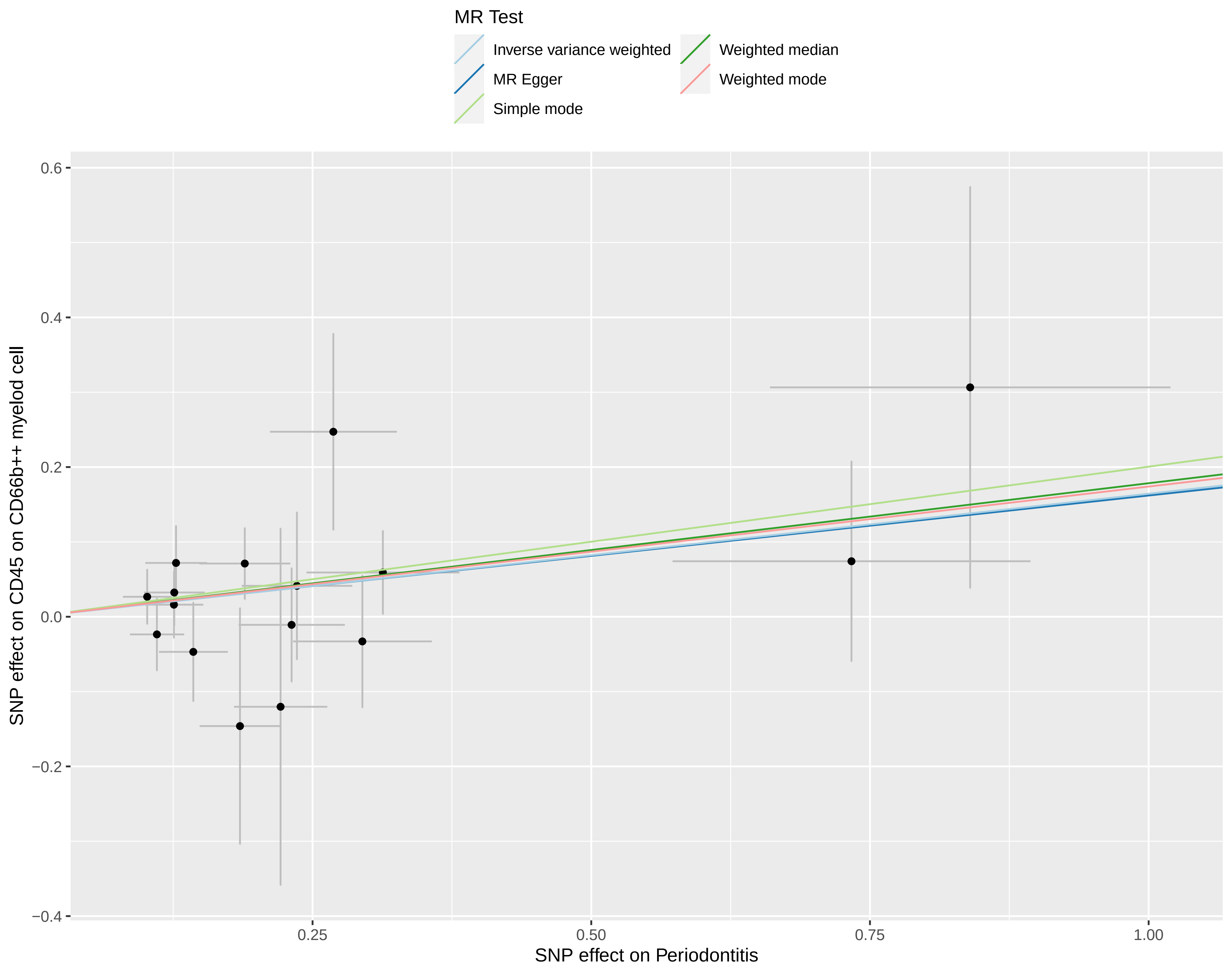

MR Test
Inverse variance weighted
MR Egger
Simple mode
Weighted median
Weighted mode
SNP effect on CD45 on CD66b++ myelod cell
0.6
0.4
0.2
0.0
−0.2
−0.4
0.25
0.50
0.75
1.00
SNP effect on Periodontitis

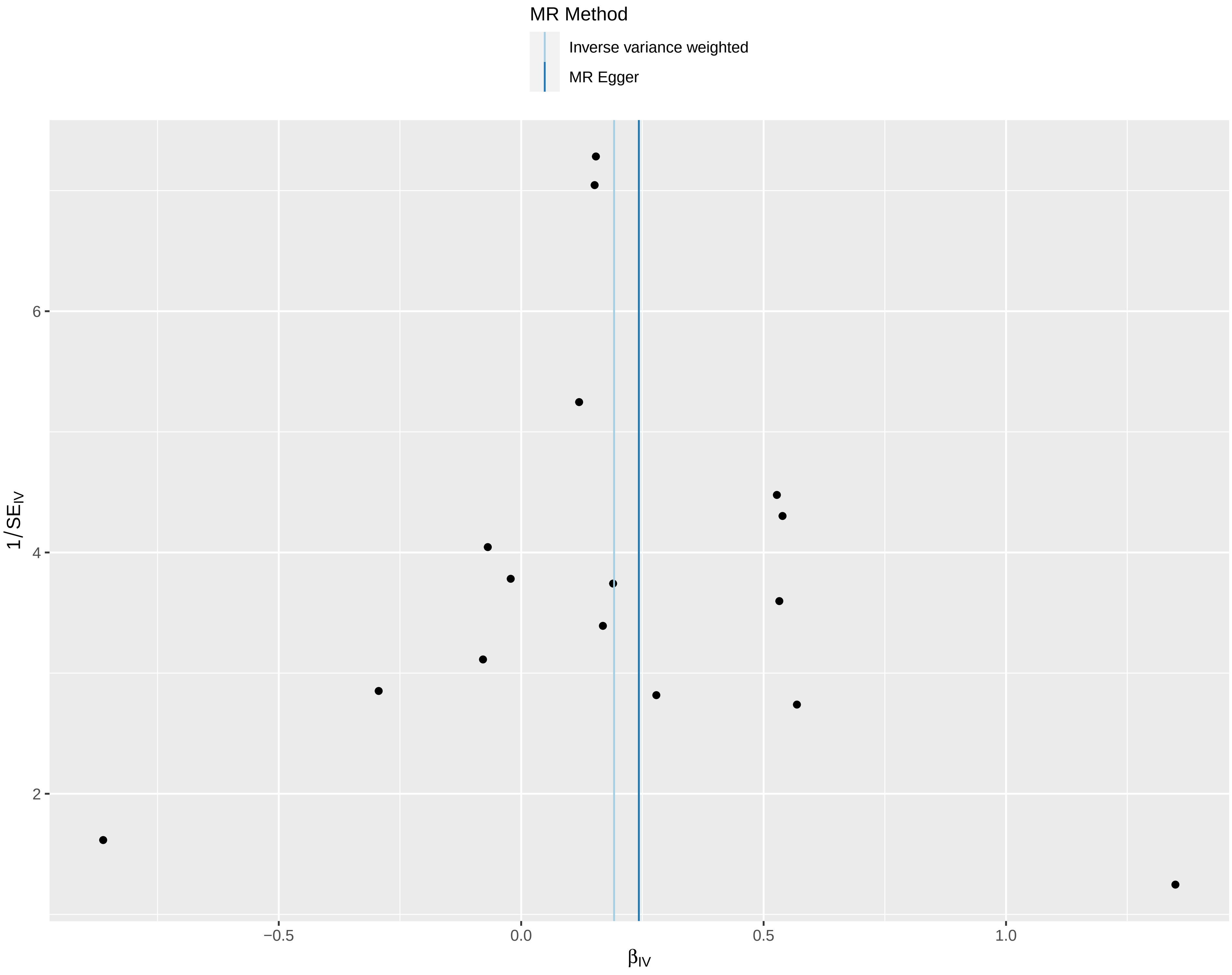
MR Method
Inverse variance weighted
MR Egger
1/SE_IV
6
4
2
−0.5
0.0
0.5
1.0
β_IV

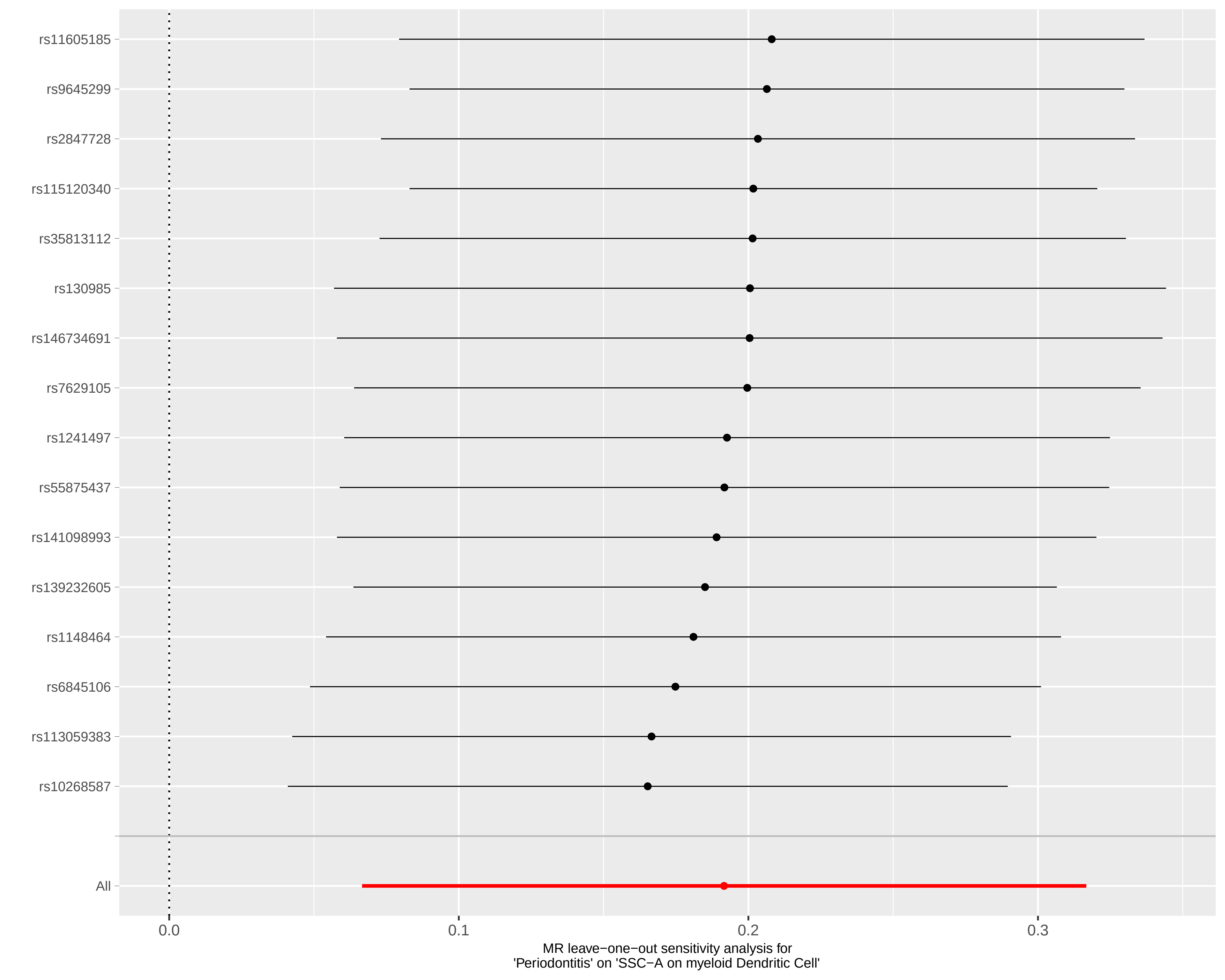

rs11605185
rs9645299
rs2847728
rs115120340
rs35813112
rs130985
rs146734691
rs7629105
rs1241497
rs55875437
rs141098993
rs139232605
rs1148464
rs6845106
rs113059383
rs10268587
All
0.0
0.1
0.2
0.3
MR leave−one−out sensitivity analysis for
'Periodontitis' on 'SSC−A on myeloid Dendritic Cell'

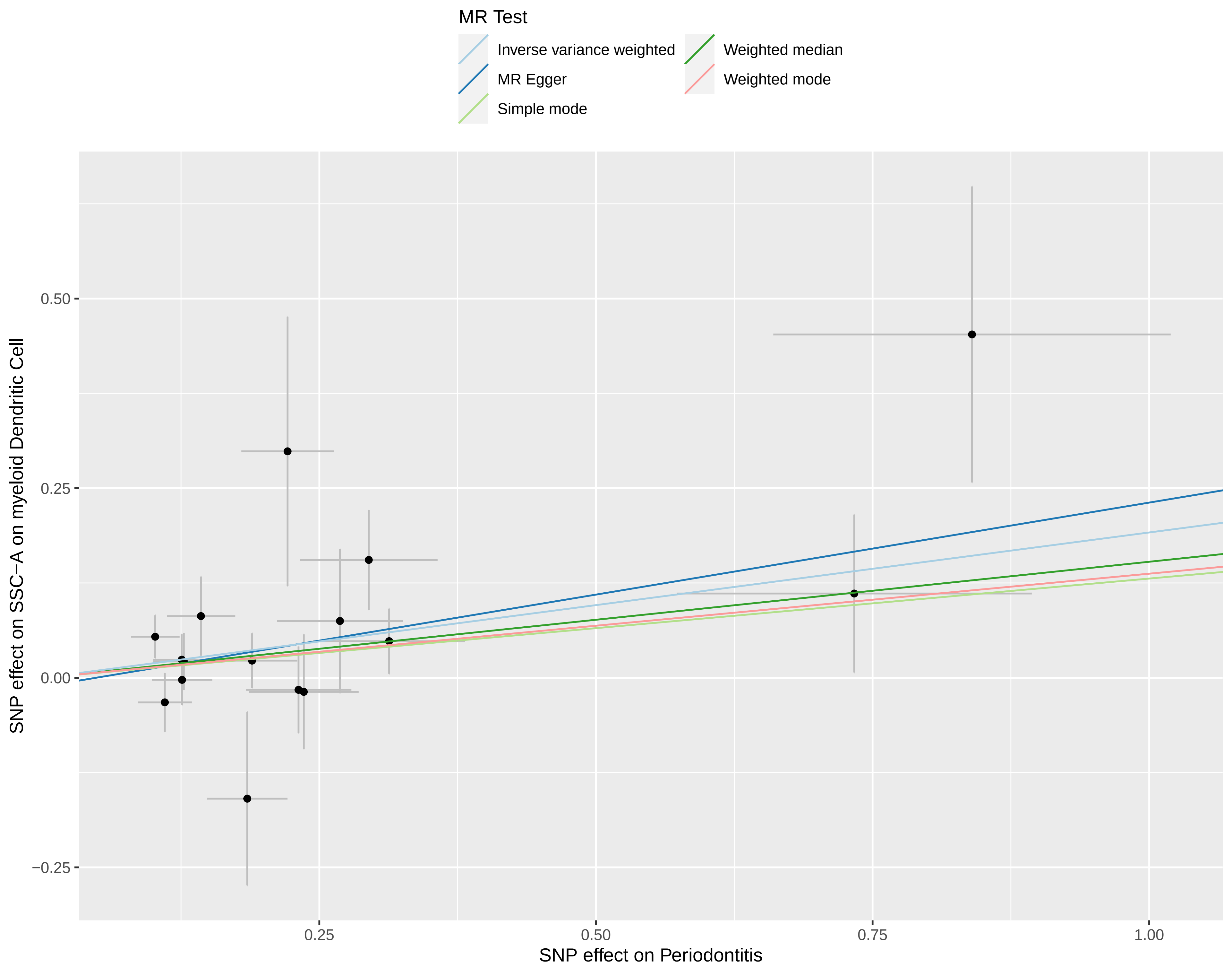
MR Test
Inverse variance weighted
MR Egger
Simple mode
Weighted median
Weighted mode
SNP effect on SSC−A on myeloid Dendritic Cell
0.50
0.25
0.00
−0.25
0.25
0.50
0.75
1.00
SNP effect on Periodontitis

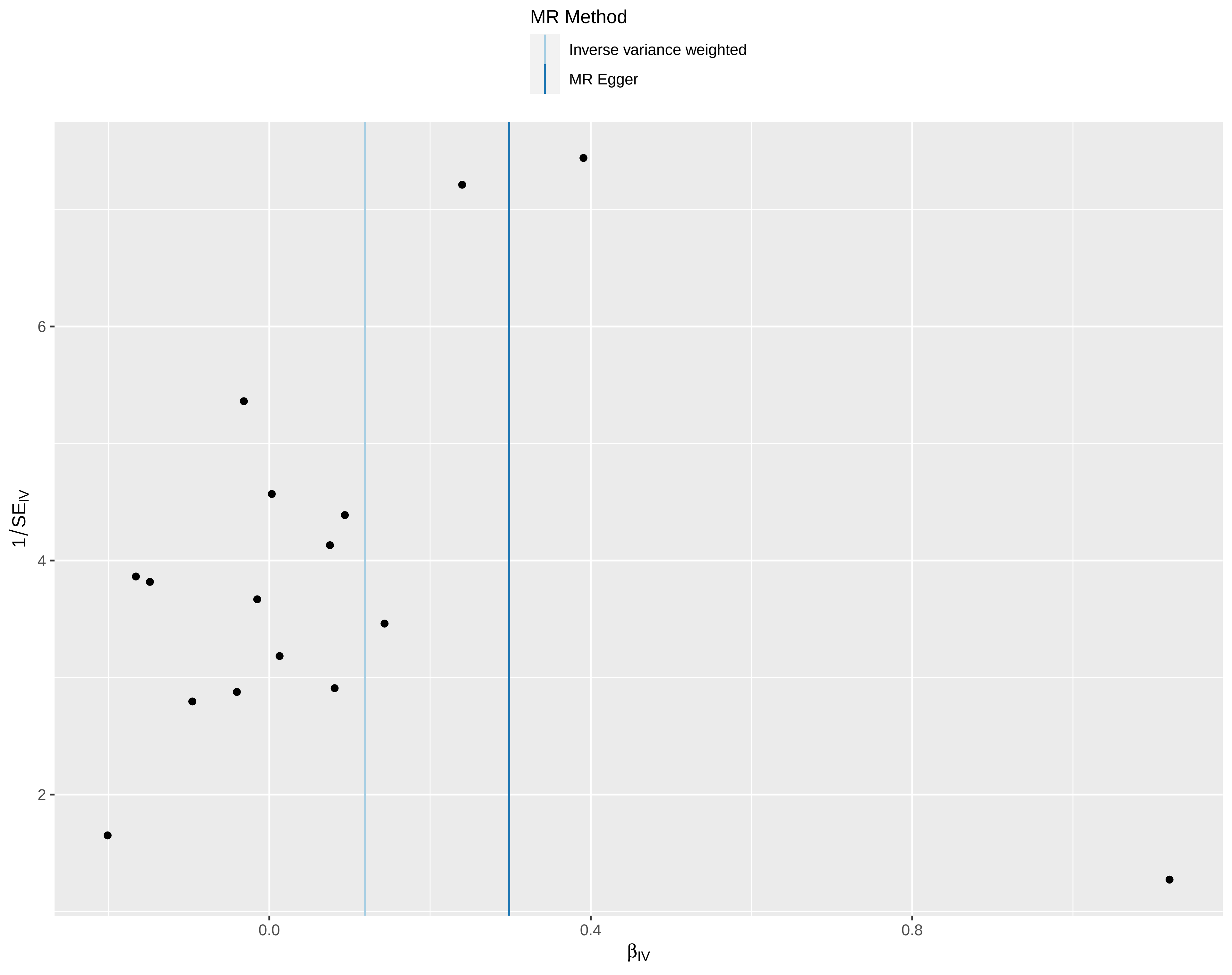
MR Method
Inverse variance weighted
MR Egger
6
4
2
1/SE$_{IV}$
0.0
0.4
0.8
β$_{IV}$

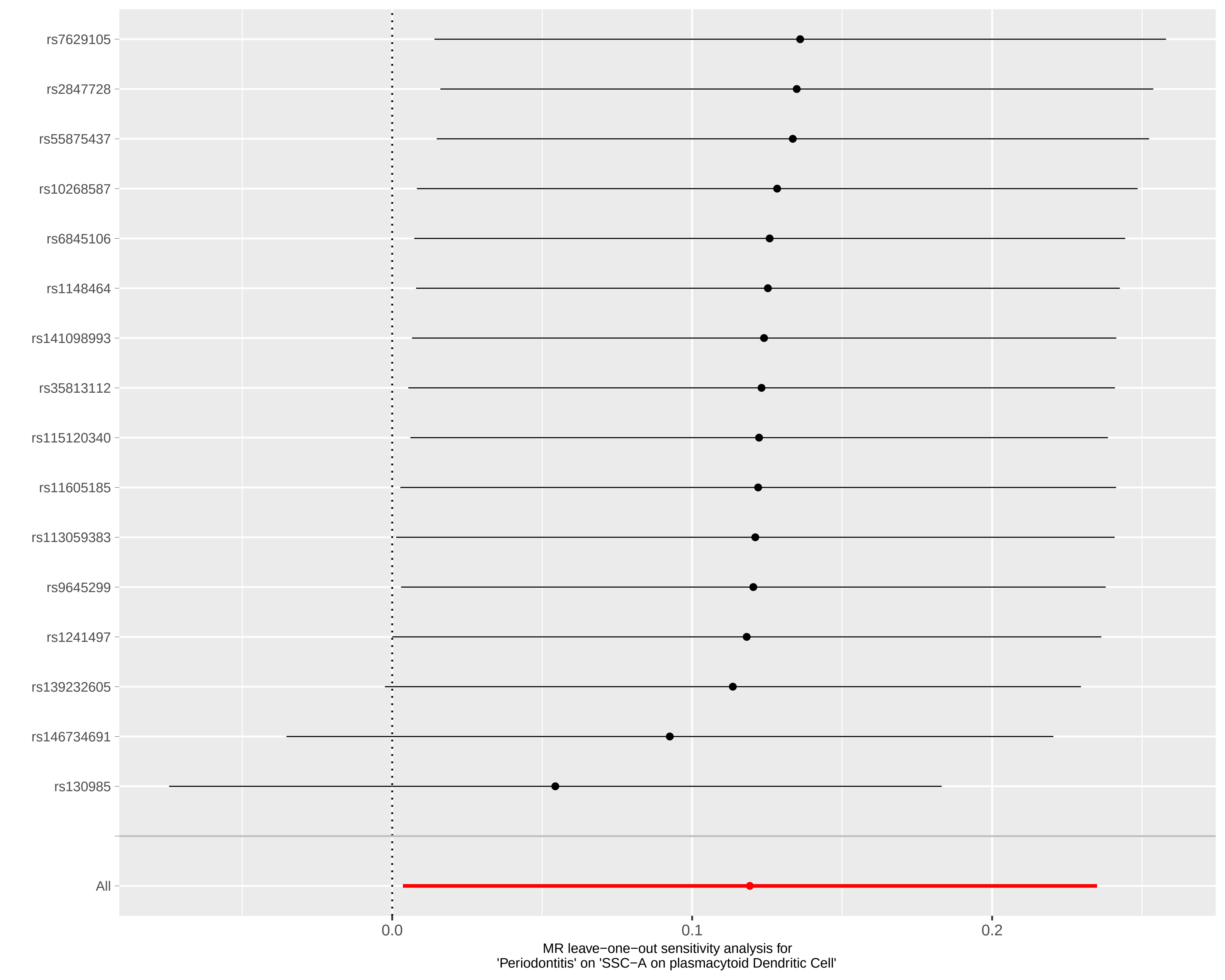
rs7629105
rs2847728
rs55875437
rs10268587
rs6845106
rs1148464
rs141098993
rs35813112
rs115120340
rs11605185
rs113059383
rs9645299
rs1241497
rs139232605
rs146734691
rs130985
All
0.0
0.1
0.2
MR leave−one−out sensitivity analysis for
'Periodontitis' on 'SSC−A on plasmacytoid Dendritic Cell'

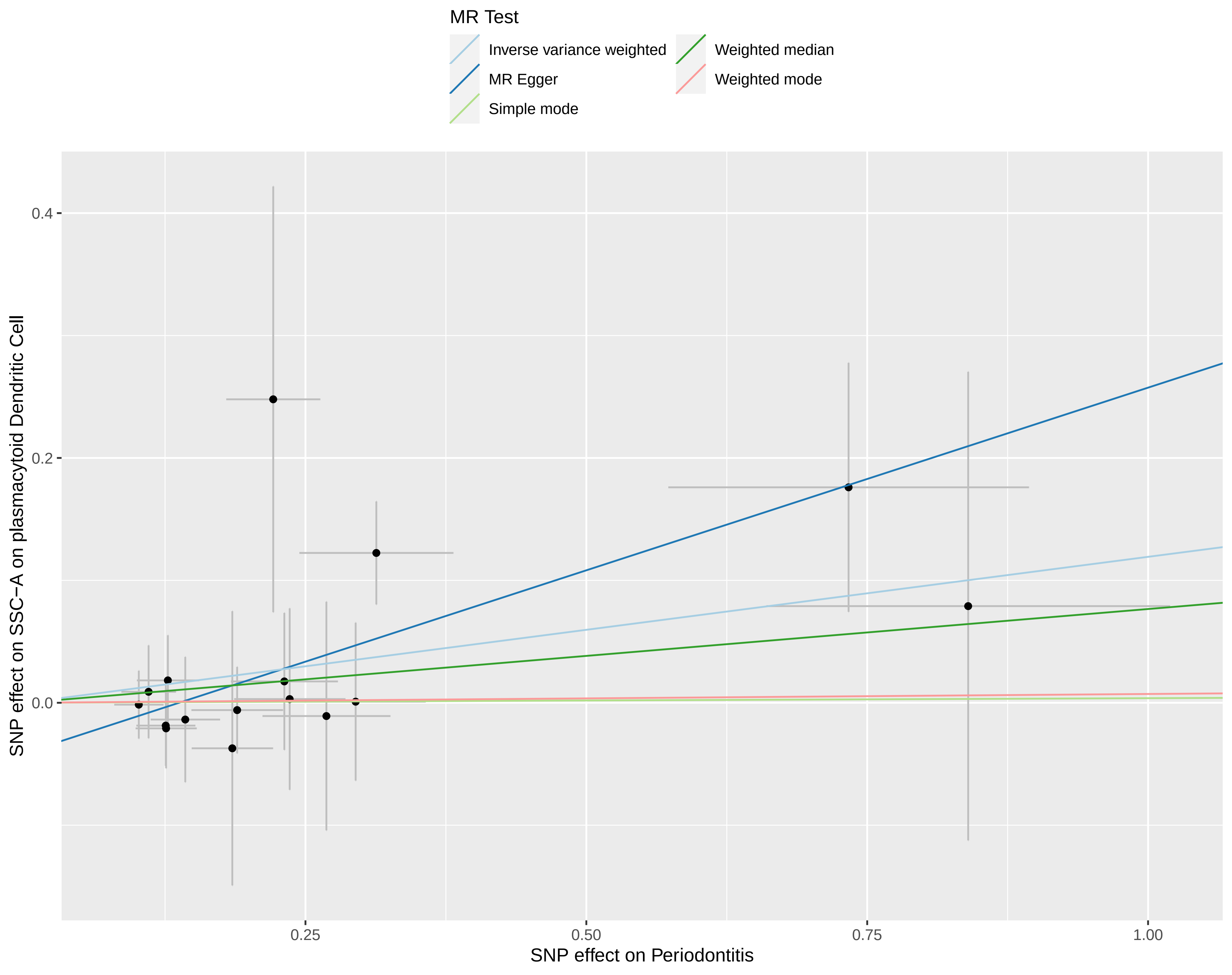

MR Test
Inverse variance weighted
MR Egger
Simple mode
Weighted median
Weighted mode
0.4
0.2
0.0
SNP effect on SSC−A on plasmacytoid Dendritic Cell
0.25
0.50
0.75
1.00
SNP effect on Periodontitis

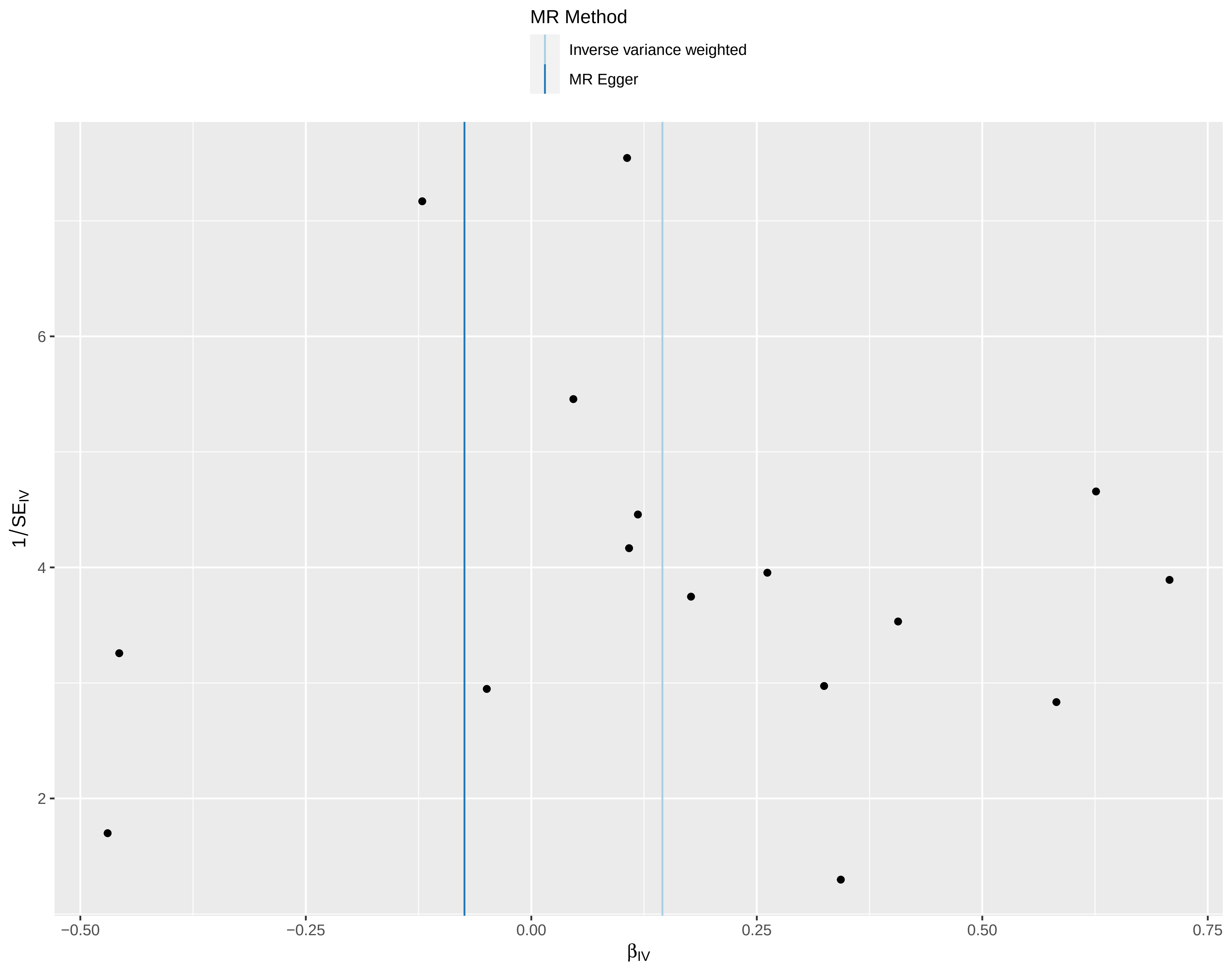

MR Method
Inverse variance weighted
MR Egger
1/SE_IV
6
4
2
−0.50
−0.25
0.00
0.25
0.50
0.75
β_IV

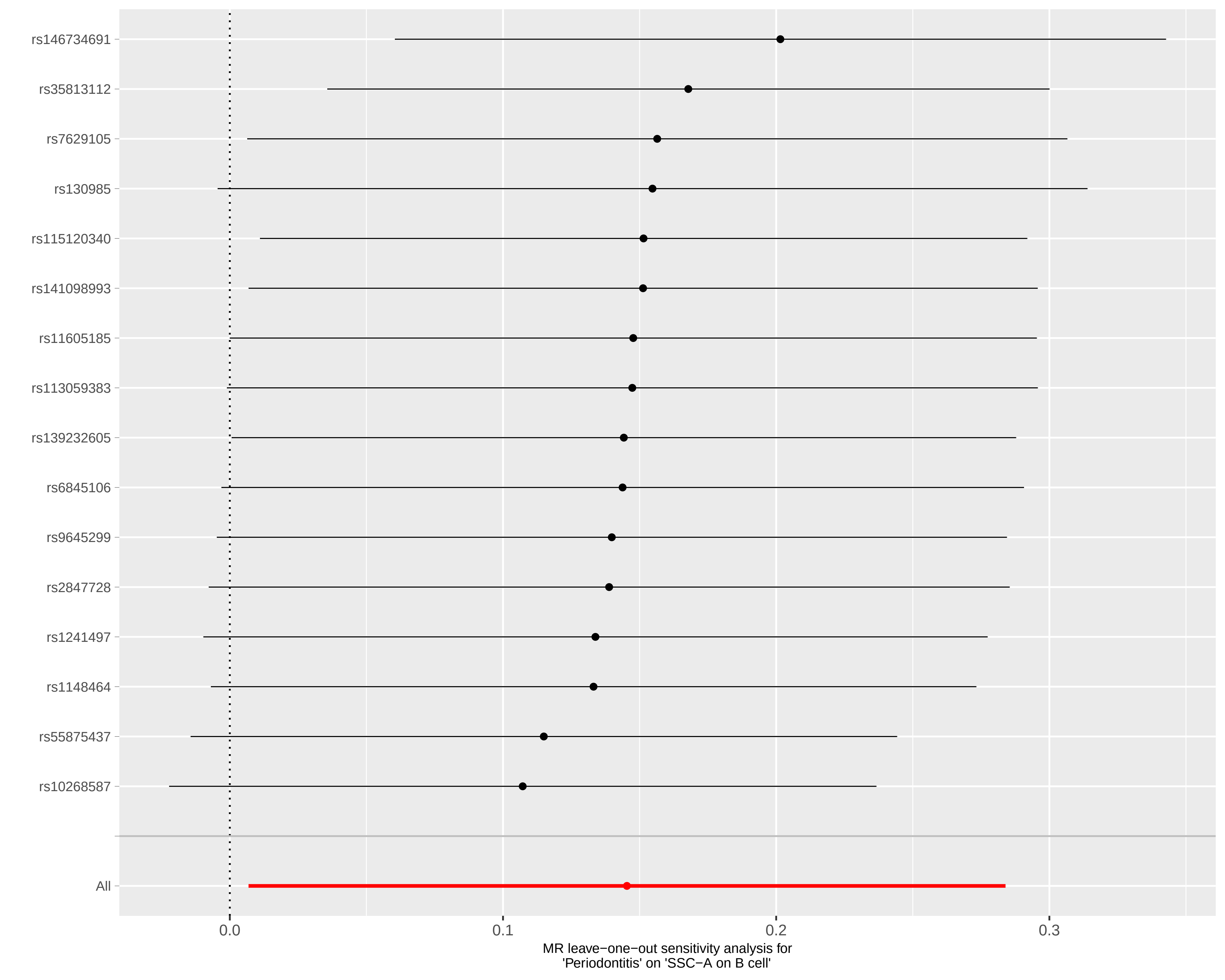
rs146734691
rs35813112
rs7629105
rs130985
rs115120340
rs141098993
rs11605185
rs113059383
rs139232605
rs6845106
rs9645299
rs2847728
rs1241497
rs1148464
rs55875437
rs10268587
All
0.0
0.1
0.2
0.3
MR leave−one−out sensitivity analysis for
'Periodontitis' on 'SSC−A on B cell'

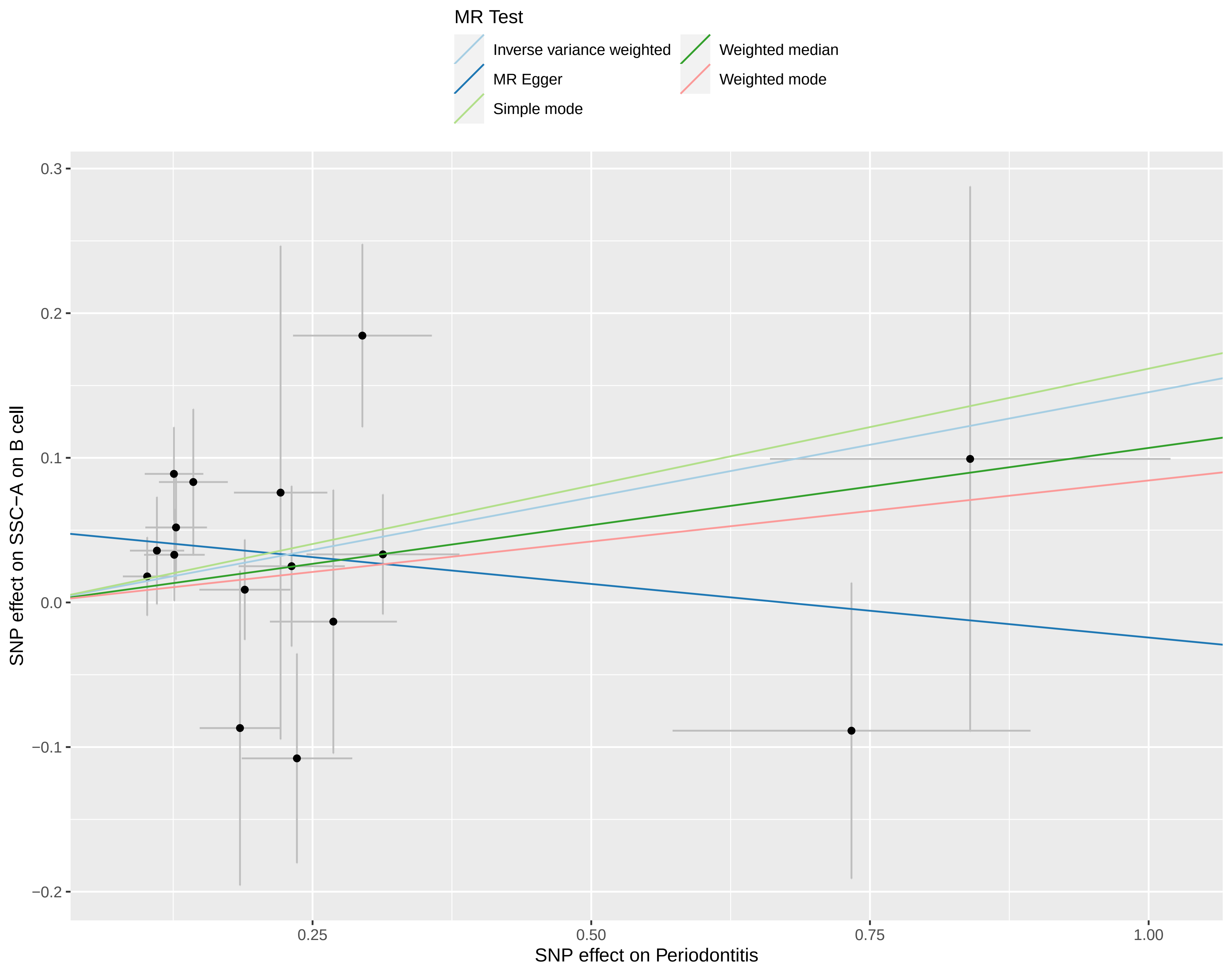

MR Test
Inverse variance weighted
MR Egger
Simple mode
Weighted median
Weighted mode
SNP effect on SSC−A on B cell
SNP effect on Periodontitis
0.3
0.2
0.1
0.0
−0.1
−0.2
0.25
0.50
0.75
1.00

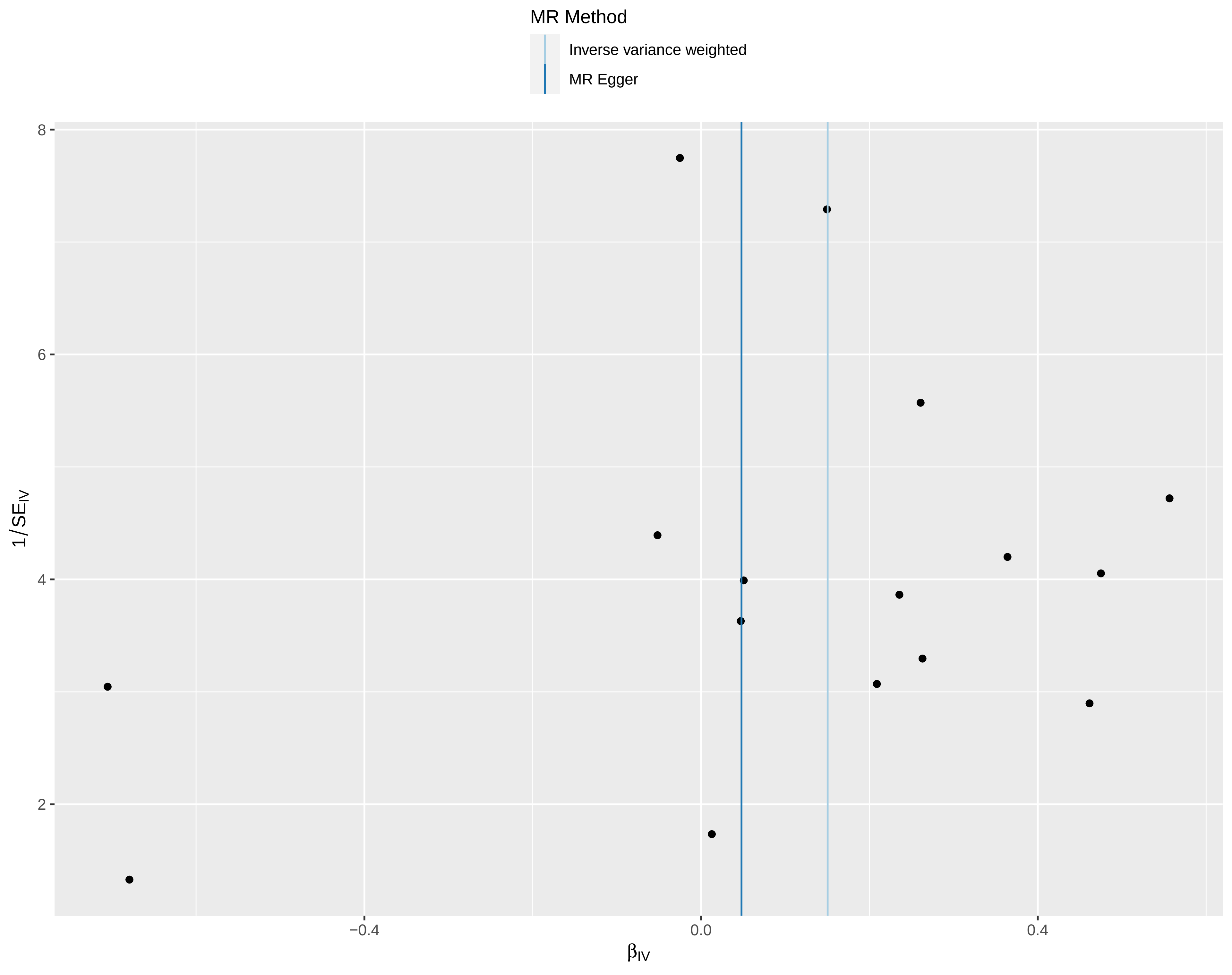

MR Method
Inverse variance weighted
MR Egger
8
6
4
2
1/SE_IV
−0.4
0.0
0.4
β_IV

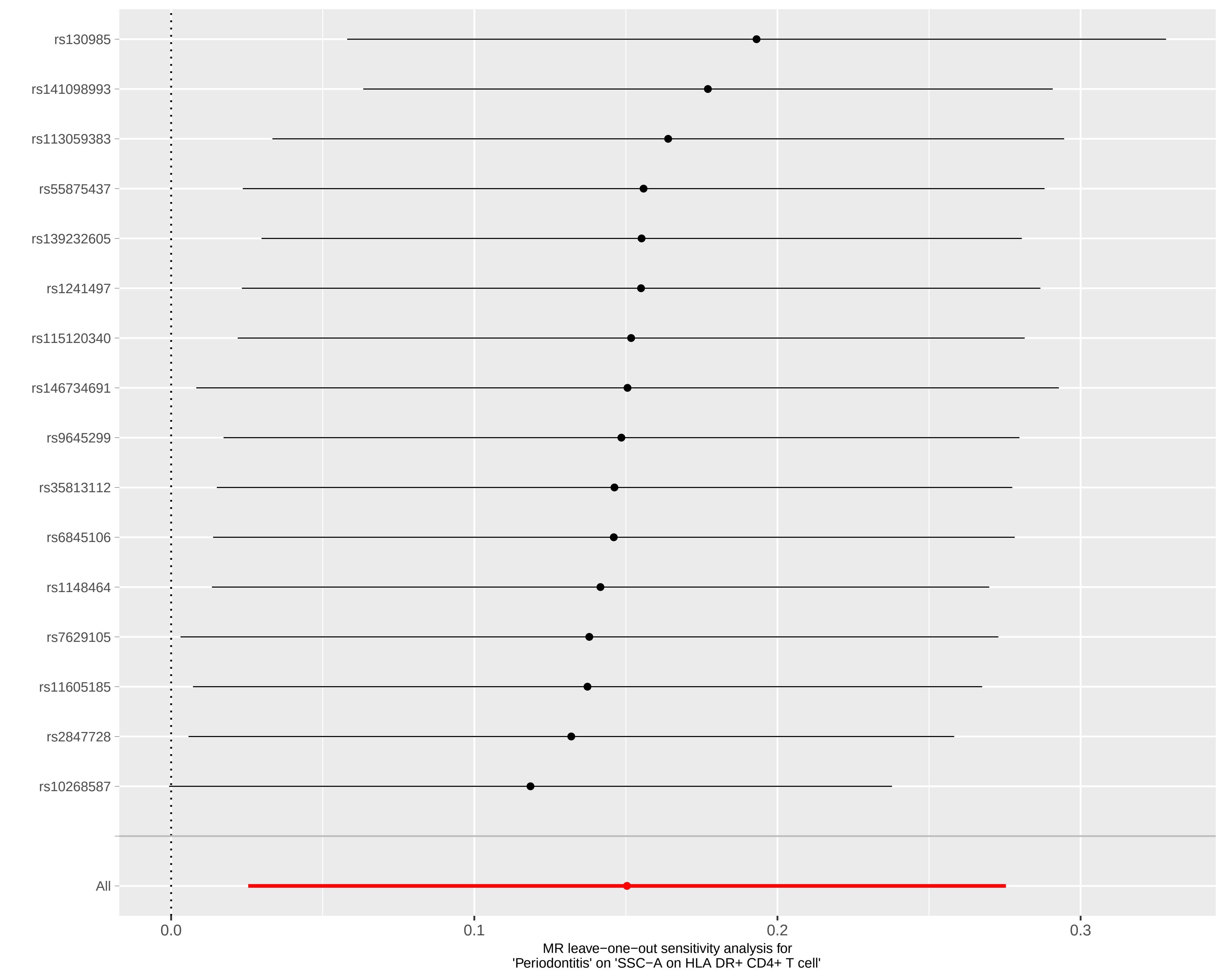

rs130985
rs141098993
rs113059383
rs55875437
rs139232605
rs1241497
rs115120340
rs146734691
rs9645299
rs35813112
rs6845106
rs1148464
rs7629105
rs11605185
rs2847728
rs10268587
All
0.0
0.1
0.2
0.3
MR leave−one−out sensitivity analysis for
'Periodontitis' on 'SSC−A on HLA DR+ CD4+ T cell'

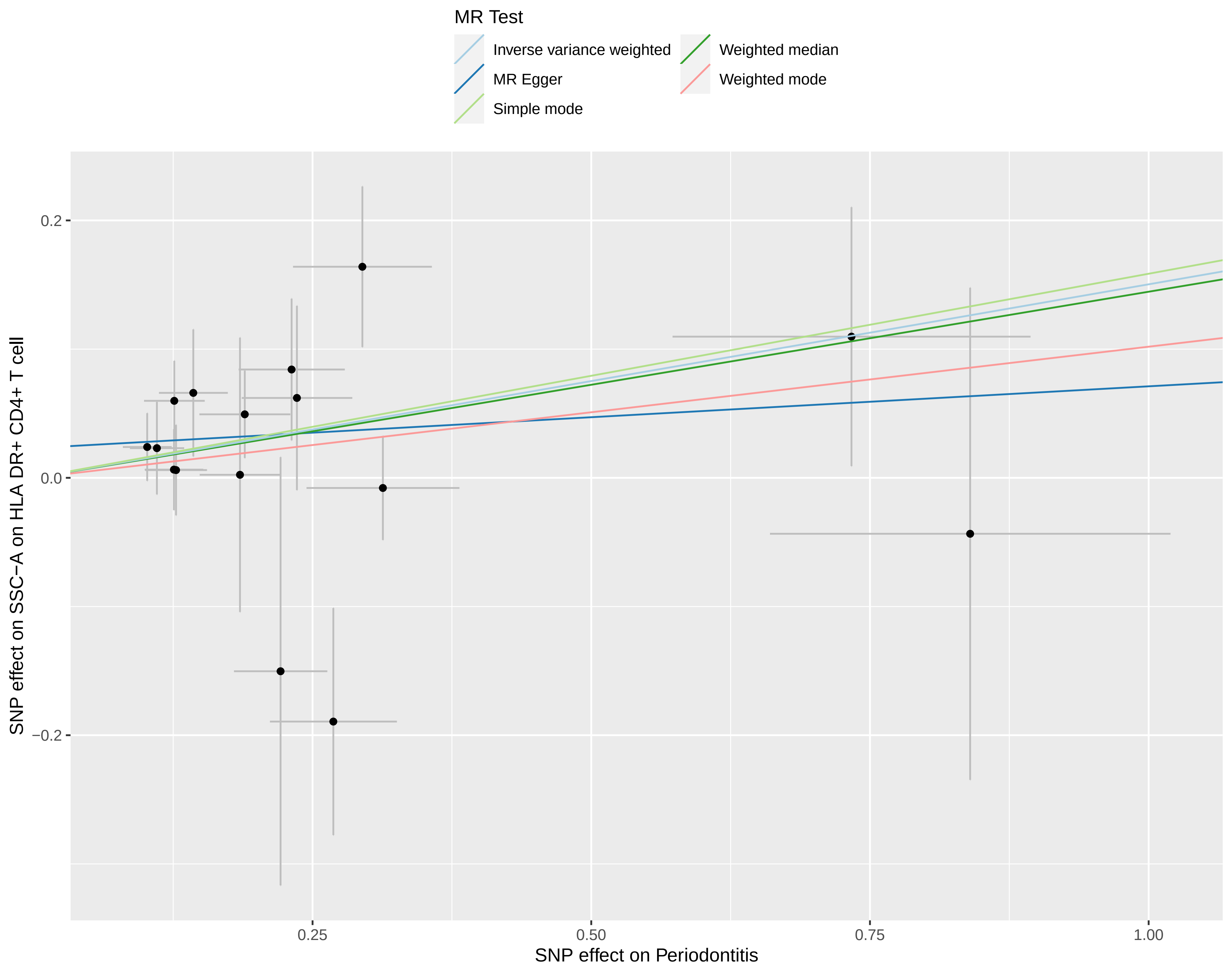

MR Test
Inverse variance weighted
MR Egger
Simple mode
Weighted median
Weighted mode
0.2
0.0
−0.2
0.25
0.50
0.75
1.00
SNP effect on SSC−A on HLA DR+ CD4+ T cell
SNP effect on Periodontitis

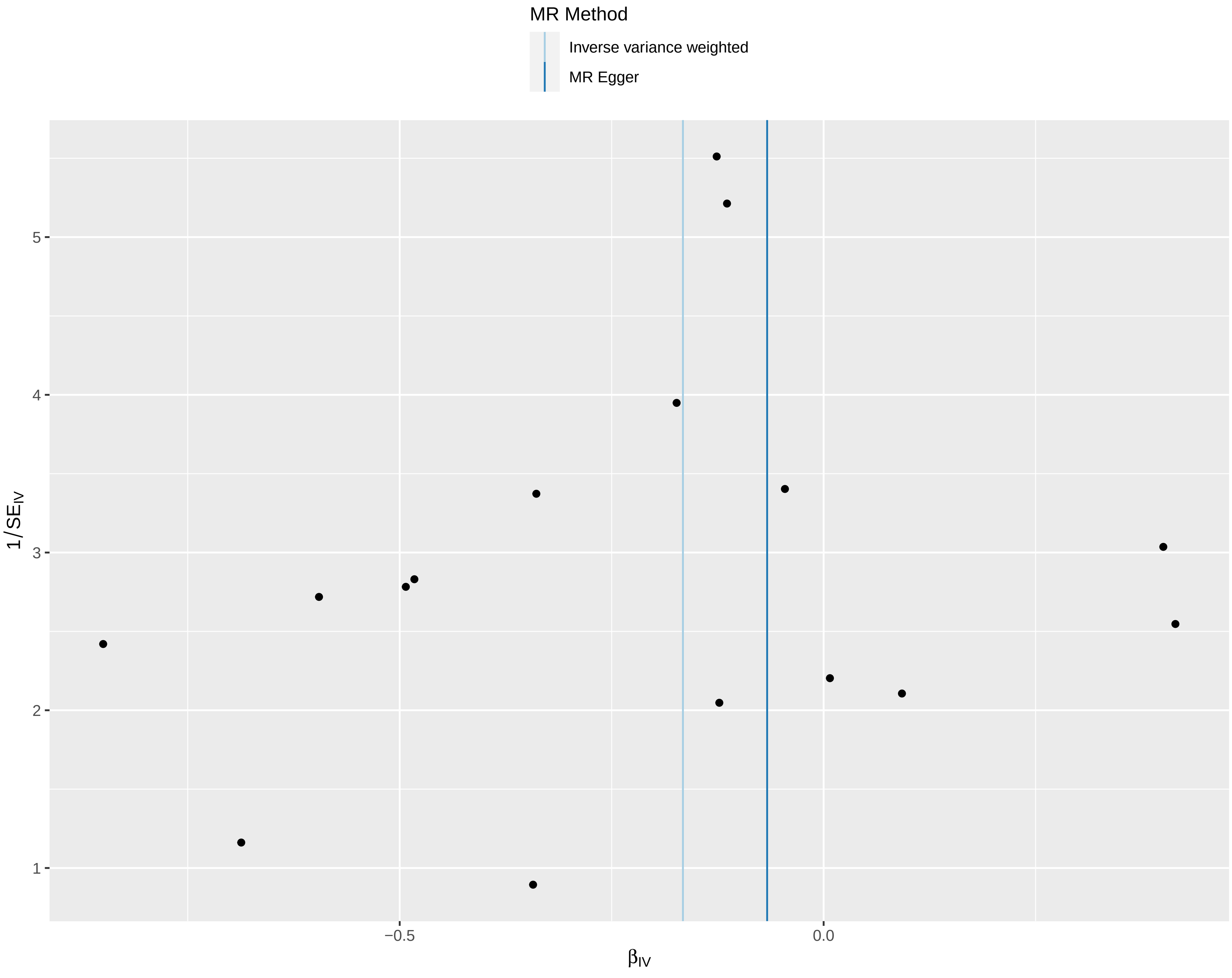

MR Method
Inverse variance weighted
MR Egger
5
4
3
2
1
1/SE_IV
−0.5
0.0
β_IV

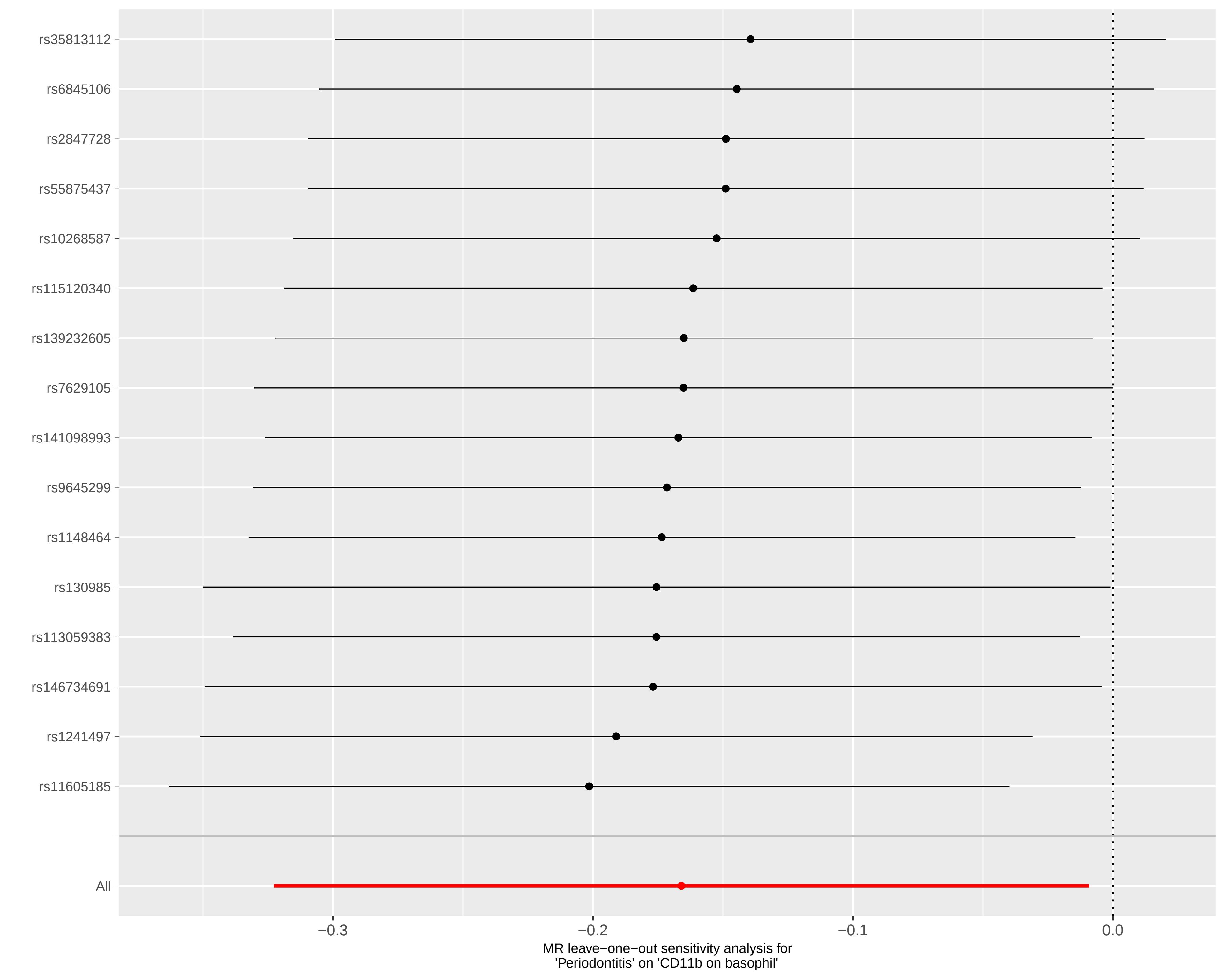
rs35813112
rs6845106
rs2847728
rs55875437
rs10268587
rs115120340
rs139232605
rs7629105
rs141098993
rs9645299
rs1148464
rs130985
rs113059383
rs146734691
rs1241497
rs11605185
All
−0.3
−0.2
−0.1
0.0
MR leave−one−out sensitivity analysis for
'Periodontitis' on 'CD11b on basophil'

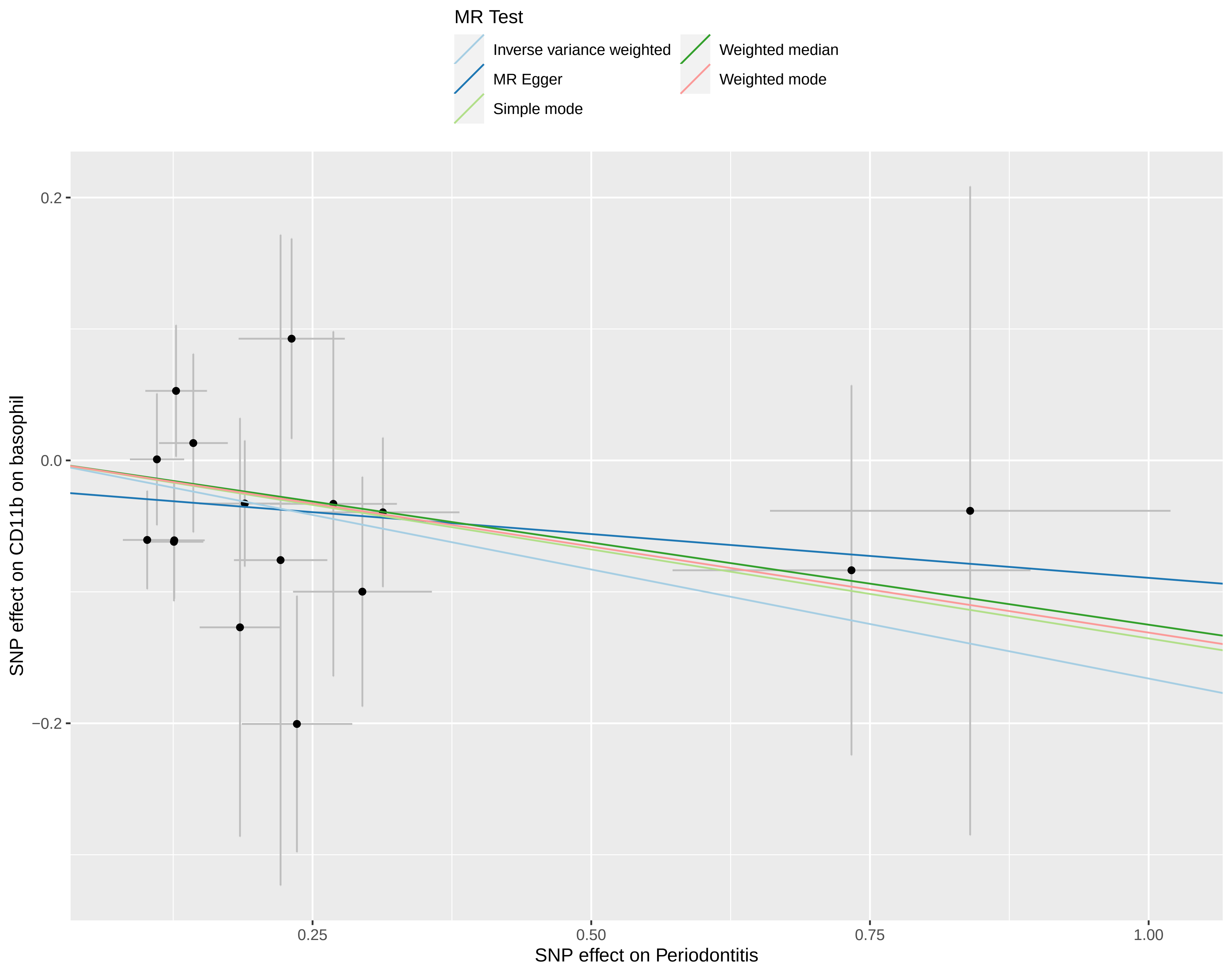

MR Test
Inverse variance weighted
MR Egger
Simple mode
Weighted median
Weighted mode
0.2
0.0
−0.2
0.25
0.50
0.75
1.00
SNP effect on CD11b on basophil
SNP effect on Periodontitis

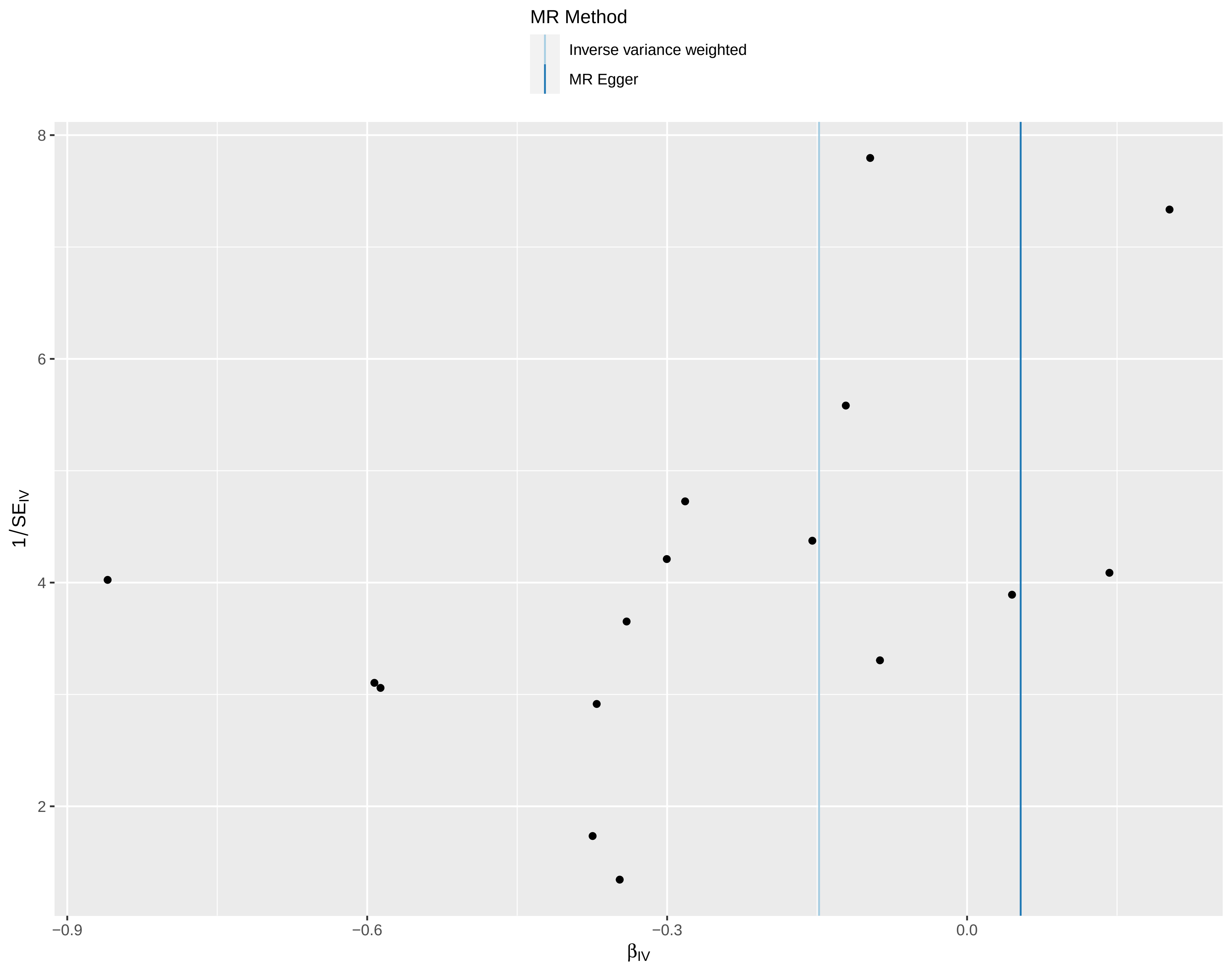

MR Method
Inverse variance weighted
MR Egger
8
6
4
2
1/SE_IV
−0.9
−0.6
−0.3
0.0
β_IV

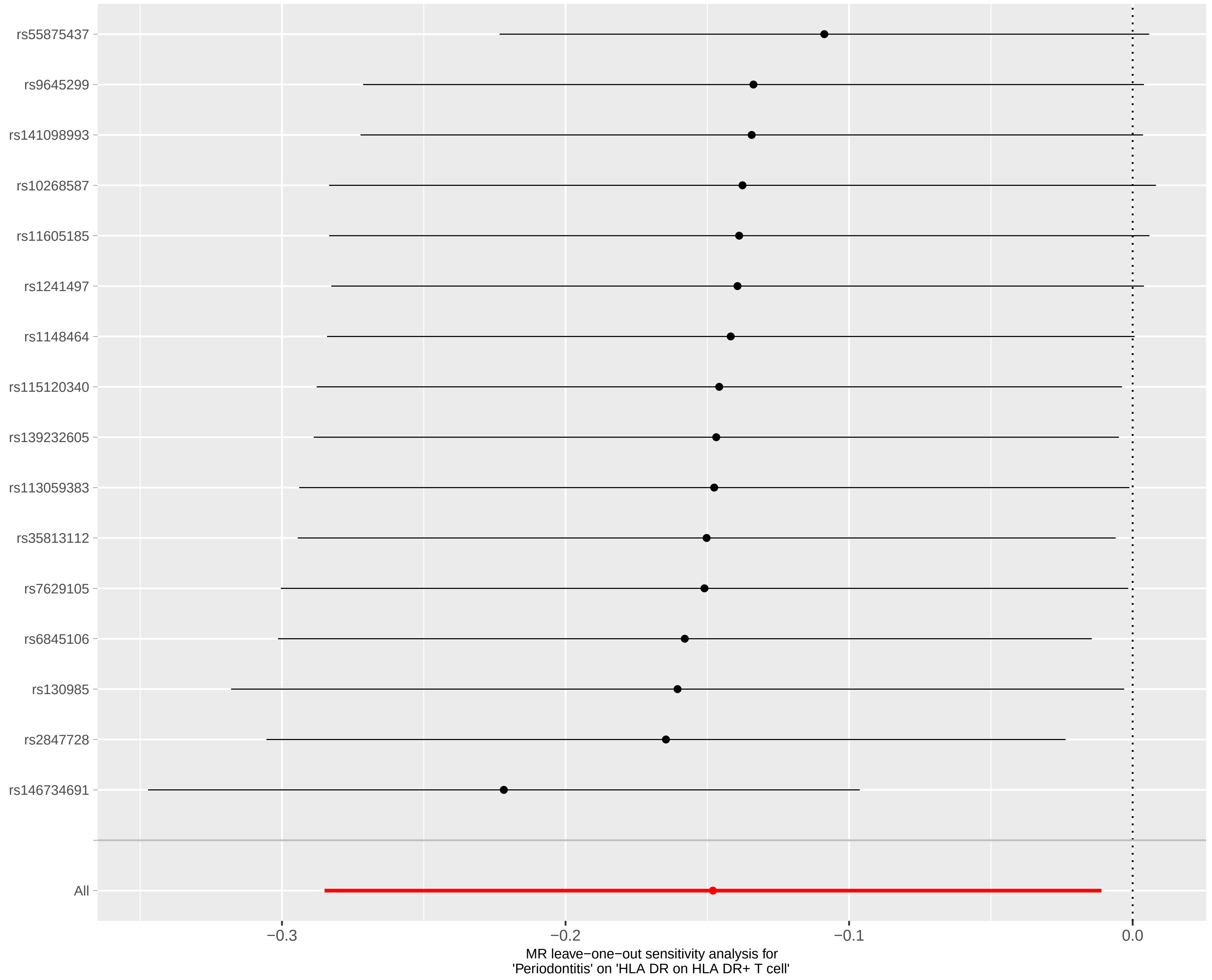
rs55875437
rs9645299
rs141098993
rs10268587
rs11605185
rs1241497
rs1148464
rs115120340
rs139232605
rs113059383
rs35813112
rs7629105
rs6845106
rs130985
rs2847728
rs146734691
All
−0.3
−0.2
−0.1
0.0
MR leave−one−out sensitivity analysis for
'Periodontitis' on 'HLA DR on HLA DR+ T cell'

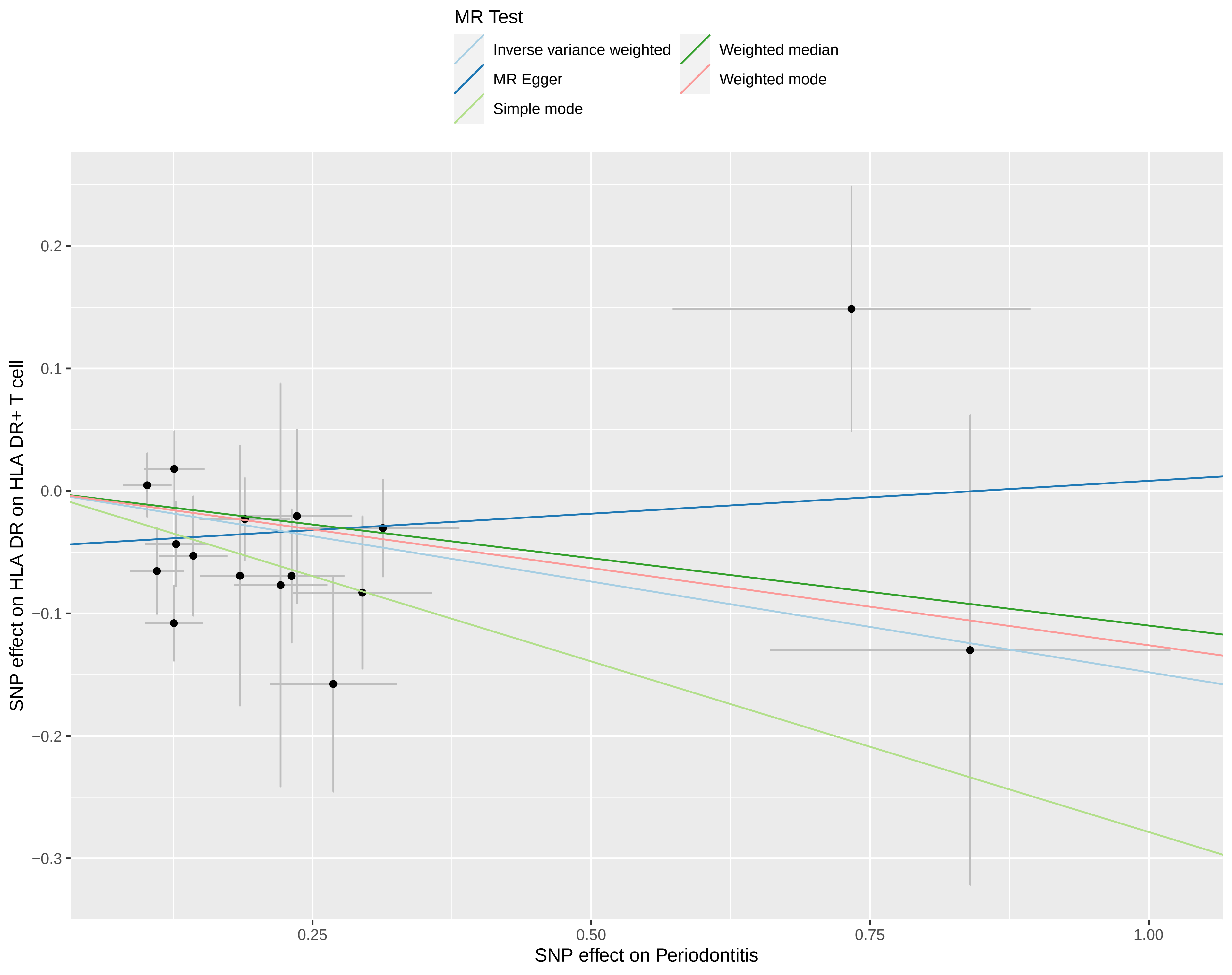
MR Test
Inverse variance weighted
MR Egger
Simple mode
Weighted median
Weighted mode
SNP effect on HLA DR on HLA DR+ T cell
0.2
0.1
0.0
−0.1
−0.2
−0.3
0.25
0.50
0.75
1.00
SNP effect on Periodontitis
